# Supplementary figures and images for: Optimizing CMV therapy: Population pharmacokinetics and Monte Carlo simulations for letermovir and maribavir dosage
Source: PLoS One. 2025 Apr 28;20(4):e0321180. doi: 10.1371/journal.pone.0321180 (PMC12036903; doi:10.1371/journal.pone.0321180)

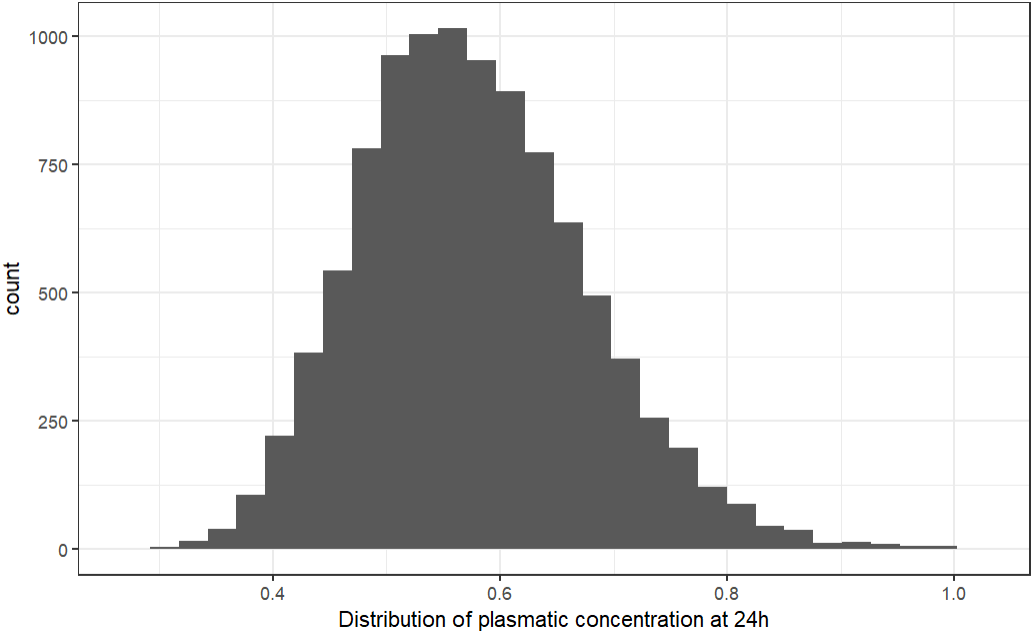

Supplement: S1 Fig — (TIF) [file pone.0321180.s001.tif]

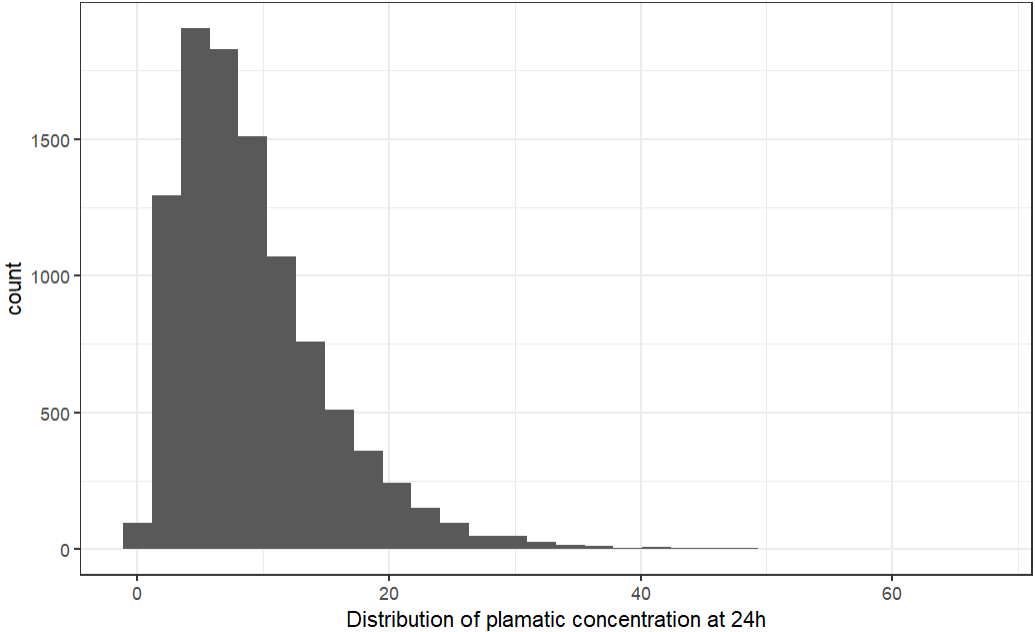

Supplement: S2 Fig — (TIF) [file pone.0321180.s002.tif]
