# Supplementary material for: Optimizing CMV therapy: Population pharmacokinetics and Monte Carlo simulations for letermovir and maribavir dosage
Source: PLoS One. 2025 Apr 28;20(4):e0321180. doi: 10.1371/journal.pone.0321180 (PMC12036903; doi:10.1371/journal.pone.0321180)
Supplement: S1 Table — (PDF) [file pone.0321180.s003.pdf]

Part 1

Days of treatments	Without loading doses						Loading dose D1					
	P. O			I.V			P. O			I.V		
	Mean ±sd	Geometric Mean	Median (Q1-Q3)	Mean ±sd	Geometric Mean	Median (Q1-Q3)	Mean ±sd	Geometric Mean	Median (Q1-Q3)	Mean ±sd	Geometric Mean	Median (Q1-Q3)
D1	453±79,1	446	447 (396-503)	460±81,1	453	454 (402-511)	905±157	892	894 (793-1000)	920±161	906	908 (805-1020)
D2	547±93,7	539	539 (479-604)	554±94,7	546	545 (487-612)	641±110	632	633 (563-707)	649±112	639	640 (571-718)
D3	580±98,2	571	572 (512-641)	590±103	581	580 (518-654)	613±104	604	606 (539-677)	623±108	614	611 (547-691)
D4	591±101	582	582 (520-655)	599±102	590	591 (526-663)	603±103	595	594 (532-668)	613±105	604	606 (538-679)
D5	596±101	587	588 (526-659)	604±103	595	596 (531-667)	600±102	591	591 (528-662)	611±106	602	601 (536-675)
D6	597±101	589	590 (526-661)	607±104	598	598 (534-672)	599±101	591	593 (528-663)	608±105	599	599 (536-672)
D7	598±102	590	589 (527-661)	607±104	598	598 (533-670)	599±102	591	590 (527-661)	606±104	597	597 (533-671)
D8	598±102	590	590 (526-662)	607±105	598	598 (535-671)	598±103	590	590 (527-661)	607±104	598	598 (533-671)
D9	599±103	590	590 (527-661)	607±104	598	598 (533-672)	599±103	590	590 (525-662)	608±103	599	599 (536-670)
D10	600±102	592	592 (529-662)	608±105	599	600 (534-674)	600±102	591	590 (528-662)	608±104	600	601 (534-673)

Part 2

Days of treatments	Loading doses D1-D2						Loading doses D1-D2-D3					
	P. O			I.V			P. O			I.V		
	Mean ±sd	Geometric Mean	Median (Q1-Q3)	Mean ±sd	Geometric Mean	Median (Q1-Q3)	Mean ±sd	Geometric Mean	Median (Q1-Q3)	Mean ±sd	Geometric Mean	Median (Q1-Q3)
D1	905±157	892	894 (793-1000)	920±161	906	908 (805-1020)	905±157	892	894 (793-1000)	920±161	906	908 (805-1020)
D2	1009±187	1008	1008 (960-1200)	1110±190	1090	1090 (973-1220)	1009±187	1008	1008 (960-1200)	1110±190	1090	1090 (973-1220)
D3	708±122	697	700 (622-783)	719±127	708	706 (629-798)	1160±195	1140	1150 (1020-1280)	1180±204	1160	1160 (1040-1310)
D4	636±110	627	626 (560-704)	646±113	636	639 (566-716)	731±128	720	718 (641-811)	742±131	730	734 (649-823)
D5	612±105	603	604 (538-677)	623±109	614	613 (546-689)	645±112	635	636 (565-714)	656±117	646	645 (574-727)
D6	604±102	596	597 (532-668)	613±106	604	604 (540-678)	617±105	608	609 (542-682)	625±109	616	616 (550-693)
D7	601±102	592	592 (529-663)	608±105	599	599 (535-673)	606±103	597	597 (533-669)	613±106	604	604 (538-679)
D8	598±103	590	590 (525-662)	607±104	598	598 (533-671)	598±103	590	590 (525-662)	607±104	598	598 (533-671)
D9	599±103	590	590 (525-662)	608±103	599	599 (536-670)	599±103	590	590 (525-662)	608±103	599	599 (536-670)
D10	600±102	591	591 (528-662)	608±104	600	601 (534-673)	600±102	591	591 (528-662)	608±103	600	601 (534-673)
