# Supplementary material for: Optimizing CMV therapy: Population pharmacokinetics and Monte Carlo simulations for letermovir and maribavir dosage
Source: PLoS One. 2025 Apr 28;20(4):e0321180. doi: 10.1371/journal.pone.0321180 (PMC12036903; doi:10.1371/journal.pone.0321180)
Supplement: S2 Table — (PDF) [file pone.0321180.s004.pdf]

Part 1

Days of treatments	Without loading doses						Loading dose D1					
	P. O			I.V			P. O			I.V		
	Mean ±sd	Geometric Mean	Median (Q1-Q3)	Mean ±sd	Geometric Mean	Median (Q1-Q3)	Mean ±sd	Geometric Mean	Median (Q1-Q3)	Mean ±sd	Geometric Mean	Median (Q1-Q3)
D1	893±53,2	892	893 (857-930)	923±54,1	922	923 (886-960)	1790±105	1780	1790 (1710-1860)	1850±107	1840	1850 (1770-1920)
D2	1120±49,8	1120	1130 (1009-1160)	1160±50,7	1160	1160 (1130-1200)	1360±64,7	1360	1370 (1330-1390)	1400±47,8	1400	1400 (1370-1430)
D3	1220±34,4	1210	1220 (1190-1240)	1250±35,8	1250	1260 (1230-1280)	1310±22,3	1310	1310 (1290-1320)	1340±23,7	1340	1350 (1330-1360)
D4	1250±23,3	1250	1260 (1240-1270)	1290±24,9	1290	1290 (1270-1310)	1290±17,8	1290	1290 (1280-1300)	1330±17,9	1330	1330 (1320-1340)
D5	1270±18,5	1270	1270 (1260-1280)	1310±19,4	1310	1310 (1290-1320)	1290±16,9	1290	1290 (1270-1300)	1320±17,1	1320	1320 (1310-1330)
D6	1280±16,9	1280	1280 (1270-1290)	1310±17,2	1310	1310 (130-1320)	1280±16,5	1280	1280 (1270-1300)	1320±16,9	1320	1320 (1310-1330)
D7	1280±16,6	1280	1280 (1270-1290)	1320±16,7	1320	1320 (1310-1330)	1280±16,4	1280	1280 (1270-1290)	1320±16,8	1320	1320 (1310-1330)
D8	1280±16,9	1280	1280 (1270-1290)	1320±16,7	1320	1320 (1310-1330)	1280±16,8	1280	1280 (1270-1290)	1320±16,6	1320	1320 (1310-1330)
D9	1280±16,6	1280	1280 (1270-1290)	1320±16,9	1320	1320 (1310-1330)	1280±16,6	1280	1280 (1270-1290)	1320±17	1320	1320 (1310-1330)
D10	1280±16,4	1280	1280 (1270-1290)	1320±16,7	1320	1320 (1310-1330)	1280±16,8	1280	1280 (1270-1290)	1320±16,7	1320	1320 (1310-1330)

Part 2

Days of treatments	Loading doses D1-D2						Loading doses D1-D2-D3					
	P. O			I.V			P. O			I.V		
	Mean ±sd	Geometric Mean	Median (Q1-Q3)	Mean ±sd	Geometric Mean	Median (Q1-Q3)	Mean ±sd	Geometric Mean	Median (Q1-Q3)	Mean ±sd	Geometric Mean	Median (Q1-Q3)
D1	1790±105	1780	1790 (1710-1860)	1850±107	1840	1850 (1770-1920)	1790±105	1780	1790 (1710-1860)	1850±107	1840	1850 (1770-1920)
D2	2250±98,4	2250	2260 (219-2320)	2320±101	2320	2330 (2250-2390)	2250±98,4	2250	2260 (2190-2320)	2320±101	2320	2330 (2250-2390)
D3	1540±25,2	1540	1540 (1520-1550)	1580±26,5	1580	1580 (1560-1600)	2430±65,6	2430	2440 (2390-2480)	2500±69,1	2500	2510 (2460-2550)
D4	1380±25	1380	1380 (1370-1400)	1420±24,1	1420	1420 (1400-1440)	1610±32,5	1610	1620 (1600-1640)	1660±31,1	1660	1660 (1640-1680)
D5	1320±22,4	1320	1320 (1310-1340)	1360±22,1	1360	1360 (1350-1380)	1410±36,8	1410	1420 (1390-1440)	1450±36,4	1450	1460 (1430-1480)
D6	1300±19,6	1300	1300 (1290-1310)	1340±19,4	1340	1340 (1320-1350)	1340±29,6	1340	1340 (1320-1360)	1380±29,1	1380	1380 (1360-1400)
D7	1290±17,8	1290	1290 (1280-1300)	1333±18	1330	1330 (1320-1340)	1310±22,7	1310	1310 (1290-1320)	1340±22,7	1340	1340 (1330-1360)
D8	1280±16,8	1280	1280 (1270-1290)	1320±16,6	1320	1320 (1310-1330)	1280±16,8	1280	1280 (1270-1290)	1320±16,6	1320	1320 (1310-1330)
D9	1280±16,6	1280	1280 (1270-1290)	1320±17	1320	1320 (1310-1330)	1280±16,6	1280	1280 (1270-1290)	1320±17	1320	1320 (1310-1330)
D10	1280±16,8	1280	1280 (1270-1290)	1320±16,7	1320	1320 (1310-1330)	1280±16,8	1280	1280 (1270-1290)	1320±16,7	1320	1320 (1310-1330)
