# Supplementary material for: Optimizing CMV therapy: Population pharmacokinetics and Monte Carlo simulations for letermovir and maribavir dosage
Source: PLoS One. 2025 Apr 28;20(4):e0321180. doi: 10.1371/journal.pone.0321180 (PMC12036903; doi:10.1371/journal.pone.0321180)
Supplement: S1 File — (HTML) [file pone.0321180.s005.html]

Maribavir


# Maribavir

#### Hamza

#### 30\_10\_2023

##Loading of the packages

```
library(mrgsolve)
```

```
## 
## Attaching package: 'mrgsolve'
```

```
## The following object is masked from 'package:stats':
## 
##     filter
```

```
library(tidyverse)
```

```
## Warning: package 'ggplot2' was built under R version 4.3.3
```

```
## ── Attaching core tidyverse packages ──────────────────────── tidyverse 2.0.0 ──
## ✔ dplyr     1.1.2     ✔ readr     2.1.4
## ✔ forcats   1.0.0     ✔ stringr   1.5.0
## ✔ ggplot2   3.5.0     ✔ tibble    3.2.1
## ✔ lubridate 1.9.2     ✔ tidyr     1.3.0
## ✔ purrr     1.0.2
```

```
## ── Conflicts ────────────────────────────────────────── tidyverse_conflicts() ──
## ✖ dplyr::filter() masks mrgsolve::filter(), stats::filter()
## ✖ dplyr::lag()    masks stats::lag()
## ℹ Use the conflicted package (<http://conflicted.r-lib.org/>) to force all conflicts to become errors
```

```
library(truncnorm)
library(ggplot2)
library(dplyr)
library(Pmetrics)
```

```
## 
## Attaching package: 'Pmetrics'
## 
## The following object is masked from 'package:stringr':
## 
##     fixed
```

#Modèle PKPOP Population pharmacokinetic modeling and simulation of
maribavir to support dose selection and regulatory approval in
adolescents with posttransplant refractory cytomegalovirus, K. Sun et
al, https://pubmed.ncbi.nlm.nih.gov/36789522/

```
code <- "
[SET] end=100, delta=0.1

[PARAM] @annotated
WTBL : 70 : Baseline weight (kg)
DOSE : 800 : dose classique (mg)
CYP3AINH : 0 : effect on CL inhibition(1) non inhibition(0)
CYP3AIND : 0 : effect on CL inhibition(1) non inhibition(0)
HSCMV : 1  : CMV + in HCT or SOT 

WTV2 : 0.406659 : effect of WT on V2
WTQ : 1.9488 : effect of WT on Q
WTV3 : 0.662937 : effect of WT on Vp
doseKA : -2.087 :effect of dose on KA
WTCL : 0.113898 : effect of WT on CL
CMVCL : -0.281686 : effect of CMV on CL
CYP3AINHCL : 0.70135 : effect of CYP3AINH on CL
CYP3AINDCL : 2.24497 : effect of CYP3AIND on CL

TVCL : 1.36003 : typical value of ln_CL 
TVV2 : 2.94463 : typical value of ln_V2
TVQ : -0.191013 : typical value of ln_Q 
TVV3 : 2.14074 : typical value of ln_V3
TVKA : -1.20083 : typical value of ln_KA
ALAG1 : -1.33168 : typical value of ln_ALAG1

[CMT] @annotated
GUT  : Dosing compartment (mg)
CENT : Central comaprtment (mg)
PERI : Peripheral compartment (mg)


[MAIN] 
double CLWT = log(WTBL/70) * (WTCL);
double VCWT = log(WTBL/70) * (WTV2);
double QWT = log(WTBL/70) * (WTQ);
double VPWT = log(WTBL/70) * (WTV3);
double KADOSE = pow((DOSE/800), doseKA);
double CYP3AINH_value = (CYP3AINH == 1) ? 1.0 : 0.0;
double CYP3AIND_value = (CYP3AIND== 1) ? 1.0 : 0.0;

double MU_1 = TVCL+ CLWT + CMVCL * HSCMV;
double CL = exp(MU_1 + ETACL) * pow(CYP3AINHCL, CYP3AINH_value) * pow(CYP3AINDCL, CYP3AIND_value); 

double MU_2 = TVV2 + VCWT;
double V2 = exp(MU_2 + ETAV2);

double MU_3 = TVQ + QWT;
double Q = exp(MU_3 + ETAQ);

double MU_4 = TVV3 + VPWT;
double V3 = exp(MU_4 + ETAV3);

double KA = exp(TVKA + ETAKA) * KADOSE;

double ALAG_gut = exp(ALAG1 + ETAALAG1);


[OMEGA] @annotated @block
ETACL : 0.223122 : ETA on clearance
ETAV2 : 0.121074 0.107449 : ETA on V2
ETAQ : -0.118376 -0.0525969 0.574004 : ETA on Q
ETAV3 : 0.0354346 0.0580477 0.442099 0.711749 : ETA on V3
ETAKA : 0.13287 0.172609  -0.500902 -0.391298 1.32526 : ETA on KA
ETAALAG1 : -0.0251937 -0.0388526 0.104635 0.0538585 -0.273076 0.14367 : ETA on ALAG1


[SIGMA] 
EPS(1) :0.0001 : proportionnal Residual unexplained variability 0.0671655
EPS(2) : 0 :  additive Residual unexplained variability


[ODE]
 dxdt_GUT = ALAG_gut - (KA * GUT);
 dxdt_CENT = (KA * GUT) + (Q * (PERI/V3)) - (CL * (CENT/V2)) - (Q * (CENT/V2)); 
 dxdt_PERI = (Q * (CENT/V2)) - (Q * (PERI/V3));


[TABLE] 
capture CP = (CENT/V2) *(1 + EPS(1)) + EPS(2);
int i = 0;
while(CP<0 && i <100) {
simeps();
CP = (CENT/V2) *(1 + EPS(1)) + EPS(2);
++i;
}
" 


# Compilation
my_model <- mcode("marib_model",code)
```

```
## Building marib_model ... done.
```

### 400mg/12h

##Graph 10 patients

```
ev1 <-  ev(ID = 1:10, amt = 400, ii=12, cmt = 1, addl=4 , ss=1)
data_ev_test <- as_tibble(ev1) %>% arrange(ID) 

set.seed(1234)
out_test <- my_model %>% 
 data_set(data_ev_test) %>%
  Req(CP) %>%
  mrgsim(end = 48, delta = 1)

as_tibble(out_test) %>% filter(between(time, 0,48)) %>%
    ggplot(aes(x = time, y = CP, color = as.factor(ID))) + 
  geom_point(show.legend = FALSE) + 
  geom_line(show.legend = FALSE) +
  theme(legend.position = "none")+ 
  labs(x = "Time (h)", y = "Maribavir concentration (mg/L)") + 
  theme_bw()
```

## 10 000 simulations

```
data_marib <- expand.ev(ID = 1:10000, amt = 400, ii=12, addl=1, ss=1)
data2 <- data_marib %>% mutate( WT =rtruncnorm(n(),a=50, b=120, mean=75, sd=10 )) 
set.seed(23456)

sim_marib <- my_model %>% 
  data_set(data2) %>%
  Req(CP) %>%
  mrgsim(delta = 0.1, end = 24)
summary(sim_marib)
```

```
##        ID             time             CP        
##  Min.   :    1   Min.   : 0.00   Min.   : 0.000  
##  1st Qu.: 2501   1st Qu.: 5.90   1st Qu.: 7.792  
##  Median : 5000   Median :11.95   Median :11.430  
##  Mean   : 5000   Mean   :11.95   Mean   :12.654  
##  3rd Qu.: 7500   3rd Qu.:18.00   3rd Qu.:16.127  
##  Max.   :10000   Max.   :24.00   Max.   :65.600
```

#Cmax : Cmax µg/mL

```
#Moyenne arithmétique
cmax <- as_tibble(sim_marib) %>% 
  filter(between(time, 0, 12)) %>% 
  group_by(ID) %>% 
  slice_max(CP) %>%
  summarise(mean_CP = mean(CP)) 

overall_mean_cmax <- cmax %>%
  summarise(overall_mean_cmax = mean(mean_CP), sd(mean_CP)) 

print(cmax)
```

```
## # A tibble: 10,000 × 2
##       ID mean_CP
##    <dbl>   <dbl>
##  1     1   14.4 
##  2     2    9.17
##  3     3    9.13
##  4     4   13.8 
##  5     5   15.1 
##  6     6    7.40
##  7     7   20.4 
##  8     8   13.1 
##  9     9   13.9 
## 10    10   14.6 
## # ℹ 9,990 more rows
```

```
print(overall_mean_cmax)
```

```
## # A tibble: 1 × 2
##   overall_mean_cmax `sd(mean_CP)`
##               <dbl>         <dbl>
## 1              16.1          6.91
```

```
#Moyenne géométrique
cmax <- as_tibble(sim_marib) %>% 
  filter(between(time, 0, 12)) %>% 
  group_by(ID) %>% 
  slice_max(CP) %>%
  summarise(mean_CP = exp ( mean ( log (CP ) ) )  ) 

overall_mean_cmax_gm <- cmax %>%
  summarise(overall_mean_cmax = exp ( mean ( log (mean_CP ) ) )  )

print(cmax)
```

```
## # A tibble: 10,000 × 2
##       ID mean_CP
##    <dbl>   <dbl>
##  1     1   14.4 
##  2     2    9.17
##  3     3    9.13
##  4     4   13.8 
##  5     5   15.1 
##  6     6    7.40
##  7     7   20.4 
##  8     8   13.1 
##  9     9   13.9 
## 10    10   14.6 
## # ℹ 9,990 more rows
```

```
print(overall_mean_cmax_gm)
```

```
## # A tibble: 1 × 1
##   overall_mean_cmax
##               <dbl>
## 1              14.8
```

```
#Médianne
cmax <- as_tibble(sim_marib) %>% 
  filter(between(time, 0, 12)) %>% 
  group_by(ID) %>% 
  slice_max(CP) %>%
  summarise(mean_CP = median(CP)) 

overall_mean_cmax <- cmax %>%
  summarise(overall_mean_cmax = fivenum(mean_CP))
```

```
## Warning: Returning more (or less) than 1 row per `summarise()` group was deprecated in
## dplyr 1.1.0.
## ℹ Please use `reframe()` instead.
## ℹ When switching from `summarise()` to `reframe()`, remember that `reframe()`
##   always returns an ungrouped data frame and adjust accordingly.
## Call `lifecycle::last_lifecycle_warnings()` to see where this warning was
## generated.
```

```
print(cmax)
```

```
## # A tibble: 10,000 × 2
##       ID mean_CP
##    <dbl>   <dbl>
##  1     1   14.4 
##  2     2    9.17
##  3     3    9.13
##  4     4   13.8 
##  5     5   15.1 
##  6     6    7.40
##  7     7   20.4 
##  8     8   13.1 
##  9     9   13.9 
## 10    10   14.6 
## # ℹ 9,990 more rows
```

```
print(overall_mean_cmax)
```

```
## # A tibble: 5 × 1
##   overall_mean_cmax
##               <dbl>
## 1              2.75
## 2             11.3 
## 3             14.9 
## 4             19.7 
## 5             65.6
```

#C0 (through concentration, T=11.5h)

```
#Moyenne arithmétique
my_model %>% 
  data_set(data2) %>%
  Req(CP) %>%
  mrgsim(delta = 0.1, end = 144) %>% filter(time==11.5) %>%summarise (mean = mean(CP), sd(CP))
```

```
## # A tibble: 1 × 2
##    mean `sd(CP)`
##   <dbl>    <dbl>
## 1  9.15     6.21
```

```
#Moyenne géométrique
my_model %>% 
  data_set(data2) %>%
  Req(CP) %>%
  mrgsim(delta = 0.1, end = 144) %>% filter(time==11.5) %>%summarise(gm_CMin =  exp ( mean ( log (CP ) ) ) , n= n() )
```

```
## # A tibble: 1 × 2
##   gm_CMin     n
##     <dbl> <int>
## 1    7.31 10000
```

```
#Médiane
my_model %>% 
  data_set(data2) %>%
  Req(CP) %>%
  mrgsim(delta = 0.1, end = 144) %>% filter(time==11.5) %>%summarise (median = fivenum(CP))
```

```
## Warning: Returning more (or less) than 1 row per `summarise()` group was deprecated in
## dplyr 1.1.0.
## ℹ Please use `reframe()` instead.
## ℹ When switching from `summarise()` to `reframe()`, remember that `reframe()`
##   always returns an ungrouped data frame and adjust accordingly.
## Call `lifecycle::last_lifecycle_warnings()` to see where this warning was
## generated.
```

```
## # A tibble: 5 × 1
##   median
##    <dbl>
## 1  0.309
## 2  4.76 
## 3  7.69 
## 4 11.8  
## 5 77.3
```

###10 000 simulations : 400mg/8h

##Graph 10 patients

```
ev1 <-  ev(ID = 1:10, amt = 400, ii=8, cmt = 1, addl=4 , ss=1)
data_ev_test <- as_tibble(ev1) %>% arrange(ID) 

set.seed(1234)
out_test <- my_model %>% 
 data_set(data_ev_test) %>%
  Req(CP) %>%
  mrgsim(end = 48, delta = 1)

as_tibble(out_test) %>% filter(between(time, 0,48)) %>%
    ggplot(aes(x = time, y = CP, color = as.factor(ID))) + 
  geom_point(show.legend = FALSE) + 
  geom_line(show.legend = FALSE) +
  theme(legend.position = "none")+ 
  labs(x = "Time (h)", y = "Maribavir concentration (mg/L)") + 
  theme_bw()
```

```
data_marib <- expand.ev(ID = 1:10000, amt = 400, ii=8, addl=1, ss=1)
data2 <- data_marib %>% mutate( WT =rtruncnorm(n(),a=50, b=120, mean=75, sd=10 )) 
set.seed(23456)

sim_marib <- my_model %>% 
  data_set(data2) %>%
  Req(CP) %>%
  mrgsim(delta = 0.1, end = 24)
```

```
## Warning in (function (x, data, idata = no_idata_set(), carry_out = carry.out, : [steady_bolus] ID 1017 failed to reach steady state
##   ss_n: 500, ss_rtol: 1e-08, ss_atol: 1e-08
```

```
summary(sim_marib)
```

```
##        ID             time             CP        
##  Min.   :    1   Min.   : 0.00   Min.   : 0.000  
##  1st Qu.: 2501   1st Qu.: 5.90   1st Qu.: 9.474  
##  Median : 5000   Median :11.95   Median :14.820  
##  Mean   : 5000   Mean   :11.95   Mean   :16.564  
##  3rd Qu.: 7500   3rd Qu.:18.00   3rd Qu.:21.608  
##  Max.   :10000   Max.   :24.00   Max.   :90.491
```

#Cmax : Cmax µg/mL

```
#Moyenne arithmétique
cmax <- as_tibble(sim_marib) %>% 
  filter(between(time, 0, 8)) %>% 
  group_by(ID) %>% 
  slice_max(CP) %>%
  summarise(mean_CP = mean(CP)) 

overall_mean_cmax <- cmax %>%
  summarise(overall_mean_cmax = mean(mean_CP), sd(mean_CP)) 

print(cmax)
```

```
## # A tibble: 10,000 × 2
##       ID mean_CP
##    <dbl>   <dbl>
##  1     1   19.9 
##  2     2   12.6 
##  3     3   10.8 
##  4     4   15.9 
##  5     5   18.0 
##  6     6    9.00
##  7     7   28.1 
##  8     8   15.0 
##  9     9   19.0 
## 10    10   19.5 
## # ℹ 9,990 more rows
```

```
print(overall_mean_cmax)
```

```
## # A tibble: 1 × 2
##   overall_mean_cmax `sd(mean_CP)`
##               <dbl>         <dbl>
## 1              21.6          9.75
```

```
#Moyenne géométrique
cmax <- as_tibble(sim_marib) %>% 
  filter(between(time, 0, 8)) %>% 
  group_by(ID) %>% 
  slice_max(CP) %>%
  summarise(mean_CP = exp ( mean ( log (CP ) ) )  ) 

overall_mean_cmax_gm <- cmax %>%
  summarise(overall_mean_cmax = exp ( mean ( log (mean_CP ) ) )  )

print(cmax)
```

```
## # A tibble: 10,000 × 2
##       ID mean_CP
##    <dbl>   <dbl>
##  1     1   19.9 
##  2     2   12.6 
##  3     3   10.8 
##  4     4   15.9 
##  5     5   18.0 
##  6     6    9.00
##  7     7   28.1 
##  8     8   15.0 
##  9     9   19.0 
## 10    10   19.5 
## # ℹ 9,990 more rows
```

```
print(overall_mean_cmax_gm)
```

```
## # A tibble: 1 × 1
##   overall_mean_cmax
##               <dbl>
## 1              19.7
```

```
#Médianne
cmax <- as_tibble(sim_marib) %>% 
  filter(between(time, 0, 8)) %>% 
  group_by(ID) %>% 
  slice_max(CP) %>%
  summarise(mean_CP = median(CP)) 

overall_mean_cmax <- cmax %>%
  summarise(overall_mean_cmax = fivenum(mean_CP))
```

```
## Warning: Returning more (or less) than 1 row per `summarise()` group was deprecated in
## dplyr 1.1.0.
## ℹ Please use `reframe()` instead.
## ℹ When switching from `summarise()` to `reframe()`, remember that `reframe()`
##   always returns an ungrouped data frame and adjust accordingly.
## Call `lifecycle::last_lifecycle_warnings()` to see where this warning was
## generated.
```

```
print(cmax)
```

```
## # A tibble: 10,000 × 2
##       ID mean_CP
##    <dbl>   <dbl>
##  1     1   19.9 
##  2     2   12.6 
##  3     3   10.8 
##  4     4   15.9 
##  5     5   18.0 
##  6     6    9.00
##  7     7   28.1 
##  8     8   15.0 
##  9     9   19.0 
## 10    10   19.5 
## # ℹ 9,990 more rows
```

```
print(overall_mean_cmax)
```

```
## # A tibble: 5 × 1
##   overall_mean_cmax
##               <dbl>
## 1              3.77
## 2             14.8 
## 3             19.7 
## 4             26.4 
## 5             90.5
```

#C0 (juste avant la prochaine dose, soit à 7.5h)

```
#Moyenne arithmétique
my_model %>% 
  data_set(data2) %>%
  Req(CP) %>%
  mrgsim(delta = 0.1, end = 144) %>% filter(time==7.5) %>%summarise (mean = mean(CP), sd(CP))
```

```
## # A tibble: 1 × 2
##    mean `sd(CP)`
##   <dbl>    <dbl>
## 1  16.1     9.50
```

```
#Moyenne géométrique
my_model %>% 
  data_set(data2) %>%
  Req(CP) %>%
  mrgsim(delta = 0.1, end = 144) %>% filter(time==7.5) %>%summarise(gm_CMin =  exp ( mean ( log (CP ) ) ) , n= n() )
```

```
## Warning in (function (x, data, idata = no_idata_set(), carry_out = carry.out, : [steady_bolus] ID 2361 failed to reach steady state
##   ss_n: 500, ss_rtol: 1e-08, ss_atol: 1e-08
```

```
## # A tibble: 1 × 2
##   gm_CMin     n
##     <dbl> <int>
## 1    13.6 10000
```

```
#Médiane
my_model %>% 
  data_set(data2) %>%
  Req(CP) %>%
  mrgsim(delta = 0.1, end = 144) %>% filter(time==7.5) %>%summarise (median = fivenum(CP))
```

```
## Warning: Returning more (or less) than 1 row per `summarise()` group was deprecated in
## dplyr 1.1.0.
## ℹ Please use `reframe()` instead.
## ℹ When switching from `summarise()` to `reframe()`, remember that `reframe()`
##   always returns an ungrouped data frame and adjust accordingly.
## Call `lifecycle::last_lifecycle_warnings()` to see where this warning was
## generated.
```

```
## # A tibble: 5 × 1
##   median
##    <dbl>
## 1   1.15
## 2   9.43
## 3  14.0 
## 4  20.2 
## 5 117.
```

###10 000 simulations : 600mg/12h

##Graph 10 patients

```
ev1 <-  ev(ID = 1:10, amt = 600, ii=12, cmt = 1, addl=4 , ss=1)
data_ev_test <- as_tibble(ev1) %>% arrange(ID) 

set.seed(1234)
out_test <- my_model %>% 
 data_set(data_ev_test) %>%
  Req(CP) %>%
  mrgsim(end = 48, delta = 1)

as_tibble(out_test) %>% filter(between(time, 0,48)) %>%
    ggplot(aes(x = time, y = CP, color = as.factor(ID))) + 
  geom_point(show.legend = FALSE) + 
  geom_line(show.legend = FALSE) +
  theme(legend.position = "none")+ 
  labs(x = "Time (h)", y = "Maribavir concentration (mg/L)") + 
  theme_bw()
```

```
data_marib <- expand.ev(ID = 1:10000, amt = 600, ii=12, addl=1, ss=1)
data2 <- data_marib %>% mutate( WT =rtruncnorm(n(),a=50, b=120, mean=75, sd=10 )) 
set.seed(23456)

sim_marib <- my_model %>% 
  data_set(data2) %>%
  Req(CP) %>%
  mrgsim(delta = 0.1, end = 24)
summary(sim_marib)
```

```
##        ID             time             CP       
##  Min.   :    1   Min.   : 0.00   Min.   : 0.00  
##  1st Qu.: 2501   1st Qu.: 5.90   1st Qu.:11.65  
##  Median : 5000   Median :11.95   Median :17.10  
##  Mean   : 5000   Mean   :11.95   Mean   :18.93  
##  3rd Qu.: 7500   3rd Qu.:18.00   3rd Qu.:24.13  
##  Max.   :10000   Max.   :24.00   Max.   :98.26
```

#Cmax : Cmax µg/mL

```
#Moyenne arithmétique
cmax <- as_tibble(sim_marib) %>% 
  filter(between(time, 0, 12)) %>% 
  group_by(ID) %>% 
  slice_max(CP) %>%
  summarise(mean_CP = mean(CP)) 

overall_mean_cmax <- cmax %>%
  summarise(overall_mean_cmax = mean(mean_CP), sd(mean_CP)) 

print(cmax)
```

```
## # A tibble: 10,000 × 2
##       ID mean_CP
##    <dbl>   <dbl>
##  1     1    21.6
##  2     2    13.7
##  3     3    13.7
##  4     4    20.7
##  5     5    22.6
##  6     6    11.1
##  7     7    30.5
##  8     8    19.6
##  9     9    20.7
## 10    10    21.9
## # ℹ 9,990 more rows
```

```
print(overall_mean_cmax)
```

```
## # A tibble: 1 × 2
##   overall_mean_cmax `sd(mean_CP)`
##               <dbl>         <dbl>
## 1              24.2          10.3
```

```
#Moyenne géométrique
cmax <- as_tibble(sim_marib) %>% 
  filter(between(time, 0, 12)) %>% 
  group_by(ID) %>% 
  slice_max(CP) %>%
  summarise(mean_CP = exp ( mean ( log (CP ) ) )  ) 

overall_mean_cmax_gm <- cmax %>%
  summarise(overall_mean_cmax = exp ( mean ( log (mean_CP ) ) )  )

print(cmax)
```

```
## # A tibble: 10,000 × 2
##       ID mean_CP
##    <dbl>   <dbl>
##  1     1    21.6
##  2     2    13.7
##  3     3    13.7
##  4     4    20.7
##  5     5    22.6
##  6     6    11.1
##  7     7    30.5
##  8     8    19.6
##  9     9    20.7
## 10    10    21.9
## # ℹ 9,990 more rows
```

```
print(overall_mean_cmax_gm)
```

```
## # A tibble: 1 × 1
##   overall_mean_cmax
##               <dbl>
## 1              22.2
```

```
#Médianne
cmax <- as_tibble(sim_marib) %>% 
  filter(between(time, 0, 12)) %>% 
  group_by(ID) %>% 
  slice_max(CP) %>%
  summarise(mean_CP = median(CP)) 

overall_mean_cmax <- cmax %>%
  summarise(overall_mean_cmax = fivenum(mean_CP))
```

```
## Warning: Returning more (or less) than 1 row per `summarise()` group was deprecated in
## dplyr 1.1.0.
## ℹ Please use `reframe()` instead.
## ℹ When switching from `summarise()` to `reframe()`, remember that `reframe()`
##   always returns an ungrouped data frame and adjust accordingly.
## Call `lifecycle::last_lifecycle_warnings()` to see where this warning was
## generated.
```

```
print(cmax)
```

```
## # A tibble: 10,000 × 2
##       ID mean_CP
##    <dbl>   <dbl>
##  1     1    21.6
##  2     2    13.7
##  3     3    13.7
##  4     4    20.7
##  5     5    22.6
##  6     6    11.1
##  7     7    30.5
##  8     8    19.6
##  9     9    20.7
## 10    10    21.9
## # ℹ 9,990 more rows
```

```
print(overall_mean_cmax)
```

```
## # A tibble: 5 × 1
##   overall_mean_cmax
##               <dbl>
## 1              4.11
## 2             16.9 
## 3             22.3 
## 4             29.5 
## 5             98.3
```

#C0 (through concentrations : T=11.5h)

```
#Moyenne arithmétique
my_model %>% 
  data_set(data2) %>%
  Req(CP) %>%
  mrgsim(delta = 0.1, end = 144) %>% filter(time==11.5) %>%summarise (mean = mean(CP), sd(CP))
```

```
## # A tibble: 1 × 2
##    mean `sd(CP)`
##   <dbl>    <dbl>
## 1  13.7     9.29
```

```
#Moyenne géométrique
my_model %>% 
  data_set(data2) %>%
  Req(CP) %>%
  mrgsim(delta = 0.1, end = 144) %>% filter(time==11.5) %>%summarise(gm_CMin =  exp ( mean ( log (CP ) ) ) , n= n() )
```

```
## # A tibble: 1 × 2
##   gm_CMin     n
##     <dbl> <int>
## 1    10.9 10000
```

```
#Médiane
my_model %>% 
  data_set(data2) %>%
  Req(CP) %>%
  mrgsim(delta = 0.1, end = 144) %>% filter(time==11.5) %>%summarise (median = fivenum(CP))
```

```
## Warning: Returning more (or less) than 1 row per `summarise()` group was deprecated in
## dplyr 1.1.0.
## ℹ Please use `reframe()` instead.
## ℹ When switching from `summarise()` to `reframe()`, remember that `reframe()`
##   always returns an ungrouped data frame and adjust accordingly.
## Call `lifecycle::last_lifecycle_warnings()` to see where this warning was
## generated.
```

```
## # A tibble: 5 × 1
##    median
##     <dbl>
## 1   0.457
## 2   7.11 
## 3  11.5  
## 4  17.6  
## 5 115.
```

### 10 000 simulations : 800mg/12h

##Graph 10 patients

```
ev1 <-  ev(ID = 1:10, amt = 800, ii=12, cmt = 1, addl=4 , ss=1)
data_ev_test <- as_tibble(ev1) %>% arrange(ID) 

set.seed(1234)
out_test <- my_model %>% 
 data_set(data_ev_test) %>%
  Req(CP) %>%
  mrgsim(end = 48, delta = 1)

as_tibble(out_test) %>% filter(between(time, 0,48)) %>%
    ggplot(aes(x = time, y = CP, color = as.factor(ID))) + 
  geom_point(show.legend = FALSE) + 
  geom_line(show.legend = FALSE) +
  theme(legend.position = "none")+ 
  labs(x = "Time (h)", y = "Maribavir concentration (mg/L)") + 
  theme_bw()
```

```
data_marib <- expand.ev(ID = 1:10000, amt = 800, ii=12, addl=1, ss=1)
data2 <- data_marib %>% mutate( WT =rtruncnorm(n(),a=50, b=120,  75, sd=10 )) 
set.seed(23456)

sim_marib <- my_model %>% 
  data_set(data2) %>%
  Req(CP) %>%
  mrgsim(delta = 0.1, end = 24)
summary(sim_marib)
```

```
##        ID             time             CP        
##  Min.   :    1   Min.   : 0.00   Min.   :  0.00  
##  1st Qu.: 2501   1st Qu.: 5.90   1st Qu.: 15.51  
##  Median : 5000   Median :11.95   Median : 22.76  
##  Mean   : 5000   Mean   :11.95   Mean   : 25.20  
##  3rd Qu.: 7500   3rd Qu.:18.00   3rd Qu.: 32.12  
##  Max.   :10000   Max.   :24.00   Max.   :130.92
```

#Cmax : Cmax µg/mL

```
#Moyenne arithmétique
cmax <- as_tibble(sim_marib) %>% 
  filter(between(time, 0, 12)) %>% 
  group_by(ID) %>% 
  slice_max(CP) %>%
  summarise(mean_CP = mean(CP)) 

overall_mean_cmax <- cmax %>%
  summarise(overall_mean_cmax = mean(mean_CP), sd(mean_CP)) 

print(cmax)
```

```
## # A tibble: 10,000 × 2
##       ID mean_CP
##    <dbl>   <dbl>
##  1     1    28.8
##  2     2    18.3
##  3     3    18.2
##  4     4    27.7
##  5     5    30.0
##  6     6    14.8
##  7     7    40.6
##  8     8    26.1
##  9     9    27.6
## 10    10    29.1
## # ℹ 9,990 more rows
```

```
print(overall_mean_cmax)
```

```
## # A tibble: 1 × 2
##   overall_mean_cmax `sd(mean_CP)`
##               <dbl>         <dbl>
## 1              32.2          13.8
```

```
#Moyenne géométrique
cmax <- as_tibble(sim_marib) %>% 
  filter(between(time, 0, 12)) %>% 
  group_by(ID) %>% 
  slice_max(CP) %>%
  summarise(mean_CP = exp ( mean ( log (CP ) ) )  ) 

overall_mean_cmax_gm <- cmax %>%
  summarise(overall_mean_cmax = exp ( mean ( log (mean_CP ) ) )  )

print(cmax)
```

```
## # A tibble: 10,000 × 2
##       ID mean_CP
##    <dbl>   <dbl>
##  1     1    28.8
##  2     2    18.3
##  3     3    18.2
##  4     4    27.7
##  5     5    30.0
##  6     6    14.8
##  7     7    40.6
##  8     8    26.1
##  9     9    27.6
## 10    10    29.1
## # ℹ 9,990 more rows
```

```
print(overall_mean_cmax_gm)
```

```
## # A tibble: 1 × 1
##   overall_mean_cmax
##               <dbl>
## 1              29.6
```

```
#Médianne
cmax <- as_tibble(sim_marib) %>% 
  filter(between(time, 0, 12)) %>% 
  group_by(ID) %>% 
  slice_max(CP) %>%
  summarise(mean_CP = median(CP)) 

overall_mean_cmax <- cmax %>%
  summarise(overall_mean_cmax = fivenum(mean_CP))
```

```
## Warning: Returning more (or less) than 1 row per `summarise()` group was deprecated in
## dplyr 1.1.0.
## ℹ Please use `reframe()` instead.
## ℹ When switching from `summarise()` to `reframe()`, remember that `reframe()`
##   always returns an ungrouped data frame and adjust accordingly.
## Call `lifecycle::last_lifecycle_warnings()` to see where this warning was
## generated.
```

```
print(cmax)
```

```
## # A tibble: 10,000 × 2
##       ID mean_CP
##    <dbl>   <dbl>
##  1     1    28.8
##  2     2    18.3
##  3     3    18.2
##  4     4    27.7
##  5     5    30.0
##  6     6    14.8
##  7     7    40.6
##  8     8    26.1
##  9     9    27.6
## 10    10    29.1
## # ℹ 9,990 more rows
```

```
print(overall_mean_cmax)
```

```
## # A tibble: 5 × 1
##   overall_mean_cmax
##               <dbl>
## 1              5.48
## 2             22.5 
## 3             29.6 
## 4             39.2 
## 5            131.
```

#C0 (through concentration T= à 11.5h)

```
#Moyenne arithmétique
my_model %>% 
  data_set(data2) %>%
  Req(CP) %>%
  mrgsim(delta = 0.1, end = 144) %>% filter(time==11.5) %>%summarise (mean = mean(CP), sd(CP))
```

```
## # A tibble: 1 × 2
##    mean `sd(CP)`
##   <dbl>    <dbl>
## 1  18.2     12.4
```

```
#Moyenne géométrique
my_model %>% 
  data_set(data2) %>%
  Req(CP) %>%
  mrgsim(delta = 0.1, end = 144) %>% filter(time==11.5) %>%summarise(gm_CMin =  exp ( mean ( log (CP ) ) ) , n= n() )
```

```
## # A tibble: 1 × 2
##   gm_CMin     n
##     <dbl> <int>
## 1    14.5 10000
```

```
#Médiane
my_model %>% 
  data_set(data2) %>%
  Req(CP) %>%
  mrgsim(delta = 0.1, end = 144) %>% filter(time==11.5) %>%summarise (median = fivenum(CP))
```

```
## Warning: Returning more (or less) than 1 row per `summarise()` group was deprecated in
## dplyr 1.1.0.
## ℹ Please use `reframe()` instead.
## ℹ When switching from `summarise()` to `reframe()`, remember that `reframe()`
##   always returns an ungrouped data frame and adjust accordingly.
## Call `lifecycle::last_lifecycle_warnings()` to see where this warning was
## generated.
```

```
## # A tibble: 5 × 1
##    median
##     <dbl>
## 1   0.604
## 2   9.45 
## 3  15.3  
## 4  23.4  
## 5 153.
```

###10 000 simulations : 800mg/8h

##Graph 10 patients

```
ev1 <-  ev(ID = 1:10, amt = 800, ii=8, cmt = 1, addl=4 , ss=1)
data_ev_test <- as_tibble(ev1) %>% arrange(ID) 

set.seed(1234)
out_test <- my_model %>% 
 data_set(data_ev_test) %>%
  Req(CP) %>%
  mrgsim(end = 48, delta = 1)

as_tibble(out_test) %>% filter(between(time, 0,48)) %>%
    ggplot(aes(x = time, y = CP, color = as.factor(ID))) + 
  geom_point(show.legend = FALSE) + 
  geom_line(show.legend = FALSE) +
  theme(legend.position = "none")+ 
  labs(x = "Time (h)", y = "Maribavir concentration (mg/L)") + 
  theme_bw()
```

```
data_marib <- expand.ev(ID = 1:10000, amt = 800, ii=8, addl=1, ss=1)
data2 <- data_marib %>% mutate( WT =rtruncnorm(n(),a=50, b=120, mean=75, sd=10 )) 
set.seed(23456)

sim_marib <- my_model %>% 
  data_set(data2) %>%
  Req(CP) %>%
  mrgsim(delta = 0.1, end = 24)
```

```
## Warning in (function (x, data, idata = no_idata_set(), carry_out = carry.out, : [steady_bolus] ID 1017 failed to reach steady state
##   ss_n: 500, ss_rtol: 1e-08, ss_atol: 1e-08
```

```
summary(sim_marib)
```

```
##        ID             time             CP        
##  Min.   :    1   Min.   : 0.00   Min.   :  0.00  
##  1st Qu.: 2501   1st Qu.: 5.90   1st Qu.: 18.88  
##  Median : 5000   Median :11.95   Median : 29.55  
##  Mean   : 5000   Mean   :11.95   Mean   : 33.02  
##  3rd Qu.: 7500   3rd Qu.:18.00   3rd Qu.: 43.08  
##  Max.   :10000   Max.   :24.00   Max.   :180.39
```

#Cmax : Cmax µg/mL

```
#Moyenne arithmétique
cmax <- as_tibble(sim_marib) %>% 
  filter(between(time, 0, 8)) %>% 
  group_by(ID) %>% 
  slice_max(CP) %>%
  summarise(mean_CP = mean(CP)) 

overall_mean_cmax <- cmax %>%
  summarise(overall_mean_cmax = mean(mean_CP), sd(mean_CP)) 

print(cmax)
```

```
## # A tibble: 10,000 × 2
##       ID mean_CP
##    <dbl>   <dbl>
##  1     1    39.8
##  2     2    25.1
##  3     3    21.6
##  4     4    31.8
##  5     5    36.0
##  6     6    18.0
##  7     7    56.1
##  8     8    30.0
##  9     9    37.9
## 10    10    38.9
## # ℹ 9,990 more rows
```

```
print(overall_mean_cmax)
```

```
## # A tibble: 1 × 2
##   overall_mean_cmax `sd(mean_CP)`
##               <dbl>         <dbl>
## 1              43.1          19.4
```

```
#Moyenne géométrique
cmax <- as_tibble(sim_marib) %>% 
  filter(between(time, 0, 8)) %>% 
  group_by(ID) %>% 
  slice_max(CP) %>%
  summarise(mean_CP = exp ( mean ( log (CP ) ) )  ) 

overall_mean_cmax_gm <- cmax %>%
  summarise(overall_mean_cmax = exp ( mean ( log (mean_CP ) ) )  )

print(cmax)
```

```
## # A tibble: 10,000 × 2
##       ID mean_CP
##    <dbl>   <dbl>
##  1     1    39.8
##  2     2    25.1
##  3     3    21.6
##  4     4    31.8
##  5     5    36.0
##  6     6    18.0
##  7     7    56.1
##  8     8    30.0
##  9     9    37.9
## 10    10    38.9
## # ℹ 9,990 more rows
```

```
print(overall_mean_cmax_gm)
```

```
## # A tibble: 1 × 1
##   overall_mean_cmax
##               <dbl>
## 1              39.3
```

```
#Médianne
cmax <- as_tibble(sim_marib) %>% 
  filter(between(time, 0, 8)) %>% 
  group_by(ID) %>% 
  slice_max(CP) %>%
  summarise(mean_CP = median(CP)) 

overall_mean_cmax <- cmax %>%
  summarise(overall_mean_cmax = fivenum(mean_CP))
```

```
## Warning: Returning more (or less) than 1 row per `summarise()` group was deprecated in
## dplyr 1.1.0.
## ℹ Please use `reframe()` instead.
## ℹ When switching from `summarise()` to `reframe()`, remember that `reframe()`
##   always returns an ungrouped data frame and adjust accordingly.
## Call `lifecycle::last_lifecycle_warnings()` to see where this warning was
## generated.
```

```
print(cmax)
```

```
## # A tibble: 10,000 × 2
##       ID mean_CP
##    <dbl>   <dbl>
##  1     1    39.8
##  2     2    25.1
##  3     3    21.6
##  4     4    31.8
##  5     5    36.0
##  6     6    18.0
##  7     7    56.1
##  8     8    30.0
##  9     9    37.9
## 10    10    38.9
## # ℹ 9,990 more rows
```

```
print(overall_mean_cmax)
```

```
## # A tibble: 5 × 1
##   overall_mean_cmax
##               <dbl>
## 1              7.51
## 2             29.5 
## 3             39.2 
## 4             52.6 
## 5            180.
```

#C0 (Through concentration : T= 7.5h)

```
#Moyenne arithmétique
my_model %>% 
  data_set(data2) %>%
  Req(CP) %>%
  mrgsim(delta = 0.1, end = 144) %>% filter(time==7.5) %>%summarise (mean = mean(CP), sd(CP))
```

```
## # A tibble: 1 × 2
##    mean `sd(CP)`
##   <dbl>    <dbl>
## 1  32.1     18.9
```

```
#Moyenne géométrique
my_model %>% 
  data_set(data2) %>%
  Req(CP) %>%
  mrgsim(delta = 0.1, end = 144) %>% filter(time==7.5) %>%summarise(gm_CMin =  exp ( mean ( log (CP ) ) ) , n= n() )
```

```
## Warning in (function (x, data, idata = no_idata_set(), carry_out = carry.out, : [steady_bolus] ID 2361 failed to reach steady state
##   ss_n: 500, ss_rtol: 1e-08, ss_atol: 1e-08
```

```
## # A tibble: 1 × 2
##   gm_CMin     n
##     <dbl> <int>
## 1    27.2 10000
```

```
#Médiane
my_model %>% 
  data_set(data2) %>%
  Req(CP) %>%
  mrgsim(delta = 0.1, end = 144) %>% filter(time==7.5) %>%summarise (median = fivenum(CP))
```

```
## Warning: Returning more (or less) than 1 row per `summarise()` group was deprecated in
## dplyr 1.1.0.
## ℹ Please use `reframe()` instead.
## ℹ When switching from `summarise()` to `reframe()`, remember that `reframe()`
##   always returns an ungrouped data frame and adjust accordingly.
## Call `lifecycle::last_lifecycle_warnings()` to see where this warning was
## generated.
```

```
## # A tibble: 5 × 1
##   median
##    <dbl>
## 1   2.28
## 2  18.8 
## 3  27.9 
## 4  40.3 
## 5 233.
```

##PTA###

#######MARIBAVIR : missing dose : Resumption at normal dosage
##############

```
set.seed(1234)

e1 <- ev(ID = 1:10000, amt = 400,ii= 12,  ss=1)
e2 <- ev(ID = 1:10000, amt = 400, ii = 12,  ss=0)
efinal <- seq(e1, wait= 12 , e2)
data2 <- efinal %>% mutate( WT =rtruncnorm(n(),a=50, b=120, mean=75, sd=10 )) 
data_ev_test <- as_tibble(data2) %>% arrange(ID) 

sim_marib <- my_model %>% 
  data_set(data_ev_test) %>%
  Req(CP) %>%
  mrgsim(delta = 0.1, end =50 )
```

# Proportion of subjects with a residual concentration (Cmin) > CI50 : Cmin > 2.257 mg/L

```
n1 <- my_model %>% 
   data_set(data_ev_test) %>%
   Req(CP) %>%
   # zero_re() %>%
   mrgsim(end = 50, delta = 0.5) %>% filter(time==35.5) %>% summarise(prop_sup_2.257 = mean(CP>2.257)*100)
```

# Proportion of subjects with a residual concentration (Cmin) > moyenne range CI50 : Cmin > 6.4 mg/L

```
n2 <- my_model %>% 
   data_set(data_ev_test) %>%
   Req(CP) %>%
   # zero_re() %>%
   mrgsim(end = 50, delta = 0.5) %>% filter(time==35.5) %>% summarise(prop_sup_6.4 = mean(CP>6.4)*100)
```

# Proportion of subjects with a residual concentration (Cmin) > haut range CI50 : Cmin > 10.534 mg/L

```
n3 <- my_model %>% 
   data_set(data_ev_test) %>%
   Req(CP) %>%
   # zero_re() %>%
   mrgsim(end = 50, delta = 0.5) %>% filter(time==35.5) %>% summarise(prop_sup_10.534 = mean(CP>10.534)*100)
```

# Proportion of subjects with a residual concentration (Cmin) > bas range 5CI50 : Cmin > 11.285 mg/L

```
n4 <- my_model %>% 
   data_set(data_ev_test) %>%
   Req(CP) %>%
   # zero_re() %>%
   mrgsim(end = 50, delta = 0.5) %>% filter(time==35.5) %>% summarise(prop_sup_11.285 = mean(CP>11.285)*100)
```

# Proportion of subjects with a residual concentration (Cmin) > moyenne range 5CI50 : Cmin > 31.978 mg/L

```
n5 <- my_model %>% 
   data_set(data_ev_test) %>%
   Req(CP) %>%
   # zero_re() %>%
   mrgsim(end = 50, delta = 0.5) %>% filter(time==35.5) %>% summarise(prop_sup_31.978 = mean(CP>31.978)*100)
```

# Proportion of subjects with a residual concentration (Cmin) > haut range 5CI50 : Cmin > 52.67 mg/L

```
n6 <- my_model %>% 
   data_set(data_ev_test) %>%
   Req(CP) %>%
   # zero_re() %>%
   mrgsim(end = 50, delta = 0.5) %>% filter(time==35.5) %>% summarise(prop_sup_52.67 = mean(CP>52.67)*100)
```

```
## Warning in (function (x, data, idata = no_idata_set(), carry_out = carry.out, : [steady_bolus] ID 9405 failed to reach steady state
##   ss_n: 500, ss_rtol: 1e-08, ss_atol: 1e-08
```

#######MARIBAVIR : missing dose : Resumption at double dosage
############

```
set.seed(1234)

e1 <- ev(ID = 1:10000, amt = 400,ii= 12,  ss=1)
e2 <- ev(ID = 1:10000, amt = 800, ii = 12,  ss=0)
efinal <- seq(e1, wait= 12 , e2)
data2 <- efinal %>% mutate( WT =rtruncnorm(n(),a=50, b=120, mean=75, sd=10 )) 
data_ev_test <- as_tibble(data2) %>% arrange(ID) 

sim_marib <- my_model %>% 
  data_set(data_ev_test) %>%
  Req(CP) %>%
  mrgsim(delta = 0.1, end =50 )
```

# Proportion of subjects with a residual concentration (Cmin) > CI50 : Cmin > 2.257 mg/L

```
d1 <- my_model %>% 
   data_set(data_ev_test) %>%
   Req(CP) %>%
   # zero_re() %>%
   mrgsim(end = 50, delta = 0.5) %>% filter(time==35.5) %>% summarise(prop_sup_2.257 = mean(CP>2.257)*100)
```

# Proportion of subjects with a residual concentration (Cmin) > moyenne range CI50 : Cmin > 6.4 mg/L

```
d2 <- my_model %>% 
   data_set(data_ev_test) %>%
   Req(CP) %>%
   # zero_re() %>%
   mrgsim(end = 50, delta = 0.5) %>% filter(time==35.5) %>% summarise(prop_sup_6.4 = mean(CP>6.4)*100)
```

# Proportion of subjects with a residual concentration (Cmin) > haut range CI50 : Cmin > 10.534 mg/L

```
d3 <- my_model %>% 
   data_set(data_ev_test) %>%
   Req(CP) %>%
   # zero_re() %>%
   mrgsim(end = 50, delta = 0.5) %>% filter(time==35.5) %>% summarise(prop_sup_10.534 = mean(CP>10.534)*100)
```

# Proportion of subjects with a residual concentration (Cmin) > bas range 5CI50 : Cmin > 11.285 mg/L

```
d4 <- my_model %>% 
   data_set(data_ev_test) %>%
   Req(CP) %>%
   # zero_re() %>%
   mrgsim(end = 50, delta = 0.5) %>% filter(time==35.5) %>% summarise(prop_sup_11.285 = mean(CP>11.285)*100)
```

# Proportion of subjects with a residual concentration (Cmin) > moyenne range 5CI50 : Cmin > 31.978 mg/L

```
d5 <- my_model %>% 
   data_set(data_ev_test) %>%
   Req(CP) %>%
   # zero_re() %>%
   mrgsim(end = 50, delta = 0.5) %>% filter(time==35.5) %>% summarise(prop_sup_31.978 = mean(CP>31.978)*100)
```

# Proportion of subjects with a residual concentration (Cmin) > haut range 5CI50 : Cmin > 52.67 mg/L

```
d6 <- my_model %>% 
   data_set(data_ev_test) %>%
   Req(CP) %>%
   # zero_re() %>%
   mrgsim(end = 50, delta = 0.5) %>% filter(time==35.5) %>% summarise(prop_sup_52.67 = mean(CP>52.67)*100)
```

```
## Warning in (function (x, data, idata = no_idata_set(), carry_out = carry.out, : [steady_bolus] ID 9405 failed to reach steady state
##   ss_n: 500, ss_rtol: 1e-08, ss_atol: 1e-08
```

#######MARIBAVIR : missing dose : Resumption at 1.5xdosage
############

```
set.seed(1234)

e1 <- ev(ID = 1:10000, amt = 400,ii= 12,  ss=1)
e2 <- ev(ID = 1:10000, amt = 600, ii = 12,  ss=0)
efinal <- seq(e1, wait= 12 , e2)
data2 <- efinal %>% mutate( WT =rtruncnorm(n(),a=50, b=120, mean=75, sd=10 )) 
data_ev_test <- as_tibble(data2) %>% arrange(ID) 

sim_marib <- my_model %>% 
  data_set(data_ev_test) %>%
  Req(CP) %>%
  mrgsim(delta = 0.1, end =50 )
```

# Proportion of subjects with a residual concentration (Cmin) > CI50 : Cmin > 2.257 mg/L

```
m1 <- my_model %>% 
   data_set(data_ev_test) %>%
   Req(CP) %>%
   # zero_re() %>%
   mrgsim(end = 50, delta = 0.5) %>% filter(time==35.5) %>% summarise(prop_sup_2.257 = mean(CP>2.257)*100)
```

# Proportion of subjects with a residual concentration (Cmin) > moyenne range CI50 : Cmin > 6.4 mg/L

```
m2 <- my_model %>% 
   data_set(data_ev_test) %>%
   Req(CP) %>%
   # zero_re() %>%
   mrgsim(end = 50, delta = 0.5) %>% filter(time==35.5) %>% summarise(prop_sup_6.4 = mean(CP>6.4)*100)
```

# Proportion of subjects with a residual concentration (Cmin) > haut range CI50 : Cmin > 10.534 mg/L

```
m3 <- my_model %>% 
   data_set(data_ev_test) %>%
   Req(CP) %>%
   # zero_re() %>%
   mrgsim(end = 50, delta = 0.5) %>% filter(time==35.5) %>% summarise(prop_sup_10.534 = mean(CP>10.534)*100)
```

# Proportion of subjects with a residual concentration (Cmin) > bas range 5CI50 : Cmin > 11.285 mg/L

```
m4 <- my_model %>% 
   data_set(data_ev_test) %>%
   Req(CP) %>%
   # zero_re() %>%
   mrgsim(end = 50, delta = 0.5) %>% filter(time==35.5) %>% summarise(prop_sup_11.285 = mean(CP>11.285)*100)
```

# Proportion of subjects with a residual concentration (Cmin) > moyenne range 5CI50 : Cmin > 31.978 mg/L

```
m5 <- my_model %>% 
   data_set(data_ev_test) %>%
   Req(CP) %>%
   # zero_re() %>%
   mrgsim(end = 50, delta = 0.5) %>% filter(time==35.5) %>% summarise(prop_sup_31.978 = mean(CP>31.978)*100)
```

# Proportion of subjects with a residual concentration (Cmin) > haut range 5CI50 : Cmin > 52.67 mg/L

```
m6 <- my_model %>% 
   data_set(data_ev_test) %>%
   Req(CP) %>%
   # zero_re() %>%
   mrgsim(end = 50, delta = 0.5) %>% filter(time==35.5) %>% summarise(prop_sup_52.67 = mean(CP>52.67)*100)
```

```
## Warning in (function (x, data, idata = no_idata_set(), carry_out = carry.out, : [steady_bolus] ID 9405 failed to reach steady state
##   ss_n: 500, ss_rtol: 1e-08, ss_atol: 1e-08
```

```
library(dplyr)
library(ggplot2)
library(mrgsolve)
library(tidyr)
library(truncnorm)

# Initialisation des données et des modèles
set.seed(1234)

# Création des événements pour le modèle
create_data <- function(amt) {
  e1 <- ev(ID = 1:10000, amt = 400, ii = 12, ss = 1)
  e2 <- ev(ID = 1:10000, amt = amt, ii = 12, ss = 0)
  efinal <- as.data.frame(seq(e1, wait = 12, e2))
  efinal %>% mutate(WT = rtruncnorm(n(), a = 50, b = 120, mean = 75, sd = 10)) %>%
    arrange(ID)
}

# Fonction pour obtenir les proportions
get_proportion <- function(data, conc) {
  my_model %>% 
    data_set(data) %>%
    Req(CP) %>%
    mrgsim(end = 50, delta = 0.5) %>%
    filter(time == 35.5) %>%
    summarise(proportion = mean(CP > conc) * 100) %>%
    pull(proportion)
}

# Simulation pour différents scénarios
amt_list <- list(400, 800, 600)
names_list <- list("n", "d", "m")
concentrations <- c(2.257, 6.4, 10.534, 11.285, 31.978, 52.67)
results <- data.frame()

for (i in seq_along(amt_list)) {
  data_ev_test <- create_data(amt_list[[i]])
  proportions <- sapply(concentrations, function(c) get_proportion(data_ev_test, c))
  scenario <- rep(names_list[[i]], length(concentrations))
  results <- rbind(results, data.frame(Scenario = scenario, Concentration = concentrations, Proportion = proportions))
}

# Mapping des noms de scenario aux doses
results$Scenario <- recode(results$Scenario, n = "400 mg", d = "800 mg", m = "600 mg")

# Création du graphique
fig4 <- ggplot(results, aes(x = Concentration, y = Proportion, color = Scenario, group = Scenario)) +
  geom_point(size = 3) +
  geom_line() +
  geom_hline(yintercept = 90, color = "grey", linetype = "solid") +
  geom_vline(xintercept = 2.56, color = "blue", linetype = "dashed")  +
  geom_vline(xintercept = 10.53, color = "blue", linetype = "dashed")  +
  geom_vline(xintercept = 11.29, color = "red", linetype = "dashed")  +
  geom_vline(xintercept = 52.67, color = "red", linetype = "dashed")  +
  scale_y_continuous(limits = c(0, 100), breaks = seq(0, 100, 25)) +
  labs(title = "Probability of target attainment",
       x = "Inhibitory concentration targets (mg/L)",
       y = "Proportions with success (%)",
       color = "Dose (mg)") +
  theme_minimal() +
  theme(legend.position = "right") +
 scale_color_grey()

fig4
```

######PTA with different posologies####

##400/12h

```
set.seed(23456)
data_marib <- expand.ev(ID = 1:10000, amt = 400, ii=12, addl=1, ss=1)
data2_400_12 <- data_marib %>% mutate( WT =rtruncnorm(n(),a=50, b=120, mean=75, sd=10 ))
```

```
# Proportion of subjects with a residual concentration (Cmin) > CI50 : Cmin > 2.257 mg/L 
a1 <- my_model %>% 
   data_set(data2_400_12) %>%
   Req(CP) %>%
   # zero_re() %>%
   mrgsim(end = 50, delta = 0.5) %>% filter(time==11.5) %>% summarise(prop_sup_2.257 = mean(CP>2.257)*100)
```

# Proportion of subjects with a residual concentration (Cmin) > moyenne range CI50 : Cmin > 6.4 mg/L

```
a2 <- my_model %>% 
   data_set(data2_400_12) %>%
   Req(CP) %>%
   # zero_re() %>%
   mrgsim(end = 50, delta = 0.5) %>% filter(time==11.5) %>% summarise(prop_sup_6.4 = mean(CP>6.4)*100)
```

# Proportion of subjects with a residual concentration (Cmin) > haut range CI50 : Cmin > 10.534 mg/L

```
a3 <- my_model %>% 
   data_set(data2_400_12) %>%
   Req(CP) %>%
   # zero_re() %>%
   mrgsim(end = 50, delta = 0.5) %>% filter(time==11.5) %>% summarise(prop_sup_10.534 = mean(CP>10.534)*100)
```

# Proportion of subjects with a residual concentration (Cmin) > bas range 5CI50 : Cmin > 11.285 mg/L

```
a4 <- my_model %>% 
   data_set(data2_400_12) %>%
   Req(CP) %>%
   # zero_re() %>%
   mrgsim(end = 50, delta = 0.5) %>% filter(time==11.5) %>% summarise(prop_sup_11.285 = mean(CP>11.285)*100)
```

# Proportion of subjects with a residual concentration (Cmin) > moyenne range 5CI50 : Cmin > 31.978 mg/L

```
a5 <- my_model %>% 
   data_set(data2_400_12) %>%
   Req(CP) %>%
   # zero_re() %>%
   mrgsim(end = 50, delta = 0.5) %>% filter(time==11.5) %>% summarise(prop_sup_31.978 = mean(CP>31.978)*100)
```

```
## Warning in (function (x, data, idata = no_idata_set(), carry_out = carry.out, : [steady_bolus] ID 309 failed to reach steady state
##   ss_n: 500, ss_rtol: 1e-08, ss_atol: 1e-08
```

# Proportion of subjects with a residual concentration (Cmin) > haut range 5CI50 : Cmin > 52.67 mg/L

```
a6 <- my_model %>% 
   data_set(data2_400_12) %>%
   Req(CP) %>%
   # zero_re() %>%
   mrgsim(end = 50, delta = 0.5) %>% filter(time==11.5) %>% summarise(prop_sup_52.67 = mean(CP>52.67)*100)
```

##600/12h

```
set.seed(23456)
data_marib <- expand.ev(ID = 1:10000, amt = 600, ii=12, addl=1, ss=1)
data2_600_12 <- data_marib %>% mutate( WT =rtruncnorm(n(),a=50, b=120, mean=75, sd=10 ))
```

```
# Proportion of subjects with a residual concentration (Cmin) > CI50 : Cmin > 2.257 mg/L 
b1 <- my_model %>% 
   data_set(data2_600_12) %>%
   Req(CP) %>%
   # zero_re() %>%
   mrgsim(end = 50, delta = 0.5) %>% filter(time==11.5) %>% summarise(prop_sup_2.257 = mean(CP>2.257)*100)
```

# Proportion of subjects with a residual concentration (Cmin) > moyenne range CI50 : Cmin > 6.4 mg/L

```
b2 <- my_model %>% 
   data_set(data2_600_12) %>%
   Req(CP) %>%
   # zero_re() %>%
   mrgsim(end = 50, delta = 0.5) %>% filter(time==11.5) %>% summarise(prop_sup_6.4 = mean(CP>6.4)*100)
```

# Proportion of subjects with a residual concentration (Cmin) > haut range CI50 : Cmin > 10.534 mg/L

```
b3 <- my_model %>% 
   data_set(data2_600_12) %>%
   Req(CP) %>%
   # zero_re() %>%
   mrgsim(end = 50, delta = 0.5) %>% filter(time==11.5) %>% summarise(prop_sup_10.534 = mean(CP>10.534)*100)
```

# Proportion of subjects with a residual concentration (Cmin) > bas range 5CI50 : Cmin > 11.285 mg/L

```
b4 <- my_model %>% 
   data_set(data2_600_12) %>%
   Req(CP) %>%
   # zero_re() %>%
   mrgsim(end = 50, delta = 0.5) %>% filter(time==11.5) %>% summarise(prop_sup_11.285 = mean(CP>11.285)*100)
```

# Proportion of subjects with a residual concentration (Cmin) > moyenne range 5CI50 : Cmin > 31.978 mg/L

```
b5 <- my_model %>% 
   data_set(data2_600_12) %>%
   Req(CP) %>%
   # zero_re() %>%
   mrgsim(end = 50, delta = 0.5) %>% filter(time==11.5) %>% summarise(prop_sup_31.978 = mean(CP>31.978)*100)
```

```
## Warning in (function (x, data, idata = no_idata_set(), carry_out = carry.out, : [steady_bolus] ID 309 failed to reach steady state
##   ss_n: 500, ss_rtol: 1e-08, ss_atol: 1e-08
```

# Proportion of subjects with a residual concentration (Cmin) > haut range 5CI50 : Cmin > 52.67 mg/L

```
b6 <- my_model %>% 
   data_set(data2_600_12) %>%
   Req(CP) %>%
   # zero_re() %>%
   mrgsim(end = 50, delta = 0.5) %>% filter(time==11.5) %>% summarise(prop_sup_52.67 = mean(CP>52.67)*100)
```

##800/12h

```
set.seed(23456)
data_marib <- expand.ev(ID = 1:10000, amt = 800, ii=12, addl=1, ss=1)
data2_800_12 <- data_marib %>% mutate( WT =rtruncnorm(n(),a=50, b=120, mean=75, sd=10 ))
```

```
# Proportion of subjects with a residual concentration (Cmin) > CI50 : Cmin > 2.257 mg/L 
c1 <- my_model %>% 
   data_set(data2_800_12) %>%
   Req(CP) %>%
   # zero_re() %>%
   mrgsim(end = 50, delta = 0.5) %>% filter(time==11.5) %>% summarise(prop_sup_2.257 = mean(CP>2.257)*100)
```

# Proportion of subjects with a residual concentration (Cmin) > moyenne range CI50 : Cmin > 6.4 mg/L

```
c2 <- my_model %>% 
   data_set(data2_800_12) %>%
   Req(CP) %>%
   # zero_re() %>%
   mrgsim(end = 50, delta = 0.5) %>% filter(time==11.5) %>% summarise(prop_sup_6.4 = mean(CP>6.4)*100)
```

# Proportion of subjects with a residual concentration (Cmin) > haut range CI50 : Cmin > 10.534 mg/L

```
c3 <- my_model %>% 
   data_set(data2_800_12) %>%
   Req(CP) %>%
   # zero_re() %>%
   mrgsim(end = 50, delta = 0.5) %>% filter(time==11.5) %>% summarise(prop_sup_10.534 = mean(CP>10.534)*100)
```

# Proportion of subjects with a residual concentration (Cmin) > bas range 5CI50 : Cmin > 11.285 mg/L

```
c4 <- my_model %>% 
   data_set(data2_800_12) %>%
   Req(CP) %>%
   # zero_re() %>%
   mrgsim(end = 50, delta = 0.5) %>% filter(time==11.5) %>% summarise(prop_sup_11.285 = mean(CP>11.285)*100)
```

# Proportion of subjects with a residual concentration (Cmin) > moyenne range 5CI50 : Cmin > 31.978 mg/L

```
c5 <- my_model %>% 
   data_set(data2_800_12) %>%
   Req(CP) %>%
   # zero_re() %>%
   mrgsim(end = 50, delta = 0.5) %>% filter(time==11.5) %>% summarise(prop_sup_31.978 = mean(CP>31.978)*100)
```

```
## Warning in (function (x, data, idata = no_idata_set(), carry_out = carry.out, : [steady_bolus] ID 309 failed to reach steady state
##   ss_n: 500, ss_rtol: 1e-08, ss_atol: 1e-08
```

# Proportion of subjects with a residual concentration (Cmin) > haut range 5CI50 : Cmin > 52.67 mg/L

```
c6 <- my_model %>% 
   data_set(data2_800_12) %>%
   Req(CP) %>%
   # zero_re() %>%
   mrgsim(end = 50, delta = 0.5) %>% filter(time==11.5) %>% summarise(prop_sup_52.67 = mean(CP>52.67)*100)
```

##800/8h

```
set.seed(23456)
data_marib <- expand.ev(ID = 1:10000, amt = 800, ii=8, addl=1, ss=1)
data2_800_8 <- data_marib %>% mutate( WT =rtruncnorm(n(),a=50, b=120, mean=75, sd=10 ))
```

```
# Proportion of subjects with a residual concentration (Cmin) > CI50 : Cmin > 2.257 mg/L 
d1 <- my_model %>% 
   data_set(data2_800_8) %>%
   Req(CP) %>%
   # zero_re() %>%
   mrgsim(end = 50, delta = 0.5) %>% filter(time==7.5) %>% summarise(prop_sup_2.257 = mean(CP>2.257)*100)
```

# Proportion of subjects with a residual concentration (Cmin) > moyenne range CI50 : Cmin > 6.4 mg/L

```
d2 <- my_model %>% 
   data_set(data2_800_8) %>%
   Req(CP) %>%
   # zero_re() %>%
   mrgsim(end = 50, delta = 0.5) %>% filter(time==7.5) %>% summarise(prop_sup_6.4 = mean(CP>6.4)*100)
```

# Proportion of subjects with a residual concentration (Cmin) > haut range CI50 : Cmin > 10.534 mg/L

```
d3 <- my_model %>% 
   data_set(data2_800_8) %>%
   Req(CP) %>%
   # zero_re() %>%
   mrgsim(end = 50, delta = 0.5) %>% filter(time==7.5) %>% summarise(prop_sup_10.534 = mean(CP>10.534)*100)
```

# Proportion of subjects with a residual concentration (Cmin) > bas range 5CI50 : Cmin > 11.285 mg/L

```
d4 <- my_model %>% 
   data_set(data2_800_8) %>%
   Req(CP) %>%
   # zero_re() %>%
   mrgsim(end = 50, delta = 0.5) %>% filter(time==7.5) %>% summarise(prop_sup_11.285 = mean(CP>11.285)*100)
```

```
## Warning in (function (x, data, idata = no_idata_set(), carry_out = carry.out, : [steady_bolus] ID 2394 failed to reach steady state
##   ss_n: 500, ss_rtol: 1e-08, ss_atol: 1e-08
```

# Proportion of subjects with a residual concentration (Cmin) > moyenne range 5CI50 : Cmin > 31.978 mg/L

```
d5 <- my_model %>% 
   data_set(data2_800_8) %>%
   Req(CP) %>%
   # zero_re() %>%
   mrgsim(end = 50, delta = 0.5) %>% filter(time==7.5) %>% summarise(prop_sup_31.978 = mean(CP>31.978)*100)
```

```
## Warning in (function (x, data, idata = no_idata_set(), carry_out = carry.out, : [steady_bolus] ID 309 failed to reach steady state
##   ss_n: 500, ss_rtol: 1e-08, ss_atol: 1e-08
```

# Proportion of subjects with a residual concentration (Cmin) > haut range 5CI50 : Cmin > 52.67 mg/L

```
d6 <- my_model %>% 
   data_set(data2_800_8) %>%
   Req(CP) %>%
   # zero_re() %>%
   mrgsim(end = 50, delta = 0.5) %>% filter(time==7.5) %>% summarise(prop_sup_52.67 = mean(CP>52.67)*100)
```

##400/8h

```
set.seed(23456)
data_marib <- expand.ev(ID = 1:10000, amt = 400, ii=8, addl=1, ss=1)
data2_400_8 <- data_marib %>% mutate( WT =rtruncnorm(n(),a=50, b=120, mean=75, sd=10 ))
```

```
# Proportion of subjects with a residual concentration (Cmin) > CI50 : Cmin > 2.257 mg/L 
e1 <- my_model %>% 
   data_set(data2_400_8) %>%
   Req(CP) %>%
   # zero_re() %>%
   mrgsim(end = 50, delta = 0.5) %>% filter(time==7.5) %>% summarise(prop_sup_2.257 = mean(CP>2.257)*100)
```

# Proportion of subjects with a residual concentration (Cmin) > moyenne range CI50 : Cmin > 6.4 mg/L

```
e2 <- my_model %>% 
   data_set(data2_400_8) %>%
   Req(CP) %>%
   # zero_re() %>%
   mrgsim(end = 50, delta = 0.5) %>% filter(time==7.5) %>% summarise(prop_sup_6.4 = mean(CP>6.4)*100)
```

# Proportion of subjects with a residual concentration (Cmin) > haut range CI50 : Cmin > 10.534 mg/L

```
e3 <- my_model %>% 
   data_set(data2_400_8) %>%
   Req(CP) %>%
   # zero_re() %>%
   mrgsim(end = 50, delta = 0.5) %>% filter(time==7.5) %>% summarise(prop_sup_10.534 = mean(CP>10.534)*100)
```

# Proportion of subjects with a residual concentration (Cmin) > bas range 5CI50 : Cmin > 11.285 mg/L

```
e4 <- my_model %>% 
   data_set(data2_400_8) %>%
   Req(CP) %>%
   # zero_re() %>%
   mrgsim(end = 50, delta = 0.5) %>% filter(time==7.5) %>% summarise(prop_sup_11.285 = mean(CP>11.285)*100)
```

```
## Warning in (function (x, data, idata = no_idata_set(), carry_out = carry.out, : [steady_bolus] ID 2394 failed to reach steady state
##   ss_n: 500, ss_rtol: 1e-08, ss_atol: 1e-08
```

# Proportion of subjects with a residual concentration (Cmin) > moyenne range 5CI50 : Cmin > 31.978 mg/L

```
e5 <- my_model %>% 
   data_set(data2_400_8) %>%
   Req(CP) %>%
   # zero_re() %>%
   mrgsim(end = 50, delta = 0.5) %>% filter(time==7.5) %>% summarise(prop_sup_31.978 = mean(CP>31.978)*100)
```

```
## Warning in (function (x, data, idata = no_idata_set(), carry_out = carry.out, : [steady_bolus] ID 309 failed to reach steady state
##   ss_n: 500, ss_rtol: 1e-08, ss_atol: 1e-08
```

# Proportion of subjects with a residual concentration (Cmin) > haut range 5CI50 : Cmin > 52.67 mg/L

```
e6 <- my_model %>% 
   data_set(data2_400_8) %>%
   Req(CP) %>%
   # zero_re() %>%
   mrgsim(end = 50, delta = 0.5) %>% filter(time==7.5) %>% summarise(prop_sup_52.67 = mean(CP>52.67)*100)
```

```
# Combinaison des résultats
results <- data.frame(
  Scenario = c(rep("400 mg/12h", 6), rep("600 mg/12h", 6), rep("800 mg/12h", 6), rep("800 mg/8h", 6), rep("400 mg/8h", 6)),
  Concentration = rep(c(2.257, 6.4, 10.534, 11.285, 31.978, 52.67), 5),
  Proportion = c(
    a1$prop_sup_2.257, a2$prop_sup_6.4, a3$prop_sup_10.534, a4$prop_sup_11.285, a5$prop_sup_31.978, a6$prop_sup_52.67,
    b1$prop_sup_2.257, b2$prop_sup_6.4, b3$prop_sup_10.534, b4$prop_sup_11.285, b5$prop_sup_31.978, b6$prop_sup_52.67,
    c1$prop_sup_2.257, c2$prop_sup_6.4, c3$prop_sup_10.534, c4$prop_sup_11.285, c5$prop_sup_31.978, c6$prop_sup_52.67,
    d1$prop_sup_2.257, d2$prop_sup_6.4, d3$prop_sup_10.534, d4$prop_sup_11.285, d5$prop_sup_31.978, d6$prop_sup_52.67,
    e1$prop_sup_2.257, e2$prop_sup_6.4, e3$prop_sup_10.534, e4$prop_sup_11.285, e5$prop_sup_31.978, e6$prop_sup_52.67
  )
)

# Visualisation des résultats
p1 <- ggplot(results, aes(x = Concentration, y = Proportion, color = Scenario, group = Scenario)) +
  geom_point(size = 2) +
  geom_line() +
  geom_hline(yintercept = 90, color = "grey", linetype = "solid") +
  geom_vline(xintercept = 2.56, color = "blue", linetype = "dashed")  +
  geom_vline(xintercept = 10.53, color = "blue", linetype = "dashed")  +
  geom_vline(xintercept = 11.29, color = "red", linetype = "dashed")  +
  geom_vline(xintercept = 52.67, color = "red", linetype = "dashed")  +
  scale_y_continuous(limits = c(0, 100), breaks = seq(0, 100, 25)) +
 
  labs(
       x = "Inhibitory concentration targets (mg/L)",
       y = "Proportions with success (%)",
       color = "Dose regimen") +
  theme_minimal() +
  theme(legend.position = "right") +
scale_color_grey()

p1
```

```
# Filtrer les résultats pour les concentrations entre 2.257 et 10.534
results_filtered <- results %>% filter(Concentration >= 2.257 & Concentration <= 10.534)

# Visualisation des résultats avec zoom sur l'axe des abscisses
p2 <- ggplot(results_filtered, aes(x = Concentration, y = Proportion, color = Scenario, group = Scenario)) +
  geom_point(size = 2) +
  geom_line() +
  geom_hline(yintercept = 90, color = "grey", linetype = "solid") +
  geom_vline(xintercept = 2.257, color = "blue", linetype = "dashed")  +
  geom_vline(xintercept = 10.533, color = "blue", linetype = "dashed")  +
  scale_y_continuous(limits = c(0, 100), breaks = seq(0, 100, 25)) +
  scale_x_continuous(limits = c(2, 10.534), breaks = seq(0, 10.534, 2)) + 
  
  labs(
       x = "Inhibitory concentration targets (mg/L)",
       y = "Proportions with success (%)",
       color = "Dose regimen") +
  theme_minimal() +
  theme(legend.position = "right") + 
  scale_color_grey()

p2
```
