# Supplementary material for: Optimizing CMV therapy: Population pharmacokinetics and Monte Carlo simulations for letermovir and maribavir dosage
Source: PLoS One. 2025 Apr 28;20(4):e0321180. doi: 10.1371/journal.pone.0321180 (PMC12036903; doi:10.1371/journal.pone.0321180)
Supplement: S2 File — (HTML) [file pone.0321180.s006.html]

Letermovir


# Letermovir

#### Yeleen

#### 21\_09\_2023

```
knitr::opts_chunk$set(echo = TRUE)
options(tibble.print_max = Inf)
```

##Loading of the packages

```
library(mrgsolve)
```

```
## 
## Attaching package: 'mrgsolve'
```

```
## The following object is masked from 'package:stats':
## 
##     filter
```

```
library(tidyverse)
```

```
## Warning: package 'ggplot2' was built under R version 4.3.3
```

```
## ── Attaching core tidyverse packages ──────────────────────── tidyverse 2.0.0 ──
## ✔ dplyr     1.1.2     ✔ readr     2.1.4
## ✔ forcats   1.0.0     ✔ stringr   1.5.0
## ✔ ggplot2   3.5.0     ✔ tibble    3.2.1
## ✔ lubridate 1.9.2     ✔ tidyr     1.3.0
## ✔ purrr     1.0.2
```

```
## ── Conflicts ────────────────────────────────────────── tidyverse_conflicts() ──
## ✖ dplyr::filter() masks mrgsolve::filter(), stats::filter()
## ✖ dplyr::lag()    masks stats::lag()
## ℹ Use the conflicted package (<http://conflicted.r-lib.org/>) to force all conflicts to become errors
```

```
library(truncnorm)
library(ggplot2)
library(Pmetrics)
```

```
## 
## Attaching package: 'Pmetrics'
## 
## The following object is masked from 'package:stringr':
## 
##     fixed
```

##Definition of the model implemented from Prohn et al ; CPT
Pharmacometrics Syst. Pharmacol.; 2021 https://ascpt.onlinelibrary.wiley.com/doi/10.1002/psp4.12593

```
code <- "
[SET] end=100, delta=0.1

 
 [PARAM] @annotated
 TVCL1: 4.84 : Typical value of clearance without CSA (L/h) 
 TVCL2: 3.38 : Typical value of clearance with CSA (L/h)
 TVF1: 0.346 : bioavailability without CSA
 TVF2: 0.849 : bioavailability with CSA
 TVV1: 19.7 : Typical central volume of distribution (L) 
 TVQ: 1.54 : Typical intercomp clearance 1 (L/h)
 TVV2: 25.8 : Typical peripheral volume of distribution 2 (L)
 TVKA1: 0.150 : Typical transfert rate constant CMV+ (1/h)
 TVKA2: 1.26 : Typical transfert rate constant CMV - (1/h)
 TVALAG: 0.674 : Typical absorption lag (h)


 ETA1: 0 : clearance
 ETA2: 0 : V2
 ETA3: 0 : KA 
 ETA4: 0 : F 
 
[PARAM] @annotated @covariates
 CSA: 0: non csa (0) csa (1)
 CMV: 0: non CMV (0) CMV (1)
 
 [CMT] @annotated
 DEPOT : Dosing compartment (mg)[ADM]
 CENT : Central comaprtment (mg) [OBS]
 PERI : First peripheral compartment (mg)
 
   
 [OMEGA] 0.00366025 0.052441 0.516961 0.018769
 
 [SIGMA] @annotated
 ADD : 0.0001 : additive Residual unexplained variability 0.383
 PROP : 0.0001 : proportionnal Residual unexplained variability 0.517
 
 [MAIN]
double CL = TVCL2 * CSA +  TVCL1 * (1-CSA)  *exp(ETA1 + ETA(1));
double V1 = TVV1;
double Q = TVQ;
double V2 = TVV2 * exp(ETA2 + ETA(2));
double KA = TVKA1 * CMV +  TVKA2 * (1-CMV)   * exp(ETA3 + ETA(3));
ALAG_DEPOT = TVALAG;
F_DEPOT =  TVF2 * CSA +  TVF1 * (1-CSA)   * exp(ETA4 + ETA(4));
 

 
[ODE]
 dxdt_DEPOT = -KA*DEPOT;
 dxdt_CENT = KA * DEPOT - (CL + Q) * CENT / V1 + Q * PERI / V2; ;
 dxdt_PERI = Q*CENT/V1 - Q*PERI/V2 ;

[TABLE] 
capture DV = (CENT/V1) *(1 + EPS(1)) + EPS(2);
int i = 0;
while(DV <0 && i <100) {
simeps();
DV = (CENT/V1) *(1 + EPS(1)) + EPS(2);++i;
};
"
```

```
my_model <- mcode("leter_model",code)
```

```
## Building leter_model ... done.
```

##Without CICLOSPORINE

###10000 patients per os

```
set.seed(1234)
data_valid <- expand.ev(ID = 1:1000, amt = 480,ii=24, CMV =1, CSA= 0, addl = 2, ss=1)

histo <- my_model %>% 
  data_set(data_valid) %>%
  Req(DV) %>%
  mrgsim(end = 24, delta = 0.5)
```

Histogram of all the concentrations simulated at 24h and mean C24h to
compare with the literature

```
histo  %>%
filter(time==23.5) %>% ggplot(aes(x = DV)) + geom_histogram() + labs(x = "distribution of CP at 24h") + theme_bw()
```

```
## `stat_bin()` using `bins = 30`. Pick better value with `binwidth`.
```

#Graph

```
e1 <- ev(ID = 1:1000, amt = 480,ii=24, CMV =1, CSA= 0, addl = 0, ss=0)
e2 <- ev(ID = 1:1000, amt = 480,ii=24, CMV =1, CSA= 0, addl = 5, ss=0)
e3 <- ev(ID = 1:1000, amt = 480,ii=24, CMV =1, CSA= 0, addl = 2, ss=1)
data_ev <- seq(e1, e2,e3)
data_leter <- as_tibble(data_ev) %>% arrange(ID) 


set.seed(1234)

sim_leter <- my_model %>% 
  data_set(data_leter) %>%
  Req(DV) %>%
  mrgsim(delta = 1, end = 280)

as_tibble(sim_leter) %>% filter(between(time, 0,280)) %>%
    ggplot(aes(x = time, y = DV, color = as.factor(ID))) + 
  geom_line(show.legend = FALSE) + 
  geom_point(show.legend = FALSE) +
  labs(y = "Letermovir (mg/L)", x = "Time (h)") + 
  theme_bw()
```

#Without loading dose

```
e1 <- ev(ID = 1:10000, amt = 480,ii=24, CMV =1, CSA= 0, addl = 0, ss=0)
e2 <- ev(ID = 1:10000, amt = 480,ii=24, CMV =1, CSA= 0, addl = 5, ss=0)
e3 <- ev(ID = 1:10000, amt = 480,ii=24, CMV =1, CSA= 0, addl = 2, ss=1)
data_ev <- seq(e1, e2,e3)
data_leter <- as_tibble(data_ev) %>% arrange(ID) 

set.seed(1234)
```

```
set.seed(1234)
temps_d_interet <- c(23, 47, 71, 95, 119, 143, 167, 191, 215, 239)
resultats <- lapply(temps_d_interet, function(temps) {
  my_model %>% 
    data_set(data_leter) %>%
    Req(DV) %>%
    mrgsim(delta = 0.1, end = 250) %>%
    filter(time == temps) %>%
    filter(!is.na(DV)) %>% 
    summarise(
      mean = mean(DV), sd(DV),
      gm_CMin = exp(mean(log(DV))),
      median = fivenum(DV),
      n = n()
    ) %>%
    mutate(temps = temps)
})
```

```
## Warning: Returning more (or less) than 1 row per `summarise()` group was deprecated in
## dplyr 1.1.0.
## ℹ Please use `reframe()` instead.
## ℹ When switching from `summarise()` to `reframe()`, remember that `reframe()`
##   always returns an ungrouped data frame and adjust accordingly.
## Call `lifecycle::last_lifecycle_warnings()` to see where this warning was
## generated.
```

```
## Warning: Returning more (or less) than 1 row per `summarise()` group was deprecated in
## dplyr 1.1.0.
## ℹ Please use `reframe()` instead.
## ℹ When switching from `summarise()` to `reframe()`, remember that `reframe()`
##   always returns an ungrouped data frame and adjust accordingly.
## Call `lifecycle::last_lifecycle_warnings()` to see where this warning was
## generated.
```

```
## Warning: Returning more (or less) than 1 row per `summarise()` group was deprecated in
## dplyr 1.1.0.
## ℹ Please use `reframe()` instead.
## ℹ When switching from `summarise()` to `reframe()`, remember that `reframe()`
##   always returns an ungrouped data frame and adjust accordingly.
## Call `lifecycle::last_lifecycle_warnings()` to see where this warning was
## generated.
```

```
## Warning: Returning more (or less) than 1 row per `summarise()` group was deprecated in
## dplyr 1.1.0.
## ℹ Please use `reframe()` instead.
## ℹ When switching from `summarise()` to `reframe()`, remember that `reframe()`
##   always returns an ungrouped data frame and adjust accordingly.
## Call `lifecycle::last_lifecycle_warnings()` to see where this warning was
## generated.
```

```
## Warning: Returning more (or less) than 1 row per `summarise()` group was deprecated in
## dplyr 1.1.0.
## ℹ Please use `reframe()` instead.
## ℹ When switching from `summarise()` to `reframe()`, remember that `reframe()`
##   always returns an ungrouped data frame and adjust accordingly.
## Call `lifecycle::last_lifecycle_warnings()` to see where this warning was
## generated.
```

```
## Warning: Returning more (or less) than 1 row per `summarise()` group was deprecated in
## dplyr 1.1.0.
## ℹ Please use `reframe()` instead.
## ℹ When switching from `summarise()` to `reframe()`, remember that `reframe()`
##   always returns an ungrouped data frame and adjust accordingly.
## Call `lifecycle::last_lifecycle_warnings()` to see where this warning was
## generated.
```

```
## Warning: Returning more (or less) than 1 row per `summarise()` group was deprecated in
## dplyr 1.1.0.
## ℹ Please use `reframe()` instead.
## ℹ When switching from `summarise()` to `reframe()`, remember that `reframe()`
##   always returns an ungrouped data frame and adjust accordingly.
## Call `lifecycle::last_lifecycle_warnings()` to see where this warning was
## generated.
```

```
## Warning: Returning more (or less) than 1 row per `summarise()` group was deprecated in
## dplyr 1.1.0.
## ℹ Please use `reframe()` instead.
## ℹ When switching from `summarise()` to `reframe()`, remember that `reframe()`
##   always returns an ungrouped data frame and adjust accordingly.
## Call `lifecycle::last_lifecycle_warnings()` to see where this warning was
## generated.
```

```
## Warning: Returning more (or less) than 1 row per `summarise()` group was deprecated in
## dplyr 1.1.0.
## ℹ Please use `reframe()` instead.
## ℹ When switching from `summarise()` to `reframe()`, remember that `reframe()`
##   always returns an ungrouped data frame and adjust accordingly.
## Call `lifecycle::last_lifecycle_warnings()` to see where this warning was
## generated.
```

```
## Warning: Returning more (or less) than 1 row per `summarise()` group was deprecated in
## dplyr 1.1.0.
## ℹ Please use `reframe()` instead.
## ℹ When switching from `summarise()` to `reframe()`, remember that `reframe()`
##   always returns an ungrouped data frame and adjust accordingly.
## Call `lifecycle::last_lifecycle_warnings()` to see where this warning was
## generated.
```

```
resultats_tibble <- bind_rows(resultats)
print(resultats_tibble)
```

```
## # A tibble: 50 × 6
##     mean `sd(DV)` gm_CMin median     n temps
##    <dbl>    <dbl>   <dbl>  <dbl> <int> <dbl>
##  1 0.453   0.0791   0.446  0.239 10000    23
##  2 0.453   0.0791   0.446  0.396 10000    23
##  3 0.453   0.0791   0.446  0.447 10000    23
##  4 0.453   0.0791   0.446  0.503 10000    23
##  5 0.453   0.0791   0.446  0.820 10000    23
##  6 0.547   0.0937   0.539  0.284 10000    47
##  7 0.547   0.0937   0.539  0.479 10000    47
##  8 0.547   0.0937   0.539  0.539 10000    47
##  9 0.547   0.0937   0.539  0.604 10000    47
## 10 0.547   0.0937   0.539  0.998 10000    47
## 11 0.580   0.0982   0.571  0.281 10000    71
## 12 0.580   0.0982   0.571  0.512 10000    71
## 13 0.580   0.0982   0.571  0.572 10000    71
## 14 0.580   0.0982   0.571  0.641 10000    71
## 15 0.580   0.0982   0.571  1.10  10000    71
## 16 0.591   0.101    0.582  0.302 10000    95
## 17 0.591   0.101    0.582  0.520 10000    95
## 18 0.591   0.101    0.582  0.582 10000    95
## 19 0.591   0.101    0.582  0.655 10000    95
## 20 0.591   0.101    0.582  1.07  10000    95
## 21 0.596   0.101    0.587  0.296 10000   119
## 22 0.596   0.101    0.587  0.526 10000   119
## 23 0.596   0.101    0.587  0.588 10000   119
## 24 0.596   0.101    0.587  0.659 10000   119
## 25 0.596   0.101    0.587  1.09  10000   119
## 26 0.597   0.101    0.589  0.317 10000   143
## 27 0.597   0.101    0.589  0.526 10000   143
## 28 0.597   0.101    0.589  0.590 10000   143
## 29 0.597   0.101    0.589  0.661 10000   143
## 30 0.597   0.101    0.589  1.15  10000   143
## 31 0.598   0.102    0.590  0.307 10000   167
## 32 0.598   0.102    0.590  0.527 10000   167
## 33 0.598   0.102    0.590  0.589 10000   167
## 34 0.598   0.102    0.590  0.661 10000   167
## 35 0.598   0.102    0.590  1.13  10000   167
## 36 0.598   0.102    0.590  0.290 10000   191
## 37 0.598   0.102    0.590  0.526 10000   191
## 38 0.598   0.102    0.590  0.590 10000   191
## 39 0.598   0.102    0.590  0.662 10000   191
## 40 0.598   0.102    0.590  1.23  10000   191
## 41 0.599   0.103    0.590  0.314 10000   215
## 42 0.599   0.103    0.590  0.527 10000   215
## 43 0.599   0.103    0.590  0.590 10000   215
## 44 0.599   0.103    0.590  0.661 10000   215
## 45 0.599   0.103    0.590  1.37  10000   215
## 46 0.600   0.102    0.592  0.313 10000   239
## 47 0.600   0.102    0.592  0.529 10000   239
## 48 0.600   0.102    0.592  0.592 10000   239
## 49 0.600   0.102    0.592  0.662 10000   239
## 50 0.600   0.102    0.592  1.10  10000   239
```

##Missing dose

#Graph

```
data<- ev(ID = 1:10, amt = 480,ii=24, CMV =1, CSA= 0, addl = 4, ss=1)
sim_leter <- my_model %>% 
  data_set(data) %>%
  Req(DV) %>%
  mrgsim(delta = 0.1, end = 280)

as_tibble(sim_leter) %>% filter(between(time, 0,280)) %>%
    ggplot(aes(x = time, y = DV, color = as.factor(ID))) + 
  geom_line(show.legend = FALSE) + 
  geom_point(show.legend = FALSE) +
  labs(y = "Letermovir (mg/L)", x = "Time (h)") + 
  theme_bw()
```

```
set.seed(1234)

ev(ID = 1:10000, amt = 480,ii=24, CMV =1, CSA= 0, ss=1)
```

```
## Events:
##          ID time amt ii cmt evid ss CMV CSA
## 1         1    0 480 24   1    1  1   1   0
## 2         2    0 480 24   1    1  1   1   0
## 3         3    0 480 24   1    1  1   1   0
## 4         4    0 480 24   1    1  1   1   0
## 5         5    0 480 24   1    1  1   1   0
## 6         6    0 480 24   1    1  1   1   0
## 7         7    0 480 24   1    1  1   1   0
## 8         8    0 480 24   1    1  1   1   0
## 9         9    0 480 24   1    1  1   1   0
## 10       10    0 480 24   1    1  1   1   0
## 11       11    0 480 24   1    1  1   1   0
## 12       12    0 480 24   1    1  1   1   0
## 13       13    0 480 24   1    1  1   1   0
## 14       14    0 480 24   1    1  1   1   0
## 15       15    0 480 24   1    1  1   1   0
## 16       16    0 480 24   1    1  1   1   0
## 17       17    0 480 24   1    1  1   1   0
## 18       18    0 480 24   1    1  1   1   0
## 19       19    0 480 24   1    1  1   1   0
## 20       20    0 480 24   1    1  1   1   0
## 21       21    0 480 24   1    1  1   1   0
## 22       22    0 480 24   1    1  1   1   0
## 23       23    0 480 24   1    1  1   1   0
## 24       24    0 480 24   1    1  1   1   0
## 25       25    0 480 24   1    1  1   1   0
## 26       26    0 480 24   1    1  1   1   0
## 27       27    0 480 24   1    1  1   1   0
## 28       28    0 480 24   1    1  1   1   0
## 29       29    0 480 24   1    1  1   1   0
## 30       30    0 480 24   1    1  1   1   0
## 31       31    0 480 24   1    1  1   1   0
## 32       32    0 480 24   1    1  1   1   0
## 33       33    0 480 24   1    1  1   1   0
## 34       34    0 480 24   1    1  1   1   0
## 35       35    0 480 24   1    1  1   1   0
## 36       36    0 480 24   1    1  1   1   0
## 37       37    0 480 24   1    1  1   1   0
## 38       38    0 480 24   1    1  1   1   0
## 39       39    0 480 24   1    1  1   1   0
## 40       40    0 480 24   1    1  1   1   0
## 41       41    0 480 24   1    1  1   1   0
## 42       42    0 480 24   1    1  1   1   0
## 43       43    0 480 24   1    1  1   1   0
## 44       44    0 480 24   1    1  1   1   0
## 45       45    0 480 24   1    1  1   1   0
## 46       46    0 480 24   1    1  1   1   0
## 47       47    0 480 24   1    1  1   1   0
## 48       48    0 480 24   1    1  1   1   0
## 49       49    0 480 24   1    1  1   1   0
## 50       50    0 480 24   1    1  1   1   0
## 51       51    0 480 24   1    1  1   1   0
## 52       52    0 480 24   1    1  1   1   0
## 53       53    0 480 24   1    1  1   1   0
## 54       54    0 480 24   1    1  1   1   0
## 55       55    0 480 24   1    1  1   1   0
## 56       56    0 480 24   1    1  1   1   0
## 57       57    0 480 24   1    1  1   1   0
## 58       58    0 480 24   1    1  1   1   0
## 59       59    0 480 24   1    1  1   1   0
## 60       60    0 480 24   1    1  1   1   0
## 61       61    0 480 24   1    1  1   1   0
## 62       62    0 480 24   1    1  1   1   0
## 63       63    0 480 24   1    1  1   1   0
## 64       64    0 480 24   1    1  1   1   0
## 65       65    0 480 24   1    1  1   1   0
## 66       66    0 480 24   1    1  1   1   0
## 67       67    0 480 24   1    1  1   1   0
## 68       68    0 480 24   1    1  1   1   0
## 69       69    0 480 24   1    1  1   1   0
## 70       70    0 480 24   1    1  1   1   0
## 71       71    0 480 24   1    1  1   1   0
## 72       72    0 480 24   1    1  1   1   0
## 73       73    0 480 24   1    1  1   1   0
## 74       74    0 480 24   1    1  1   1   0
## 75       75    0 480 24   1    1  1   1   0
## 76       76    0 480 24   1    1  1   1   0
## 77       77    0 480 24   1    1  1   1   0
## 78       78    0 480 24   1    1  1   1   0
## 79       79    0 480 24   1    1  1   1   0
## 80       80    0 480 24   1    1  1   1   0
## 81       81    0 480 24   1    1  1   1   0
## 82       82    0 480 24   1    1  1   1   0
## 83       83    0 480 24   1    1  1   1   0
## 84       84    0 480 24   1    1  1   1   0
## 85       85    0 480 24   1    1  1   1   0
## 86       86    0 480 24   1    1  1   1   0
## 87       87    0 480 24   1    1  1   1   0
## 88       88    0 480 24   1    1  1   1   0
## 89       89    0 480 24   1    1  1   1   0
## 90       90    0 480 24   1    1  1   1   0
## 91       91    0 480 24   1    1  1   1   0
## 92       92    0 480 24   1    1  1   1   0
## 93       93    0 480 24   1    1  1   1   0
## 94       94    0 480 24   1    1  1   1   0
## 95       95    0 480 24   1    1  1   1   0
## 96       96    0 480 24   1    1  1   1   0
## 97       97    0 480 24   1    1  1   1   0
## 98       98    0 480 24   1    1  1   1   0
## 99       99    0 480 24   1    1  1   1   0
## 100     100    0 480 24   1    1  1   1   0
## 101     101    0 480 24   1    1  1   1   0
## 102     102    0 480 24   1    1  1   1   0
## 103     103    0 480 24   1    1  1   1   0
## 104     104    0 480 24   1    1  1   1   0
## 105     105    0 480 24   1    1  1   1   0
## 106     106    0 480 24   1    1  1   1   0
## 107     107    0 480 24   1    1  1   1   0
## 108     108    0 480 24   1    1  1   1   0
## 109     109    0 480 24   1    1  1   1   0
## 110     110    0 480 24   1    1  1   1   0
## 111     111    0 480 24   1    1  1   1   0
## 112     112    0 480 24   1    1  1   1   0
## 113     113    0 480 24   1    1  1   1   0
## 114     114    0 480 24   1    1  1   1   0
## 115     115    0 480 24   1    1  1   1   0
## 116     116    0 480 24   1    1  1   1   0
## 117     117    0 480 24   1    1  1   1   0
## 118     118    0 480 24   1    1  1   1   0
## 119     119    0 480 24   1    1  1   1   0
## 120     120    0 480 24   1    1  1   1   0
## 121     121    0 480 24   1    1  1   1   0
## 122     122    0 480 24   1    1  1   1   0
## 123     123    0 480 24   1    1  1   1   0
## 124     124    0 480 24   1    1  1   1   0
## 125     125    0 480 24   1    1  1   1   0
## 126     126    0 480 24   1    1  1   1   0
## 127     127    0 480 24   1    1  1   1   0
## 128     128    0 480 24   1    1  1   1   0
## 129     129    0 480 24   1    1  1   1   0
## 130     130    0 480 24   1    1  1   1   0
## 131     131    0 480 24   1    1  1   1   0
## 132     132    0 480 24   1    1  1   1   0
## 133     133    0 480 24   1    1  1   1   0
## 134     134    0 480 24   1    1  1   1   0
## 135     135    0 480 24   1    1  1   1   0
## 136     136    0 480 24   1    1  1   1   0
## 137     137    0 480 24   1    1  1   1   0
## 138     138    0 480 24   1    1  1   1   0
## 139     139    0 480 24   1    1  1   1   0
## 140     140    0 480 24   1    1  1   1   0
## 141     141    0 480 24   1    1  1   1   0
## 142     142    0 480 24   1    1  1   1   0
## 143     143    0 480 24   1    1  1   1   0
## 144     144    0 480 24   1    1  1   1   0
## 145     145    0 480 24   1    1  1   1   0
## 146     146    0 480 24   1    1  1   1   0
## 147     147    0 480 24   1    1  1   1   0
## 148     148    0 480 24   1    1  1   1   0
## 149     149    0 480 24   1    1  1   1   0
## 150     150    0 480 24   1    1  1   1   0
## 151     151    0 480 24   1    1  1   1   0
## 152     152    0 480 24   1    1  1   1   0
## 153     153    0 480 24   1    1  1   1   0
## 154     154    0 480 24   1    1  1   1   0
## 155     155    0 480 24   1    1  1   1   0
## 156     156    0 480 24   1    1  1   1   0
## 157     157    0 480 24   1    1  1   1   0
## 158     158    0 480 24   1    1  1   1   0
## 159     159    0 480 24   1    1  1   1   0
## 160     160    0 480 24   1    1  1   1   0
## 161     161    0 480 24   1    1  1   1   0
## 162     162    0 480 24   1    1  1   1   0
## 163     163    0 480 24   1    1  1   1   0
## 164     164    0 480 24   1    1  1   1   0
## 165     165    0 480 24   1    1  1   1   0
## 166     166    0 480 24   1    1  1   1   0
## 167     167    0 480 24   1    1  1   1   0
## 168     168    0 480 24   1    1  1   1   0
## 169     169    0 480 24   1    1  1   1   0
## 170     170    0 480 24   1    1  1   1   0
## 171     171    0 480 24   1    1  1   1   0
## 172     172    0 480 24   1    1  1   1   0
## 173     173    0 480 24   1    1  1   1   0
## 174     174    0 480 24   1    1  1   1   0
## 175     175    0 480 24   1    1  1   1   0
## 176     176    0 480 24   1    1  1   1   0
## 177     177    0 480 24   1    1  1   1   0
## 178     178    0 480 24   1    1  1   1   0
## 179     179    0 480 24   1    1  1   1   0
## 180     180    0 480 24   1    1  1   1   0
## 181     181    0 480 24   1    1  1   1   0
## 182     182    0 480 24   1    1  1   1   0
## 183     183    0 480 24   1    1  1   1   0
## 184     184    0 480 24   1    1  1   1   0
## 185     185    0 480 24   1    1  1   1   0
## 186     186    0 480 24   1    1  1   1   0
## 187     187    0 480 24   1    1  1   1   0
## 188     188    0 480 24   1    1  1   1   0
## 189     189    0 480 24   1    1  1   1   0
## 190     190    0 480 24   1    1  1   1   0
## 191     191    0 480 24   1    1  1   1   0
## 192     192    0 480 24   1    1  1   1   0
## 193     193    0 480 24   1    1  1   1   0
## 194     194    0 480 24   1    1  1   1   0
## 195     195    0 480 24   1    1  1   1   0
## 196     196    0 480 24   1    1  1   1   0
## 197     197    0 480 24   1    1  1   1   0
## 198     198    0 480 24   1    1  1   1   0
## 199     199    0 480 24   1    1  1   1   0
## 200     200    0 480 24   1    1  1   1   0
## 201     201    0 480 24   1    1  1   1   0
## 202     202    0 480 24   1    1  1   1   0
## 203     203    0 480 24   1    1  1   1   0
## 204     204    0 480 24   1    1  1   1   0
## 205     205    0 480 24   1    1  1   1   0
## 206     206    0 480 24   1    1  1   1   0
## 207     207    0 480 24   1    1  1   1   0
## 208     208    0 480 24   1    1  1   1   0
## 209     209    0 480 24   1    1  1   1   0
## 210     210    0 480 24   1    1  1   1   0
## 211     211    0 480 24   1    1  1   1   0
## 212     212    0 480 24   1    1  1   1   0
## 213     213    0 480 24   1    1  1   1   0
## 214     214    0 480 24   1    1  1   1   0
## 215     215    0 480 24   1    1  1   1   0
## 216     216    0 480 24   1    1  1   1   0
## 217     217    0 480 24   1    1  1   1   0
## 218     218    0 480 24   1    1  1   1   0
## 219     219    0 480 24   1    1  1   1   0
## 220     220    0 480 24   1    1  1   1   0
## 221     221    0 480 24   1    1  1   1   0
## 222     222    0 480 24   1    1  1   1   0
## 223     223    0 480 24   1    1  1   1   0
## 224     224    0 480 24   1    1  1   1   0
## 225     225    0 480 24   1    1  1   1   0
## 226     226    0 480 24   1    1  1   1   0
## 227     227    0 480 24   1    1  1   1   0
## 228     228    0 480 24   1    1  1   1   0
## 229     229    0 480 24   1    1  1   1   0
## 230     230    0 480 24   1    1  1   1   0
## 231     231    0 480 24   1    1  1   1   0
## 232     232    0 480 24   1    1  1   1   0
## 233     233    0 480 24   1    1  1   1   0
## 234     234    0 480 24   1    1  1   1   0
## 235     235    0 480 24   1    1  1   1   0
## 236     236    0 480 24   1    1  1   1   0
## 237     237    0 480 24   1    1  1   1   0
## 238     238    0 480 24   1    1  1   1   0
## 239     239    0 480 24   1    1  1   1   0
## 240     240    0 480 24   1    1  1   1   0
## 241     241    0 480 24   1    1  1   1   0
## 242     242    0 480 24   1    1  1   1   0
## 243     243    0 480 24   1    1  1   1   0
## 244     244    0 480 24   1    1  1   1   0
## 245     245    0 480 24   1    1  1   1   0
## 246     246    0 480 24   1    1  1   1   0
## 247     247    0 480 24   1    1  1   1   0
## 248     248    0 480 24   1    1  1   1   0
## 249     249    0 480 24   1    1  1   1   0
## 250     250    0 480 24   1    1  1   1   0
## 251     251    0 480 24   1    1  1   1   0
## 252     252    0 480 24   1    1  1   1   0
## 253     253    0 480 24   1    1  1   1   0
## 254     254    0 480 24   1    1  1   1   0
## 255     255    0 480 24   1    1  1   1   0
## 256     256    0 480 24   1    1  1   1   0
## 257     257    0 480 24   1    1  1   1   0
## 258     258    0 480 24   1    1  1   1   0
## 259     259    0 480 24   1    1  1   1   0
## 260     260    0 480 24   1    1  1   1   0
## 261     261    0 480 24   1    1  1   1   0
## 262     262    0 480 24   1    1  1   1   0
## 263     263    0 480 24   1    1  1   1   0
## 264     264    0 480 24   1    1  1   1   0
## 265     265    0 480 24   1    1  1   1   0
## 266     266    0 480 24   1    1  1   1   0
## 267     267    0 480 24   1    1  1   1   0
## 268     268    0 480 24   1    1  1   1   0
## 269     269    0 480 24   1    1  1   1   0
## 270     270    0 480 24   1    1  1   1   0
## 271     271    0 480 24   1    1  1   1   0
## 272     272    0 480 24   1    1  1   1   0
## 273     273    0 480 24   1    1  1   1   0
## 274     274    0 480 24   1    1  1   1   0
## 275     275    0 480 24   1    1  1   1   0
## 276     276    0 480 24   1    1  1   1   0
## 277     277    0 480 24   1    1  1   1   0
## 278     278    0 480 24   1    1  1   1   0
## 279     279    0 480 24   1    1  1   1   0
## 280     280    0 480 24   1    1  1   1   0
## 281     281    0 480 24   1    1  1   1   0
## 282     282    0 480 24   1    1  1   1   0
## 283     283    0 480 24   1    1  1   1   0
## 284     284    0 480 24   1    1  1   1   0
## 285     285    0 480 24   1    1  1   1   0
## 286     286    0 480 24   1    1  1   1   0
## 287     287    0 480 24   1    1  1   1   0
## 288     288    0 480 24   1    1  1   1   0
## 289     289    0 480 24   1    1  1   1   0
## 290     290    0 480 24   1    1  1   1   0
## 291     291    0 480 24   1    1  1   1   0
## 292     292    0 480 24   1    1  1   1   0
## 293     293    0 480 24   1    1  1   1   0
## 294     294    0 480 24   1    1  1   1   0
## 295     295    0 480 24   1    1  1   1   0
## 296     296    0 480 24   1    1  1   1   0
## 297     297    0 480 24   1    1  1   1   0
## 298     298    0 480 24   1    1  1   1   0
## 299     299    0 480 24   1    1  1   1   0
## 300     300    0 480 24   1    1  1   1   0
## 301     301    0 480 24   1    1  1   1   0
## 302     302    0 480 24   1    1  1   1   0
## 303     303    0 480 24   1    1  1   1   0
## 304     304    0 480 24   1    1  1   1   0
## 305     305    0 480 24   1    1  1   1   0
## 306     306    0 480 24   1    1  1   1   0
## 307     307    0 480 24   1    1  1   1   0
## 308     308    0 480 24   1    1  1   1   0
## 309     309    0 480 24   1    1  1   1   0
## 310     310    0 480 24   1    1  1   1   0
## 311     311    0 480 24   1    1  1   1   0
## 312     312    0 480 24   1    1  1   1   0
## 313     313    0 480 24   1    1  1   1   0
## 314     314    0 480 24   1    1  1   1   0
## 315     315    0 480 24   1    1  1   1   0
## 316     316    0 480 24   1    1  1   1   0
## 317     317    0 480 24   1    1  1   1   0
## 318     318    0 480 24   1    1  1   1   0
## 319     319    0 480 24   1    1  1   1   0
## 320     320    0 480 24   1    1  1   1   0
## 321     321    0 480 24   1    1  1   1   0
## 322     322    0 480 24   1    1  1   1   0
## 323     323    0 480 24   1    1  1   1   0
## 324     324    0 480 24   1    1  1   1   0
## 325     325    0 480 24   1    1  1   1   0
## 326     326    0 480 24   1    1  1   1   0
## 327     327    0 480 24   1    1  1   1   0
## 328     328    0 480 24   1    1  1   1   0
## 329     329    0 480 24   1    1  1   1   0
## 330     330    0 480 24   1    1  1   1   0
## 331     331    0 480 24   1    1  1   1   0
## 332     332    0 480 24   1    1  1   1   0
## 333     333    0 480 24   1    1  1   1   0
## 334     334    0 480 24   1    1  1   1   0
## 335     335    0 480 24   1    1  1   1   0
## 336     336    0 480 24   1    1  1   1   0
## 337     337    0 480 24   1    1  1   1   0
## 338     338    0 480 24   1    1  1   1   0
## 339     339    0 480 24   1    1  1   1   0
## 340     340    0 480 24   1    1  1   1   0
## 341     341    0 480 24   1    1  1   1   0
## 342     342    0 480 24   1    1  1   1   0
## 343     343    0 480 24   1    1  1   1   0
## 344     344    0 480 24   1    1  1   1   0
## 345     345    0 480 24   1    1  1   1   0
## 346     346    0 480 24   1    1  1   1   0
## 347     347    0 480 24   1    1  1   1   0
## 348     348    0 480 24   1    1  1   1   0
## 349     349    0 480 24   1    1  1   1   0
## 350     350    0 480 24   1    1  1   1   0
## 351     351    0 480 24   1    1  1   1   0
## 352     352    0 480 24   1    1  1   1   0
## 353     353    0 480 24   1    1  1   1   0
## 354     354    0 480 24   1    1  1   1   0
## 355     355    0 480 24   1    1  1   1   0
## 356     356    0 480 24   1    1  1   1   0
## 357     357    0 480 24   1    1  1   1   0
## 358     358    0 480 24   1    1  1   1   0
## 359     359    0 480 24   1    1  1   1   0
## 360     360    0 480 24   1    1  1   1   0
## 361     361    0 480 24   1    1  1   1   0
## 362     362    0 480 24   1    1  1   1   0
## 363     363    0 480 24   1    1  1   1   0
## 364     364    0 480 24   1    1  1   1   0
## 365     365    0 480 24   1    1  1   1   0
## 366     366    0 480 24   1    1  1   1   0
## 367     367    0 480 24   1    1  1   1   0
## 368     368    0 480 24   1    1  1   1   0
## 369     369    0 480 24   1    1  1   1   0
## 370     370    0 480 24   1    1  1   1   0
## 371     371    0 480 24   1    1  1   1   0
## 372     372    0 480 24   1    1  1   1   0
## 373     373    0 480 24   1    1  1   1   0
## 374     374    0 480 24   1    1  1   1   0
## 375     375    0 480 24   1    1  1   1   0
## 376     376    0 480 24   1    1  1   1   0
## 377     377    0 480 24   1    1  1   1   0
## 378     378    0 480 24   1    1  1   1   0
## 379     379    0 480 24   1    1  1   1   0
## 380     380    0 480 24   1    1  1   1   0
## 381     381    0 480 24   1    1  1   1   0
## 382     382    0 480 24   1    1  1   1   0
## 383     383    0 480 24   1    1  1   1   0
## 384     384    0 480 24   1    1  1   1   0
## 385     385    0 480 24   1    1  1   1   0
## 386     386    0 480 24   1    1  1   1   0
## 387     387    0 480 24   1    1  1   1   0
## 388     388    0 480 24   1    1  1   1   0
## 389     389    0 480 24   1    1  1   1   0
## 390     390    0 480 24   1    1  1   1   0
## 391     391    0 480 24   1    1  1   1   0
## 392     392    0 480 24   1    1  1   1   0
## 393     393    0 480 24   1    1  1   1   0
## 394     394    0 480 24   1    1  1   1   0
## 395     395    0 480 24   1    1  1   1   0
## 396     396    0 480 24   1    1  1   1   0
## 397     397    0 480 24   1    1  1   1   0
## 398     398    0 480 24   1    1  1   1   0
## 399     399    0 480 24   1    1  1   1   0
## 400     400    0 480 24   1    1  1   1   0
## 401     401    0 480 24   1    1  1   1   0
## 402     402    0 480 24   1    1  1   1   0
## 403     403    0 480 24   1    1  1   1   0
## 404     404    0 480 24   1    1  1   1   0
## 405     405    0 480 24   1    1  1   1   0
## 406     406    0 480 24   1    1  1   1   0
## 407     407    0 480 24   1    1  1   1   0
## 408     408    0 480 24   1    1  1   1   0
## 409     409    0 480 24   1    1  1   1   0
## 410     410    0 480 24   1    1  1   1   0
## 411     411    0 480 24   1    1  1   1   0
## 412     412    0 480 24   1    1  1   1   0
## 413     413    0 480 24   1    1  1   1   0
## 414     414    0 480 24   1    1  1   1   0
## 415     415    0 480 24   1    1  1   1   0
## 416     416    0 480 24   1    1  1   1   0
## 417     417    0 480 24   1    1  1   1   0
## 418     418    0 480 24   1    1  1   1   0
## 419     419    0 480 24   1    1  1   1   0
## 420     420    0 480 24   1    1  1   1   0
## 421     421    0 480 24   1    1  1   1   0
## 422     422    0 480 24   1    1  1   1   0
## 423     423    0 480 24   1    1  1   1   0
## 424     424    0 480 24   1    1  1   1   0
## 425     425    0 480 24   1    1  1   1   0
## 426     426    0 480 24   1    1  1   1   0
## 427     427    0 480 24   1    1  1   1   0
## 428     428    0 480 24   1    1  1   1   0
## 429     429    0 480 24   1    1  1   1   0
## 430     430    0 480 24   1    1  1   1   0
## 431     431    0 480 24   1    1  1   1   0
## 432     432    0 480 24   1    1  1   1   0
## 433     433    0 480 24   1    1  1   1   0
## 434     434    0 480 24   1    1  1   1   0
## 435     435    0 480 24   1    1  1   1   0
## 436     436    0 480 24   1    1  1   1   0
## 437     437    0 480 24   1    1  1   1   0
## 438     438    0 480 24   1    1  1   1   0
## 439     439    0 480 24   1    1  1   1   0
## 440     440    0 480 24   1    1  1   1   0
## 441     441    0 480 24   1    1  1   1   0
## 442     442    0 480 24   1    1  1   1   0
## 443     443    0 480 24   1    1  1   1   0
## 444     444    0 480 24   1    1  1   1   0
## 445     445    0 480 24   1    1  1   1   0
## 446     446    0 480 24   1    1  1   1   0
## 447     447    0 480 24   1    1  1   1   0
## 448     448    0 480 24   1    1  1   1   0
## 449     449    0 480 24   1    1  1   1   0
## 450     450    0 480 24   1    1  1   1   0
## 451     451    0 480 24   1    1  1   1   0
## 452     452    0 480 24   1    1  1   1   0
## 453     453    0 480 24   1    1  1   1   0
## 454     454    0 480 24   1    1  1   1   0
## 455     455    0 480 24   1    1  1   1   0
## 456     456    0 480 24   1    1  1   1   0
## 457     457    0 480 24   1    1  1   1   0
## 458     458    0 480 24   1    1  1   1   0
## 459     459    0 480 24   1    1  1   1   0
## 460     460    0 480 24   1    1  1   1   0
## 461     461    0 480 24   1    1  1   1   0
## 462     462    0 480 24   1    1  1   1   0
## 463     463    0 480 24   1    1  1   1   0
## 464     464    0 480 24   1    1  1   1   0
## 465     465    0 480 24   1    1  1   1   0
## 466     466    0 480 24   1    1  1   1   0
## 467     467    0 480 24   1    1  1   1   0
## 468     468    0 480 24   1    1  1   1   0
## 469     469    0 480 24   1    1  1   1   0
## 470     470    0 480 24   1    1  1   1   0
## 471     471    0 480 24   1    1  1   1   0
## 472     472    0 480 24   1    1  1   1   0
## 473     473    0 480 24   1    1  1   1   0
## 474     474    0 480 24   1    1  1   1   0
## 475     475    0 480 24   1    1  1   1   0
## 476     476    0 480 24   1    1  1   1   0
## 477     477    0 480 24   1    1  1   1   0
## 478     478    0 480 24   1    1  1   1   0
## 479     479    0 480 24   1    1  1   1   0
## 480     480    0 480 24   1    1  1   1   0
## 481     481    0 480 24   1    1  1   1   0
## 482     482    0 480 24   1    1  1   1   0
## 483     483    0 480 24   1    1  1   1   0
## 484     484    0 480 24   1    1  1   1   0
## 485     485    0 480 24   1    1  1   1   0
## 486     486    0 480 24   1    1  1   1   0
## 487     487    0 480 24   1    1  1   1   0
## 488     488    0 480 24   1    1  1   1   0
## 489     489    0 480 24   1    1  1   1   0
## 490     490    0 480 24   1    1  1   1   0
## 491     491    0 480 24   1    1  1   1   0
## 492     492    0 480 24   1    1  1   1   0
## 493     493    0 480 24   1    1  1   1   0
## 494     494    0 480 24   1    1  1   1   0
## 495     495    0 480 24   1    1  1   1   0
## 496     496    0 480 24   1    1  1   1   0
## 497     497    0 480 24   1    1  1   1   0
## 498     498    0 480 24   1    1  1   1   0
## 499     499    0 480 24   1    1  1   1   0
## 500     500    0 480 24   1    1  1   1   0
## 501     501    0 480 24   1    1  1   1   0
## 502     502    0 480 24   1    1  1   1   0
## 503     503    0 480 24   1    1  1   1   0
## 504     504    0 480 24   1    1  1   1   0
## 505     505    0 480 24   1    1  1   1   0
## 506     506    0 480 24   1    1  1   1   0
## 507     507    0 480 24   1    1  1   1   0
## 508     508    0 480 24   1    1  1   1   0
## 509     509    0 480 24   1    1  1   1   0
## 510     510    0 480 24   1    1  1   1   0
## 511     511    0 480 24   1    1  1   1   0
## 512     512    0 480 24   1    1  1   1   0
## 513     513    0 480 24   1    1  1   1   0
## 514     514    0 480 24   1    1  1   1   0
## 515     515    0 480 24   1    1  1   1   0
## 516     516    0 480 24   1    1  1   1   0
## 517     517    0 480 24   1    1  1   1   0
## 518     518    0 480 24   1    1  1   1   0
## 519     519    0 480 24   1    1  1   1   0
## 520     520    0 480 24   1    1  1   1   0
## 521     521    0 480 24   1    1  1   1   0
## 522     522    0 480 24   1    1  1   1   0
## 523     523    0 480 24   1    1  1   1   0
## 524     524    0 480 24   1    1  1   1   0
## 525     525    0 480 24   1    1  1   1   0
## 526     526    0 480 24   1    1  1   1   0
## 527     527    0 480 24   1    1  1   1   0
## 528     528    0 480 24   1    1  1   1   0
## 529     529    0 480 24   1    1  1   1   0
## 530     530    0 480 24   1    1  1   1   0
## 531     531    0 480 24   1    1  1   1   0
## 532     532    0 480 24   1    1  1   1   0
## 533     533    0 480 24   1    1  1   1   0
## 534     534    0 480 24   1    1  1   1   0
## 535     535    0 480 24   1    1  1   1   0
## 536     536    0 480 24   1    1  1   1   0
## 537     537    0 480 24   1    1  1   1   0
## 538     538    0 480 24   1    1  1   1   0
## 539     539    0 480 24   1    1  1   1   0
## 540     540    0 480 24   1    1  1   1   0
## 541     541    0 480 24   1    1  1   1   0
## 542     542    0 480 24   1    1  1   1   0
## 543     543    0 480 24   1    1  1   1   0
## 544     544    0 480 24   1    1  1   1   0
## 545     545    0 480 24   1    1  1   1   0
## 546     546    0 480 24   1    1  1   1   0
## 547     547    0 480 24   1    1  1   1   0
## 548     548    0 480 24   1    1  1   1   0
## 549     549    0 480 24   1    1  1   1   0
## 550     550    0 480 24   1    1  1   1   0
## 551     551    0 480 24   1    1  1   1   0
## 552     552    0 480 24   1    1  1   1   0
## 553     553    0 480 24   1    1  1   1   0
## 554     554    0 480 24   1    1  1   1   0
## 555     555    0 480 24   1    1  1   1   0
## 556     556    0 480 24   1    1  1   1   0
## 557     557    0 480 24   1    1  1   1   0
## 558     558    0 480 24   1    1  1   1   0
## 559     559    0 480 24   1    1  1   1   0
## 560     560    0 480 24   1    1  1   1   0
## 561     561    0 480 24   1    1  1   1   0
## 562     562    0 480 24   1    1  1   1   0
## 563     563    0 480 24   1    1  1   1   0
## 564     564    0 480 24   1    1  1   1   0
## 565     565    0 480 24   1    1  1   1   0
## 566     566    0 480 24   1    1  1   1   0
## 567     567    0 480 24   1    1  1   1   0
## 568     568    0 480 24   1    1  1   1   0
## 569     569    0 480 24   1    1  1   1   0
## 570     570    0 480 24   1    1  1   1   0
## 571     571    0 480 24   1    1  1   1   0
## 572     572    0 480 24   1    1  1   1   0
## 573     573    0 480 24   1    1  1   1   0
## 574     574    0 480 24   1    1  1   1   0
## 575     575    0 480 24   1    1  1   1   0
## 576     576    0 480 24   1    1  1   1   0
## 577     577    0 480 24   1    1  1   1   0
## 578     578    0 480 24   1    1  1   1   0
## 579     579    0 480 24   1    1  1   1   0
## 580     580    0 480 24   1    1  1   1   0
## 581     581    0 480 24   1    1  1   1   0
## 582     582    0 480 24   1    1  1   1   0
## 583     583    0 480 24   1    1  1   1   0
## 584     584    0 480 24   1    1  1   1   0
## 585     585    0 480 24   1    1  1   1   0
## 586     586    0 480 24   1    1  1   1   0
## 587     587    0 480 24   1    1  1   1   0
## 588     588    0 480 24   1    1  1   1   0
## 589     589    0 480 24   1    1  1   1   0
## 590     590    0 480 24   1    1  1   1   0
## 591     591    0 480 24   1    1  1   1   0
## 592     592    0 480 24   1    1  1   1   0
## 593     593    0 480 24   1    1  1   1   0
## 594     594    0 480 24   1    1  1   1   0
## 595     595    0 480 24   1    1  1   1   0
## 596     596    0 480 24   1    1  1   1   0
## 597     597    0 480 24   1    1  1   1   0
## 598     598    0 480 24   1    1  1   1   0
## 599     599    0 480 24   1    1  1   1   0
## 600     600    0 480 24   1    1  1   1   0
## 601     601    0 480 24   1    1  1   1   0
## 602     602    0 480 24   1    1  1   1   0
## 603     603    0 480 24   1    1  1   1   0
## 604     604    0 480 24   1    1  1   1   0
## 605     605    0 480 24   1    1  1   1   0
## 606     606    0 480 24   1    1  1   1   0
## 607     607    0 480 24   1    1  1   1   0
## 608     608    0 480 24   1    1  1   1   0
## 609     609    0 480 24   1    1  1   1   0
## 610     610    0 480 24   1    1  1   1   0
## 611     611    0 480 24   1    1  1   1   0
## 612     612    0 480 24   1    1  1   1   0
## 613     613    0 480 24   1    1  1   1   0
## 614     614    0 480 24   1    1  1   1   0
## 615     615    0 480 24   1    1  1   1   0
## 616     616    0 480 24   1    1  1   1   0
## 617     617    0 480 24   1    1  1   1   0
## 618     618    0 480 24   1    1  1   1   0
## 619     619    0 480 24   1    1  1   1   0
## 620     620    0 480 24   1    1  1   1   0
## 621     621    0 480 24   1    1  1   1   0
## 622     622    0 480 24   1    1  1   1   0
## 623     623    0 480 24   1    1  1   1   0
## 624     624    0 480 24   1    1  1   1   0
## 625     625    0 480 24   1    1  1   1   0
## 626     626    0 480 24   1    1  1   1   0
## 627     627    0 480 24   1    1  1   1   0
## 628     628    0 480 24   1    1  1   1   0
## 629     629    0 480 24   1    1  1   1   0
## 630     630    0 480 24   1    1  1   1   0
## 631     631    0 480 24   1    1  1   1   0
## 632     632    0 480 24   1    1  1   1   0
## 633     633    0 480 24   1    1  1   1   0
## 634     634    0 480 24   1    1  1   1   0
## 635     635    0 480 24   1    1  1   1   0
## 636     636    0 480 24   1    1  1   1   0
## 637     637    0 480 24   1    1  1   1   0
## 638     638    0 480 24   1    1  1   1   0
## 639     639    0 480 24   1    1  1   1   0
## 640     640    0 480 24   1    1  1   1   0
## 641     641    0 480 24   1    1  1   1   0
## 642     642    0 480 24   1    1  1   1   0
## 643     643    0 480 24   1    1  1   1   0
## 644     644    0 480 24   1    1  1   1   0
## 645     645    0 480 24   1    1  1   1   0
## 646     646    0 480 24   1    1  1   1   0
## 647     647    0 480 24   1    1  1   1   0
## 648     648    0 480 24   1    1  1   1   0
## 649     649    0 480 24   1    1  1   1   0
## 650     650    0 480 24   1    1  1   1   0
## 651     651    0 480 24   1    1  1   1   0
## 652     652    0 480 24   1    1  1   1   0
## 653     653    0 480 24   1    1  1   1   0
## 654     654    0 480 24   1    1  1   1   0
## 655     655    0 480 24   1    1  1   1   0
## 656     656    0 480 24   1    1  1   1   0
## 657     657    0 480 24   1    1  1   1   0
## 658     658    0 480 24   1    1  1   1   0
## 659     659    0 480 24   1    1  1   1   0
## 660     660    0 480 24   1    1  1   1   0
## 661     661    0 480 24   1    1  1   1   0
## 662     662    0 480 24   1    1  1   1   0
## 663     663    0 480 24   1    1  1   1   0
## 664     664    0 480 24   1    1  1   1   0
## 665     665    0 480 24   1    1  1   1   0
## 666     666    0 480 24   1    1  1   1   0
## 667     667    0 480 24   1    1  1   1   0
## 668     668    0 480 24   1    1  1   1   0
## 669     669    0 480 24   1    1  1   1   0
## 670     670    0 480 24   1    1  1   1   0
## 671     671    0 480 24   1    1  1   1   0
## 672     672    0 480 24   1    1  1   1   0
## 673     673    0 480 24   1    1  1   1   0
## 674     674    0 480 24   1    1  1   1   0
## 675     675    0 480 24   1    1  1   1   0
## 676     676    0 480 24   1    1  1   1   0
## 677     677    0 480 24   1    1  1   1   0
## 678     678    0 480 24   1    1  1   1   0
## 679     679    0 480 24   1    1  1   1   0
## 680     680    0 480 24   1    1  1   1   0
## 681     681    0 480 24   1    1  1   1   0
## 682     682    0 480 24   1    1  1   1   0
## 683     683    0 480 24   1    1  1   1   0
## 684     684    0 480 24   1    1  1   1   0
## 685     685    0 480 24   1    1  1   1   0
## 686     686    0 480 24   1    1  1   1   0
## 687     687    0 480 24   1    1  1   1   0
## 688     688    0 480 24   1    1  1   1   0
## 689     689    0 480 24   1    1  1   1   0
## 690     690    0 480 24   1    1  1   1   0
## 691     691    0 480 24   1    1  1   1   0
## 692     692    0 480 24   1    1  1   1   0
## 693     693    0 480 24   1    1  1   1   0
## 694     694    0 480 24   1    1  1   1   0
## 695     695    0 480 24   1    1  1   1   0
## 696     696    0 480 24   1    1  1   1   0
## 697     697    0 480 24   1    1  1   1   0
## 698     698    0 480 24   1    1  1   1   0
## 699     699    0 480 24   1    1  1   1   0
## 700     700    0 480 24   1    1  1   1   0
## 701     701    0 480 24   1    1  1   1   0
## 702     702    0 480 24   1    1  1   1   0
## 703     703    0 480 24   1    1  1   1   0
## 704     704    0 480 24   1    1  1   1   0
## 705     705    0 480 24   1    1  1   1   0
## 706     706    0 480 24   1    1  1   1   0
## 707     707    0 480 24   1    1  1   1   0
## 708     708    0 480 24   1    1  1   1   0
## 709     709    0 480 24   1    1  1   1   0
## 710     710    0 480 24   1    1  1   1   0
## 711     711    0 480 24   1    1  1   1   0
## 712     712    0 480 24   1    1  1   1   0
## 713     713    0 480 24   1    1  1   1   0
## 714     714    0 480 24   1    1  1   1   0
## 715     715    0 480 24   1    1  1   1   0
## 716     716    0 480 24   1    1  1   1   0
## 717     717    0 480 24   1    1  1   1   0
## 718     718    0 480 24   1    1  1   1   0
## 719     719    0 480 24   1    1  1   1   0
## 720     720    0 480 24   1    1  1   1   0
## 721     721    0 480 24   1    1  1   1   0
## 722     722    0 480 24   1    1  1   1   0
## 723     723    0 480 24   1    1  1   1   0
## 724     724    0 480 24   1    1  1   1   0
## 725     725    0 480 24   1    1  1   1   0
## 726     726    0 480 24   1    1  1   1   0
## 727     727    0 480 24   1    1  1   1   0
## 728     728    0 480 24   1    1  1   1   0
## 729     729    0 480 24   1    1  1   1   0
## 730     730    0 480 24   1    1  1   1   0
## 731     731    0 480 24   1    1  1   1   0
## 732     732    0 480 24   1    1  1   1   0
## 733     733    0 480 24   1    1  1   1   0
## 734     734    0 480 24   1    1  1   1   0
## 735     735    0 480 24   1    1  1   1   0
## 736     736    0 480 24   1    1  1   1   0
## 737     737    0 480 24   1    1  1   1   0
## 738     738    0 480 24   1    1  1   1   0
## 739     739    0 480 24   1    1  1   1   0
## 740     740    0 480 24   1    1  1   1   0
## 741     741    0 480 24   1    1  1   1   0
## 742     742    0 480 24   1    1  1   1   0
## 743     743    0 480 24   1    1  1   1   0
## 744     744    0 480 24   1    1  1   1   0
## 745     745    0 480 24   1    1  1   1   0
## 746     746    0 480 24   1    1  1   1   0
## 747     747    0 480 24   1    1  1   1   0
## 748     748    0 480 24   1    1  1   1   0
## 749     749    0 480 24   1    1  1   1   0
## 750     750    0 480 24   1    1  1   1   0
## 751     751    0 480 24   1    1  1   1   0
## 752     752    0 480 24   1    1  1   1   0
## 753     753    0 480 24   1    1  1   1   0
## 754     754    0 480 24   1    1  1   1   0
## 755     755    0 480 24   1    1  1   1   0
## 756     756    0 480 24   1    1  1   1   0
## 757     757    0 480 24   1    1  1   1   0
## 758     758    0 480 24   1    1  1   1   0
## 759     759    0 480 24   1    1  1   1   0
## 760     760    0 480 24   1    1  1   1   0
## 761     761    0 480 24   1    1  1   1   0
## 762     762    0 480 24   1    1  1   1   0
## 763     763    0 480 24   1    1  1   1   0
## 764     764    0 480 24   1    1  1   1   0
## 765     765    0 480 24   1    1  1   1   0
## 766     766    0 480 24   1    1  1   1   0
## 767     767    0 480 24   1    1  1   1   0
## 768     768    0 480 24   1    1  1   1   0
## 769     769    0 480 24   1    1  1   1   0
## 770     770    0 480 24   1    1  1   1   0
## 771     771    0 480 24   1    1  1   1   0
## 772     772    0 480 24   1    1  1   1   0
## 773     773    0 480 24   1    1  1   1   0
## 774     774    0 480 24   1    1  1   1   0
## 775     775    0 480 24   1    1  1   1   0
## 776     776    0 480 24   1    1  1   1   0
## 777     777    0 480 24   1    1  1   1   0
## 778     778    0 480 24   1    1  1   1   0
## 779     779    0 480 24   1    1  1   1   0
## 780     780    0 480 24   1    1  1   1   0
## 781     781    0 480 24   1    1  1   1   0
## 782     782    0 480 24   1    1  1   1   0
## 783     783    0 480 24   1    1  1   1   0
## 784     784    0 480 24   1    1  1   1   0
## 785     785    0 480 24   1    1  1   1   0
## 786     786    0 480 24   1    1  1   1   0
## 787     787    0 480 24   1    1  1   1   0
## 788     788    0 480 24   1    1  1   1   0
## 789     789    0 480 24   1    1  1   1   0
## 790     790    0 480 24   1    1  1   1   0
## 791     791    0 480 24   1    1  1   1   0
## 792     792    0 480 24   1    1  1   1   0
## 793     793    0 480 24   1    1  1   1   0
## 794     794    0 480 24   1    1  1   1   0
## 795     795    0 480 24   1    1  1   1   0
## 796     796    0 480 24   1    1  1   1   0
## 797     797    0 480 24   1    1  1   1   0
## 798     798    0 480 24   1    1  1   1   0
## 799     799    0 480 24   1    1  1   1   0
## 800     800    0 480 24   1    1  1   1   0
## 801     801    0 480 24   1    1  1   1   0
## 802     802    0 480 24   1    1  1   1   0
## 803     803    0 480 24   1    1  1   1   0
## 804     804    0 480 24   1    1  1   1   0
## 805     805    0 480 24   1    1  1   1   0
## 806     806    0 480 24   1    1  1   1   0
## 807     807    0 480 24   1    1  1   1   0
## 808     808    0 480 24   1    1  1   1   0
## 809     809    0 480 24   1    1  1   1   0
## 810     810    0 480 24   1    1  1   1   0
## 811     811    0 480 24   1    1  1   1   0
## 812     812    0 480 24   1    1  1   1   0
## 813     813    0 480 24   1    1  1   1   0
## 814     814    0 480 24   1    1  1   1   0
## 815     815    0 480 24   1    1  1   1   0
## 816     816    0 480 24   1    1  1   1   0
## 817     817    0 480 24   1    1  1   1   0
## 818     818    0 480 24   1    1  1   1   0
## 819     819    0 480 24   1    1  1   1   0
## 820     820    0 480 24   1    1  1   1   0
## 821     821    0 480 24   1    1  1   1   0
## 822     822    0 480 24   1    1  1   1   0
## 823     823    0 480 24   1    1  1   1   0
## 824     824    0 480 24   1    1  1   1   0
## 825     825    0 480 24   1    1  1   1   0
## 826     826    0 480 24   1    1  1   1   0
## 827     827    0 480 24   1    1  1   1   0
## 828     828    0 480 24   1    1  1   1   0
## 829     829    0 480 24   1    1  1   1   0
## 830     830    0 480 24   1    1  1   1   0
## 831     831    0 480 24   1    1  1   1   0
## 832     832    0 480 24   1    1  1   1   0
## 833     833    0 480 24   1    1  1   1   0
## 834     834    0 480 24   1    1  1   1   0
## 835     835    0 480 24   1    1  1   1   0
## 836     836    0 480 24   1    1  1   1   0
## 837     837    0 480 24   1    1  1   1   0
## 838     838    0 480 24   1    1  1   1   0
## 839     839    0 480 24   1    1  1   1   0
## 840     840    0 480 24   1    1  1   1   0
## 841     841    0 480 24   1    1  1   1   0
## 842     842    0 480 24   1    1  1   1   0
## 843     843    0 480 24   1    1  1   1   0
## 844     844    0 480 24   1    1  1   1   0
## 845     845    0 480 24   1    1  1   1   0
## 846     846    0 480 24   1    1  1   1   0
## 847     847    0 480 24   1    1  1   1   0
## 848     848    0 480 24   1    1  1   1   0
## 849     849    0 480 24   1    1  1   1   0
## 850     850    0 480 24   1    1  1   1   0
## 851     851    0 480 24   1    1  1   1   0
## 852     852    0 480 24   1    1  1   1   0
## 853     853    0 480 24   1    1  1   1   0
## 854     854    0 480 24   1    1  1   1   0
## 855     855    0 480 24   1    1  1   1   0
## 856     856    0 480 24   1    1  1   1   0
## 857     857    0 480 24   1    1  1   1   0
## 858     858    0 480 24   1    1  1   1   0
## 859     859    0 480 24   1    1  1   1   0
## 860     860    0 480 24   1    1  1   1   0
## 861     861    0 480 24   1    1  1   1   0
## 862     862    0 480 24   1    1  1   1   0
## 863     863    0 480 24   1    1  1   1   0
## 864     864    0 480 24   1    1  1   1   0
## 865     865    0 480 24   1    1  1   1   0
## 866     866    0 480 24   1    1  1   1   0
## 867     867    0 480 24   1    1  1   1   0
## 868     868    0 480 24   1    1  1   1   0
## 869     869    0 480 24   1    1  1   1   0
## 870     870    0 480 24   1    1  1   1   0
## 871     871    0 480 24   1    1  1   1   0
## 872     872    0 480 24   1    1  1   1   0
## 873     873    0 480 24   1    1  1   1   0
## 874     874    0 480 24   1    1  1   1   0
## 875     875    0 480 24   1    1  1   1   0
## 876     876    0 480 24   1    1  1   1   0
## 877     877    0 480 24   1    1  1   1   0
## 878     878    0 480 24   1    1  1   1   0
## 879     879    0 480 24   1    1  1   1   0
## 880     880    0 480 24   1    1  1   1   0
## 881     881    0 480 24   1    1  1   1   0
## 882     882    0 480 24   1    1  1   1   0
## 883     883    0 480 24   1    1  1   1   0
## 884     884    0 480 24   1    1  1   1   0
## 885     885    0 480 24   1    1  1   1   0
## 886     886    0 480 24   1    1  1   1   0
## 887     887    0 480 24   1    1  1   1   0
## 888     888    0 480 24   1    1  1   1   0
## 889     889    0 480 24   1    1  1   1   0
## 890     890    0 480 24   1    1  1   1   0
## 891     891    0 480 24   1    1  1   1   0
## 892     892    0 480 24   1    1  1   1   0
## 893     893    0 480 24   1    1  1   1   0
## 894     894    0 480 24   1    1  1   1   0
## 895     895    0 480 24   1    1  1   1   0
## 896     896    0 480 24   1    1  1   1   0
## 897     897    0 480 24   1    1  1   1   0
## 898     898    0 480 24   1    1  1   1   0
## 899     899    0 480 24   1    1  1   1   0
## 900     900    0 480 24   1    1  1   1   0
## 901     901    0 480 24   1    1  1   1   0
## 902     902    0 480 24   1    1  1   1   0
## 903     903    0 480 24   1    1  1   1   0
## 904     904    0 480 24   1    1  1   1   0
## 905     905    0 480 24   1    1  1   1   0
## 906     906    0 480 24   1    1  1   1   0
## 907     907    0 480 24   1    1  1   1   0
## 908     908    0 480 24   1    1  1   1   0
## 909     909    0 480 24   1    1  1   1   0
## 910     910    0 480 24   1    1  1   1   0
## 911     911    0 480 24   1    1  1   1   0
## 912     912    0 480 24   1    1  1   1   0
## 913     913    0 480 24   1    1  1   1   0
## 914     914    0 480 24   1    1  1   1   0
## 915     915    0 480 24   1    1  1   1   0
## 916     916    0 480 24   1    1  1   1   0
## 917     917    0 480 24   1    1  1   1   0
## 918     918    0 480 24   1    1  1   1   0
## 919     919    0 480 24   1    1  1   1   0
## 920     920    0 480 24   1    1  1   1   0
## 921     921    0 480 24   1    1  1   1   0
## 922     922    0 480 24   1    1  1   1   0
## 923     923    0 480 24   1    1  1   1   0
## 924     924    0 480 24   1    1  1   1   0
## 925     925    0 480 24   1    1  1   1   0
## 926     926    0 480 24   1    1  1   1   0
## 927     927    0 480 24   1    1  1   1   0
## 928     928    0 480 24   1    1  1   1   0
## 929     929    0 480 24   1    1  1   1   0
## 930     930    0 480 24   1    1  1   1   0
## 931     931    0 480 24   1    1  1   1   0
## 932     932    0 480 24   1    1  1   1   0
## 933     933    0 480 24   1    1  1   1   0
## 934     934    0 480 24   1    1  1   1   0
## 935     935    0 480 24   1    1  1   1   0
## 936     936    0 480 24   1    1  1   1   0
## 937     937    0 480 24   1    1  1   1   0
## 938     938    0 480 24   1    1  1   1   0
## 939     939    0 480 24   1    1  1   1   0
## 940     940    0 480 24   1    1  1   1   0
## 941     941    0 480 24   1    1  1   1   0
## 942     942    0 480 24   1    1  1   1   0
## 943     943    0 480 24   1    1  1   1   0
## 944     944    0 480 24   1    1  1   1   0
## 945     945    0 480 24   1    1  1   1   0
## 946     946    0 480 24   1    1  1   1   0
## 947     947    0 480 24   1    1  1   1   0
## 948     948    0 480 24   1    1  1   1   0
## 949     949    0 480 24   1    1  1   1   0
## 950     950    0 480 24   1    1  1   1   0
## 951     951    0 480 24   1    1  1   1   0
## 952     952    0 480 24   1    1  1   1   0
## 953     953    0 480 24   1    1  1   1   0
## 954     954    0 480 24   1    1  1   1   0
## 955     955    0 480 24   1    1  1   1   0
## 956     956    0 480 24   1    1  1   1   0
## 957     957    0 480 24   1    1  1   1   0
## 958     958    0 480 24   1    1  1   1   0
## 959     959    0 480 24   1    1  1   1   0
## 960     960    0 480 24   1    1  1   1   0
## 961     961    0 480 24   1    1  1   1   0
## 962     962    0 480 24   1    1  1   1   0
## 963     963    0 480 24   1    1  1   1   0
## 964     964    0 480 24   1    1  1   1   0
## 965     965    0 480 24   1    1  1   1   0
## 966     966    0 480 24   1    1  1   1   0
## 967     967    0 480 24   1    1  1   1   0
## 968     968    0 480 24   1    1  1   1   0
## 969     969    0 480 24   1    1  1   1   0
## 970     970    0 480 24   1    1  1   1   0
## 971     971    0 480 24   1    1  1   1   0
## 972     972    0 480 24   1    1  1   1   0
## 973     973    0 480 24   1    1  1   1   0
## 974     974    0 480 24   1    1  1   1   0
## 975     975    0 480 24   1    1  1   1   0
## 976     976    0 480 24   1    1  1   1   0
## 977     977    0 480 24   1    1  1   1   0
## 978     978    0 480 24   1    1  1   1   0
## 979     979    0 480 24   1    1  1   1   0
## 980     980    0 480 24   1    1  1   1   0
## 981     981    0 480 24   1    1  1   1   0
## 982     982    0 480 24   1    1  1   1   0
## 983     983    0 480 24   1    1  1   1   0
## 984     984    0 480 24   1    1  1   1   0
## 985     985    0 480 24   1    1  1   1   0
## 986     986    0 480 24   1    1  1   1   0
## 987     987    0 480 24   1    1  1   1   0
## 988     988    0 480 24   1    1  1   1   0
## 989     989    0 480 24   1    1  1   1   0
## 990     990    0 480 24   1    1  1   1   0
## 991     991    0 480 24   1    1  1   1   0
## 992     992    0 480 24   1    1  1   1   0
## 993     993    0 480 24   1    1  1   1   0
## 994     994    0 480 24   1    1  1   1   0
## 995     995    0 480 24   1    1  1   1   0
## 996     996    0 480 24   1    1  1   1   0
## 997     997    0 480 24   1    1  1   1   0
## 998     998    0 480 24   1    1  1   1   0
## 999     999    0 480 24   1    1  1   1   0
## 1000   1000    0 480 24   1    1  1   1   0
## 1001   1001    0 480 24   1    1  1   1   0
## 1002   1002    0 480 24   1    1  1   1   0
## 1003   1003    0 480 24   1    1  1   1   0
## 1004   1004    0 480 24   1    1  1   1   0
## 1005   1005    0 480 24   1    1  1   1   0
## 1006   1006    0 480 24   1    1  1   1   0
## 1007   1007    0 480 24   1    1  1   1   0
## 1008   1008    0 480 24   1    1  1   1   0
## 1009   1009    0 480 24   1    1  1   1   0
## 1010   1010    0 480 24   1    1  1   1   0
## 1011   1011    0 480 24   1    1  1   1   0
## 1012   1012    0 480 24   1    1  1   1   0
## 1013   1013    0 480 24   1    1  1   1   0
## 1014   1014    0 480 24   1    1  1   1   0
## 1015   1015    0 480 24   1    1  1   1   0
## 1016   1016    0 480 24   1    1  1   1   0
## 1017   1017    0 480 24   1    1  1   1   0
## 1018   1018    0 480 24   1    1  1   1   0
## 1019   1019    0 480 24   1    1  1   1   0
## 1020   1020    0 480 24   1    1  1   1   0
## 1021   1021    0 480 24   1    1  1   1   0
## 1022   1022    0 480 24   1    1  1   1   0
## 1023   1023    0 480 24   1    1  1   1   0
## 1024   1024    0 480 24   1    1  1   1   0
## 1025   1025    0 480 24   1    1  1   1   0
## 1026   1026    0 480 24   1    1  1   1   0
## 1027   1027    0 480 24   1    1  1   1   0
## 1028   1028    0 480 24   1    1  1   1   0
## 1029   1029    0 480 24   1    1  1   1   0
## 1030   1030    0 480 24   1    1  1   1   0
## 1031   1031    0 480 24   1    1  1   1   0
## 1032   1032    0 480 24   1    1  1   1   0
## 1033   1033    0 480 24   1    1  1   1   0
## 1034   1034    0 480 24   1    1  1   1   0
## 1035   1035    0 480 24   1    1  1   1   0
## 1036   1036    0 480 24   1    1  1   1   0
## 1037   1037    0 480 24   1    1  1   1   0
## 1038   1038    0 480 24   1    1  1   1   0
## 1039   1039    0 480 24   1    1  1   1   0
## 1040   1040    0 480 24   1    1  1   1   0
## 1041   1041    0 480 24   1    1  1   1   0
## 1042   1042    0 480 24   1    1  1   1   0
## 1043   1043    0 480 24   1    1  1   1   0
## 1044   1044    0 480 24   1    1  1   1   0
## 1045   1045    0 480 24   1    1  1   1   0
## 1046   1046    0 480 24   1    1  1   1   0
## 1047   1047    0 480 24   1    1  1   1   0
## 1048   1048    0 480 24   1    1  1   1   0
## 1049   1049    0 480 24   1    1  1   1   0
## 1050   1050    0 480 24   1    1  1   1   0
## 1051   1051    0 480 24   1    1  1   1   0
## 1052   1052    0 480 24   1    1  1   1   0
## 1053   1053    0 480 24   1    1  1   1   0
## 1054   1054    0 480 24   1    1  1   1   0
## 1055   1055    0 480 24   1    1  1   1   0
## 1056   1056    0 480 24   1    1  1   1   0
## 1057   1057    0 480 24   1    1  1   1   0
## 1058   1058    0 480 24   1    1  1   1   0
## 1059   1059    0 480 24   1    1  1   1   0
## 1060   1060    0 480 24   1    1  1   1   0
## 1061   1061    0 480 24   1    1  1   1   0
## 1062   1062    0 480 24   1    1  1   1   0
## 1063   1063    0 480 24   1    1  1   1   0
## 1064   1064    0 480 24   1    1  1   1   0
## 1065   1065    0 480 24   1    1  1   1   0
## 1066   1066    0 480 24   1    1  1   1   0
## 1067   1067    0 480 24   1    1  1   1   0
## 1068   1068    0 480 24   1    1  1   1   0
## 1069   1069    0 480 24   1    1  1   1   0
## 1070   1070    0 480 24   1    1  1   1   0
## 1071   1071    0 480 24   1    1  1   1   0
## 1072   1072    0 480 24   1    1  1   1   0
## 1073   1073    0 480 24   1    1  1   1   0
## 1074   1074    0 480 24   1    1  1   1   0
## 1075   1075    0 480 24   1    1  1   1   0
## 1076   1076    0 480 24   1    1  1   1   0
## 1077   1077    0 480 24   1    1  1   1   0
## 1078   1078    0 480 24   1    1  1   1   0
## 1079   1079    0 480 24   1    1  1   1   0
## 1080   1080    0 480 24   1    1  1   1   0
## 1081   1081    0 480 24   1    1  1   1   0
## 1082   1082    0 480 24   1    1  1   1   0
## 1083   1083    0 480 24   1    1  1   1   0
## 1084   1084    0 480 24   1    1  1   1   0
## 1085   1085    0 480 24   1    1  1   1   0
## 1086   1086    0 480 24   1    1  1   1   0
## 1087   1087    0 480 24   1    1  1   1   0
## 1088   1088    0 480 24   1    1  1   1   0
## 1089   1089    0 480 24   1    1  1   1   0
## 1090   1090    0 480 24   1    1  1   1   0
## 1091   1091    0 480 24   1    1  1   1   0
## 1092   1092    0 480 24   1    1  1   1   0
## 1093   1093    0 480 24   1    1  1   1   0
## 1094   1094    0 480 24   1    1  1   1   0
## 1095   1095    0 480 24   1    1  1   1   0
## 1096   1096    0 480 24   1    1  1   1   0
## 1097   1097    0 480 24   1    1  1   1   0
## 1098   1098    0 480 24   1    1  1   1   0
## 1099   1099    0 480 24   1    1  1   1   0
## 1100   1100    0 480 24   1    1  1   1   0
## 1101   1101    0 480 24   1    1  1   1   0
## 1102   1102    0 480 24   1    1  1   1   0
## 1103   1103    0 480 24   1    1  1   1   0
## 1104   1104    0 480 24   1    1  1   1   0
## 1105   1105    0 480 24   1    1  1   1   0
## 1106   1106    0 480 24   1    1  1   1   0
## 1107   1107    0 480 24   1    1  1   1   0
## 1108   1108    0 480 24   1    1  1   1   0
## 1109   1109    0 480 24   1    1  1   1   0
## 1110   1110    0 480 24   1    1  1   1   0
## 1111   1111    0 480 24   1    1  1   1   0
## 1112   1112    0 480 24   1    1  1   1   0
## 1113   1113    0 480 24   1    1  1   1   0
## 1114   1114    0 480 24   1    1  1   1   0
## 1115   1115    0 480 24   1    1  1   1   0
## 1116   1116    0 480 24   1    1  1   1   0
## 1117   1117    0 480 24   1    1  1   1   0
## 1118   1118    0 480 24   1    1  1   1   0
## 1119   1119    0 480 24   1    1  1   1   0
## 1120   1120    0 480 24   1    1  1   1   0
## 1121   1121    0 480 24   1    1  1   1   0
## 1122   1122    0 480 24   1    1  1   1   0
## 1123   1123    0 480 24   1    1  1   1   0
## 1124   1124    0 480 24   1    1  1   1   0
## 1125   1125    0 480 24   1    1  1   1   0
## 1126   1126    0 480 24   1    1  1   1   0
## 1127   1127    0 480 24   1    1  1   1   0
## 1128   1128    0 480 24   1    1  1   1   0
## 1129   1129    0 480 24   1    1  1   1   0
## 1130   1130    0 480 24   1    1  1   1   0
## 1131   1131    0 480 24   1    1  1   1   0
## 1132   1132    0 480 24   1    1  1   1   0
## 1133   1133    0 480 24   1    1  1   1   0
## 1134   1134    0 480 24   1    1  1   1   0
## 1135   1135    0 480 24   1    1  1   1   0
## 1136   1136    0 480 24   1    1  1   1   0
## 1137   1137    0 480 24   1    1  1   1   0
## 1138   1138    0 480 24   1    1  1   1   0
## 1139   1139    0 480 24   1    1  1   1   0
## 1140   1140    0 480 24   1    1  1   1   0
## 1141   1141    0 480 24   1    1  1   1   0
## 1142   1142    0 480 24   1    1  1   1   0
## 1143   1143    0 480 24   1    1  1   1   0
## 1144   1144    0 480 24   1    1  1   1   0
## 1145   1145    0 480 24   1    1  1   1   0
## 1146   1146    0 480 24   1    1  1   1   0
## 1147   1147    0 480 24   1    1  1   1   0
## 1148   1148    0 480 24   1    1  1   1   0
## 1149   1149    0 480 24   1    1  1   1   0
## 1150   1150    0 480 24   1    1  1   1   0
## 1151   1151    0 480 24   1    1  1   1   0
## 1152   1152    0 480 24   1    1  1   1   0
## 1153   1153    0 480 24   1    1  1   1   0
## 1154   1154    0 480 24   1    1  1   1   0
## 1155   1155    0 480 24   1    1  1   1   0
## 1156   1156    0 480 24   1    1  1   1   0
## 1157   1157    0 480 24   1    1  1   1   0
## 1158   1158    0 480 24   1    1  1   1   0
## 1159   1159    0 480 24   1    1  1   1   0
## 1160   1160    0 480 24   1    1  1   1   0
## 1161   1161    0 480 24   1    1  1   1   0
## 1162   1162    0 480 24   1    1  1   1   0
## 1163   1163    0 480 24   1    1  1   1   0
## 1164   1164    0 480 24   1    1  1   1   0
## 1165   1165    0 480 24   1    1  1   1   0
## 1166   1166    0 480 24   1    1  1   1   0
## 1167   1167    0 480 24   1    1  1   1   0
## 1168   1168    0 480 24   1    1  1   1   0
## 1169   1169    0 480 24   1    1  1   1   0
## 1170   1170    0 480 24   1    1  1   1   0
## 1171   1171    0 480 24   1    1  1   1   0
## 1172   1172    0 480 24   1    1  1   1   0
## 1173   1173    0 480 24   1    1  1   1   0
## 1174   1174    0 480 24   1    1  1   1   0
## 1175   1175    0 480 24   1    1  1   1   0
## 1176   1176    0 480 24   1    1  1   1   0
## 1177   1177    0 480 24   1    1  1   1   0
## 1178   1178    0 480 24   1    1  1   1   0
## 1179   1179    0 480 24   1    1  1   1   0
## 1180   1180    0 480 24   1    1  1   1   0
## 1181   1181    0 480 24   1    1  1   1   0
## 1182   1182    0 480 24   1    1  1   1   0
## 1183   1183    0 480 24   1    1  1   1   0
## 1184   1184    0 480 24   1    1  1   1   0
## 1185   1185    0 480 24   1    1  1   1   0
## 1186   1186    0 480 24   1    1  1   1   0
## 1187   1187    0 480 24   1    1  1   1   0
## 1188   1188    0 480 24   1    1  1   1   0
## 1189   1189    0 480 24   1    1  1   1   0
## 1190   1190    0 480 24   1    1  1   1   0
## 1191   1191    0 480 24   1    1  1   1   0
## 1192   1192    0 480 24   1    1  1   1   0
## 1193   1193    0 480 24   1    1  1   1   0
## 1194   1194    0 480 24   1    1  1   1   0
## 1195   1195    0 480 24   1    1  1   1   0
## 1196   1196    0 480 24   1    1  1   1   0
## 1197   1197    0 480 24   1    1  1   1   0
## 1198   1198    0 480 24   1    1  1   1   0
## 1199   1199    0 480 24   1    1  1   1   0
## 1200   1200    0 480 24   1    1  1   1   0
## 1201   1201    0 480 24   1    1  1   1   0
## 1202   1202    0 480 24   1    1  1   1   0
## 1203   1203    0 480 24   1    1  1   1   0
## 1204   1204    0 480 24   1    1  1   1   0
## 1205   1205    0 480 24   1    1  1   1   0
## 1206   1206    0 480 24   1    1  1   1   0
## 1207   1207    0 480 24   1    1  1   1   0
## 1208   1208    0 480 24   1    1  1   1   0
## 1209   1209    0 480 24   1    1  1   1   0
## 1210   1210    0 480 24   1    1  1   1   0
## 1211   1211    0 480 24   1    1  1   1   0
## 1212   1212    0 480 24   1    1  1   1   0
## 1213   1213    0 480 24   1    1  1   1   0
## 1214   1214    0 480 24   1    1  1   1   0
## 1215   1215    0 480 24   1    1  1   1   0
## 1216   1216    0 480 24   1    1  1   1   0
## 1217   1217    0 480 24   1    1  1   1   0
## 1218   1218    0 480 24   1    1  1   1   0
## 1219   1219    0 480 24   1    1  1   1   0
## 1220   1220    0 480 24   1    1  1   1   0
## 1221   1221    0 480 24   1    1  1   1   0
## 1222   1222    0 480 24   1    1  1   1   0
## 1223   1223    0 480 24   1    1  1   1   0
## 1224   1224    0 480 24   1    1  1   1   0
## 1225   1225    0 480 24   1    1  1   1   0
## 1226   1226    0 480 24   1    1  1   1   0
## 1227   1227    0 480 24   1    1  1   1   0
## 1228   1228    0 480 24   1    1  1   1   0
## 1229   1229    0 480 24   1    1  1   1   0
## 1230   1230    0 480 24   1    1  1   1   0
## 1231   1231    0 480 24   1    1  1   1   0
## 1232   1232    0 480 24   1    1  1   1   0
## 1233   1233    0 480 24   1    1  1   1   0
## 1234   1234    0 480 24   1    1  1   1   0
## 1235   1235    0 480 24   1    1  1   1   0
## 1236   1236    0 480 24   1    1  1   1   0
## 1237   1237    0 480 24   1    1  1   1   0
## 1238   1238    0 480 24   1    1  1   1   0
## 1239   1239    0 480 24   1    1  1   1   0
## 1240   1240    0 480 24   1    1  1   1   0
## 1241   1241    0 480 24   1    1  1   1   0
## 1242   1242    0 480 24   1    1  1   1   0
## 1243   1243    0 480 24   1    1  1   1   0
## 1244   1244    0 480 24   1    1  1   1   0
## 1245   1245    0 480 24   1    1  1   1   0
## 1246   1246    0 480 24   1    1  1   1   0
## 1247   1247    0 480 24   1    1  1   1   0
## 1248   1248    0 480 24   1    1  1   1   0
## 1249   1249    0 480 24   1    1  1   1   0
## 1250   1250    0 480 24   1    1  1   1   0
## 1251   1251    0 480 24   1    1  1   1   0
## 1252   1252    0 480 24   1    1  1   1   0
## 1253   1253    0 480 24   1    1  1   1   0
## 1254   1254    0 480 24   1    1  1   1   0
## 1255   1255    0 480 24   1    1  1   1   0
## 1256   1256    0 480 24   1    1  1   1   0
## 1257   1257    0 480 24   1    1  1   1   0
## 1258   1258    0 480 24   1    1  1   1   0
## 1259   1259    0 480 24   1    1  1   1   0
## 1260   1260    0 480 24   1    1  1   1   0
## 1261   1261    0 480 24   1    1  1   1   0
## 1262   1262    0 480 24   1    1  1   1   0
## 1263   1263    0 480 24   1    1  1   1   0
## 1264   1264    0 480 24   1    1  1   1   0
## 1265   1265    0 480 24   1    1  1   1   0
## 1266   1266    0 480 24   1    1  1   1   0
## 1267   1267    0 480 24   1    1  1   1   0
## 1268   1268    0 480 24   1    1  1   1   0
## 1269   1269    0 480 24   1    1  1   1   0
## 1270   1270    0 480 24   1    1  1   1   0
## 1271   1271    0 480 24   1    1  1   1   0
## 1272   1272    0 480 24   1    1  1   1   0
## 1273   1273    0 480 24   1    1  1   1   0
## 1274   1274    0 480 24   1    1  1   1   0
## 1275   1275    0 480 24   1    1  1   1   0
## 1276   1276    0 480 24   1    1  1   1   0
## 1277   1277    0 480 24   1    1  1   1   0
## 1278   1278    0 480 24   1    1  1   1   0
## 1279   1279    0 480 24   1    1  1   1   0
## 1280   1280    0 480 24   1    1  1   1   0
## 1281   1281    0 480 24   1    1  1   1   0
## 1282   1282    0 480 24   1    1  1   1   0
## 1283   1283    0 480 24   1    1  1   1   0
## 1284   1284    0 480 24   1    1  1   1   0
## 1285   1285    0 480 24   1    1  1   1   0
## 1286   1286    0 480 24   1    1  1   1   0
## 1287   1287    0 480 24   1    1  1   1   0
## 1288   1288    0 480 24   1    1  1   1   0
## 1289   1289    0 480 24   1    1  1   1   0
## 1290   1290    0 480 24   1    1  1   1   0
## 1291   1291    0 480 24   1    1  1   1   0
## 1292   1292    0 480 24   1    1  1   1   0
## 1293   1293    0 480 24   1    1  1   1   0
## 1294   1294    0 480 24   1    1  1   1   0
## 1295   1295    0 480 24   1    1  1   1   0
## 1296   1296    0 480 24   1    1  1   1   0
## 1297   1297    0 480 24   1    1  1   1   0
## 1298   1298    0 480 24   1    1  1   1   0
## 1299   1299    0 480 24   1    1  1   1   0
## 1300   1300    0 480 24   1    1  1   1   0
## 1301   1301    0 480 24   1    1  1   1   0
## 1302   1302    0 480 24   1    1  1   1   0
## 1303   1303    0 480 24   1    1  1   1   0
## 1304   1304    0 480 24   1    1  1   1   0
## 1305   1305    0 480 24   1    1  1   1   0
## 1306   1306    0 480 24   1    1  1   1   0
## 1307   1307    0 480 24   1    1  1   1   0
## 1308   1308    0 480 24   1    1  1   1   0
## 1309   1309    0 480 24   1    1  1   1   0
## 1310   1310    0 480 24   1    1  1   1   0
## 1311   1311    0 480 24   1    1  1   1   0
## 1312   1312    0 480 24   1    1  1   1   0
## 1313   1313    0 480 24   1    1  1   1   0
## 1314   1314    0 480 24   1    1  1   1   0
## 1315   1315    0 480 24   1    1  1   1   0
## 1316   1316    0 480 24   1    1  1   1   0
## 1317   1317    0 480 24   1    1  1   1   0
## 1318   1318    0 480 24   1    1  1   1   0
## 1319   1319    0 480 24   1    1  1   1   0
## 1320   1320    0 480 24   1    1  1   1   0
## 1321   1321    0 480 24   1    1  1   1   0
## 1322   1322    0 480 24   1    1  1   1   0
## 1323   1323    0 480 24   1    1  1   1   0
## 1324   1324    0 480 24   1    1  1   1   0
## 1325   1325    0 480 24   1    1  1   1   0
## 1326   1326    0 480 24   1    1  1   1   0
## 1327   1327    0 480 24   1    1  1   1   0
## 1328   1328    0 480 24   1    1  1   1   0
## 1329   1329    0 480 24   1    1  1   1   0
## 1330   1330    0 480 24   1    1  1   1   0
## 1331   1331    0 480 24   1    1  1   1   0
## 1332   1332    0 480 24   1    1  1   1   0
## 1333   1333    0 480 24   1    1  1   1   0
## 1334   1334    0 480 24   1    1  1   1   0
## 1335   1335    0 480 24   1    1  1   1   0
## 1336   1336    0 480 24   1    1  1   1   0
## 1337   1337    0 480 24   1    1  1   1   0
## 1338   1338    0 480 24   1    1  1   1   0
## 1339   1339    0 480 24   1    1  1   1   0
## 1340   1340    0 480 24   1    1  1   1   0
## 1341   1341    0 480 24   1    1  1   1   0
## 1342   1342    0 480 24   1    1  1   1   0
## 1343   1343    0 480 24   1    1  1   1   0
## 1344   1344    0 480 24   1    1  1   1   0
## 1345   1345    0 480 24   1    1  1   1   0
## 1346   1346    0 480 24   1    1  1   1   0
## 1347   1347    0 480 24   1    1  1   1   0
## 1348   1348    0 480 24   1    1  1   1   0
## 1349   1349    0 480 24   1    1  1   1   0
## 1350   1350    0 480 24   1    1  1   1   0
## 1351   1351    0 480 24   1    1  1   1   0
## 1352   1352    0 480 24   1    1  1   1   0
## 1353   1353    0 480 24   1    1  1   1   0
## 1354   1354    0 480 24   1    1  1   1   0
## 1355   1355    0 480 24   1    1  1   1   0
## 1356   1356    0 480 24   1    1  1   1   0
## 1357   1357    0 480 24   1    1  1   1   0
## 1358   1358    0 480 24   1    1  1   1   0
## 1359   1359    0 480 24   1    1  1   1   0
## 1360   1360    0 480 24   1    1  1   1   0
## 1361   1361    0 480 24   1    1  1   1   0
## 1362   1362    0 480 24   1    1  1   1   0
## 1363   1363    0 480 24   1    1  1   1   0
## 1364   1364    0 480 24   1    1  1   1   0
## 1365   1365    0 480 24   1    1  1   1   0
## 1366   1366    0 480 24   1    1  1   1   0
## 1367   1367    0 480 24   1    1  1   1   0
## 1368   1368    0 480 24   1    1  1   1   0
## 1369   1369    0 480 24   1    1  1   1   0
## 1370   1370    0 480 24   1    1  1   1   0
## 1371   1371    0 480 24   1    1  1   1   0
## 1372   1372    0 480 24   1    1  1   1   0
## 1373   1373    0 480 24   1    1  1   1   0
## 1374   1374    0 480 24   1    1  1   1   0
## 1375   1375    0 480 24   1    1  1   1   0
## 1376   1376    0 480 24   1    1  1   1   0
## 1377   1377    0 480 24   1    1  1   1   0
## 1378   1378    0 480 24   1    1  1   1   0
## 1379   1379    0 480 24   1    1  1   1   0
## 1380   1380    0 480 24   1    1  1   1   0
## 1381   1381    0 480 24   1    1  1   1   0
## 1382   1382    0 480 24   1    1  1   1   0
## 1383   1383    0 480 24   1    1  1   1   0
## 1384   1384    0 480 24   1    1  1   1   0
## 1385   1385    0 480 24   1    1  1   1   0
## 1386   1386    0 480 24   1    1  1   1   0
## 1387   1387    0 480 24   1    1  1   1   0
## 1388   1388    0 480 24   1    1  1   1   0
## 1389   1389    0 480 24   1    1  1   1   0
## 1390   1390    0 480 24   1    1  1   1   0
## 1391   1391    0 480 24   1    1  1   1   0
## 1392   1392    0 480 24   1    1  1   1   0
## 1393   1393    0 480 24   1    1  1   1   0
## 1394   1394    0 480 24   1    1  1   1   0
## 1395   1395    0 480 24   1    1  1   1   0
## 1396   1396    0 480 24   1    1  1   1   0
## 1397   1397    0 480 24   1    1  1   1   0
## 1398   1398    0 480 24   1    1  1   1   0
## 1399   1399    0 480 24   1    1  1   1   0
## 1400   1400    0 480 24   1    1  1   1   0
## 1401   1401    0 480 24   1    1  1   1   0
## 1402   1402    0 480 24   1    1  1   1   0
## 1403   1403    0 480 24   1    1  1   1   0
## 1404   1404    0 480 24   1    1  1   1   0
## 1405   1405    0 480 24   1    1  1   1   0
## 1406   1406    0 480 24   1    1  1   1   0
## 1407   1407    0 480 24   1    1  1   1   0
## 1408   1408    0 480 24   1    1  1   1   0
## 1409   1409    0 480 24   1    1  1   1   0
## 1410   1410    0 480 24   1    1  1   1   0
## 1411   1411    0 480 24   1    1  1   1   0
## 1412   1412    0 480 24   1    1  1   1   0
## 1413   1413    0 480 24   1    1  1   1   0
## 1414   1414    0 480 24   1    1  1   1   0
## 1415   1415    0 480 24   1    1  1   1   0
## 1416   1416    0 480 24   1    1  1   1   0
## 1417   1417    0 480 24   1    1  1   1   0
## 1418   1418    0 480 24   1    1  1   1   0
## 1419   1419    0 480 24   1    1  1   1   0
## 1420   1420    0 480 24   1    1  1   1   0
## 1421   1421    0 480 24   1    1  1   1   0
## 1422   1422    0 480 24   1    1  1   1   0
## 1423   1423    0 480 24   1    1  1   1   0
## 1424   1424    0 480 24   1    1  1   1   0
## 1425   1425    0 480 24   1    1  1   1   0
## 1426   1426    0 480 24   1    1  1   1   0
## 1427   1427    0 480 24   1    1  1   1   0
## 1428   1428    0 480 24   1    1  1   1   0
## 1429   1429    0 480 24   1    1  1   1   0
## 1430   1430    0 480 24   1    1  1   1   0
## 1431   1431    0 480 24   1    1  1   1   0
## 1432   1432    0 480 24   1    1  1   1   0
## 1433   1433    0 480 24   1    1  1   1   0
## 1434   1434    0 480 24   1    1  1   1   0
## 1435   1435    0 480 24   1    1  1   1   0
## 1436   1436    0 480 24   1    1  1   1   0
## 1437   1437    0 480 24   1    1  1   1   0
## 1438   1438    0 480 24   1    1  1   1   0
## 1439   1439    0 480 24   1    1  1   1   0
## 1440   1440    0 480 24   1    1  1   1   0
## 1441   1441    0 480 24   1    1  1   1   0
## 1442   1442    0 480 24   1    1  1   1   0
## 1443   1443    0 480 24   1    1  1   1   0
## 1444   1444    0 480 24   1    1  1   1   0
## 1445   1445    0 480 24   1    1  1   1   0
## 1446   1446    0 480 24   1    1  1   1   0
## 1447   1447    0 480 24   1    1  1   1   0
## 1448   1448    0 480 24   1    1  1   1   0
## 1449   1449    0 480 24   1    1  1   1   0
## 1450   1450    0 480 24   1    1  1   1   0
## 1451   1451    0 480 24   1    1  1   1   0
## 1452   1452    0 480 24   1    1  1   1   0
## 1453   1453    0 480 24   1    1  1   1   0
## 1454   1454    0 480 24   1    1  1   1   0
## 1455   1455    0 480 24   1    1  1   1   0
## 1456   1456    0 480 24   1    1  1   1   0
## 1457   1457    0 480 24   1    1  1   1   0
## 1458   1458    0 480 24   1    1  1   1   0
## 1459   1459    0 480 24   1    1  1   1   0
## 1460   1460    0 480 24   1    1  1   1   0
## 1461   1461    0 480 24   1    1  1   1   0
## 1462   1462    0 480 24   1    1  1   1   0
## 1463   1463    0 480 24   1    1  1   1   0
## 1464   1464    0 480 24   1    1  1   1   0
## 1465   1465    0 480 24   1    1  1   1   0
## 1466   1466    0 480 24   1    1  1   1   0
## 1467   1467    0 480 24   1    1  1   1   0
## 1468   1468    0 480 24   1    1  1   1   0
## 1469   1469    0 480 24   1    1  1   1   0
## 1470   1470    0 480 24   1    1  1   1   0
## 1471   1471    0 480 24   1    1  1   1   0
## 1472   1472    0 480 24   1    1  1   1   0
## 1473   1473    0 480 24   1    1  1   1   0
## 1474   1474    0 480 24   1    1  1   1   0
## 1475   1475    0 480 24   1    1  1   1   0
## 1476   1476    0 480 24   1    1  1   1   0
## 1477   1477    0 480 24   1    1  1   1   0
## 1478   1478    0 480 24   1    1  1   1   0
## 1479   1479    0 480 24   1    1  1   1   0
## 1480   1480    0 480 24   1    1  1   1   0
## 1481   1481    0 480 24   1    1  1   1   0
## 1482   1482    0 480 24   1    1  1   1   0
## 1483   1483    0 480 24   1    1  1   1   0
## 1484   1484    0 480 24   1    1  1   1   0
## 1485   1485    0 480 24   1    1  1   1   0
## 1486   1486    0 480 24   1    1  1   1   0
## 1487   1487    0 480 24   1    1  1   1   0
## 1488   1488    0 480 24   1    1  1   1   0
## 1489   1489    0 480 24   1    1  1   1   0
## 1490   1490    0 480 24   1    1  1   1   0
## 1491   1491    0 480 24   1    1  1   1   0
## 1492   1492    0 480 24   1    1  1   1   0
## 1493   1493    0 480 24   1    1  1   1   0
## 1494   1494    0 480 24   1    1  1   1   0
## 1495   1495    0 480 24   1    1  1   1   0
## 1496   1496    0 480 24   1    1  1   1   0
## 1497   1497    0 480 24   1    1  1   1   0
## 1498   1498    0 480 24   1    1  1   1   0
## 1499   1499    0 480 24   1    1  1   1   0
## 1500   1500    0 480 24   1    1  1   1   0
## 1501   1501    0 480 24   1    1  1   1   0
## 1502   1502    0 480 24   1    1  1   1   0
## 1503   1503    0 480 24   1    1  1   1   0
## 1504   1504    0 480 24   1    1  1   1   0
## 1505   1505    0 480 24   1    1  1   1   0
## 1506   1506    0 480 24   1    1  1   1   0
## 1507   1507    0 480 24   1    1  1   1   0
## 1508   1508    0 480 24   1    1  1   1   0
## 1509   1509    0 480 24   1    1  1   1   0
## 1510   1510    0 480 24   1    1  1   1   0
## 1511   1511    0 480 24   1    1  1   1   0
## 1512   1512    0 480 24   1    1  1   1   0
## 1513   1513    0 480 24   1    1  1   1   0
## 1514   1514    0 480 24   1    1  1   1   0
## 1515   1515    0 480 24   1    1  1   1   0
## 1516   1516    0 480 24   1    1  1   1   0
## 1517   1517    0 480 24   1    1  1   1   0
## 1518   1518    0 480 24   1    1  1   1   0
## 1519   1519    0 480 24   1    1  1   1   0
## 1520   1520    0 480 24   1    1  1   1   0
## 1521   1521    0 480 24   1    1  1   1   0
## 1522   1522    0 480 24   1    1  1   1   0
## 1523   1523    0 480 24   1    1  1   1   0
## 1524   1524    0 480 24   1    1  1   1   0
## 1525   1525    0 480 24   1    1  1   1   0
## 1526   1526    0 480 24   1    1  1   1   0
## 1527   1527    0 480 24   1    1  1   1   0
## 1528   1528    0 480 24   1    1  1   1   0
## 1529   1529    0 480 24   1    1  1   1   0
## 1530   1530    0 480 24   1    1  1   1   0
## 1531   1531    0 480 24   1    1  1   1   0
## 1532   1532    0 480 24   1    1  1   1   0
## 1533   1533    0 480 24   1    1  1   1   0
## 1534   1534    0 480 24   1    1  1   1   0
## 1535   1535    0 480 24   1    1  1   1   0
## 1536   1536    0 480 24   1    1  1   1   0
## 1537   1537    0 480 24   1    1  1   1   0
## 1538   1538    0 480 24   1    1  1   1   0
## 1539   1539    0 480 24   1    1  1   1   0
## 1540   1540    0 480 24   1    1  1   1   0
## 1541   1541    0 480 24   1    1  1   1   0
## 1542   1542    0 480 24   1    1  1   1   0
## 1543   1543    0 480 24   1    1  1   1   0
## 1544   1544    0 480 24   1    1  1   1   0
## 1545   1545    0 480 24   1    1  1   1   0
## 1546   1546    0 480 24   1    1  1   1   0
## 1547   1547    0 480 24   1    1  1   1   0
## 1548   1548    0 480 24   1    1  1   1   0
## 1549   1549    0 480 24   1    1  1   1   0
## 1550   1550    0 480 24   1    1  1   1   0
## 1551   1551    0 480 24   1    1  1   1   0
## 1552   1552    0 480 24   1    1  1   1   0
## 1553   1553    0 480 24   1    1  1   1   0
## 1554   1554    0 480 24   1    1  1   1   0
## 1555   1555    0 480 24   1    1  1   1   0
## 1556   1556    0 480 24   1    1  1   1   0
## 1557   1557    0 480 24   1    1  1   1   0
## 1558   1558    0 480 24   1    1  1   1   0
## 1559   1559    0 480 24   1    1  1   1   0
## 1560   1560    0 480 24   1    1  1   1   0
## 1561   1561    0 480 24   1    1  1   1   0
## 1562   1562    0 480 24   1    1  1   1   0
## 1563   1563    0 480 24   1    1  1   1   0
## 1564   1564    0 480 24   1    1  1   1   0
## 1565   1565    0 480 24   1    1  1   1   0
## 1566   1566    0 480 24   1    1  1   1   0
## 1567   1567    0 480 24   1    1  1   1   0
## 1568   1568    0 480 24   1    1  1   1   0
## 1569   1569    0 480 24   1    1  1   1   0
## 1570   1570    0 480 24   1    1  1   1   0
## 1571   1571    0 480 24   1    1  1   1   0
## 1572   1572    0 480 24   1    1  1   1   0
## 1573   1573    0 480 24   1    1  1   1   0
## 1574   1574    0 480 24   1    1  1   1   0
## 1575   1575    0 480 24   1    1  1   1   0
## 1576   1576    0 480 24   1    1  1   1   0
## 1577   1577    0 480 24   1    1  1   1   0
## 1578   1578    0 480 24   1    1  1   1   0
## 1579   1579    0 480 24   1    1  1   1   0
## 1580   1580    0 480 24   1    1  1   1   0
## 1581   1581    0 480 24   1    1  1   1   0
## 1582   1582    0 480 24   1    1  1   1   0
## 1583   1583    0 480 24   1    1  1   1   0
## 1584   1584    0 480 24   1    1  1   1   0
## 1585   1585    0 480 24   1    1  1   1   0
## 1586   1586    0 480 24   1    1  1   1   0
## 1587   1587    0 480 24   1    1  1   1   0
## 1588   1588    0 480 24   1    1  1   1   0
## 1589   1589    0 480 24   1    1  1   1   0
## 1590   1590    0 480 24   1    1  1   1   0
## 1591   1591    0 480 24   1    1  1   1   0
## 1592   1592    0 480 24   1    1  1   1   0
## 1593   1593    0 480 24   1    1  1   1   0
## 1594   1594    0 480 24   1    1  1   1   0
## 1595   1595    0 480 24   1    1  1   1   0
## 1596   1596    0 480 24   1    1  1   1   0
## 1597   1597    0 480 24   1    1  1   1   0
## 1598   1598    0 480 24   1    1  1   1   0
## 1599   1599    0 480 24   1    1  1   1   0
## 1600   1600    0 480 24   1    1  1   1   0
## 1601   1601    0 480 24   1    1  1   1   0
## 1602   1602    0 480 24   1    1  1   1   0
## 1603   1603    0 480 24   1    1  1   1   0
## 1604   1604    0 480 24   1    1  1   1   0
## 1605   1605    0 480 24   1    1  1   1   0
## 1606   1606    0 480 24   1    1  1   1   0
## 1607   1607    0 480 24   1    1  1   1   0
## 1608   1608    0 480 24   1    1  1   1   0
## 1609   1609    0 480 24   1    1  1   1   0
## 1610   1610    0 480 24   1    1  1   1   0
## 1611   1611    0 480 24   1    1  1   1   0
## 1612   1612    0 480 24   1    1  1   1   0
## 1613   1613    0 480 24   1    1  1   1   0
## 1614   1614    0 480 24   1    1  1   1   0
## 1615   1615    0 480 24   1    1  1   1   0
## 1616   1616    0 480 24   1    1  1   1   0
## 1617   1617    0 480 24   1    1  1   1   0
## 1618   1618    0 480 24   1    1  1   1   0
## 1619   1619    0 480 24   1    1  1   1   0
## 1620   1620    0 480 24   1    1  1   1   0
## 1621   1621    0 480 24   1    1  1   1   0
## 1622   1622    0 480 24   1    1  1   1   0
## 1623   1623    0 480 24   1    1  1   1   0
## 1624   1624    0 480 24   1    1  1   1   0
## 1625   1625    0 480 24   1    1  1   1   0
## 1626   1626    0 480 24   1    1  1   1   0
## 1627   1627    0 480 24   1    1  1   1   0
## 1628   1628    0 480 24   1    1  1   1   0
## 1629   1629    0 480 24   1    1  1   1   0
## 1630   1630    0 480 24   1    1  1   1   0
## 1631   1631    0 480 24   1    1  1   1   0
## 1632   1632    0 480 24   1    1  1   1   0
## 1633   1633    0 480 24   1    1  1   1   0
## 1634   1634    0 480 24   1    1  1   1   0
## 1635   1635    0 480 24   1    1  1   1   0
## 1636   1636    0 480 24   1    1  1   1   0
## 1637   1637    0 480 24   1    1  1   1   0
## 1638   1638    0 480 24   1    1  1   1   0
## 1639   1639    0 480 24   1    1  1   1   0
## 1640   1640    0 480 24   1    1  1   1   0
## 1641   1641    0 480 24   1    1  1   1   0
## 1642   1642    0 480 24   1    1  1   1   0
## 1643   1643    0 480 24   1    1  1   1   0
## 1644   1644    0 480 24   1    1  1   1   0
## 1645   1645    0 480 24   1    1  1   1   0
## 1646   1646    0 480 24   1    1  1   1   0
## 1647   1647    0 480 24   1    1  1   1   0
## 1648   1648    0 480 24   1    1  1   1   0
## 1649   1649    0 480 24   1    1  1   1   0
## 1650   1650    0 480 24   1    1  1   1   0
## 1651   1651    0 480 24   1    1  1   1   0
## 1652   1652    0 480 24   1    1  1   1   0
## 1653   1653    0 480 24   1    1  1   1   0
## 1654   1654    0 480 24   1    1  1   1   0
## 1655   1655    0 480 24   1    1  1   1   0
## 1656   1656    0 480 24   1    1  1   1   0
## 1657   1657    0 480 24   1    1  1   1   0
## 1658   1658    0 480 24   1    1  1   1   0
## 1659   1659    0 480 24   1    1  1   1   0
## 1660   1660    0 480 24   1    1  1   1   0
## 1661   1661    0 480 24   1    1  1   1   0
## 1662   1662    0 480 24   1    1  1   1   0
## 1663   1663    0 480 24   1    1  1   1   0
## 1664   1664    0 480 24   1    1  1   1   0
## 1665   1665    0 480 24   1    1  1   1   0
## 1666   1666    0 480 24   1    1  1   1   0
## 1667   1667    0 480 24   1    1  1   1   0
## 1668   1668    0 480 24   1    1  1   1   0
## 1669   1669    0 480 24   1    1  1   1   0
## 1670   1670    0 480 24   1    1  1   1   0
## 1671   1671    0 480 24   1    1  1   1   0
## 1672   1672    0 480 24   1    1  1   1   0
## 1673   1673    0 480 24   1    1  1   1   0
## 1674   1674    0 480 24   1    1  1   1   0
## 1675   1675    0 480 24   1    1  1   1   0
## 1676   1676    0 480 24   1    1  1   1   0
## 1677   1677    0 480 24   1    1  1   1   0
## 1678   1678    0 480 24   1    1  1   1   0
## 1679   1679    0 480 24   1    1  1   1   0
## 1680   1680    0 480 24   1    1  1   1   0
## 1681   1681    0 480 24   1    1  1   1   0
## 1682   1682    0 480 24   1    1  1   1   0
## 1683   1683    0 480 24   1    1  1   1   0
## 1684   1684    0 480 24   1    1  1   1   0
## 1685   1685    0 480 24   1    1  1   1   0
## 1686   1686    0 480 24   1    1  1   1   0
## 1687   1687    0 480 24   1    1  1   1   0
## 1688   1688    0 480 24   1    1  1   1   0
## 1689   1689    0 480 24   1    1  1   1   0
## 1690   1690    0 480 24   1    1  1   1   0
## 1691   1691    0 480 24   1    1  1   1   0
## 1692   1692    0 480 24   1    1  1   1   0
## 1693   1693    0 480 24   1    1  1   1   0
## 1694   1694    0 480 24   1    1  1   1   0
## 1695   1695    0 480 24   1    1  1   1   0
## 1696   1696    0 480 24   1    1  1   1   0
## 1697   1697    0 480 24   1    1  1   1   0
## 1698   1698    0 480 24   1    1  1   1   0
## 1699   1699    0 480 24   1    1  1   1   0
## 1700   1700    0 480 24   1    1  1   1   0
## 1701   1701    0 480 24   1    1  1   1   0
## 1702   1702    0 480 24   1    1  1   1   0
## 1703   1703    0 480 24   1    1  1   1   0
## 1704   1704    0 480 24   1    1  1   1   0
## 1705   1705    0 480 24   1    1  1   1   0
## 1706   1706    0 480 24   1    1  1   1   0
## 1707   1707    0 480 24   1    1  1   1   0
## 1708   1708    0 480 24   1    1  1   1   0
## 1709   1709    0 480 24   1    1  1   1   0
## 1710   1710    0 480 24   1    1  1   1   0
## 1711   1711    0 480 24   1    1  1   1   0
## 1712   1712    0 480 24   1    1  1   1   0
## 1713   1713    0 480 24   1    1  1   1   0
## 1714   1714    0 480 24   1    1  1   1   0
## 1715   1715    0 480 24   1    1  1   1   0
## 1716   1716    0 480 24   1    1  1   1   0
## 1717   1717    0 480 24   1    1  1   1   0
## 1718   1718    0 480 24   1    1  1   1   0
## 1719   1719    0 480 24   1    1  1   1   0
## 1720   1720    0 480 24   1    1  1   1   0
## 1721   1721    0 480 24   1    1  1   1   0
## 1722   1722    0 480 24   1    1  1   1   0
## 1723   1723    0 480 24   1    1  1   1   0
## 1724   1724    0 480 24   1    1  1   1   0
## 1725   1725    0 480 24   1    1  1   1   0
## 1726   1726    0 480 24   1    1  1   1   0
## 1727   1727    0 480 24   1    1  1   1   0
## 1728   1728    0 480 24   1    1  1   1   0
## 1729   1729    0 480 24   1    1  1   1   0
## 1730   1730    0 480 24   1    1  1   1   0
## 1731   1731    0 480 24   1    1  1   1   0
## 1732   1732    0 480 24   1    1  1   1   0
## 1733   1733    0 480 24   1    1  1   1   0
## 1734   1734    0 480 24   1    1  1   1   0
## 1735   1735    0 480 24   1    1  1   1   0
## 1736   1736    0 480 24   1    1  1   1   0
## 1737   1737    0 480 24   1    1  1   1   0
## 1738   1738    0 480 24   1    1  1   1   0
## 1739   1739    0 480 24   1    1  1   1   0
## 1740   1740    0 480 24   1    1  1   1   0
## 1741   1741    0 480 24   1    1  1   1   0
## 1742   1742    0 480 24   1    1  1   1   0
## 1743   1743    0 480 24   1    1  1   1   0
## 1744   1744    0 480 24   1    1  1   1   0
## 1745   1745    0 480 24   1    1  1   1   0
## 1746   1746    0 480 24   1    1  1   1   0
## 1747   1747    0 480 24   1    1  1   1   0
## 1748   1748    0 480 24   1    1  1   1   0
## 1749   1749    0 480 24   1    1  1   1   0
## 1750   1750    0 480 24   1    1  1   1   0
## 1751   1751    0 480 24   1    1  1   1   0
## 1752   1752    0 480 24   1    1  1   1   0
## 1753   1753    0 480 24   1    1  1   1   0
## 1754   1754    0 480 24   1    1  1   1   0
## 1755   1755    0 480 24   1    1  1   1   0
## 1756   1756    0 480 24   1    1  1   1   0
## 1757   1757    0 480 24   1    1  1   1   0
## 1758   1758    0 480 24   1    1  1   1   0
## 1759   1759    0 480 24   1    1  1   1   0
## 1760   1760    0 480 24   1    1  1   1   0
## 1761   1761    0 480 24   1    1  1   1   0
## 1762   1762    0 480 24   1    1  1   1   0
## 1763   1763    0 480 24   1    1  1   1   0
## 1764   1764    0 480 24   1    1  1   1   0
## 1765   1765    0 480 24   1    1  1   1   0
## 1766   1766    0 480 24   1    1  1   1   0
## 1767   1767    0 480 24   1    1  1   1   0
## 1768   1768    0 480 24   1    1  1   1   0
## 1769   1769    0 480 24   1    1  1   1   0
## 1770   1770    0 480 24   1    1  1   1   0
## 1771   1771    0 480 24   1    1  1   1   0
## 1772   1772    0 480 24   1    1  1   1   0
## 1773   1773    0 480 24   1    1  1   1   0
## 1774   1774    0 480 24   1    1  1   1   0
## 1775   1775    0 480 24   1    1  1   1   0
## 1776   1776    0 480 24   1    1  1   1   0
## 1777   1777    0 480 24   1    1  1   1   0
## 1778   1778    0 480 24   1    1  1   1   0
## 1779   1779    0 480 24   1    1  1   1   0
## 1780   1780    0 480 24   1    1  1   1   0
## 1781   1781    0 480 24   1    1  1   1   0
## 1782   1782    0 480 24   1    1  1   1   0
## 1783   1783    0 480 24   1    1  1   1   0
## 1784   1784    0 480 24   1    1  1   1   0
## 1785   1785    0 480 24   1    1  1   1   0
## 1786   1786    0 480 24   1    1  1   1   0
## 1787   1787    0 480 24   1    1  1   1   0
## 1788   1788    0 480 24   1    1  1   1   0
## 1789   1789    0 480 24   1    1  1   1   0
## 1790   1790    0 480 24   1    1  1   1   0
## 1791   1791    0 480 24   1    1  1   1   0
## 1792   1792    0 480 24   1    1  1   1   0
## 1793   1793    0 480 24   1    1  1   1   0
## 1794   1794    0 480 24   1    1  1   1   0
## 1795   1795    0 480 24   1    1  1   1   0
## 1796   1796    0 480 24   1    1  1   1   0
## 1797   1797    0 480 24   1    1  1   1   0
## 1798   1798    0 480 24   1    1  1   1   0
## 1799   1799    0 480 24   1    1  1   1   0
## 1800   1800    0 480 24   1    1  1   1   0
## 1801   1801    0 480 24   1    1  1   1   0
## 1802   1802    0 480 24   1    1  1   1   0
## 1803   1803    0 480 24   1    1  1   1   0
## 1804   1804    0 480 24   1    1  1   1   0
## 1805   1805    0 480 24   1    1  1   1   0
## 1806   1806    0 480 24   1    1  1   1   0
## 1807   1807    0 480 24   1    1  1   1   0
## 1808   1808    0 480 24   1    1  1   1   0
## 1809   1809    0 480 24   1    1  1   1   0
## 1810   1810    0 480 24   1    1  1   1   0
## 1811   1811    0 480 24   1    1  1   1   0
## 1812   1812    0 480 24   1    1  1   1   0
## 1813   1813    0 480 24   1    1  1   1   0
## 1814   1814    0 480 24   1    1  1   1   0
## 1815   1815    0 480 24   1    1  1   1   0
## 1816   1816    0 480 24   1    1  1   1   0
## 1817   1817    0 480 24   1    1  1   1   0
## 1818   1818    0 480 24   1    1  1   1   0
## 1819   1819    0 480 24   1    1  1   1   0
## 1820   1820    0 480 24   1    1  1   1   0
## 1821   1821    0 480 24   1    1  1   1   0
## 1822   1822    0 480 24   1    1  1   1   0
## 1823   1823    0 480 24   1    1  1   1   0
## 1824   1824    0 480 24   1    1  1   1   0
## 1825   1825    0 480 24   1    1  1   1   0
## 1826   1826    0 480 24   1    1  1   1   0
## 1827   1827    0 480 24   1    1  1   1   0
## 1828   1828    0 480 24   1    1  1   1   0
## 1829   1829    0 480 24   1    1  1   1   0
## 1830   1830    0 480 24   1    1  1   1   0
## 1831   1831    0 480 24   1    1  1   1   0
## 1832   1832    0 480 24   1    1  1   1   0
## 1833   1833    0 480 24   1    1  1   1   0
## 1834   1834    0 480 24   1    1  1   1   0
## 1835   1835    0 480 24   1    1  1   1   0
## 1836   1836    0 480 24   1    1  1   1   0
## 1837   1837    0 480 24   1    1  1   1   0
## 1838   1838    0 480 24   1    1  1   1   0
## 1839   1839    0 480 24   1    1  1   1   0
## 1840   1840    0 480 24   1    1  1   1   0
## 1841   1841    0 480 24   1    1  1   1   0
## 1842   1842    0 480 24   1    1  1   1   0
## 1843   1843    0 480 24   1    1  1   1   0
## 1844   1844    0 480 24   1    1  1   1   0
## 1845   1845    0 480 24   1    1  1   1   0
## 1846   1846    0 480 24   1    1  1   1   0
## 1847   1847    0 480 24   1    1  1   1   0
## 1848   1848    0 480 24   1    1  1   1   0
## 1849   1849    0 480 24   1    1  1   1   0
## 1850   1850    0 480 24   1    1  1   1   0
## 1851   1851    0 480 24   1    1  1   1   0
## 1852   1852    0 480 24   1    1  1   1   0
## 1853   1853    0 480 24   1    1  1   1   0
## 1854   1854    0 480 24   1    1  1   1   0
## 1855   1855    0 480 24   1    1  1   1   0
## 1856   1856    0 480 24   1    1  1   1   0
## 1857   1857    0 480 24   1    1  1   1   0
## 1858   1858    0 480 24   1    1  1   1   0
## 1859   1859    0 480 24   1    1  1   1   0
## 1860   1860    0 480 24   1    1  1   1   0
## 1861   1861    0 480 24   1    1  1   1   0
## 1862   1862    0 480 24   1    1  1   1   0
## 1863   1863    0 480 24   1    1  1   1   0
## 1864   1864    0 480 24   1    1  1   1   0
## 1865   1865    0 480 24   1    1  1   1   0
## 1866   1866    0 480 24   1    1  1   1   0
## 1867   1867    0 480 24   1    1  1   1   0
## 1868   1868    0 480 24   1    1  1   1   0
## 1869   1869    0 480 24   1    1  1   1   0
## 1870   1870    0 480 24   1    1  1   1   0
## 1871   1871    0 480 24   1    1  1   1   0
## 1872   1872    0 480 24   1    1  1   1   0
## 1873   1873    0 480 24   1    1  1   1   0
## 1874   1874    0 480 24   1    1  1   1   0
## 1875   1875    0 480 24   1    1  1   1   0
## 1876   1876    0 480 24   1    1  1   1   0
## 1877   1877    0 480 24   1    1  1   1   0
## 1878   1878    0 480 24   1    1  1   1   0
## 1879   1879    0 480 24   1    1  1   1   0
## 1880   1880    0 480 24   1    1  1   1   0
## 1881   1881    0 480 24   1    1  1   1   0
## 1882   1882    0 480 24   1    1  1   1   0
## 1883   1883    0 480 24   1    1  1   1   0
## 1884   1884    0 480 24   1    1  1   1   0
## 1885   1885    0 480 24   1    1  1   1   0
## 1886   1886    0 480 24   1    1  1   1   0
## 1887   1887    0 480 24   1    1  1   1   0
## 1888   1888    0 480 24   1    1  1   1   0
## 1889   1889    0 480 24   1    1  1   1   0
## 1890   1890    0 480 24   1    1  1   1   0
## 1891   1891    0 480 24   1    1  1   1   0
## 1892   1892    0 480 24   1    1  1   1   0
## 1893   1893    0 480 24   1    1  1   1   0
## 1894   1894    0 480 24   1    1  1   1   0
## 1895   1895    0 480 24   1    1  1   1   0
## 1896   1896    0 480 24   1    1  1   1   0
## 1897   1897    0 480 24   1    1  1   1   0
## 1898   1898    0 480 24   1    1  1   1   0
## 1899   1899    0 480 24   1    1  1   1   0
## 1900   1900    0 480 24   1    1  1   1   0
## 1901   1901    0 480 24   1    1  1   1   0
## 1902   1902    0 480 24   1    1  1   1   0
## 1903   1903    0 480 24   1    1  1   1   0
## 1904   1904    0 480 24   1    1  1   1   0
## 1905   1905    0 480 24   1    1  1   1   0
## 1906   1906    0 480 24   1    1  1   1   0
## 1907   1907    0 480 24   1    1  1   1   0
## 1908   1908    0 480 24   1    1  1   1   0
## 1909   1909    0 480 24   1    1  1   1   0
## 1910   1910    0 480 24   1    1  1   1   0
## 1911   1911    0 480 24   1    1  1   1   0
## 1912   1912    0 480 24   1    1  1   1   0
## 1913   1913    0 480 24   1    1  1   1   0
## 1914   1914    0 480 24   1    1  1   1   0
## 1915   1915    0 480 24   1    1  1   1   0
## 1916   1916    0 480 24   1    1  1   1   0
## 1917   1917    0 480 24   1    1  1   1   0
## 1918   1918    0 480 24   1    1  1   1   0
## 1919   1919    0 480 24   1    1  1   1   0
## 1920   1920    0 480 24   1    1  1   1   0
## 1921   1921    0 480 24   1    1  1   1   0
## 1922   1922    0 480 24   1    1  1   1   0
## 1923   1923    0 480 24   1    1  1   1   0
## 1924   1924    0 480 24   1    1  1   1   0
## 1925   1925    0 480 24   1    1  1   1   0
## 1926   1926    0 480 24   1    1  1   1   0
## 1927   1927    0 480 24   1    1  1   1   0
## 1928   1928    0 480 24   1    1  1   1   0
## 1929   1929    0 480 24   1    1  1   1   0
## 1930   1930    0 480 24   1    1  1   1   0
## 1931   1931    0 480 24   1    1  1   1   0
## 1932   1932    0 480 24   1    1  1   1   0
## 1933   1933    0 480 24   1    1  1   1   0
## 1934   1934    0 480 24   1    1  1   1   0
## 1935   1935    0 480 24   1    1  1   1   0
## 1936   1936    0 480 24   1    1  1   1   0
## 1937   1937    0 480 24   1    1  1   1   0
## 1938   1938    0 480 24   1    1  1   1   0
## 1939   1939    0 480 24   1    1  1   1   0
## 1940   1940    0 480 24   1    1  1   1   0
## 1941   1941    0 480 24   1    1  1   1   0
## 1942   1942    0 480 24   1    1  1   1   0
## 1943   1943    0 480 24   1    1  1   1   0
## 1944   1944    0 480 24   1    1  1   1   0
## 1945   1945    0 480 24   1    1  1   1   0
## 1946   1946    0 480 24   1    1  1   1   0
## 1947   1947    0 480 24   1    1  1   1   0
## 1948   1948    0 480 24   1    1  1   1   0
## 1949   1949    0 480 24   1    1  1   1   0
## 1950   1950    0 480 24   1    1  1   1   0
## 1951   1951    0 480 24   1    1  1   1   0
## 1952   1952    0 480 24   1    1  1   1   0
## 1953   1953    0 480 24   1    1  1   1   0
## 1954   1954    0 480 24   1    1  1   1   0
## 1955   1955    0 480 24   1    1  1   1   0
## 1956   1956    0 480 24   1    1  1   1   0
## 1957   1957    0 480 24   1    1  1   1   0
## 1958   1958    0 480 24   1    1  1   1   0
## 1959   1959    0 480 24   1    1  1   1   0
## 1960   1960    0 480 24   1    1  1   1   0
## 1961   1961    0 480 24   1    1  1   1   0
## 1962   1962    0 480 24   1    1  1   1   0
## 1963   1963    0 480 24   1    1  1   1   0
## 1964   1964    0 480 24   1    1  1   1   0
## 1965   1965    0 480 24   1    1  1   1   0
## 1966   1966    0 480 24   1    1  1   1   0
## 1967   1967    0 480 24   1    1  1   1   0
## 1968   1968    0 480 24   1    1  1   1   0
## 1969   1969    0 480 24   1    1  1   1   0
## 1970   1970    0 480 24   1    1  1   1   0
## 1971   1971    0 480 24   1    1  1   1   0
## 1972   1972    0 480 24   1    1  1   1   0
## 1973   1973    0 480 24   1    1  1   1   0
## 1974   1974    0 480 24   1    1  1   1   0
## 1975   1975    0 480 24   1    1  1   1   0
## 1976   1976    0 480 24   1    1  1   1   0
## 1977   1977    0 480 24   1    1  1   1   0
## 1978   1978    0 480 24   1    1  1   1   0
## 1979   1979    0 480 24   1    1  1   1   0
## 1980   1980    0 480 24   1    1  1   1   0
## 1981   1981    0 480 24   1    1  1   1   0
## 1982   1982    0 480 24   1    1  1   1   0
## 1983   1983    0 480 24   1    1  1   1   0
## 1984   1984    0 480 24   1    1  1   1   0
## 1985   1985    0 480 24   1    1  1   1   0
## 1986   1986    0 480 24   1    1  1   1   0
## 1987   1987    0 480 24   1    1  1   1   0
## 1988   1988    0 480 24   1    1  1   1   0
## 1989   1989    0 480 24   1    1  1   1   0
## 1990   1990    0 480 24   1    1  1   1   0
## 1991   1991    0 480 24   1    1  1   1   0
## 1992   1992    0 480 24   1    1  1   1   0
## 1993   1993    0 480 24   1    1  1   1   0
## 1994   1994    0 480 24   1    1  1   1   0
## 1995   1995    0 480 24   1    1  1   1   0
## 1996   1996    0 480 24   1    1  1   1   0
## 1997   1997    0 480 24   1    1  1   1   0
## 1998   1998    0 480 24   1    1  1   1   0
## 1999   1999    0 480 24   1    1  1   1   0
## 2000   2000    0 480 24   1    1  1   1   0
## 2001   2001    0 480 24   1    1  1   1   0
## 2002   2002    0 480 24   1    1  1   1   0
## 2003   2003    0 480 24   1    1  1   1   0
## 2004   2004    0 480 24   1    1  1   1   0
## 2005   2005    0 480 24   1    1  1   1   0
## 2006   2006    0 480 24   1    1  1   1   0
## 2007   2007    0 480 24   1    1  1   1   0
## 2008   2008    0 480 24   1    1  1   1   0
## 2009   2009    0 480 24   1    1  1   1   0
## 2010   2010    0 480 24   1    1  1   1   0
## 2011   2011    0 480 24   1    1  1   1   0
## 2012   2012    0 480 24   1    1  1   1   0
## 2013   2013    0 480 24   1    1  1   1   0
## 2014   2014    0 480 24   1    1  1   1   0
## 2015   2015    0 480 24   1    1  1   1   0
## 2016   2016    0 480 24   1    1  1   1   0
## 2017   2017    0 480 24   1    1  1   1   0
## 2018   2018    0 480 24   1    1  1   1   0
## 2019   2019    0 480 24   1    1  1   1   0
## 2020   2020    0 480 24   1    1  1   1   0
## 2021   2021    0 480 24   1    1  1   1   0
## 2022   2022    0 480 24   1    1  1   1   0
## 2023   2023    0 480 24   1    1  1   1   0
## 2024   2024    0 480 24   1    1  1   1   0
## 2025   2025    0 480 24   1    1  1   1   0
## 2026   2026    0 480 24   1    1  1   1   0
## 2027   2027    0 480 24   1    1  1   1   0
## 2028   2028    0 480 24   1    1  1   1   0
## 2029   2029    0 480 24   1    1  1   1   0
## 2030   2030    0 480 24   1    1  1   1   0
## 2031   2031    0 480 24   1    1  1   1   0
## 2032   2032    0 480 24   1    1  1   1   0
## 2033   2033    0 480 24   1    1  1   1   0
## 2034   2034    0 480 24   1    1  1   1   0
## 2035   2035    0 480 24   1    1  1   1   0
## 2036   2036    0 480 24   1    1  1   1   0
## 2037   2037    0 480 24   1    1  1   1   0
## 2038   2038    0 480 24   1    1  1   1   0
## 2039   2039    0 480 24   1    1  1   1   0
## 2040   2040    0 480 24   1    1  1   1   0
## 2041   2041    0 480 24   1    1  1   1   0
## 2042   2042    0 480 24   1    1  1   1   0
## 2043   2043    0 480 24   1    1  1   1   0
## 2044   2044    0 480 24   1    1  1   1   0
## 2045   2045    0 480 24   1    1  1   1   0
## 2046   2046    0 480 24   1    1  1   1   0
## 2047   2047    0 480 24   1    1  1   1   0
## 2048   2048    0 480 24   1    1  1   1   0
## 2049   2049    0 480 24   1    1  1   1   0
## 2050   2050    0 480 24   1    1  1   1   0
## 2051   2051    0 480 24   1    1  1   1   0
## 2052   2052    0 480 24   1    1  1   1   0
## 2053   2053    0 480 24   1    1  1   1   0
## 2054   2054    0 480 24   1    1  1   1   0
## 2055   2055    0 480 24   1    1  1   1   0
## 2056   2056    0 480 24   1    1  1   1   0
## 2057   2057    0 480 24   1    1  1   1   0
## 2058   2058    0 480 24   1    1  1   1   0
## 2059   2059    0 480 24   1    1  1   1   0
## 2060   2060    0 480 24   1    1  1   1   0
## 2061   2061    0 480 24   1    1  1   1   0
## 2062   2062    0 480 24   1    1  1   1   0
## 2063   2063    0 480 24   1    1  1   1   0
## 2064   2064    0 480 24   1    1  1   1   0
## 2065   2065    0 480 24   1    1  1   1   0
## 2066   2066    0 480 24   1    1  1   1   0
## 2067   2067    0 480 24   1    1  1   1   0
## 2068   2068    0 480 24   1    1  1   1   0
## 2069   2069    0 480 24   1    1  1   1   0
## 2070   2070    0 480 24   1    1  1   1   0
## 2071   2071    0 480 24   1    1  1   1   0
## 2072   2072    0 480 24   1    1  1   1   0
## 2073   2073    0 480 24   1    1  1   1   0
## 2074   2074    0 480 24   1    1  1   1   0
## 2075   2075    0 480 24   1    1  1   1   0
## 2076   2076    0 480 24   1    1  1   1   0
## 2077   2077    0 480 24   1    1  1   1   0
## 2078   2078    0 480 24   1    1  1   1   0
## 2079   2079    0 480 24   1    1  1   1   0
## 2080   2080    0 480 24   1    1  1   1   0
## 2081   2081    0 480 24   1    1  1   1   0
## 2082   2082    0 480 24   1    1  1   1   0
## 2083   2083    0 480 24   1    1  1   1   0
## 2084   2084    0 480 24   1    1  1   1   0
## 2085   2085    0 480 24   1    1  1   1   0
## 2086   2086    0 480 24   1    1  1   1   0
## 2087   2087    0 480 24   1    1  1   1   0
## 2088   2088    0 480 24   1    1  1   1   0
## 2089   2089    0 480 24   1    1  1   1   0
## 2090   2090    0 480 24   1    1  1   1   0
## 2091   2091    0 480 24   1    1  1   1   0
## 2092   2092    0 480 24   1    1  1   1   0
## 2093   2093    0 480 24   1    1  1   1   0
## 2094   2094    0 480 24   1    1  1   1   0
## 2095   2095    0 480 24   1    1  1   1   0
## 2096   2096    0 480 24   1    1  1   1   0
## 2097   2097    0 480 24   1    1  1   1   0
## 2098   2098    0 480 24   1    1  1   1   0
## 2099   2099    0 480 24   1    1  1   1   0
## 2100   2100    0 480 24   1    1  1   1   0
## 2101   2101    0 480 24   1    1  1   1   0
## 2102   2102    0 480 24   1    1  1   1   0
## 2103   2103    0 480 24   1    1  1   1   0
## 2104   2104    0 480 24   1    1  1   1   0
## 2105   2105    0 480 24   1    1  1   1   0
## 2106   2106    0 480 24   1    1  1   1   0
## 2107   2107    0 480 24   1    1  1   1   0
## 2108   2108    0 480 24   1    1  1   1   0
## 2109   2109    0 480 24   1    1  1   1   0
## 2110   2110    0 480 24   1    1  1   1   0
## 2111   2111    0 480 24   1    1  1   1   0
## 2112   2112    0 480 24   1    1  1   1   0
## 2113   2113    0 480 24   1    1  1   1   0
## 2114   2114    0 480 24   1    1  1   1   0
## 2115   2115    0 480 24   1    1  1   1   0
## 2116   2116    0 480 24   1    1  1   1   0
## 2117   2117    0 480 24   1    1  1   1   0
## 2118   2118    0 480 24   1    1  1   1   0
## 2119   2119    0 480 24   1    1  1   1   0
## 2120   2120    0 480 24   1    1  1   1   0
## 2121   2121    0 480 24   1    1  1   1   0
## 2122   2122    0 480 24   1    1  1   1   0
## 2123   2123    0 480 24   1    1  1   1   0
## 2124   2124    0 480 24   1    1  1   1   0
## 2125   2125    0 480 24   1    1  1   1   0
## 2126   2126    0 480 24   1    1  1   1   0
## 2127   2127    0 480 24   1    1  1   1   0
## 2128   2128    0 480 24   1    1  1   1   0
## 2129   2129    0 480 24   1    1  1   1   0
## 2130   2130    0 480 24   1    1  1   1   0
## 2131   2131    0 480 24   1    1  1   1   0
## 2132   2132    0 480 24   1    1  1   1   0
## 2133   2133    0 480 24   1    1  1   1   0
## 2134   2134    0 480 24   1    1  1   1   0
## 2135   2135    0 480 24   1    1  1   1   0
## 2136   2136    0 480 24   1    1  1   1   0
## 2137   2137    0 480 24   1    1  1   1   0
## 2138   2138    0 480 24   1    1  1   1   0
## 2139   2139    0 480 24   1    1  1   1   0
## 2140   2140    0 480 24   1    1  1   1   0
## 2141   2141    0 480 24   1    1  1   1   0
## 2142   2142    0 480 24   1    1  1   1   0
## 2143   2143    0 480 24   1    1  1   1   0
## 2144   2144    0 480 24   1    1  1   1   0
## 2145   2145    0 480 24   1    1  1   1   0
## 2146   2146    0 480 24   1    1  1   1   0
## 2147   2147    0 480 24   1    1  1   1   0
## 2148   2148    0 480 24   1    1  1   1   0
## 2149   2149    0 480 24   1    1  1   1   0
## 2150   2150    0 480 24   1    1  1   1   0
## 2151   2151    0 480 24   1    1  1   1   0
## 2152   2152    0 480 24   1    1  1   1   0
## 2153   2153    0 480 24   1    1  1   1   0
## 2154   2154    0 480 24   1    1  1   1   0
## 2155   2155    0 480 24   1    1  1   1   0
## 2156   2156    0 480 24   1    1  1   1   0
## 2157   2157    0 480 24   1    1  1   1   0
## 2158   2158    0 480 24   1    1  1   1   0
## 2159   2159    0 480 24   1    1  1   1   0
## 2160   2160    0 480 24   1    1  1   1   0
## 2161   2161    0 480 24   1    1  1   1   0
## 2162   2162    0 480 24   1    1  1   1   0
## 2163   2163    0 480 24   1    1  1   1   0
## 2164   2164    0 480 24   1    1  1   1   0
## 2165   2165    0 480 24   1    1  1   1   0
## 2166   2166    0 480 24   1    1  1   1   0
## 2167   2167    0 480 24   1    1  1   1   0
## 2168   2168    0 480 24   1    1  1   1   0
## 2169   2169    0 480 24   1    1  1   1   0
## 2170   2170    0 480 24   1    1  1   1   0
## 2171   2171    0 480 24   1    1  1   1   0
## 2172   2172    0 480 24   1    1  1   1   0
## 2173   2173    0 480 24   1    1  1   1   0
## 2174   2174    0 480 24   1    1  1   1   0
## 2175   2175    0 480 24   1    1  1   1   0
## 2176   2176    0 480 24   1    1  1   1   0
## 2177   2177    0 480 24   1    1  1   1   0
## 2178   2178    0 480 24   1    1  1   1   0
## 2179   2179    0 480 24   1    1  1   1   0
## 2180   2180    0 480 24   1    1  1   1   0
## 2181   2181    0 480 24   1    1  1   1   0
## 2182   2182    0 480 24   1    1  1   1   0
## 2183   2183    0 480 24   1    1  1   1   0
## 2184   2184    0 480 24   1    1  1   1   0
## 2185   2185    0 480 24   1    1  1   1   0
## 2186   2186    0 480 24   1    1  1   1   0
## 2187   2187    0 480 24   1    1  1   1   0
## 2188   2188    0 480 24   1    1  1   1   0
## 2189   2189    0 480 24   1    1  1   1   0
## 2190   2190    0 480 24   1    1  1   1   0
## 2191   2191    0 480 24   1    1  1   1   0
## 2192   2192    0 480 24   1    1  1   1   0
## 2193   2193    0 480 24   1    1  1   1   0
## 2194   2194    0 480 24   1    1  1   1   0
## 2195   2195    0 480 24   1    1  1   1   0
## 2196   2196    0 480 24   1    1  1   1   0
## 2197   2197    0 480 24   1    1  1   1   0
## 2198   2198    0 480 24   1    1  1   1   0
## 2199   2199    0 480 24   1    1  1   1   0
## 2200   2200    0 480 24   1    1  1   1   0
## 2201   2201    0 480 24   1    1  1   1   0
## 2202   2202    0 480 24   1    1  1   1   0
## 2203   2203    0 480 24   1    1  1   1   0
## 2204   2204    0 480 24   1    1  1   1   0
## 2205   2205    0 480 24   1    1  1   1   0
## 2206   2206    0 480 24   1    1  1   1   0
## 2207   2207    0 480 24   1    1  1   1   0
## 2208   2208    0 480 24   1    1  1   1   0
## 2209   2209    0 480 24   1    1  1   1   0
## 2210   2210    0 480 24   1    1  1   1   0
## 2211   2211    0 480 24   1    1  1   1   0
## 2212   2212    0 480 24   1    1  1   1   0
## 2213   2213    0 480 24   1    1  1   1   0
## 2214   2214    0 480 24   1    1  1   1   0
## 2215   2215    0 480 24   1    1  1   1   0
## 2216   2216    0 480 24   1    1  1   1   0
## 2217   2217    0 480 24   1    1  1   1   0
## 2218   2218    0 480 24   1    1  1   1   0
## 2219   2219    0 480 24   1    1  1   1   0
## 2220   2220    0 480 24   1    1  1   1   0
## 2221   2221    0 480 24   1    1  1   1   0
## 2222   2222    0 480 24   1    1  1   1   0
## 2223   2223    0 480 24   1    1  1   1   0
## 2224   2224    0 480 24   1    1  1   1   0
## 2225   2225    0 480 24   1    1  1   1   0
## 2226   2226    0 480 24   1    1  1   1   0
## 2227   2227    0 480 24   1    1  1   1   0
## 2228   2228    0 480 24   1    1  1   1   0
## 2229   2229    0 480 24   1    1  1   1   0
## 2230   2230    0 480 24   1    1  1   1   0
## 2231   2231    0 480 24   1    1  1   1   0
## 2232   2232    0 480 24   1    1  1   1   0
## 2233   2233    0 480 24   1    1  1   1   0
## 2234   2234    0 480 24   1    1  1   1   0
## 2235   2235    0 480 24   1    1  1   1   0
## 2236   2236    0 480 24   1    1  1   1   0
## 2237   2237    0 480 24   1    1  1   1   0
## 2238   2238    0 480 24   1    1  1   1   0
## 2239   2239    0 480 24   1    1  1   1   0
## 2240   2240    0 480 24   1    1  1   1   0
## 2241   2241    0 480 24   1    1  1   1   0
## 2242   2242    0 480 24   1    1  1   1   0
## 2243   2243    0 480 24   1    1  1   1   0
## 2244   2244    0 480 24   1    1  1   1   0
## 2245   2245    0 480 24   1    1  1   1   0
## 2246   2246    0 480 24   1    1  1   1   0
## 2247   2247    0 480 24   1    1  1   1   0
## 2248   2248    0 480 24   1    1  1   1   0
## 2249   2249    0 480 24   1    1  1   1   0
## 2250   2250    0 480 24   1    1  1   1   0
## 2251   2251    0 480 24   1    1  1   1   0
## 2252   2252    0 480 24   1    1  1   1   0
## 2253   2253    0 480 24   1    1  1   1   0
## 2254   2254    0 480 24   1    1  1   1   0
## 2255   2255    0 480 24   1    1  1   1   0
## 2256   2256    0 480 24   1    1  1   1   0
## 2257   2257    0 480 24   1    1  1   1   0
## 2258   2258    0 480 24   1    1  1   1   0
## 2259   2259    0 480 24   1    1  1   1   0
## 2260   2260    0 480 24   1    1  1   1   0
## 2261   2261    0 480 24   1    1  1   1   0
## 2262   2262    0 480 24   1    1  1   1   0
## 2263   2263    0 480 24   1    1  1   1   0
## 2264   2264    0 480 24   1    1  1   1   0
## 2265   2265    0 480 24   1    1  1   1   0
## 2266   2266    0 480 24   1    1  1   1   0
## 2267   2267    0 480 24   1    1  1   1   0
## 2268   2268    0 480 24   1    1  1   1   0
## 2269   2269    0 480 24   1    1  1   1   0
## 2270   2270    0 480 24   1    1  1   1   0
## 2271   2271    0 480 24   1    1  1   1   0
## 2272   2272    0 480 24   1    1  1   1   0
## 2273   2273    0 480 24   1    1  1   1   0
## 2274   2274    0 480 24   1    1  1   1   0
## 2275   2275    0 480 24   1    1  1   1   0
## 2276   2276    0 480 24   1    1  1   1   0
## 2277   2277    0 480 24   1    1  1   1   0
## 2278   2278    0 480 24   1    1  1   1   0
## 2279   2279    0 480 24   1    1  1   1   0
## 2280   2280    0 480 24   1    1  1   1   0
## 2281   2281    0 480 24   1    1  1   1   0
## 2282   2282    0 480 24   1    1  1   1   0
## 2283   2283    0 480 24   1    1  1   1   0
## 2284   2284    0 480 24   1    1  1   1   0
## 2285   2285    0 480 24   1    1  1   1   0
## 2286   2286    0 480 24   1    1  1   1   0
## 2287   2287    0 480 24   1    1  1   1   0
## 2288   2288    0 480 24   1    1  1   1   0
## 2289   2289    0 480 24   1    1  1   1   0
## 2290   2290    0 480 24   1    1  1   1   0
## 2291   2291    0 480 24   1    1  1   1   0
## 2292   2292    0 480 24   1    1  1   1   0
## 2293   2293    0 480 24   1    1  1   1   0
## 2294   2294    0 480 24   1    1  1   1   0
## 2295   2295    0 480 24   1    1  1   1   0
## 2296   2296    0 480 24   1    1  1   1   0
## 2297   2297    0 480 24   1    1  1   1   0
## 2298   2298    0 480 24   1    1  1   1   0
## 2299   2299    0 480 24   1    1  1   1   0
## 2300   2300    0 480 24   1    1  1   1   0
## 2301   2301    0 480 24   1    1  1   1   0
## 2302   2302    0 480 24   1    1  1   1   0
## 2303   2303    0 480 24   1    1  1   1   0
## 2304   2304    0 480 24   1    1  1   1   0
## 2305   2305    0 480 24   1    1  1   1   0
## 2306   2306    0 480 24   1    1  1   1   0
## 2307   2307    0 480 24   1    1  1   1   0
## 2308   2308    0 480 24   1    1  1   1   0
## 2309   2309    0 480 24   1    1  1   1   0
## 2310   2310    0 480 24   1    1  1   1   0
## 2311   2311    0 480 24   1    1  1   1   0
## 2312   2312    0 480 24   1    1  1   1   0
## 2313   2313    0 480 24   1    1  1   1   0
## 2314   2314    0 480 24   1    1  1   1   0
## 2315   2315    0 480 24   1    1  1   1   0
## 2316   2316    0 480 24   1    1  1   1   0
## 2317   2317    0 480 24   1    1  1   1   0
## 2318   2318    0 480 24   1    1  1   1   0
## 2319   2319    0 480 24   1    1  1   1   0
## 2320   2320    0 480 24   1    1  1   1   0
## 2321   2321    0 480 24   1    1  1   1   0
## 2322   2322    0 480 24   1    1  1   1   0
## 2323   2323    0 480 24   1    1  1   1   0
## 2324   2324    0 480 24   1    1  1   1   0
## 2325   2325    0 480 24   1    1  1   1   0
## 2326   2326    0 480 24   1    1  1   1   0
## 2327   2327    0 480 24   1    1  1   1   0
## 2328   2328    0 480 24   1    1  1   1   0
## 2329   2329    0 480 24   1    1  1   1   0
## 2330   2330    0 480 24   1    1  1   1   0
## 2331   2331    0 480 24   1    1  1   1   0
## 2332   2332    0 480 24   1    1  1   1   0
## 2333   2333    0 480 24   1    1  1   1   0
## 2334   2334    0 480 24   1    1  1   1   0
## 2335   2335    0 480 24   1    1  1   1   0
## 2336   2336    0 480 24   1    1  1   1   0
## 2337   2337    0 480 24   1    1  1   1   0
## 2338   2338    0 480 24   1    1  1   1   0
## 2339   2339    0 480 24   1    1  1   1   0
## 2340   2340    0 480 24   1    1  1   1   0
## 2341   2341    0 480 24   1    1  1   1   0
## 2342   2342    0 480 24   1    1  1   1   0
## 2343   2343    0 480 24   1    1  1   1   0
## 2344   2344    0 480 24   1    1  1   1   0
## 2345   2345    0 480 24   1    1  1   1   0
## 2346   2346    0 480 24   1    1  1   1   0
## 2347   2347    0 480 24   1    1  1   1   0
## 2348   2348    0 480 24   1    1  1   1   0
## 2349   2349    0 480 24   1    1  1   1   0
## 2350   2350    0 480 24   1    1  1   1   0
## 2351   2351    0 480 24   1    1  1   1   0
## 2352   2352    0 480 24   1    1  1   1   0
## 2353   2353    0 480 24   1    1  1   1   0
## 2354   2354    0 480 24   1    1  1   1   0
## 2355   2355    0 480 24   1    1  1   1   0
## 2356   2356    0 480 24   1    1  1   1   0
## 2357   2357    0 480 24   1    1  1   1   0
## 2358   2358    0 480 24   1    1  1   1   0
## 2359   2359    0 480 24   1    1  1   1   0
## 2360   2360    0 480 24   1    1  1   1   0
## 2361   2361    0 480 24   1    1  1   1   0
## 2362   2362    0 480 24   1    1  1   1   0
## 2363   2363    0 480 24   1    1  1   1   0
## 2364   2364    0 480 24   1    1  1   1   0
## 2365   2365    0 480 24   1    1  1   1   0
## 2366   2366    0 480 24   1    1  1   1   0
## 2367   2367    0 480 24   1    1  1   1   0
## 2368   2368    0 480 24   1    1  1   1   0
## 2369   2369    0 480 24   1    1  1   1   0
## 2370   2370    0 480 24   1    1  1   1   0
## 2371   2371    0 480 24   1    1  1   1   0
## 2372   2372    0 480 24   1    1  1   1   0
## 2373   2373    0 480 24   1    1  1   1   0
## 2374   2374    0 480 24   1    1  1   1   0
## 2375   2375    0 480 24   1    1  1   1   0
## 2376   2376    0 480 24   1    1  1   1   0
## 2377   2377    0 480 24   1    1  1   1   0
## 2378   2378    0 480 24   1    1  1   1   0
## 2379   2379    0 480 24   1    1  1   1   0
## 2380   2380    0 480 24   1    1  1   1   0
## 2381   2381    0 480 24   1    1  1   1   0
## 2382   2382    0 480 24   1    1  1   1   0
## 2383   2383    0 480 24   1    1  1   1   0
## 2384   2384    0 480 24   1    1  1   1   0
## 2385   2385    0 480 24   1    1  1   1   0
## 2386   2386    0 480 24   1    1  1   1   0
## 2387   2387    0 480 24   1    1  1   1   0
## 2388   2388    0 480 24   1    1  1   1   0
## 2389   2389    0 480 24   1    1  1   1   0
## 2390   2390    0 480 24   1    1  1   1   0
## 2391   2391    0 480 24   1    1  1   1   0
## 2392   2392    0 480 24   1    1  1   1   0
## 2393   2393    0 480 24   1    1  1   1   0
## 2394   2394    0 480 24   1    1  1   1   0
## 2395   2395    0 480 24   1    1  1   1   0
## 2396   2396    0 480 24   1    1  1   1   0
## 2397   2397    0 480 24   1    1  1   1   0
## 2398   2398    0 480 24   1    1  1   1   0
## 2399   2399    0 480 24   1    1  1   1   0
## 2400   2400    0 480 24   1    1  1   1   0
## 2401   2401    0 480 24   1    1  1   1   0
## 2402   2402    0 480 24   1    1  1   1   0
## 2403   2403    0 480 24   1    1  1   1   0
## 2404   2404    0 480 24   1    1  1   1   0
## 2405   2405    0 480 24   1    1  1   1   0
## 2406   2406    0 480 24   1    1  1   1   0
## 2407   2407    0 480 24   1    1  1   1   0
## 2408   2408    0 480 24   1    1  1   1   0
## 2409   2409    0 480 24   1    1  1   1   0
## 2410   2410    0 480 24   1    1  1   1   0
## 2411   2411    0 480 24   1    1  1   1   0
## 2412   2412    0 480 24   1    1  1   1   0
## 2413   2413    0 480 24   1    1  1   1   0
## 2414   2414    0 480 24   1    1  1   1   0
## 2415   2415    0 480 24   1    1  1   1   0
## 2416   2416    0 480 24   1    1  1   1   0
## 2417   2417    0 480 24   1    1  1   1   0
## 2418   2418    0 480 24   1    1  1   1   0
## 2419   2419    0 480 24   1    1  1   1   0
## 2420   2420    0 480 24   1    1  1   1   0
## 2421   2421    0 480 24   1    1  1   1   0
## 2422   2422    0 480 24   1    1  1   1   0
## 2423   2423    0 480 24   1    1  1   1   0
## 2424   2424    0 480 24   1    1  1   1   0
## 2425   2425    0 480 24   1    1  1   1   0
## 2426   2426    0 480 24   1    1  1   1   0
## 2427   2427    0 480 24   1    1  1   1   0
## 2428   2428    0 480 24   1    1  1   1   0
## 2429   2429    0 480 24   1    1  1   1   0
## 2430   2430    0 480 24   1    1  1   1   0
## 2431   2431    0 480 24   1    1  1   1   0
## 2432   2432    0 480 24   1    1  1   1   0
## 2433   2433    0 480 24   1    1  1   1   0
## 2434   2434    0 480 24   1    1  1   1   0
## 2435   2435    0 480 24   1    1  1   1   0
## 2436   2436    0 480 24   1    1  1   1   0
## 2437   2437    0 480 24   1    1  1   1   0
## 2438   2438    0 480 24   1    1  1   1   0
## 2439   2439    0 480 24   1    1  1   1   0
## 2440   2440    0 480 24   1    1  1   1   0
## 2441   2441    0 480 24   1    1  1   1   0
## 2442   2442    0 480 24   1    1  1   1   0
## 2443   2443    0 480 24   1    1  1   1   0
## 2444   2444    0 480 24   1    1  1   1   0
## 2445   2445    0 480 24   1    1  1   1   0
## 2446   2446    0 480 24   1    1  1   1   0
## 2447   2447    0 480 24   1    1  1   1   0
## 2448   2448    0 480 24   1    1  1   1   0
## 2449   2449    0 480 24   1    1  1   1   0
## 2450   2450    0 480 24   1    1  1   1   0
## 2451   2451    0 480 24   1    1  1   1   0
## 2452   2452    0 480 24   1    1  1   1   0
## 2453   2453    0 480 24   1    1  1   1   0
## 2454   2454    0 480 24   1    1  1   1   0
## 2455   2455    0 480 24   1    1  1   1   0
## 2456   2456    0 480 24   1    1  1   1   0
## 2457   2457    0 480 24   1    1  1   1   0
## 2458   2458    0 480 24   1    1  1   1   0
## 2459   2459    0 480 24   1    1  1   1   0
## 2460   2460    0 480 24   1    1  1   1   0
## 2461   2461    0 480 24   1    1  1   1   0
## 2462   2462    0 480 24   1    1  1   1   0
## 2463   2463    0 480 24   1    1  1   1   0
## 2464   2464    0 480 24   1    1  1   1   0
## 2465   2465    0 480 24   1    1  1   1   0
## 2466   2466    0 480 24   1    1  1   1   0
## 2467   2467    0 480 24   1    1  1   1   0
## 2468   2468    0 480 24   1    1  1   1   0
## 2469   2469    0 480 24   1    1  1   1   0
## 2470   2470    0 480 24   1    1  1   1   0
## 2471   2471    0 480 24   1    1  1   1   0
## 2472   2472    0 480 24   1    1  1   1   0
## 2473   2473    0 480 24   1    1  1   1   0
## 2474   2474    0 480 24   1    1  1   1   0
## 2475   2475    0 480 24   1    1  1   1   0
## 2476   2476    0 480 24   1    1  1   1   0
## 2477   2477    0 480 24   1    1  1   1   0
## 2478   2478    0 480 24   1    1  1   1   0
## 2479   2479    0 480 24   1    1  1   1   0
## 2480   2480    0 480 24   1    1  1   1   0
## 2481   2481    0 480 24   1    1  1   1   0
## 2482   2482    0 480 24   1    1  1   1   0
## 2483   2483    0 480 24   1    1  1   1   0
## 2484   2484    0 480 24   1    1  1   1   0
## 2485   2485    0 480 24   1    1  1   1   0
## 2486   2486    0 480 24   1    1  1   1   0
## 2487   2487    0 480 24   1    1  1   1   0
## 2488   2488    0 480 24   1    1  1   1   0
## 2489   2489    0 480 24   1    1  1   1   0
## 2490   2490    0 480 24   1    1  1   1   0
## 2491   2491    0 480 24   1    1  1   1   0
## 2492   2492    0 480 24   1    1  1   1   0
## 2493   2493    0 480 24   1    1  1   1   0
## 2494   2494    0 480 24   1    1  1   1   0
## 2495   2495    0 480 24   1    1  1   1   0
## 2496   2496    0 480 24   1    1  1   1   0
## 2497   2497    0 480 24   1    1  1   1   0
## 2498   2498    0 480 24   1    1  1   1   0
## 2499   2499    0 480 24   1    1  1   1   0
## 2500   2500    0 480 24   1    1  1   1   0
## 2501   2501    0 480 24   1    1  1   1   0
## 2502   2502    0 480 24   1    1  1   1   0
## 2503   2503    0 480 24   1    1  1   1   0
## 2504   2504    0 480 24   1    1  1   1   0
## 2505   2505    0 480 24   1    1  1   1   0
## 2506   2506    0 480 24   1    1  1   1   0
## 2507   2507    0 480 24   1    1  1   1   0
## 2508   2508    0 480 24   1    1  1   1   0
## 2509   2509    0 480 24   1    1  1   1   0
## 2510   2510    0 480 24   1    1  1   1   0
## 2511   2511    0 480 24   1    1  1   1   0
## 2512   2512    0 480 24   1    1  1   1   0
## 2513   2513    0 480 24   1    1  1   1   0
## 2514   2514    0 480 24   1    1  1   1   0
## 2515   2515    0 480 24   1    1  1   1   0
## 2516   2516    0 480 24   1    1  1   1   0
## 2517   2517    0 480 24   1    1  1   1   0
## 2518   2518    0 480 24   1    1  1   1   0
## 2519   2519    0 480 24   1    1  1   1   0
## 2520   2520    0 480 24   1    1  1   1   0
## 2521   2521    0 480 24   1    1  1   1   0
## 2522   2522    0 480 24   1    1  1   1   0
## 2523   2523    0 480 24   1    1  1   1   0
## 2524   2524    0 480 24   1    1  1   1   0
## 2525   2525    0 480 24   1    1  1   1   0
## 2526   2526    0 480 24   1    1  1   1   0
## 2527   2527    0 480 24   1    1  1   1   0
## 2528   2528    0 480 24   1    1  1   1   0
## 2529   2529    0 480 24   1    1  1   1   0
## 2530   2530    0 480 24   1    1  1   1   0
## 2531   2531    0 480 24   1    1  1   1   0
## 2532   2532    0 480 24   1    1  1   1   0
## 2533   2533    0 480 24   1    1  1   1   0
## 2534   2534    0 480 24   1    1  1   1   0
## 2535   2535    0 480 24   1    1  1   1   0
## 2536   2536    0 480 24   1    1  1   1   0
## 2537   2537    0 480 24   1    1  1   1   0
## 2538   2538    0 480 24   1    1  1   1   0
## 2539   2539    0 480 24   1    1  1   1   0
## 2540   2540    0 480 24   1    1  1   1   0
## 2541   2541    0 480 24   1    1  1   1   0
## 2542   2542    0 480 24   1    1  1   1   0
## 2543   2543    0 480 24   1    1  1   1   0
## 2544   2544    0 480 24   1    1  1   1   0
## 2545   2545    0 480 24   1    1  1   1   0
## 2546   2546    0 480 24   1    1  1   1   0
## 2547   2547    0 480 24   1    1  1   1   0
## 2548   2548    0 480 24   1    1  1   1   0
## 2549   2549    0 480 24   1    1  1   1   0
## 2550   2550    0 480 24   1    1  1   1   0
## 2551   2551    0 480 24   1    1  1   1   0
## 2552   2552    0 480 24   1    1  1   1   0
## 2553   2553    0 480 24   1    1  1   1   0
## 2554   2554    0 480 24   1    1  1   1   0
## 2555   2555    0 480 24   1    1  1   1   0
## 2556   2556    0 480 24   1    1  1   1   0
## 2557   2557    0 480 24   1    1  1   1   0
## 2558   2558    0 480 24   1    1  1   1   0
## 2559   2559    0 480 24   1    1  1   1   0
## 2560   2560    0 480 24   1    1  1   1   0
## 2561   2561    0 480 24   1    1  1   1   0
## 2562   2562    0 480 24   1    1  1   1   0
## 2563   2563    0 480 24   1    1  1   1   0
## 2564   2564    0 480 24   1    1  1   1   0
## 2565   2565    0 480 24   1    1  1   1   0
## 2566   2566    0 480 24   1    1  1   1   0
## 2567   2567    0 480 24   1    1  1   1   0
## 2568   2568    0 480 24   1    1  1   1   0
## 2569   2569    0 480 24   1    1  1   1   0
## 2570   2570    0 480 24   1    1  1   1   0
## 2571   2571    0 480 24   1    1  1   1   0
## 2572   2572    0 480 24   1    1  1   1   0
## 2573   2573    0 480 24   1    1  1   1   0
## 2574   2574    0 480 24   1    1  1   1   0
## 2575   2575    0 480 24   1    1  1   1   0
## 2576   2576    0 480 24   1    1  1   1   0
## 2577   2577    0 480 24   1    1  1   1   0
## 2578   2578    0 480 24   1    1  1   1   0
## 2579   2579    0 480 24   1    1  1   1   0
## 2580   2580    0 480 24   1    1  1   1   0
## 2581   2581    0 480 24   1    1  1   1   0
## 2582   2582    0 480 24   1    1  1   1   0
## 2583   2583    0 480 24   1    1  1   1   0
## 2584   2584    0 480 24   1    1  1   1   0
## 2585   2585    0 480 24   1    1  1   1   0
## 2586   2586    0 480 24   1    1  1   1   0
## 2587   2587    0 480 24   1    1  1   1   0
## 2588   2588    0 480 24   1    1  1   1   0
## 2589   2589    0 480 24   1    1  1   1   0
## 2590   2590    0 480 24   1    1  1   1   0
## 2591   2591    0 480 24   1    1  1   1   0
## 2592   2592    0 480 24   1    1  1   1   0
## 2593   2593    0 480 24   1    1  1   1   0
## 2594   2594    0 480 24   1    1  1   1   0
## 2595   2595    0 480 24   1    1  1   1   0
## 2596   2596    0 480 24   1    1  1   1   0
## 2597   2597    0 480 24   1    1  1   1   0
## 2598   2598    0 480 24   1    1  1   1   0
## 2599   2599    0 480 24   1    1  1   1   0
## 2600   2600    0 480 24   1    1  1   1   0
## 2601   2601    0 480 24   1    1  1   1   0
## 2602   2602    0 480 24   1    1  1   1   0
## 2603   2603    0 480 24   1    1  1   1   0
## 2604   2604    0 480 24   1    1  1   1   0
## 2605   2605    0 480 24   1    1  1   1   0
## 2606   2606    0 480 24   1    1  1   1   0
## 2607   2607    0 480 24   1    1  1   1   0
## 2608   2608    0 480 24   1    1  1   1   0
## 2609   2609    0 480 24   1    1  1   1   0
## 2610   2610    0 480 24   1    1  1   1   0
## 2611   2611    0 480 24   1    1  1   1   0
## 2612   2612    0 480 24   1    1  1   1   0
## 2613   2613    0 480 24   1    1  1   1   0
## 2614   2614    0 480 24   1    1  1   1   0
## 2615   2615    0 480 24   1    1  1   1   0
## 2616   2616    0 480 24   1    1  1   1   0
## 2617   2617    0 480 24   1    1  1   1   0
## 2618   2618    0 480 24   1    1  1   1   0
## 2619   2619    0 480 24   1    1  1   1   0
## 2620   2620    0 480 24   1    1  1   1   0
## 2621   2621    0 480 24   1    1  1   1   0
## 2622   2622    0 480 24   1    1  1   1   0
## 2623   2623    0 480 24   1    1  1   1   0
## 2624   2624    0 480 24   1    1  1   1   0
## 2625   2625    0 480 24   1    1  1   1   0
## 2626   2626    0 480 24   1    1  1   1   0
## 2627   2627    0 480 24   1    1  1   1   0
## 2628   2628    0 480 24   1    1  1   1   0
## 2629   2629    0 480 24   1    1  1   1   0
## 2630   2630    0 480 24   1    1  1   1   0
## 2631   2631    0 480 24   1    1  1   1   0
## 2632   2632    0 480 24   1    1  1   1   0
## 2633   2633    0 480 24   1    1  1   1   0
## 2634   2634    0 480 24   1    1  1   1   0
## 2635   2635    0 480 24   1    1  1   1   0
## 2636   2636    0 480 24   1    1  1   1   0
## 2637   2637    0 480 24   1    1  1   1   0
## 2638   2638    0 480 24   1    1  1   1   0
## 2639   2639    0 480 24   1    1  1   1   0
## 2640   2640    0 480 24   1    1  1   1   0
## 2641   2641    0 480 24   1    1  1   1   0
## 2642   2642    0 480 24   1    1  1   1   0
## 2643   2643    0 480 24   1    1  1   1   0
## 2644   2644    0 480 24   1    1  1   1   0
## 2645   2645    0 480 24   1    1  1   1   0
## 2646   2646    0 480 24   1    1  1   1   0
## 2647   2647    0 480 24   1    1  1   1   0
## 2648   2648    0 480 24   1    1  1   1   0
## 2649   2649    0 480 24   1    1  1   1   0
## 2650   2650    0 480 24   1    1  1   1   0
## 2651   2651    0 480 24   1    1  1   1   0
## 2652   2652    0 480 24   1    1  1   1   0
## 2653   2653    0 480 24   1    1  1   1   0
## 2654   2654    0 480 24   1    1  1   1   0
## 2655   2655    0 480 24   1    1  1   1   0
## 2656   2656    0 480 24   1    1  1   1   0
## 2657   2657    0 480 24   1    1  1   1   0
## 2658   2658    0 480 24   1    1  1   1   0
## 2659   2659    0 480 24   1    1  1   1   0
## 2660   2660    0 480 24   1    1  1   1   0
## 2661   2661    0 480 24   1    1  1   1   0
## 2662   2662    0 480 24   1    1  1   1   0
## 2663   2663    0 480 24   1    1  1   1   0
## 2664   2664    0 480 24   1    1  1   1   0
## 2665   2665    0 480 24   1    1  1   1   0
## 2666   2666    0 480 24   1    1  1   1   0
## 2667   2667    0 480 24   1    1  1   1   0
## 2668   2668    0 480 24   1    1  1   1   0
## 2669   2669    0 480 24   1    1  1   1   0
## 2670   2670    0 480 24   1    1  1   1   0
## 2671   2671    0 480 24   1    1  1   1   0
## 2672   2672    0 480 24   1    1  1   1   0
## 2673   2673    0 480 24   1    1  1   1   0
## 2674   2674    0 480 24   1    1  1   1   0
## 2675   2675    0 480 24   1    1  1   1   0
## 2676   2676    0 480 24   1    1  1   1   0
## 2677   2677    0 480 24   1    1  1   1   0
## 2678   2678    0 480 24   1    1  1   1   0
## 2679   2679    0 480 24   1    1  1   1   0
## 2680   2680    0 480 24   1    1  1   1   0
## 2681   2681    0 480 24   1    1  1   1   0
## 2682   2682    0 480 24   1    1  1   1   0
## 2683   2683    0 480 24   1    1  1   1   0
## 2684   2684    0 480 24   1    1  1   1   0
## 2685   2685    0 480 24   1    1  1   1   0
## 2686   2686    0 480 24   1    1  1   1   0
## 2687   2687    0 480 24   1    1  1   1   0
## 2688   2688    0 480 24   1    1  1   1   0
## 2689   2689    0 480 24   1    1  1   1   0
## 2690   2690    0 480 24   1    1  1   1   0
## 2691   2691    0 480 24   1    1  1   1   0
## 2692   2692    0 480 24   1    1  1   1   0
## 2693   2693    0 480 24   1    1  1   1   0
## 2694   2694    0 480 24   1    1  1   1   0
## 2695   2695    0 480 24   1    1  1   1   0
## 2696   2696    0 480 24   1    1  1   1   0
## 2697   2697    0 480 24   1    1  1   1   0
## 2698   2698    0 480 24   1    1  1   1   0
## 2699   2699    0 480 24   1    1  1   1   0
## 2700   2700    0 480 24   1    1  1   1   0
## 2701   2701    0 480 24   1    1  1   1   0
## 2702   2702    0 480 24   1    1  1   1   0
## 2703   2703    0 480 24   1    1  1   1   0
## 2704   2704    0 480 24   1    1  1   1   0
## 2705   2705    0 480 24   1    1  1   1   0
## 2706   2706    0 480 24   1    1  1   1   0
## 2707   2707    0 480 24   1    1  1   1   0
## 2708   2708    0 480 24   1    1  1   1   0
## 2709   2709    0 480 24   1    1  1   1   0
## 2710   2710    0 480 24   1    1  1   1   0
## 2711   2711    0 480 24   1    1  1   1   0
## 2712   2712    0 480 24   1    1  1   1   0
## 2713   2713    0 480 24   1    1  1   1   0
## 2714   2714    0 480 24   1    1  1   1   0
## 2715   2715    0 480 24   1    1  1   1   0
## 2716   2716    0 480 24   1    1  1   1   0
## 2717   2717    0 480 24   1    1  1   1   0
## 2718   2718    0 480 24   1    1  1   1   0
## 2719   2719    0 480 24   1    1  1   1   0
## 2720   2720    0 480 24   1    1  1   1   0
## 2721   2721    0 480 24   1    1  1   1   0
## 2722   2722    0 480 24   1    1  1   1   0
## 2723   2723    0 480 24   1    1  1   1   0
## 2724   2724    0 480 24   1    1  1   1   0
## 2725   2725    0 480 24   1    1  1   1   0
## 2726   2726    0 480 24   1    1  1   1   0
## 2727   2727    0 480 24   1    1  1   1   0
## 2728   2728    0 480 24   1    1  1   1   0
## 2729   2729    0 480 24   1    1  1   1   0
## 2730   2730    0 480 24   1    1  1   1   0
## 2731   2731    0 480 24   1    1  1   1   0
## 2732   2732    0 480 24   1    1  1   1   0
## 2733   2733    0 480 24   1    1  1   1   0
## 2734   2734    0 480 24   1    1  1   1   0
## 2735   2735    0 480 24   1    1  1   1   0
## 2736   2736    0 480 24   1    1  1   1   0
## 2737   2737    0 480 24   1    1  1   1   0
## 2738   2738    0 480 24   1    1  1   1   0
## 2739   2739    0 480 24   1    1  1   1   0
## 2740   2740    0 480 24   1    1  1   1   0
## 2741   2741    0 480 24   1    1  1   1   0
## 2742   2742    0 480 24   1    1  1   1   0
## 2743   2743    0 480 24   1    1  1   1   0
## 2744   2744    0 480 24   1    1  1   1   0
## 2745   2745    0 480 24   1    1  1   1   0
## 2746   2746    0 480 24   1    1  1   1   0
## 2747   2747    0 480 24   1    1  1   1   0
## 2748   2748    0 480 24   1    1  1   1   0
## 2749   2749    0 480 24   1    1  1   1   0
## 2750   2750    0 480 24   1    1  1   1   0
## 2751   2751    0 480 24   1    1  1   1   0
## 2752   2752    0 480 24   1    1  1   1   0
## 2753   2753    0 480 24   1    1  1   1   0
## 2754   2754    0 480 24   1    1  1   1   0
## 2755   2755    0 480 24   1    1  1   1   0
## 2756   2756    0 480 24   1    1  1   1   0
## 2757   2757    0 480 24   1    1  1   1   0
## 2758   2758    0 480 24   1    1  1   1   0
## 2759   2759    0 480 24   1    1  1   1   0
## 2760   2760    0 480 24   1    1  1   1   0
## 2761   2761    0 480 24   1    1  1   1   0
## 2762   2762    0 480 24   1    1  1   1   0
## 2763   2763    0 480 24   1    1  1   1   0
## 2764   2764    0 480 24   1    1  1   1   0
## 2765   2765    0 480 24   1    1  1   1   0
## 2766   2766    0 480 24   1    1  1   1   0
## 2767   2767    0 480 24   1    1  1   1   0
## 2768   2768    0 480 24   1    1  1   1   0
## 2769   2769    0 480 24   1    1  1   1   0
## 2770   2770    0 480 24   1    1  1   1   0
## 2771   2771    0 480 24   1    1  1   1   0
## 2772   2772    0 480 24   1    1  1   1   0
## 2773   2773    0 480 24   1    1  1   1   0
## 2774   2774    0 480 24   1    1  1   1   0
## 2775   2775    0 480 24   1    1  1   1   0
## 2776   2776    0 480 24   1    1  1   1   0
## 2777   2777    0 480 24   1    1  1   1   0
## 2778   2778    0 480 24   1    1  1   1   0
## 2779   2779    0 480 24   1    1  1   1   0
## 2780   2780    0 480 24   1    1  1   1   0
## 2781   2781    0 480 24   1    1  1   1   0
## 2782   2782    0 480 24   1    1  1   1   0
## 2783   2783    0 480 24   1    1  1   1   0
## 2784   2784    0 480 24   1    1  1   1   0
## 2785   2785    0 480 24   1    1  1   1   0
## 2786   2786    0 480 24   1    1  1   1   0
## 2787   2787    0 480 24   1    1  1   1   0
## 2788   2788    0 480 24   1    1  1   1   0
## 2789   2789    0 480 24   1    1  1   1   0
## 2790   2790    0 480 24   1    1  1   1   0
## 2791   2791    0 480 24   1    1  1   1   0
## 2792   2792    0 480 24   1    1  1   1   0
## 2793   2793    0 480 24   1    1  1   1   0
## 2794   2794    0 480 24   1    1  1   1   0
## 2795   2795    0 480 24   1    1  1   1   0
## 2796   2796    0 480 24   1    1  1   1   0
## 2797   2797    0 480 24   1    1  1   1   0
## 2798   2798    0 480 24   1    1  1   1   0
## 2799   2799    0 480 24   1    1  1   1   0
## 2800   2800    0 480 24   1    1  1   1   0
## 2801   2801    0 480 24   1    1  1   1   0
## 2802   2802    0 480 24   1    1  1   1   0
## 2803   2803    0 480 24   1    1  1   1   0
## 2804   2804    0 480 24   1    1  1   1   0
## 2805   2805    0 480 24   1    1  1   1   0
## 2806   2806    0 480 24   1    1  1   1   0
## 2807   2807    0 480 24   1    1  1   1   0
## 2808   2808    0 480 24   1    1  1   1   0
## 2809   2809    0 480 24   1    1  1   1   0
## 2810   2810    0 480 24   1    1  1   1   0
## 2811   2811    0 480 24   1    1  1   1   0
## 2812   2812    0 480 24   1    1  1   1   0
## 2813   2813    0 480 24   1    1  1   1   0
## 2814   2814    0 480 24   1    1  1   1   0
## 2815   2815    0 480 24   1    1  1   1   0
## 2816   2816    0 480 24   1    1  1   1   0
## 2817   2817    0 480 24   1    1  1   1   0
## 2818   2818    0 480 24   1    1  1   1   0
## 2819   2819    0 480 24   1    1  1   1   0
## 2820   2820    0 480 24   1    1  1   1   0
## 2821   2821    0 480 24   1    1  1   1   0
## 2822   2822    0 480 24   1    1  1   1   0
## 2823   2823    0 480 24   1    1  1   1   0
## 2824   2824    0 480 24   1    1  1   1   0
## 2825   2825    0 480 24   1    1  1   1   0
## 2826   2826    0 480 24   1    1  1   1   0
## 2827   2827    0 480 24   1    1  1   1   0
## 2828   2828    0 480 24   1    1  1   1   0
## 2829   2829    0 480 24   1    1  1   1   0
## 2830   2830    0 480 24   1    1  1   1   0
## 2831   2831    0 480 24   1    1  1   1   0
## 2832   2832    0 480 24   1    1  1   1   0
## 2833   2833    0 480 24   1    1  1   1   0
## 2834   2834    0 480 24   1    1  1   1   0
## 2835   2835    0 480 24   1    1  1   1   0
## 2836   2836    0 480 24   1    1  1   1   0
## 2837   2837    0 480 24   1    1  1   1   0
## 2838   2838    0 480 24   1    1  1   1   0
## 2839   2839    0 480 24   1    1  1   1   0
## 2840   2840    0 480 24   1    1  1   1   0
## 2841   2841    0 480 24   1    1  1   1   0
## 2842   2842    0 480 24   1    1  1   1   0
## 2843   2843    0 480 24   1    1  1   1   0
## 2844   2844    0 480 24   1    1  1   1   0
## 2845   2845    0 480 24   1    1  1   1   0
## 2846   2846    0 480 24   1    1  1   1   0
## 2847   2847    0 480 24   1    1  1   1   0
## 2848   2848    0 480 24   1    1  1   1   0
## 2849   2849    0 480 24   1    1  1   1   0
## 2850   2850    0 480 24   1    1  1   1   0
## 2851   2851    0 480 24   1    1  1   1   0
## 2852   2852    0 480 24   1    1  1   1   0
## 2853   2853    0 480 24   1    1  1   1   0
## 2854   2854    0 480 24   1    1  1   1   0
## 2855   2855    0 480 24   1    1  1   1   0
## 2856   2856    0 480 24   1    1  1   1   0
## 2857   2857    0 480 24   1    1  1   1   0
## 2858   2858    0 480 24   1    1  1   1   0
## 2859   2859    0 480 24   1    1  1   1   0
## 2860   2860    0 480 24   1    1  1   1   0
## 2861   2861    0 480 24   1    1  1   1   0
## 2862   2862    0 480 24   1    1  1   1   0
## 2863   2863    0 480 24   1    1  1   1   0
## 2864   2864    0 480 24   1    1  1   1   0
## 2865   2865    0 480 24   1    1  1   1   0
## 2866   2866    0 480 24   1    1  1   1   0
## 2867   2867    0 480 24   1    1  1   1   0
## 2868   2868    0 480 24   1    1  1   1   0
## 2869   2869    0 480 24   1    1  1   1   0
## 2870   2870    0 480 24   1    1  1   1   0
## 2871   2871    0 480 24   1    1  1   1   0
## 2872   2872    0 480 24   1    1  1   1   0
## 2873   2873    0 480 24   1    1  1   1   0
## 2874   2874    0 480 24   1    1  1   1   0
## 2875   2875    0 480 24   1    1  1   1   0
## 2876   2876    0 480 24   1    1  1   1   0
## 2877   2877    0 480 24   1    1  1   1   0
## 2878   2878    0 480 24   1    1  1   1   0
## 2879   2879    0 480 24   1    1  1   1   0
## 2880   2880    0 480 24   1    1  1   1   0
## 2881   2881    0 480 24   1    1  1   1   0
## 2882   2882    0 480 24   1    1  1   1   0
## 2883   2883    0 480 24   1    1  1   1   0
## 2884   2884    0 480 24   1    1  1   1   0
## 2885   2885    0 480 24   1    1  1   1   0
## 2886   2886    0 480 24   1    1  1   1   0
## 2887   2887    0 480 24   1    1  1   1   0
## 2888   2888    0 480 24   1    1  1   1   0
## 2889   2889    0 480 24   1    1  1   1   0
## 2890   2890    0 480 24   1    1  1   1   0
## 2891   2891    0 480 24   1    1  1   1   0
## 2892   2892    0 480 24   1    1  1   1   0
## 2893   2893    0 480 24   1    1  1   1   0
## 2894   2894    0 480 24   1    1  1   1   0
## 2895   2895    0 480 24   1    1  1   1   0
## 2896   2896    0 480 24   1    1  1   1   0
## 2897   2897    0 480 24   1    1  1   1   0
## 2898   2898    0 480 24   1    1  1   1   0
## 2899   2899    0 480 24   1    1  1   1   0
## 2900   2900    0 480 24   1    1  1   1   0
## 2901   2901    0 480 24   1    1  1   1   0
## 2902   2902    0 480 24   1    1  1   1   0
## 2903   2903    0 480 24   1    1  1   1   0
## 2904   2904    0 480 24   1    1  1   1   0
## 2905   2905    0 480 24   1    1  1   1   0
## 2906   2906    0 480 24   1    1  1   1   0
## 2907   2907    0 480 24   1    1  1   1   0
## 2908   2908    0 480 24   1    1  1   1   0
## 2909   2909    0 480 24   1    1  1   1   0
## 2910   2910    0 480 24   1    1  1   1   0
## 2911   2911    0 480 24   1    1  1   1   0
## 2912   2912    0 480 24   1    1  1   1   0
## 2913   2913    0 480 24   1    1  1   1   0
## 2914   2914    0 480 24   1    1  1   1   0
## 2915   2915    0 480 24   1    1  1   1   0
## 2916   2916    0 480 24   1    1  1   1   0
## 2917   2917    0 480 24   1    1  1   1   0
## 2918   2918    0 480 24   1    1  1   1   0
## 2919   2919    0 480 24   1    1  1   1   0
## 2920   2920    0 480 24   1    1  1   1   0
## 2921   2921    0 480 24   1    1  1   1   0
## 2922   2922    0 480 24   1    1  1   1   0
## 2923   2923    0 480 24   1    1  1   1   0
## 2924   2924    0 480 24   1    1  1   1   0
## 2925   2925    0 480 24   1    1  1   1   0
## 2926   2926    0 480 24   1    1  1   1   0
## 2927   2927    0 480 24   1    1  1   1   0
## 2928   2928    0 480 24   1    1  1   1   0
## 2929   2929    0 480 24   1    1  1   1   0
## 2930   2930    0 480 24   1    1  1   1   0
## 2931   2931    0 480 24   1    1  1   1   0
## 2932   2932    0 480 24   1    1  1   1   0
## 2933   2933    0 480 24   1    1  1   1   0
## 2934   2934    0 480 24   1    1  1   1   0
## 2935   2935    0 480 24   1    1  1   1   0
## 2936   2936    0 480 24   1    1  1   1   0
## 2937   2937    0 480 24   1    1  1   1   0
## 2938   2938    0 480 24   1    1  1   1   0
## 2939   2939    0 480 24   1    1  1   1   0
## 2940   2940    0 480 24   1    1  1   1   0
## 2941   2941    0 480 24   1    1  1   1   0
## 2942   2942    0 480 24   1    1  1   1   0
## 2943   2943    0 480 24   1    1  1   1   0
## 2944   2944    0 480 24   1    1  1   1   0
## 2945   2945    0 480 24   1    1  1   1   0
## 2946   2946    0 480 24   1    1  1   1   0
## 2947   2947    0 480 24   1    1  1   1   0
## 2948   2948    0 480 24   1    1  1   1   0
## 2949   2949    0 480 24   1    1  1   1   0
## 2950   2950    0 480 24   1    1  1   1   0
## 2951   2951    0 480 24   1    1  1   1   0
## 2952   2952    0 480 24   1    1  1   1   0
## 2953   2953    0 480 24   1    1  1   1   0
## 2954   2954    0 480 24   1    1  1   1   0
## 2955   2955    0 480 24   1    1  1   1   0
## 2956   2956    0 480 24   1    1  1   1   0
## 2957   2957    0 480 24   1    1  1   1   0
## 2958   2958    0 480 24   1    1  1   1   0
## 2959   2959    0 480 24   1    1  1   1   0
## 2960   2960    0 480 24   1    1  1   1   0
## 2961   2961    0 480 24   1    1  1   1   0
## 2962   2962    0 480 24   1    1  1   1   0
## 2963   2963    0 480 24   1    1  1   1   0
## 2964   2964    0 480 24   1    1  1   1   0
## 2965   2965    0 480 24   1    1  1   1   0
## 2966   2966    0 480 24   1    1  1   1   0
## 2967   2967    0 480 24   1    1  1   1   0
## 2968   2968    0 480 24   1    1  1   1   0
## 2969   2969    0 480 24   1    1  1   1   0
## 2970   2970    0 480 24   1    1  1   1   0
## 2971   2971    0 480 24   1    1  1   1   0
## 2972   2972    0 480 24   1    1  1   1   0
## 2973   2973    0 480 24   1    1  1   1   0
## 2974   2974    0 480 24   1    1  1   1   0
## 2975   2975    0 480 24   1    1  1   1   0
## 2976   2976    0 480 24   1    1  1   1   0
## 2977   2977    0 480 24   1    1  1   1   0
## 2978   2978    0 480 24   1    1  1   1   0
## 2979   2979    0 480 24   1    1  1   1   0
## 2980   2980    0 480 24   1    1  1   1   0
## 2981   2981    0 480 24   1    1  1   1   0
## 2982   2982    0 480 24   1    1  1   1   0
## 2983   2983    0 480 24   1    1  1   1   0
## 2984   2984    0 480 24   1    1  1   1   0
## 2985   2985    0 480 24   1    1  1   1   0
## 2986   2986    0 480 24   1    1  1   1   0
## 2987   2987    0 480 24   1    1  1   1   0
## 2988   2988    0 480 24   1    1  1   1   0
## 2989   2989    0 480 24   1    1  1   1   0
## 2990   2990    0 480 24   1    1  1   1   0
## 2991   2991    0 480 24   1    1  1   1   0
## 2992   2992    0 480 24   1    1  1   1   0
## 2993   2993    0 480 24   1    1  1   1   0
## 2994   2994    0 480 24   1    1  1   1   0
## 2995   2995    0 480 24   1    1  1   1   0
## 2996   2996    0 480 24   1    1  1   1   0
## 2997   2997    0 480 24   1    1  1   1   0
## 2998   2998    0 480 24   1    1  1   1   0
## 2999   2999    0 480 24   1    1  1   1   0
## 3000   3000    0 480 24   1    1  1   1   0
## 3001   3001    0 480 24   1    1  1   1   0
## 3002   3002    0 480 24   1    1  1   1   0
## 3003   3003    0 480 24   1    1  1   1   0
## 3004   3004    0 480 24   1    1  1   1   0
## 3005   3005    0 480 24   1    1  1   1   0
## 3006   3006    0 480 24   1    1  1   1   0
## 3007   3007    0 480 24   1    1  1   1   0
## 3008   3008    0 480 24   1    1  1   1   0
## 3009   3009    0 480 24   1    1  1   1   0
## 3010   3010    0 480 24   1    1  1   1   0
## 3011   3011    0 480 24   1    1  1   1   0
## 3012   3012    0 480 24   1    1  1   1   0
## 3013   3013    0 480 24   1    1  1   1   0
## 3014   3014    0 480 24   1    1  1   1   0
## 3015   3015    0 480 24   1    1  1   1   0
## 3016   3016    0 480 24   1    1  1   1   0
## 3017   3017    0 480 24   1    1  1   1   0
## 3018   3018    0 480 24   1    1  1   1   0
## 3019   3019    0 480 24   1    1  1   1   0
## 3020   3020    0 480 24   1    1  1   1   0
## 3021   3021    0 480 24   1    1  1   1   0
## 3022   3022    0 480 24   1    1  1   1   0
## 3023   3023    0 480 24   1    1  1   1   0
## 3024   3024    0 480 24   1    1  1   1   0
## 3025   3025    0 480 24   1    1  1   1   0
## 3026   3026    0 480 24   1    1  1   1   0
## 3027   3027    0 480 24   1    1  1   1   0
## 3028   3028    0 480 24   1    1  1   1   0
## 3029   3029    0 480 24   1    1  1   1   0
## 3030   3030    0 480 24   1    1  1   1   0
## 3031   3031    0 480 24   1    1  1   1   0
## 3032   3032    0 480 24   1    1  1   1   0
## 3033   3033    0 480 24   1    1  1   1   0
## 3034   3034    0 480 24   1    1  1   1   0
## 3035   3035    0 480 24   1    1  1   1   0
## 3036   3036    0 480 24   1    1  1   1   0
## 3037   3037    0 480 24   1    1  1   1   0
## 3038   3038    0 480 24   1    1  1   1   0
## 3039   3039    0 480 24   1    1  1   1   0
## 3040   3040    0 480 24   1    1  1   1   0
## 3041   3041    0 480 24   1    1  1   1   0
## 3042   3042    0 480 24   1    1  1   1   0
## 3043   3043    0 480 24   1    1  1   1   0
## 3044   3044    0 480 24   1    1  1   1   0
## 3045   3045    0 480 24   1    1  1   1   0
## 3046   3046    0 480 24   1    1  1   1   0
## 3047   3047    0 480 24   1    1  1   1   0
## 3048   3048    0 480 24   1    1  1   1   0
## 3049   3049    0 480 24   1    1  1   1   0
## 3050   3050    0 480 24   1    1  1   1   0
## 3051   3051    0 480 24   1    1  1   1   0
## 3052   3052    0 480 24   1    1  1   1   0
## 3053   3053    0 480 24   1    1  1   1   0
## 3054   3054    0 480 24   1    1  1   1   0
## 3055   3055    0 480 24   1    1  1   1   0
## 3056   3056    0 480 24   1    1  1   1   0
## 3057   3057    0 480 24   1    1  1   1   0
## 3058   3058    0 480 24   1    1  1   1   0
## 3059   3059    0 480 24   1    1  1   1   0
## 3060   3060    0 480 24   1    1  1   1   0
## 3061   3061    0 480 24   1    1  1   1   0
## 3062   3062    0 480 24   1    1  1   1   0
## 3063   3063    0 480 24   1    1  1   1   0
## 3064   3064    0 480 24   1    1  1   1   0
## 3065   3065    0 480 24   1    1  1   1   0
## 3066   3066    0 480 24   1    1  1   1   0
## 3067   3067    0 480 24   1    1  1   1   0
## 3068   3068    0 480 24   1    1  1   1   0
## 3069   3069    0 480 24   1    1  1   1   0
## 3070   3070    0 480 24   1    1  1   1   0
## 3071   3071    0 480 24   1    1  1   1   0
## 3072   3072    0 480 24   1    1  1   1   0
## 3073   3073    0 480 24   1    1  1   1   0
## 3074   3074    0 480 24   1    1  1   1   0
## 3075   3075    0 480 24   1    1  1   1   0
## 3076   3076    0 480 24   1    1  1   1   0
## 3077   3077    0 480 24   1    1  1   1   0
## 3078   3078    0 480 24   1    1  1   1   0
## 3079   3079    0 480 24   1    1  1   1   0
## 3080   3080    0 480 24   1    1  1   1   0
## 3081   3081    0 480 24   1    1  1   1   0
## 3082   3082    0 480 24   1    1  1   1   0
## 3083   3083    0 480 24   1    1  1   1   0
## 3084   3084    0 480 24   1    1  1   1   0
## 3085   3085    0 480 24   1    1  1   1   0
## 3086   3086    0 480 24   1    1  1   1   0
## 3087   3087    0 480 24   1    1  1   1   0
## 3088   3088    0 480 24   1    1  1   1   0
## 3089   3089    0 480 24   1    1  1   1   0
## 3090   3090    0 480 24   1    1  1   1   0
## 3091   3091    0 480 24   1    1  1   1   0
## 3092   3092    0 480 24   1    1  1   1   0
## 3093   3093    0 480 24   1    1  1   1   0
## 3094   3094    0 480 24   1    1  1   1   0
## 3095   3095    0 480 24   1    1  1   1   0
## 3096   3096    0 480 24   1    1  1   1   0
## 3097   3097    0 480 24   1    1  1   1   0
## 3098   3098    0 480 24   1    1  1   1   0
## 3099   3099    0 480 24   1    1  1   1   0
## 3100   3100    0 480 24   1    1  1   1   0
## 3101   3101    0 480 24   1    1  1   1   0
## 3102   3102    0 480 24   1    1  1   1   0
## 3103   3103    0 480 24   1    1  1   1   0
## 3104   3104    0 480 24   1    1  1   1   0
## 3105   3105    0 480 24   1    1  1   1   0
## 3106   3106    0 480 24   1    1  1   1   0
## 3107   3107    0 480 24   1    1  1   1   0
## 3108   3108    0 480 24   1    1  1   1   0
## 3109   3109    0 480 24   1    1  1   1   0
## 3110   3110    0 480 24   1    1  1   1   0
## 3111   3111    0 480 24   1    1  1   1   0
## 3112   3112    0 480 24   1    1  1   1   0
## 3113   3113    0 480 24   1    1  1   1   0
## 3114   3114    0 480 24   1    1  1   1   0
## 3115   3115    0 480 24   1    1  1   1   0
## 3116   3116    0 480 24   1    1  1   1   0
## 3117   3117    0 480 24   1    1  1   1   0
## 3118   3118    0 480 24   1    1  1   1   0
## 3119   3119    0 480 24   1    1  1   1   0
## 3120   3120    0 480 24   1    1  1   1   0
## 3121   3121    0 480 24   1    1  1   1   0
## 3122   3122    0 480 24   1    1  1   1   0
## 3123   3123    0 480 24   1    1  1   1   0
## 3124   3124    0 480 24   1    1  1   1   0
## 3125   3125    0 480 24   1    1  1   1   0
## 3126   3126    0 480 24   1    1  1   1   0
## 3127   3127    0 480 24   1    1  1   1   0
## 3128   3128    0 480 24   1    1  1   1   0
## 3129   3129    0 480 24   1    1  1   1   0
## 3130   3130    0 480 24   1    1  1   1   0
## 3131   3131    0 480 24   1    1  1   1   0
## 3132   3132    0 480 24   1    1  1   1   0
## 3133   3133    0 480 24   1    1  1   1   0
## 3134   3134    0 480 24   1    1  1   1   0
## 3135   3135    0 480 24   1    1  1   1   0
## 3136   3136    0 480 24   1    1  1   1   0
## 3137   3137    0 480 24   1    1  1   1   0
## 3138   3138    0 480 24   1    1  1   1   0
## 3139   3139    0 480 24   1    1  1   1   0
## 3140   3140    0 480 24   1    1  1   1   0
## 3141   3141    0 480 24   1    1  1   1   0
## 3142   3142    0 480 24   1    1  1   1   0
## 3143   3143    0 480 24   1    1  1   1   0
## 3144   3144    0 480 24   1    1  1   1   0
## 3145   3145    0 480 24   1    1  1   1   0
## 3146   3146    0 480 24   1    1  1   1   0
## 3147   3147    0 480 24   1    1  1   1   0
## 3148   3148    0 480 24   1    1  1   1   0
## 3149   3149    0 480 24   1    1  1   1   0
## 3150   3150    0 480 24   1    1  1   1   0
## 3151   3151    0 480 24   1    1  1   1   0
## 3152   3152    0 480 24   1    1  1   1   0
## 3153   3153    0 480 24   1    1  1   1   0
## 3154   3154    0 480 24   1    1  1   1   0
## 3155   3155    0 480 24   1    1  1   1   0
## 3156   3156    0 480 24   1    1  1   1   0
## 3157   3157    0 480 24   1    1  1   1   0
## 3158   3158    0 480 24   1    1  1   1   0
## 3159   3159    0 480 24   1    1  1   1   0
## 3160   3160    0 480 24   1    1  1   1   0
## 3161   3161    0 480 24   1    1  1   1   0
## 3162   3162    0 480 24   1    1  1   1   0
## 3163   3163    0 480 24   1    1  1   1   0
## 3164   3164    0 480 24   1    1  1   1   0
## 3165   3165    0 480 24   1    1  1   1   0
## 3166   3166    0 480 24   1    1  1   1   0
## 3167   3167    0 480 24   1    1  1   1   0
## 3168   3168    0 480 24   1    1  1   1   0
## 3169   3169    0 480 24   1    1  1   1   0
## 3170   3170    0 480 24   1    1  1   1   0
## 3171   3171    0 480 24   1    1  1   1   0
## 3172   3172    0 480 24   1    1  1   1   0
## 3173   3173    0 480 24   1    1  1   1   0
## 3174   3174    0 480 24   1    1  1   1   0
## 3175   3175    0 480 24   1    1  1   1   0
## 3176   3176    0 480 24   1    1  1   1   0
## 3177   3177    0 480 24   1    1  1   1   0
## 3178   3178    0 480 24   1    1  1   1   0
## 3179   3179    0 480 24   1    1  1   1   0
## 3180   3180    0 480 24   1    1  1   1   0
## 3181   3181    0 480 24   1    1  1   1   0
## 3182   3182    0 480 24   1    1  1   1   0
## 3183   3183    0 480 24   1    1  1   1   0
## 3184   3184    0 480 24   1    1  1   1   0
## 3185   3185    0 480 24   1    1  1   1   0
## 3186   3186    0 480 24   1    1  1   1   0
## 3187   3187    0 480 24   1    1  1   1   0
## 3188   3188    0 480 24   1    1  1   1   0
## 3189   3189    0 480 24   1    1  1   1   0
## 3190   3190    0 480 24   1    1  1   1   0
## 3191   3191    0 480 24   1    1  1   1   0
## 3192   3192    0 480 24   1    1  1   1   0
## 3193   3193    0 480 24   1    1  1   1   0
## 3194   3194    0 480 24   1    1  1   1   0
## 3195   3195    0 480 24   1    1  1   1   0
## 3196   3196    0 480 24   1    1  1   1   0
## 3197   3197    0 480 24   1    1  1   1   0
## 3198   3198    0 480 24   1    1  1   1   0
## 3199   3199    0 480 24   1    1  1   1   0
## 3200   3200    0 480 24   1    1  1   1   0
## 3201   3201    0 480 24   1    1  1   1   0
## 3202   3202    0 480 24   1    1  1   1   0
## 3203   3203    0 480 24   1    1  1   1   0
## 3204   3204    0 480 24   1    1  1   1   0
## 3205   3205    0 480 24   1    1  1   1   0
## 3206   3206    0 480 24   1    1  1   1   0
## 3207   3207    0 480 24   1    1  1   1   0
## 3208   3208    0 480 24   1    1  1   1   0
## 3209   3209    0 480 24   1    1  1   1   0
## 3210   3210    0 480 24   1    1  1   1   0
## 3211   3211    0 480 24   1    1  1   1   0
## 3212   3212    0 480 24   1    1  1   1   0
## 3213   3213    0 480 24   1    1  1   1   0
## 3214   3214    0 480 24   1    1  1   1   0
## 3215   3215    0 480 24   1    1  1   1   0
## 3216   3216    0 480 24   1    1  1   1   0
## 3217   3217    0 480 24   1    1  1   1   0
## 3218   3218    0 480 24   1    1  1   1   0
## 3219   3219    0 480 24   1    1  1   1   0
## 3220   3220    0 480 24   1    1  1   1   0
## 3221   3221    0 480 24   1    1  1   1   0
## 3222   3222    0 480 24   1    1  1   1   0
## 3223   3223    0 480 24   1    1  1   1   0
## 3224   3224    0 480 24   1    1  1   1   0
## 3225   3225    0 480 24   1    1  1   1   0
## 3226   3226    0 480 24   1    1  1   1   0
## 3227   3227    0 480 24   1    1  1   1   0
## 3228   3228    0 480 24   1    1  1   1   0
## 3229   3229    0 480 24   1    1  1   1   0
## 3230   3230    0 480 24   1    1  1   1   0
## 3231   3231    0 480 24   1    1  1   1   0
## 3232   3232    0 480 24   1    1  1   1   0
## 3233   3233    0 480 24   1    1  1   1   0
## 3234   3234    0 480 24   1    1  1   1   0
## 3235   3235    0 480 24   1    1  1   1   0
## 3236   3236    0 480 24   1    1  1   1   0
## 3237   3237    0 480 24   1    1  1   1   0
## 3238   3238    0 480 24   1    1  1   1   0
## 3239   3239    0 480 24   1    1  1   1   0
## 3240   3240    0 480 24   1    1  1   1   0
## 3241   3241    0 480 24   1    1  1   1   0
## 3242   3242    0 480 24   1    1  1   1   0
## 3243   3243    0 480 24   1    1  1   1   0
## 3244   3244    0 480 24   1    1  1   1   0
## 3245   3245    0 480 24   1    1  1   1   0
## 3246   3246    0 480 24   1    1  1   1   0
## 3247   3247    0 480 24   1    1  1   1   0
## 3248   3248    0 480 24   1    1  1   1   0
## 3249   3249    0 480 24   1    1  1   1   0
## 3250   3250    0 480 24   1    1  1   1   0
## 3251   3251    0 480 24   1    1  1   1   0
## 3252   3252    0 480 24   1    1  1   1   0
## 3253   3253    0 480 24   1    1  1   1   0
## 3254   3254    0 480 24   1    1  1   1   0
## 3255   3255    0 480 24   1    1  1   1   0
## 3256   3256    0 480 24   1    1  1   1   0
## 3257   3257    0 480 24   1    1  1   1   0
## 3258   3258    0 480 24   1    1  1   1   0
## 3259   3259    0 480 24   1    1  1   1   0
## 3260   3260    0 480 24   1    1  1   1   0
## 3261   3261    0 480 24   1    1  1   1   0
## 3262   3262    0 480 24   1    1  1   1   0
## 3263   3263    0 480 24   1    1  1   1   0
## 3264   3264    0 480 24   1    1  1   1   0
## 3265   3265    0 480 24   1    1  1   1   0
## 3266   3266    0 480 24   1    1  1   1   0
## 3267   3267    0 480 24   1    1  1   1   0
## 3268   3268    0 480 24   1    1  1   1   0
## 3269   3269    0 480 24   1    1  1   1   0
## 3270   3270    0 480 24   1    1  1   1   0
## 3271   3271    0 480 24   1    1  1   1   0
## 3272   3272    0 480 24   1    1  1   1   0
## 3273   3273    0 480 24   1    1  1   1   0
## 3274   3274    0 480 24   1    1  1   1   0
## 3275   3275    0 480 24   1    1  1   1   0
## 3276   3276    0 480 24   1    1  1   1   0
## 3277   3277    0 480 24   1    1  1   1   0
## 3278   3278    0 480 24   1    1  1   1   0
## 3279   3279    0 480 24   1    1  1   1   0
## 3280   3280    0 480 24   1    1  1   1   0
## 3281   3281    0 480 24   1    1  1   1   0
## 3282   3282    0 480 24   1    1  1   1   0
## 3283   3283    0 480 24   1    1  1   1   0
## 3284   3284    0 480 24   1    1  1   1   0
## 3285   3285    0 480 24   1    1  1   1   0
## 3286   3286    0 480 24   1    1  1   1   0
## 3287   3287    0 480 24   1    1  1   1   0
## 3288   3288    0 480 24   1    1  1   1   0
## 3289   3289    0 480 24   1    1  1   1   0
## 3290   3290    0 480 24   1    1  1   1   0
## 3291   3291    0 480 24   1    1  1   1   0
## 3292   3292    0 480 24   1    1  1   1   0
## 3293   3293    0 480 24   1    1  1   1   0
## 3294   3294    0 480 24   1    1  1   1   0
## 3295   3295    0 480 24   1    1  1   1   0
## 3296   3296    0 480 24   1    1  1   1   0
## 3297   3297    0 480 24   1    1  1   1   0
## 3298   3298    0 480 24   1    1  1   1   0
## 3299   3299    0 480 24   1    1  1   1   0
## 3300   3300    0 480 24   1    1  1   1   0
## 3301   3301    0 480 24   1    1  1   1   0
## 3302   3302    0 480 24   1    1  1   1   0
## 3303   3303    0 480 24   1    1  1   1   0
## 3304   3304    0 480 24   1    1  1   1   0
## 3305   3305    0 480 24   1    1  1   1   0
## 3306   3306    0 480 24   1    1  1   1   0
## 3307   3307    0 480 24   1    1  1   1   0
## 3308   3308    0 480 24   1    1  1   1   0
## 3309   3309    0 480 24   1    1  1   1   0
## 3310   3310    0 480 24   1    1  1   1   0
## 3311   3311    0 480 24   1    1  1   1   0
## 3312   3312    0 480 24   1    1  1   1   0
## 3313   3313    0 480 24   1    1  1   1   0
## 3314   3314    0 480 24   1    1  1   1   0
## 3315   3315    0 480 24   1    1  1   1   0
## 3316   3316    0 480 24   1    1  1   1   0
## 3317   3317    0 480 24   1    1  1   1   0
## 3318   3318    0 480 24   1    1  1   1   0
## 3319   3319    0 480 24   1    1  1   1   0
## 3320   3320    0 480 24   1    1  1   1   0
## 3321   3321    0 480 24   1    1  1   1   0
## 3322   3322    0 480 24   1    1  1   1   0
## 3323   3323    0 480 24   1    1  1   1   0
## 3324   3324    0 480 24   1    1  1   1   0
## 3325   3325    0 480 24   1    1  1   1   0
## 3326   3326    0 480 24   1    1  1   1   0
## 3327   3327    0 480 24   1    1  1   1   0
## 3328   3328    0 480 24   1    1  1   1   0
## 3329   3329    0 480 24   1    1  1   1   0
## 3330   3330    0 480 24   1    1  1   1   0
## 3331   3331    0 480 24   1    1  1   1   0
## 3332   3332    0 480 24   1    1  1   1   0
## 3333   3333    0 480 24   1    1  1   1   0
## 3334   3334    0 480 24   1    1  1   1   0
## 3335   3335    0 480 24   1    1  1   1   0
## 3336   3336    0 480 24   1    1  1   1   0
## 3337   3337    0 480 24   1    1  1   1   0
## 3338   3338    0 480 24   1    1  1   1   0
## 3339   3339    0 480 24   1    1  1   1   0
## 3340   3340    0 480 24   1    1  1   1   0
## 3341   3341    0 480 24   1    1  1   1   0
## 3342   3342    0 480 24   1    1  1   1   0
## 3343   3343    0 480 24   1    1  1   1   0
## 3344   3344    0 480 24   1    1  1   1   0
## 3345   3345    0 480 24   1    1  1   1   0
## 3346   3346    0 480 24   1    1  1   1   0
## 3347   3347    0 480 24   1    1  1   1   0
## 3348   3348    0 480 24   1    1  1   1   0
## 3349   3349    0 480 24   1    1  1   1   0
## 3350   3350    0 480 24   1    1  1   1   0
## 3351   3351    0 480 24   1    1  1   1   0
## 3352   3352    0 480 24   1    1  1   1   0
## 3353   3353    0 480 24   1    1  1   1   0
## 3354   3354    0 480 24   1    1  1   1   0
## 3355   3355    0 480 24   1    1  1   1   0
## 3356   3356    0 480 24   1    1  1   1   0
## 3357   3357    0 480 24   1    1  1   1   0
## 3358   3358    0 480 24   1    1  1   1   0
## 3359   3359    0 480 24   1    1  1   1   0
## 3360   3360    0 480 24   1    1  1   1   0
## 3361   3361    0 480 24   1    1  1   1   0
## 3362   3362    0 480 24   1    1  1   1   0
## 3363   3363    0 480 24   1    1  1   1   0
## 3364   3364    0 480 24   1    1  1   1   0
## 3365   3365    0 480 24   1    1  1   1   0
## 3366   3366    0 480 24   1    1  1   1   0
## 3367   3367    0 480 24   1    1  1   1   0
## 3368   3368    0 480 24   1    1  1   1   0
## 3369   3369    0 480 24   1    1  1   1   0
## 3370   3370    0 480 24   1    1  1   1   0
## 3371   3371    0 480 24   1    1  1   1   0
## 3372   3372    0 480 24   1    1  1   1   0
## 3373   3373    0 480 24   1    1  1   1   0
## 3374   3374    0 480 24   1    1  1   1   0
## 3375   3375    0 480 24   1    1  1   1   0
## 3376   3376    0 480 24   1    1  1   1   0
## 3377   3377    0 480 24   1    1  1   1   0
## 3378   3378    0 480 24   1    1  1   1   0
## 3379   3379    0 480 24   1    1  1   1   0
## 3380   3380    0 480 24   1    1  1   1   0
## 3381   3381    0 480 24   1    1  1   1   0
## 3382   3382    0 480 24   1    1  1   1   0
## 3383   3383    0 480 24   1    1  1   1   0
## 3384   3384    0 480 24   1    1  1   1   0
## 3385   3385    0 480 24   1    1  1   1   0
## 3386   3386    0 480 24   1    1  1   1   0
## 3387   3387    0 480 24   1    1  1   1   0
## 3388   3388    0 480 24   1    1  1   1   0
## 3389   3389    0 480 24   1    1  1   1   0
## 3390   3390    0 480 24   1    1  1   1   0
## 3391   3391    0 480 24   1    1  1   1   0
## 3392   3392    0 480 24   1    1  1   1   0
## 3393   3393    0 480 24   1    1  1   1   0
## 3394   3394    0 480 24   1    1  1   1   0
## 3395   3395    0 480 24   1    1  1   1   0
## 3396   3396    0 480 24   1    1  1   1   0
## 3397   3397    0 480 24   1    1  1   1   0
## 3398   3398    0 480 24   1    1  1   1   0
## 3399   3399    0 480 24   1    1  1   1   0
## 3400   3400    0 480 24   1    1  1   1   0
## 3401   3401    0 480 24   1    1  1   1   0
## 3402   3402    0 480 24   1    1  1   1   0
## 3403   3403    0 480 24   1    1  1   1   0
## 3404   3404    0 480 24   1    1  1   1   0
## 3405   3405    0 480 24   1    1  1   1   0
## 3406   3406    0 480 24   1    1  1   1   0
## 3407   3407    0 480 24   1    1  1   1   0
## 3408   3408    0 480 24   1    1  1   1   0
## 3409   3409    0 480 24   1    1  1   1   0
## 3410   3410    0 480 24   1    1  1   1   0
## 3411   3411    0 480 24   1    1  1   1   0
## 3412   3412    0 480 24   1    1  1   1   0
## 3413   3413    0 480 24   1    1  1   1   0
## 3414   3414    0 480 24   1    1  1   1   0
## 3415   3415    0 480 24   1    1  1   1   0
## 3416   3416    0 480 24   1    1  1   1   0
## 3417   3417    0 480 24   1    1  1   1   0
## 3418   3418    0 480 24   1    1  1   1   0
## 3419   3419    0 480 24   1    1  1   1   0
## 3420   3420    0 480 24   1    1  1   1   0
## 3421   3421    0 480 24   1    1  1   1   0
## 3422   3422    0 480 24   1    1  1   1   0
## 3423   3423    0 480 24   1    1  1   1   0
## 3424   3424    0 480 24   1    1  1   1   0
## 3425   3425    0 480 24   1    1  1   1   0
## 3426   3426    0 480 24   1    1  1   1   0
## 3427   3427    0 480 24   1    1  1   1   0
## 3428   3428    0 480 24   1    1  1   1   0
## 3429   3429    0 480 24   1    1  1   1   0
## 3430   3430    0 480 24   1    1  1   1   0
## 3431   3431    0 480 24   1    1  1   1   0
## 3432   3432    0 480 24   1    1  1   1   0
## 3433   3433    0 480 24   1    1  1   1   0
## 3434   3434    0 480 24   1    1  1   1   0
## 3435   3435    0 480 24   1    1  1   1   0
## 3436   3436    0 480 24   1    1  1   1   0
## 3437   3437    0 480 24   1    1  1   1   0
## 3438   3438    0 480 24   1    1  1   1   0
## 3439   3439    0 480 24   1    1  1   1   0
## 3440   3440    0 480 24   1    1  1   1   0
## 3441   3441    0 480 24   1    1  1   1   0
## 3442   3442    0 480 24   1    1  1   1   0
## 3443   3443    0 480 24   1    1  1   1   0
## 3444   3444    0 480 24   1    1  1   1   0
## 3445   3445    0 480 24   1    1  1   1   0
## 3446   3446    0 480 24   1    1  1   1   0
## 3447   3447    0 480 24   1    1  1   1   0
## 3448   3448    0 480 24   1    1  1   1   0
## 3449   3449    0 480 24   1    1  1   1   0
## 3450   3450    0 480 24   1    1  1   1   0
## 3451   3451    0 480 24   1    1  1   1   0
## 3452   3452    0 480 24   1    1  1   1   0
## 3453   3453    0 480 24   1    1  1   1   0
## 3454   3454    0 480 24   1    1  1   1   0
## 3455   3455    0 480 24   1    1  1   1   0
## 3456   3456    0 480 24   1    1  1   1   0
## 3457   3457    0 480 24   1    1  1   1   0
## 3458   3458    0 480 24   1    1  1   1   0
## 3459   3459    0 480 24   1    1  1   1   0
## 3460   3460    0 480 24   1    1  1   1   0
## 3461   3461    0 480 24   1    1  1   1   0
## 3462   3462    0 480 24   1    1  1   1   0
## 3463   3463    0 480 24   1    1  1   1   0
## 3464   3464    0 480 24   1    1  1   1   0
## 3465   3465    0 480 24   1    1  1   1   0
## 3466   3466    0 480 24   1    1  1   1   0
## 3467   3467    0 480 24   1    1  1   1   0
## 3468   3468    0 480 24   1    1  1   1   0
## 3469   3469    0 480 24   1    1  1   1   0
## 3470   3470    0 480 24   1    1  1   1   0
## 3471   3471    0 480 24   1    1  1   1   0
## 3472   3472    0 480 24   1    1  1   1   0
## 3473   3473    0 480 24   1    1  1   1   0
## 3474   3474    0 480 24   1    1  1   1   0
## 3475   3475    0 480 24   1    1  1   1   0
## 3476   3476    0 480 24   1    1  1   1   0
## 3477   3477    0 480 24   1    1  1   1   0
## 3478   3478    0 480 24   1    1  1   1   0
## 3479   3479    0 480 24   1    1  1   1   0
## 3480   3480    0 480 24   1    1  1   1   0
## 3481   3481    0 480 24   1    1  1   1   0
## 3482   3482    0 480 24   1    1  1   1   0
## 3483   3483    0 480 24   1    1  1   1   0
## 3484   3484    0 480 24   1    1  1   1   0
## 3485   3485    0 480 24   1    1  1   1   0
## 3486   3486    0 480 24   1    1  1   1   0
## 3487   3487    0 480 24   1    1  1   1   0
## 3488   3488    0 480 24   1    1  1   1   0
## 3489   3489    0 480 24   1    1  1   1   0
## 3490   3490    0 480 24   1    1  1   1   0
## 3491   3491    0 480 24   1    1  1   1   0
## 3492   3492    0 480 24   1    1  1   1   0
## 3493   3493    0 480 24   1    1  1   1   0
## 3494   3494    0 480 24   1    1  1   1   0
## 3495   3495    0 480 24   1    1  1   1   0
## 3496   3496    0 480 24   1    1  1   1   0
## 3497   3497    0 480 24   1    1  1   1   0
## 3498   3498    0 480 24   1    1  1   1   0
## 3499   3499    0 480 24   1    1  1   1   0
## 3500   3500    0 480 24   1    1  1   1   0
## 3501   3501    0 480 24   1    1  1   1   0
## 3502   3502    0 480 24   1    1  1   1   0
## 3503   3503    0 480 24   1    1  1   1   0
## 3504   3504    0 480 24   1    1  1   1   0
## 3505   3505    0 480 24   1    1  1   1   0
## 3506   3506    0 480 24   1    1  1   1   0
## 3507   3507    0 480 24   1    1  1   1   0
## 3508   3508    0 480 24   1    1  1   1   0
## 3509   3509    0 480 24   1    1  1   1   0
## 3510   3510    0 480 24   1    1  1   1   0
## 3511   3511    0 480 24   1    1  1   1   0
## 3512   3512    0 480 24   1    1  1   1   0
## 3513   3513    0 480 24   1    1  1   1   0
## 3514   3514    0 480 24   1    1  1   1   0
## 3515   3515    0 480 24   1    1  1   1   0
## 3516   3516    0 480 24   1    1  1   1   0
## 3517   3517    0 480 24   1    1  1   1   0
## 3518   3518    0 480 24   1    1  1   1   0
## 3519   3519    0 480 24   1    1  1   1   0
## 3520   3520    0 480 24   1    1  1   1   0
## 3521   3521    0 480 24   1    1  1   1   0
## 3522   3522    0 480 24   1    1  1   1   0
## 3523   3523    0 480 24   1    1  1   1   0
## 3524   3524    0 480 24   1    1  1   1   0
## 3525   3525    0 480 24   1    1  1   1   0
## 3526   3526    0 480 24   1    1  1   1   0
## 3527   3527    0 480 24   1    1  1   1   0
## 3528   3528    0 480 24   1    1  1   1   0
## 3529   3529    0 480 24   1    1  1   1   0
## 3530   3530    0 480 24   1    1  1   1   0
## 3531   3531    0 480 24   1    1  1   1   0
## 3532   3532    0 480 24   1    1  1   1   0
## 3533   3533    0 480 24   1    1  1   1   0
## 3534   3534    0 480 24   1    1  1   1   0
## 3535   3535    0 480 24   1    1  1   1   0
## 3536   3536    0 480 24   1    1  1   1   0
## 3537   3537    0 480 24   1    1  1   1   0
## 3538   3538    0 480 24   1    1  1   1   0
## 3539   3539    0 480 24   1    1  1   1   0
## 3540   3540    0 480 24   1    1  1   1   0
## 3541   3541    0 480 24   1    1  1   1   0
## 3542   3542    0 480 24   1    1  1   1   0
## 3543   3543    0 480 24   1    1  1   1   0
## 3544   3544    0 480 24   1    1  1   1   0
## 3545   3545    0 480 24   1    1  1   1   0
## 3546   3546    0 480 24   1    1  1   1   0
## 3547   3547    0 480 24   1    1  1   1   0
## 3548   3548    0 480 24   1    1  1   1   0
## 3549   3549    0 480 24   1    1  1   1   0
## 3550   3550    0 480 24   1    1  1   1   0
## 3551   3551    0 480 24   1    1  1   1   0
## 3552   3552    0 480 24   1    1  1   1   0
## 3553   3553    0 480 24   1    1  1   1   0
## 3554   3554    0 480 24   1    1  1   1   0
## 3555   3555    0 480 24   1    1  1   1   0
## 3556   3556    0 480 24   1    1  1   1   0
## 3557   3557    0 480 24   1    1  1   1   0
## 3558   3558    0 480 24   1    1  1   1   0
## 3559   3559    0 480 24   1    1  1   1   0
## 3560   3560    0 480 24   1    1  1   1   0
## 3561   3561    0 480 24   1    1  1   1   0
## 3562   3562    0 480 24   1    1  1   1   0
## 3563   3563    0 480 24   1    1  1   1   0
## 3564   3564    0 480 24   1    1  1   1   0
## 3565   3565    0 480 24   1    1  1   1   0
## 3566   3566    0 480 24   1    1  1   1   0
## 3567   3567    0 480 24   1    1  1   1   0
## 3568   3568    0 480 24   1    1  1   1   0
## 3569   3569    0 480 24   1    1  1   1   0
## 3570   3570    0 480 24   1    1  1   1   0
## 3571   3571    0 480 24   1    1  1   1   0
## 3572   3572    0 480 24   1    1  1   1   0
## 3573   3573    0 480 24   1    1  1   1   0
## 3574   3574    0 480 24   1    1  1   1   0
## 3575   3575    0 480 24   1    1  1   1   0
## 3576   3576    0 480 24   1    1  1   1   0
## 3577   3577    0 480 24   1    1  1   1   0
## 3578   3578    0 480 24   1    1  1   1   0
## 3579   3579    0 480 24   1    1  1   1   0
## 3580   3580    0 480 24   1    1  1   1   0
## 3581   3581    0 480 24   1    1  1   1   0
## 3582   3582    0 480 24   1    1  1   1   0
## 3583   3583    0 480 24   1    1  1   1   0
## 3584   3584    0 480 24   1    1  1   1   0
## 3585   3585    0 480 24   1    1  1   1   0
## 3586   3586    0 480 24   1    1  1   1   0
## 3587   3587    0 480 24   1    1  1   1   0
## 3588   3588    0 480 24   1    1  1   1   0
## 3589   3589    0 480 24   1    1  1   1   0
## 3590   3590    0 480 24   1    1  1   1   0
## 3591   3591    0 480 24   1    1  1   1   0
## 3592   3592    0 480 24   1    1  1   1   0
## 3593   3593    0 480 24   1    1  1   1   0
## 3594   3594    0 480 24   1    1  1   1   0
## 3595   3595    0 480 24   1    1  1   1   0
## 3596   3596    0 480 24   1    1  1   1   0
## 3597   3597    0 480 24   1    1  1   1   0
## 3598   3598    0 480 24   1    1  1   1   0
## 3599   3599    0 480 24   1    1  1   1   0
## 3600   3600    0 480 24   1    1  1   1   0
## 3601   3601    0 480 24   1    1  1   1   0
## 3602   3602    0 480 24   1    1  1   1   0
## 3603   3603    0 480 24   1    1  1   1   0
## 3604   3604    0 480 24   1    1  1   1   0
## 3605   3605    0 480 24   1    1  1   1   0
## 3606   3606    0 480 24   1    1  1   1   0
## 3607   3607    0 480 24   1    1  1   1   0
## 3608   3608    0 480 24   1    1  1   1   0
## 3609   3609    0 480 24   1    1  1   1   0
## 3610   3610    0 480 24   1    1  1   1   0
## 3611   3611    0 480 24   1    1  1   1   0
## 3612   3612    0 480 24   1    1  1   1   0
## 3613   3613    0 480 24   1    1  1   1   0
## 3614   3614    0 480 24   1    1  1   1   0
## 3615   3615    0 480 24   1    1  1   1   0
## 3616   3616    0 480 24   1    1  1   1   0
## 3617   3617    0 480 24   1    1  1   1   0
## 3618   3618    0 480 24   1    1  1   1   0
## 3619   3619    0 480 24   1    1  1   1   0
## 3620   3620    0 480 24   1    1  1   1   0
## 3621   3621    0 480 24   1    1  1   1   0
## 3622   3622    0 480 24   1    1  1   1   0
## 3623   3623    0 480 24   1    1  1   1   0
## 3624   3624    0 480 24   1    1  1   1   0
## 3625   3625    0 480 24   1    1  1   1   0
## 3626   3626    0 480 24   1    1  1   1   0
## 3627   3627    0 480 24   1    1  1   1   0
## 3628   3628    0 480 24   1    1  1   1   0
## 3629   3629    0 480 24   1    1  1   1   0
## 3630   3630    0 480 24   1    1  1   1   0
## 3631   3631    0 480 24   1    1  1   1   0
## 3632   3632    0 480 24   1    1  1   1   0
## 3633   3633    0 480 24   1    1  1   1   0
## 3634   3634    0 480 24   1    1  1   1   0
## 3635   3635    0 480 24   1    1  1   1   0
## 3636   3636    0 480 24   1    1  1   1   0
## 3637   3637    0 480 24   1    1  1   1   0
## 3638   3638    0 480 24   1    1  1   1   0
## 3639   3639    0 480 24   1    1  1   1   0
## 3640   3640    0 480 24   1    1  1   1   0
## 3641   3641    0 480 24   1    1  1   1   0
## 3642   3642    0 480 24   1    1  1   1   0
## 3643   3643    0 480 24   1    1  1   1   0
## 3644   3644    0 480 24   1    1  1   1   0
## 3645   3645    0 480 24   1    1  1   1   0
## 3646   3646    0 480 24   1    1  1   1   0
## 3647   3647    0 480 24   1    1  1   1   0
## 3648   3648    0 480 24   1    1  1   1   0
## 3649   3649    0 480 24   1    1  1   1   0
## 3650   3650    0 480 24   1    1  1   1   0
## 3651   3651    0 480 24   1    1  1   1   0
## 3652   3652    0 480 24   1    1  1   1   0
## 3653   3653    0 480 24   1    1  1   1   0
## 3654   3654    0 480 24   1    1  1   1   0
## 3655   3655    0 480 24   1    1  1   1   0
## 3656   3656    0 480 24   1    1  1   1   0
## 3657   3657    0 480 24   1    1  1   1   0
## 3658   3658    0 480 24   1    1  1   1   0
## 3659   3659    0 480 24   1    1  1   1   0
## 3660   3660    0 480 24   1    1  1   1   0
## 3661   3661    0 480 24   1    1  1   1   0
## 3662   3662    0 480 24   1    1  1   1   0
## 3663   3663    0 480 24   1    1  1   1   0
## 3664   3664    0 480 24   1    1  1   1   0
## 3665   3665    0 480 24   1    1  1   1   0
## 3666   3666    0 480 24   1    1  1   1   0
## 3667   3667    0 480 24   1    1  1   1   0
## 3668   3668    0 480 24   1    1  1   1   0
## 3669   3669    0 480 24   1    1  1   1   0
## 3670   3670    0 480 24   1    1  1   1   0
## 3671   3671    0 480 24   1    1  1   1   0
## 3672   3672    0 480 24   1    1  1   1   0
## 3673   3673    0 480 24   1    1  1   1   0
## 3674   3674    0 480 24   1    1  1   1   0
## 3675   3675    0 480 24   1    1  1   1   0
## 3676   3676    0 480 24   1    1  1   1   0
## 3677   3677    0 480 24   1    1  1   1   0
## 3678   3678    0 480 24   1    1  1   1   0
## 3679   3679    0 480 24   1    1  1   1   0
## 3680   3680    0 480 24   1    1  1   1   0
## 3681   3681    0 480 24   1    1  1   1   0
## 3682   3682    0 480 24   1    1  1   1   0
## 3683   3683    0 480 24   1    1  1   1   0
## 3684   3684    0 480 24   1    1  1   1   0
## 3685   3685    0 480 24   1    1  1   1   0
## 3686   3686    0 480 24   1    1  1   1   0
## 3687   3687    0 480 24   1    1  1   1   0
## 3688   3688    0 480 24   1    1  1   1   0
## 3689   3689    0 480 24   1    1  1   1   0
## 3690   3690    0 480 24   1    1  1   1   0
## 3691   3691    0 480 24   1    1  1   1   0
## 3692   3692    0 480 24   1    1  1   1   0
## 3693   3693    0 480 24   1    1  1   1   0
## 3694   3694    0 480 24   1    1  1   1   0
## 3695   3695    0 480 24   1    1  1   1   0
## 3696   3696    0 480 24   1    1  1   1   0
## 3697   3697    0 480 24   1    1  1   1   0
## 3698   3698    0 480 24   1    1  1   1   0
## 3699   3699    0 480 24   1    1  1   1   0
## 3700   3700    0 480 24   1    1  1   1   0
## 3701   3701    0 480 24   1    1  1   1   0
## 3702   3702    0 480 24   1    1  1   1   0
## 3703   3703    0 480 24   1    1  1   1   0
## 3704   3704    0 480 24   1    1  1   1   0
## 3705   3705    0 480 24   1    1  1   1   0
## 3706   3706    0 480 24   1    1  1   1   0
## 3707   3707    0 480 24   1    1  1   1   0
## 3708   3708    0 480 24   1    1  1   1   0
## 3709   3709    0 480 24   1    1  1   1   0
## 3710   3710    0 480 24   1    1  1   1   0
## 3711   3711    0 480 24   1    1  1   1   0
## 3712   3712    0 480 24   1    1  1   1   0
## 3713   3713    0 480 24   1    1  1   1   0
## 3714   3714    0 480 24   1    1  1   1   0
## 3715   3715    0 480 24   1    1  1   1   0
## 3716   3716    0 480 24   1    1  1   1   0
## 3717   3717    0 480 24   1    1  1   1   0
## 3718   3718    0 480 24   1    1  1   1   0
## 3719   3719    0 480 24   1    1  1   1   0
## 3720   3720    0 480 24   1    1  1   1   0
## 3721   3721    0 480 24   1    1  1   1   0
## 3722   3722    0 480 24   1    1  1   1   0
## 3723   3723    0 480 24   1    1  1   1   0
## 3724   3724    0 480 24   1    1  1   1   0
## 3725   3725    0 480 24   1    1  1   1   0
## 3726   3726    0 480 24   1    1  1   1   0
## 3727   3727    0 480 24   1    1  1   1   0
## 3728   3728    0 480 24   1    1  1   1   0
## 3729   3729    0 480 24   1    1  1   1   0
## 3730   3730    0 480 24   1    1  1   1   0
## 3731   3731    0 480 24   1    1  1   1   0
## 3732   3732    0 480 24   1    1  1   1   0
## 3733   3733    0 480 24   1    1  1   1   0
## 3734   3734    0 480 24   1    1  1   1   0
## 3735   3735    0 480 24   1    1  1   1   0
## 3736   3736    0 480 24   1    1  1   1   0
## 3737   3737    0 480 24   1    1  1   1   0
## 3738   3738    0 480 24   1    1  1   1   0
## 3739   3739    0 480 24   1    1  1   1   0
## 3740   3740    0 480 24   1    1  1   1   0
## 3741   3741    0 480 24   1    1  1   1   0
## 3742   3742    0 480 24   1    1  1   1   0
## 3743   3743    0 480 24   1    1  1   1   0
## 3744   3744    0 480 24   1    1  1   1   0
## 3745   3745    0 480 24   1    1  1   1   0
## 3746   3746    0 480 24   1    1  1   1   0
## 3747   3747    0 480 24   1    1  1   1   0
## 3748   3748    0 480 24   1    1  1   1   0
## 3749   3749    0 480 24   1    1  1   1   0
## 3750   3750    0 480 24   1    1  1   1   0
## 3751   3751    0 480 24   1    1  1   1   0
## 3752   3752    0 480 24   1    1  1   1   0
## 3753   3753    0 480 24   1    1  1   1   0
## 3754   3754    0 480 24   1    1  1   1   0
## 3755   3755    0 480 24   1    1  1   1   0
## 3756   3756    0 480 24   1    1  1   1   0
## 3757   3757    0 480 24   1    1  1   1   0
## 3758   3758    0 480 24   1    1  1   1   0
## 3759   3759    0 480 24   1    1  1   1   0
## 3760   3760    0 480 24   1    1  1   1   0
## 3761   3761    0 480 24   1    1  1   1   0
## 3762   3762    0 480 24   1    1  1   1   0
## 3763   3763    0 480 24   1    1  1   1   0
## 3764   3764    0 480 24   1    1  1   1   0
## 3765   3765    0 480 24   1    1  1   1   0
## 3766   3766    0 480 24   1    1  1   1   0
## 3767   3767    0 480 24   1    1  1   1   0
## 3768   3768    0 480 24   1    1  1   1   0
## 3769   3769    0 480 24   1    1  1   1   0
## 3770   3770    0 480 24   1    1  1   1   0
## 3771   3771    0 480 24   1    1  1   1   0
## 3772   3772    0 480 24   1    1  1   1   0
## 3773   3773    0 480 24   1    1  1   1   0
## 3774   3774    0 480 24   1    1  1   1   0
## 3775   3775    0 480 24   1    1  1   1   0
## 3776   3776    0 480 24   1    1  1   1   0
## 3777   3777    0 480 24   1    1  1   1   0
## 3778   3778    0 480 24   1    1  1   1   0
## 3779   3779    0 480 24   1    1  1   1   0
## 3780   3780    0 480 24   1    1  1   1   0
## 3781   3781    0 480 24   1    1  1   1   0
## 3782   3782    0 480 24   1    1  1   1   0
## 3783   3783    0 480 24   1    1  1   1   0
## 3784   3784    0 480 24   1    1  1   1   0
## 3785   3785    0 480 24   1    1  1   1   0
## 3786   3786    0 480 24   1    1  1   1   0
## 3787   3787    0 480 24   1    1  1   1   0
## 3788   3788    0 480 24   1    1  1   1   0
## 3789   3789    0 480 24   1    1  1   1   0
## 3790   3790    0 480 24   1    1  1   1   0
## 3791   3791    0 480 24   1    1  1   1   0
## 3792   3792    0 480 24   1    1  1   1   0
## 3793   3793    0 480 24   1    1  1   1   0
## 3794   3794    0 480 24   1    1  1   1   0
## 3795   3795    0 480 24   1    1  1   1   0
## 3796   3796    0 480 24   1    1  1   1   0
## 3797   3797    0 480 24   1    1  1   1   0
## 3798   3798    0 480 24   1    1  1   1   0
## 3799   3799    0 480 24   1    1  1   1   0
## 3800   3800    0 480 24   1    1  1   1   0
## 3801   3801    0 480 24   1    1  1   1   0
## 3802   3802    0 480 24   1    1  1   1   0
## 3803   3803    0 480 24   1    1  1   1   0
## 3804   3804    0 480 24   1    1  1   1   0
## 3805   3805    0 480 24   1    1  1   1   0
## 3806   3806    0 480 24   1    1  1   1   0
## 3807   3807    0 480 24   1    1  1   1   0
## 3808   3808    0 480 24   1    1  1   1   0
## 3809   3809    0 480 24   1    1  1   1   0
## 3810   3810    0 480 24   1    1  1   1   0
## 3811   3811    0 480 24   1    1  1   1   0
## 3812   3812    0 480 24   1    1  1   1   0
## 3813   3813    0 480 24   1    1  1   1   0
## 3814   3814    0 480 24   1    1  1   1   0
## 3815   3815    0 480 24   1    1  1   1   0
## 3816   3816    0 480 24   1    1  1   1   0
## 3817   3817    0 480 24   1    1  1   1   0
## 3818   3818    0 480 24   1    1  1   1   0
## 3819   3819    0 480 24   1    1  1   1   0
## 3820   3820    0 480 24   1    1  1   1   0
## 3821   3821    0 480 24   1    1  1   1   0
## 3822   3822    0 480 24   1    1  1   1   0
## 3823   3823    0 480 24   1    1  1   1   0
## 3824   3824    0 480 24   1    1  1   1   0
## 3825   3825    0 480 24   1    1  1   1   0
## 3826   3826    0 480 24   1    1  1   1   0
## 3827   3827    0 480 24   1    1  1   1   0
## 3828   3828    0 480 24   1    1  1   1   0
## 3829   3829    0 480 24   1    1  1   1   0
## 3830   3830    0 480 24   1    1  1   1   0
## 3831   3831    0 480 24   1    1  1   1   0
## 3832   3832    0 480 24   1    1  1   1   0
## 3833   3833    0 480 24   1    1  1   1   0
## 3834   3834    0 480 24   1    1  1   1   0
## 3835   3835    0 480 24   1    1  1   1   0
## 3836   3836    0 480 24   1    1  1   1   0
## 3837   3837    0 480 24   1    1  1   1   0
## 3838   3838    0 480 24   1    1  1   1   0
## 3839   3839    0 480 24   1    1  1   1   0
## 3840   3840    0 480 24   1    1  1   1   0
## 3841   3841    0 480 24   1    1  1   1   0
## 3842   3842    0 480 24   1    1  1   1   0
## 3843   3843    0 480 24   1    1  1   1   0
## 3844   3844    0 480 24   1    1  1   1   0
## 3845   3845    0 480 24   1    1  1   1   0
## 3846   3846    0 480 24   1    1  1   1   0
## 3847   3847    0 480 24   1    1  1   1   0
## 3848   3848    0 480 24   1    1  1   1   0
## 3849   3849    0 480 24   1    1  1   1   0
## 3850   3850    0 480 24   1    1  1   1   0
## 3851   3851    0 480 24   1    1  1   1   0
## 3852   3852    0 480 24   1    1  1   1   0
## 3853   3853    0 480 24   1    1  1   1   0
## 3854   3854    0 480 24   1    1  1   1   0
## 3855   3855    0 480 24   1    1  1   1   0
## 3856   3856    0 480 24   1    1  1   1   0
## 3857   3857    0 480 24   1    1  1   1   0
## 3858   3858    0 480 24   1    1  1   1   0
## 3859   3859    0 480 24   1    1  1   1   0
## 3860   3860    0 480 24   1    1  1   1   0
## 3861   3861    0 480 24   1    1  1   1   0
## 3862   3862    0 480 24   1    1  1   1   0
## 3863   3863    0 480 24   1    1  1   1   0
## 3864   3864    0 480 24   1    1  1   1   0
## 3865   3865    0 480 24   1    1  1   1   0
## 3866   3866    0 480 24   1    1  1   1   0
## 3867   3867    0 480 24   1    1  1   1   0
## 3868   3868    0 480 24   1    1  1   1   0
## 3869   3869    0 480 24   1    1  1   1   0
## 3870   3870    0 480 24   1    1  1   1   0
## 3871   3871    0 480 24   1    1  1   1   0
## 3872   3872    0 480 24   1    1  1   1   0
## 3873   3873    0 480 24   1    1  1   1   0
## 3874   3874    0 480 24   1    1  1   1   0
## 3875   3875    0 480 24   1    1  1   1   0
## 3876   3876    0 480 24   1    1  1   1   0
## 3877   3877    0 480 24   1    1  1   1   0
## 3878   3878    0 480 24   1    1  1   1   0
## 3879   3879    0 480 24   1    1  1   1   0
## 3880   3880    0 480 24   1    1  1   1   0
## 3881   3881    0 480 24   1    1  1   1   0
## 3882   3882    0 480 24   1    1  1   1   0
## 3883   3883    0 480 24   1    1  1   1   0
## 3884   3884    0 480 24   1    1  1   1   0
## 3885   3885    0 480 24   1    1  1   1   0
## 3886   3886    0 480 24   1    1  1   1   0
## 3887   3887    0 480 24   1    1  1   1   0
## 3888   3888    0 480 24   1    1  1   1   0
## 3889   3889    0 480 24   1    1  1   1   0
## 3890   3890    0 480 24   1    1  1   1   0
## 3891   3891    0 480 24   1    1  1   1   0
## 3892   3892    0 480 24   1    1  1   1   0
## 3893   3893    0 480 24   1    1  1   1   0
## 3894   3894    0 480 24   1    1  1   1   0
## 3895   3895    0 480 24   1    1  1   1   0
## 3896   3896    0 480 24   1    1  1   1   0
## 3897   3897    0 480 24   1    1  1   1   0
## 3898   3898    0 480 24   1    1  1   1   0
## 3899   3899    0 480 24   1    1  1   1   0
## 3900   3900    0 480 24   1    1  1   1   0
## 3901   3901    0 480 24   1    1  1   1   0
## 3902   3902    0 480 24   1    1  1   1   0
## 3903   3903    0 480 24   1    1  1   1   0
## 3904   3904    0 480 24   1    1  1   1   0
## 3905   3905    0 480 24   1    1  1   1   0
## 3906   3906    0 480 24   1    1  1   1   0
## 3907   3907    0 480 24   1    1  1   1   0
## 3908   3908    0 480 24   1    1  1   1   0
## 3909   3909    0 480 24   1    1  1   1   0
## 3910   3910    0 480 24   1    1  1   1   0
## 3911   3911    0 480 24   1    1  1   1   0
## 3912   3912    0 480 24   1    1  1   1   0
## 3913   3913    0 480 24   1    1  1   1   0
## 3914   3914    0 480 24   1    1  1   1   0
## 3915   3915    0 480 24   1    1  1   1   0
## 3916   3916    0 480 24   1    1  1   1   0
## 3917   3917    0 480 24   1    1  1   1   0
## 3918   3918    0 480 24   1    1  1   1   0
## 3919   3919    0 480 24   1    1  1   1   0
## 3920   3920    0 480 24   1    1  1   1   0
## 3921   3921    0 480 24   1    1  1   1   0
## 3922   3922    0 480 24   1    1  1   1   0
## 3923   3923    0 480 24   1    1  1   1   0
## 3924   3924    0 480 24   1    1  1   1   0
## 3925   3925    0 480 24   1    1  1   1   0
## 3926   3926    0 480 24   1    1  1   1   0
## 3927   3927    0 480 24   1    1  1   1   0
## 3928   3928    0 480 24   1    1  1   1   0
## 3929   3929    0 480 24   1    1  1   1   0
## 3930   3930    0 480 24   1    1  1   1   0
## 3931   3931    0 480 24   1    1  1   1   0
## 3932   3932    0 480 24   1    1  1   1   0
## 3933   3933    0 480 24   1    1  1   1   0
## 3934   3934    0 480 24   1    1  1   1   0
## 3935   3935    0 480 24   1    1  1   1   0
## 3936   3936    0 480 24   1    1  1   1   0
## 3937   3937    0 480 24   1    1  1   1   0
## 3938   3938    0 480 24   1    1  1   1   0
## 3939   3939    0 480 24   1    1  1   1   0
## 3940   3940    0 480 24   1    1  1   1   0
## 3941   3941    0 480 24   1    1  1   1   0
## 3942   3942    0 480 24   1    1  1   1   0
## 3943   3943    0 480 24   1    1  1   1   0
## 3944   3944    0 480 24   1    1  1   1   0
## 3945   3945    0 480 24   1    1  1   1   0
## 3946   3946    0 480 24   1    1  1   1   0
## 3947   3947    0 480 24   1    1  1   1   0
## 3948   3948    0 480 24   1    1  1   1   0
## 3949   3949    0 480 24   1    1  1   1   0
## 3950   3950    0 480 24   1    1  1   1   0
## 3951   3951    0 480 24   1    1  1   1   0
## 3952   3952    0 480 24   1    1  1   1   0
## 3953   3953    0 480 24   1    1  1   1   0
## 3954   3954    0 480 24   1    1  1   1   0
## 3955   3955    0 480 24   1    1  1   1   0
## 3956   3956    0 480 24   1    1  1   1   0
## 3957   3957    0 480 24   1    1  1   1   0
## 3958   3958    0 480 24   1    1  1   1   0
## 3959   3959    0 480 24   1    1  1   1   0
## 3960   3960    0 480 24   1    1  1   1   0
## 3961   3961    0 480 24   1    1  1   1   0
## 3962   3962    0 480 24   1    1  1   1   0
## 3963   3963    0 480 24   1    1  1   1   0
## 3964   3964    0 480 24   1    1  1   1   0
## 3965   3965    0 480 24   1    1  1   1   0
## 3966   3966    0 480 24   1    1  1   1   0
## 3967   3967    0 480 24   1    1  1   1   0
## 3968   3968    0 480 24   1    1  1   1   0
## 3969   3969    0 480 24   1    1  1   1   0
## 3970   3970    0 480 24   1    1  1   1   0
## 3971   3971    0 480 24   1    1  1   1   0
## 3972   3972    0 480 24   1    1  1   1   0
## 3973   3973    0 480 24   1    1  1   1   0
## 3974   3974    0 480 24   1    1  1   1   0
## 3975   3975    0 480 24   1    1  1   1   0
## 3976   3976    0 480 24   1    1  1   1   0
## 3977   3977    0 480 24   1    1  1   1   0
## 3978   3978    0 480 24   1    1  1   1   0
## 3979   3979    0 480 24   1    1  1   1   0
## 3980   3980    0 480 24   1    1  1   1   0
## 3981   3981    0 480 24   1    1  1   1   0
## 3982   3982    0 480 24   1    1  1   1   0
## 3983   3983    0 480 24   1    1  1   1   0
## 3984   3984    0 480 24   1    1  1   1   0
## 3985   3985    0 480 24   1    1  1   1   0
## 3986   3986    0 480 24   1    1  1   1   0
## 3987   3987    0 480 24   1    1  1   1   0
## 3988   3988    0 480 24   1    1  1   1   0
## 3989   3989    0 480 24   1    1  1   1   0
## 3990   3990    0 480 24   1    1  1   1   0
## 3991   3991    0 480 24   1    1  1   1   0
## 3992   3992    0 480 24   1    1  1   1   0
## 3993   3993    0 480 24   1    1  1   1   0
## 3994   3994    0 480 24   1    1  1   1   0
## 3995   3995    0 480 24   1    1  1   1   0
## 3996   3996    0 480 24   1    1  1   1   0
## 3997   3997    0 480 24   1    1  1   1   0
## 3998   3998    0 480 24   1    1  1   1   0
## 3999   3999    0 480 24   1    1  1   1   0
## 4000   4000    0 480 24   1    1  1   1   0
## 4001   4001    0 480 24   1    1  1   1   0
## 4002   4002    0 480 24   1    1  1   1   0
## 4003   4003    0 480 24   1    1  1   1   0
## 4004   4004    0 480 24   1    1  1   1   0
## 4005   4005    0 480 24   1    1  1   1   0
## 4006   4006    0 480 24   1    1  1   1   0
## 4007   4007    0 480 24   1    1  1   1   0
## 4008   4008    0 480 24   1    1  1   1   0
## 4009   4009    0 480 24   1    1  1   1   0
## 4010   4010    0 480 24   1    1  1   1   0
## 4011   4011    0 480 24   1    1  1   1   0
## 4012   4012    0 480 24   1    1  1   1   0
## 4013   4013    0 480 24   1    1  1   1   0
## 4014   4014    0 480 24   1    1  1   1   0
## 4015   4015    0 480 24   1    1  1   1   0
## 4016   4016    0 480 24   1    1  1   1   0
## 4017   4017    0 480 24   1    1  1   1   0
## 4018   4018    0 480 24   1    1  1   1   0
## 4019   4019    0 480 24   1    1  1   1   0
## 4020   4020    0 480 24   1    1  1   1   0
## 4021   4021    0 480 24   1    1  1   1   0
## 4022   4022    0 480 24   1    1  1   1   0
## 4023   4023    0 480 24   1    1  1   1   0
## 4024   4024    0 480 24   1    1  1   1   0
## 4025   4025    0 480 24   1    1  1   1   0
## 4026   4026    0 480 24   1    1  1   1   0
## 4027   4027    0 480 24   1    1  1   1   0
## 4028   4028    0 480 24   1    1  1   1   0
## 4029   4029    0 480 24   1    1  1   1   0
## 4030   4030    0 480 24   1    1  1   1   0
## 4031   4031    0 480 24   1    1  1   1   0
## 4032   4032    0 480 24   1    1  1   1   0
## 4033   4033    0 480 24   1    1  1   1   0
## 4034   4034    0 480 24   1    1  1   1   0
## 4035   4035    0 480 24   1    1  1   1   0
## 4036   4036    0 480 24   1    1  1   1   0
## 4037   4037    0 480 24   1    1  1   1   0
## 4038   4038    0 480 24   1    1  1   1   0
## 4039   4039    0 480 24   1    1  1   1   0
## 4040   4040    0 480 24   1    1  1   1   0
## 4041   4041    0 480 24   1    1  1   1   0
## 4042   4042    0 480 24   1    1  1   1   0
## 4043   4043    0 480 24   1    1  1   1   0
## 4044   4044    0 480 24   1    1  1   1   0
## 4045   4045    0 480 24   1    1  1   1   0
## 4046   4046    0 480 24   1    1  1   1   0
## 4047   4047    0 480 24   1    1  1   1   0
## 4048   4048    0 480 24   1    1  1   1   0
## 4049   4049    0 480 24   1    1  1   1   0
## 4050   4050    0 480 24   1    1  1   1   0
## 4051   4051    0 480 24   1    1  1   1   0
## 4052   4052    0 480 24   1    1  1   1   0
## 4053   4053    0 480 24   1    1  1   1   0
## 4054   4054    0 480 24   1    1  1   1   0
## 4055   4055    0 480 24   1    1  1   1   0
## 4056   4056    0 480 24   1    1  1   1   0
## 4057   4057    0 480 24   1    1  1   1   0
## 4058   4058    0 480 24   1    1  1   1   0
## 4059   4059    0 480 24   1    1  1   1   0
## 4060   4060    0 480 24   1    1  1   1   0
## 4061   4061    0 480 24   1    1  1   1   0
## 4062   4062    0 480 24   1    1  1   1   0
## 4063   4063    0 480 24   1    1  1   1   0
## 4064   4064    0 480 24   1    1  1   1   0
## 4065   4065    0 480 24   1    1  1   1   0
## 4066   4066    0 480 24   1    1  1   1   0
## 4067   4067    0 480 24   1    1  1   1   0
## 4068   4068    0 480 24   1    1  1   1   0
## 4069   4069    0 480 24   1    1  1   1   0
## 4070   4070    0 480 24   1    1  1   1   0
## 4071   4071    0 480 24   1    1  1   1   0
## 4072   4072    0 480 24   1    1  1   1   0
## 4073   4073    0 480 24   1    1  1   1   0
## 4074   4074    0 480 24   1    1  1   1   0
## 4075   4075    0 480 24   1    1  1   1   0
## 4076   4076    0 480 24   1    1  1   1   0
## 4077   4077    0 480 24   1    1  1   1   0
## 4078   4078    0 480 24   1    1  1   1   0
## 4079   4079    0 480 24   1    1  1   1   0
## 4080   4080    0 480 24   1    1  1   1   0
## 4081   4081    0 480 24   1    1  1   1   0
## 4082   4082    0 480 24   1    1  1   1   0
## 4083   4083    0 480 24   1    1  1   1   0
## 4084   4084    0 480 24   1    1  1   1   0
## 4085   4085    0 480 24   1    1  1   1   0
## 4086   4086    0 480 24   1    1  1   1   0
## 4087   4087    0 480 24   1    1  1   1   0
## 4088   4088    0 480 24   1    1  1   1   0
## 4089   4089    0 480 24   1    1  1   1   0
## 4090   4090    0 480 24   1    1  1   1   0
## 4091   4091    0 480 24   1    1  1   1   0
## 4092   4092    0 480 24   1    1  1   1   0
## 4093   4093    0 480 24   1    1  1   1   0
## 4094   4094    0 480 24   1    1  1   1   0
## 4095   4095    0 480 24   1    1  1   1   0
## 4096   4096    0 480 24   1    1  1   1   0
## 4097   4097    0 480 24   1    1  1   1   0
## 4098   4098    0 480 24   1    1  1   1   0
## 4099   4099    0 480 24   1    1  1   1   0
## 4100   4100    0 480 24   1    1  1   1   0
## 4101   4101    0 480 24   1    1  1   1   0
## 4102   4102    0 480 24   1    1  1   1   0
## 4103   4103    0 480 24   1    1  1   1   0
## 4104   4104    0 480 24   1    1  1   1   0
## 4105   4105    0 480 24   1    1  1   1   0
## 4106   4106    0 480 24   1    1  1   1   0
## 4107   4107    0 480 24   1    1  1   1   0
## 4108   4108    0 480 24   1    1  1   1   0
## 4109   4109    0 480 24   1    1  1   1   0
## 4110   4110    0 480 24   1    1  1   1   0
## 4111   4111    0 480 24   1    1  1   1   0
## 4112   4112    0 480 24   1    1  1   1   0
## 4113   4113    0 480 24   1    1  1   1   0
## 4114   4114    0 480 24   1    1  1   1   0
## 4115   4115    0 480 24   1    1  1   1   0
## 4116   4116    0 480 24   1    1  1   1   0
## 4117   4117    0 480 24   1    1  1   1   0
## 4118   4118    0 480 24   1    1  1   1   0
## 4119   4119    0 480 24   1    1  1   1   0
## 4120   4120    0 480 24   1    1  1   1   0
## 4121   4121    0 480 24   1    1  1   1   0
## 4122   4122    0 480 24   1    1  1   1   0
## 4123   4123    0 480 24   1    1  1   1   0
## 4124   4124    0 480 24   1    1  1   1   0
## 4125   4125    0 480 24   1    1  1   1   0
## 4126   4126    0 480 24   1    1  1   1   0
## 4127   4127    0 480 24   1    1  1   1   0
## 4128   4128    0 480 24   1    1  1   1   0
## 4129   4129    0 480 24   1    1  1   1   0
## 4130   4130    0 480 24   1    1  1   1   0
## 4131   4131    0 480 24   1    1  1   1   0
## 4132   4132    0 480 24   1    1  1   1   0
## 4133   4133    0 480 24   1    1  1   1   0
## 4134   4134    0 480 24   1    1  1   1   0
## 4135   4135    0 480 24   1    1  1   1   0
## 4136   4136    0 480 24   1    1  1   1   0
## 4137   4137    0 480 24   1    1  1   1   0
## 4138   4138    0 480 24   1    1  1   1   0
## 4139   4139    0 480 24   1    1  1   1   0
## 4140   4140    0 480 24   1    1  1   1   0
## 4141   4141    0 480 24   1    1  1   1   0
## 4142   4142    0 480 24   1    1  1   1   0
## 4143   4143    0 480 24   1    1  1   1   0
## 4144   4144    0 480 24   1    1  1   1   0
## 4145   4145    0 480 24   1    1  1   1   0
## 4146   4146    0 480 24   1    1  1   1   0
## 4147   4147    0 480 24   1    1  1   1   0
## 4148   4148    0 480 24   1    1  1   1   0
## 4149   4149    0 480 24   1    1  1   1   0
## 4150   4150    0 480 24   1    1  1   1   0
## 4151   4151    0 480 24   1    1  1   1   0
## 4152   4152    0 480 24   1    1  1   1   0
## 4153   4153    0 480 24   1    1  1   1   0
## 4154   4154    0 480 24   1    1  1   1   0
## 4155   4155    0 480 24   1    1  1   1   0
## 4156   4156    0 480 24   1    1  1   1   0
## 4157   4157    0 480 24   1    1  1   1   0
## 4158   4158    0 480 24   1    1  1   1   0
## 4159   4159    0 480 24   1    1  1   1   0
## 4160   4160    0 480 24   1    1  1   1   0
## 4161   4161    0 480 24   1    1  1   1   0
## 4162   4162    0 480 24   1    1  1   1   0
## 4163   4163    0 480 24   1    1  1   1   0
## 4164   4164    0 480 24   1    1  1   1   0
## 4165   4165    0 480 24   1    1  1   1   0
## 4166   4166    0 480 24   1    1  1   1   0
## 4167   4167    0 480 24   1    1  1   1   0
## 4168   4168    0 480 24   1    1  1   1   0
## 4169   4169    0 480 24   1    1  1   1   0
## 4170   4170    0 480 24   1    1  1   1   0
## 4171   4171    0 480 24   1    1  1   1   0
## 4172   4172    0 480 24   1    1  1   1   0
## 4173   4173    0 480 24   1    1  1   1   0
## 4174   4174    0 480 24   1    1  1   1   0
## 4175   4175    0 480 24   1    1  1   1   0
## 4176   4176    0 480 24   1    1  1   1   0
## 4177   4177    0 480 24   1    1  1   1   0
## 4178   4178    0 480 24   1    1  1   1   0
## 4179   4179    0 480 24   1    1  1   1   0
## 4180   4180    0 480 24   1    1  1   1   0
## 4181   4181    0 480 24   1    1  1   1   0
## 4182   4182    0 480 24   1    1  1   1   0
## 4183   4183    0 480 24   1    1  1   1   0
## 4184   4184    0 480 24   1    1  1   1   0
## 4185   4185    0 480 24   1    1  1   1   0
## 4186   4186    0 480 24   1    1  1   1   0
## 4187   4187    0 480 24   1    1  1   1   0
## 4188   4188    0 480 24   1    1  1   1   0
## 4189   4189    0 480 24   1    1  1   1   0
## 4190   4190    0 480 24   1    1  1   1   0
## 4191   4191    0 480 24   1    1  1   1   0
## 4192   4192    0 480 24   1    1  1   1   0
## 4193   4193    0 480 24   1    1  1   1   0
## 4194   4194    0 480 24   1    1  1   1   0
## 4195   4195    0 480 24   1    1  1   1   0
## 4196   4196    0 480 24   1    1  1   1   0
## 4197   4197    0 480 24   1    1  1   1   0
## 4198   4198    0 480 24   1    1  1   1   0
## 4199   4199    0 480 24   1    1  1   1   0
## 4200   4200    0 480 24   1    1  1   1   0
## 4201   4201    0 480 24   1    1  1   1   0
## 4202   4202    0 480 24   1    1  1   1   0
## 4203   4203    0 480 24   1    1  1   1   0
## 4204   4204    0 480 24   1    1  1   1   0
## 4205   4205    0 480 24   1    1  1   1   0
## 4206   4206    0 480 24   1    1  1   1   0
## 4207   4207    0 480 24   1    1  1   1   0
## 4208   4208    0 480 24   1    1  1   1   0
## 4209   4209    0 480 24   1    1  1   1   0
## 4210   4210    0 480 24   1    1  1   1   0
## 4211   4211    0 480 24   1    1  1   1   0
## 4212   4212    0 480 24   1    1  1   1   0
## 4213   4213    0 480 24   1    1  1   1   0
## 4214   4214    0 480 24   1    1  1   1   0
## 4215   4215    0 480 24   1    1  1   1   0
## 4216   4216    0 480 24   1    1  1   1   0
## 4217   4217    0 480 24   1    1  1   1   0
## 4218   4218    0 480 24   1    1  1   1   0
## 4219   4219    0 480 24   1    1  1   1   0
## 4220   4220    0 480 24   1    1  1   1   0
## 4221   4221    0 480 24   1    1  1   1   0
## 4222   4222    0 480 24   1    1  1   1   0
## 4223   4223    0 480 24   1    1  1   1   0
## 4224   4224    0 480 24   1    1  1   1   0
## 4225   4225    0 480 24   1    1  1   1   0
## 4226   4226    0 480 24   1    1  1   1   0
## 4227   4227    0 480 24   1    1  1   1   0
## 4228   4228    0 480 24   1    1  1   1   0
## 4229   4229    0 480 24   1    1  1   1   0
## 4230   4230    0 480 24   1    1  1   1   0
## 4231   4231    0 480 24   1    1  1   1   0
## 4232   4232    0 480 24   1    1  1   1   0
## 4233   4233    0 480 24   1    1  1   1   0
## 4234   4234    0 480 24   1    1  1   1   0
## 4235   4235    0 480 24   1    1  1   1   0
## 4236   4236    0 480 24   1    1  1   1   0
## 4237   4237    0 480 24   1    1  1   1   0
## 4238   4238    0 480 24   1    1  1   1   0
## 4239   4239    0 480 24   1    1  1   1   0
## 4240   4240    0 480 24   1    1  1   1   0
## 4241   4241    0 480 24   1    1  1   1   0
## 4242   4242    0 480 24   1    1  1   1   0
## 4243   4243    0 480 24   1    1  1   1   0
## 4244   4244    0 480 24   1    1  1   1   0
## 4245   4245    0 480 24   1    1  1   1   0
## 4246   4246    0 480 24   1    1  1   1   0
## 4247   4247    0 480 24   1    1  1   1   0
## 4248   4248    0 480 24   1    1  1   1   0
## 4249   4249    0 480 24   1    1  1   1   0
## 4250   4250    0 480 24   1    1  1   1   0
## 4251   4251    0 480 24   1    1  1   1   0
## 4252   4252    0 480 24   1    1  1   1   0
## 4253   4253    0 480 24   1    1  1   1   0
## 4254   4254    0 480 24   1    1  1   1   0
## 4255   4255    0 480 24   1    1  1   1   0
## 4256   4256    0 480 24   1    1  1   1   0
## 4257   4257    0 480 24   1    1  1   1   0
## 4258   4258    0 480 24   1    1  1   1   0
## 4259   4259    0 480 24   1    1  1   1   0
## 4260   4260    0 480 24   1    1  1   1   0
## 4261   4261    0 480 24   1    1  1   1   0
## 4262   4262    0 480 24   1    1  1   1   0
## 4263   4263    0 480 24   1    1  1   1   0
## 4264   4264    0 480 24   1    1  1   1   0
## 4265   4265    0 480 24   1    1  1   1   0
## 4266   4266    0 480 24   1    1  1   1   0
## 4267   4267    0 480 24   1    1  1   1   0
## 4268   4268    0 480 24   1    1  1   1   0
## 4269   4269    0 480 24   1    1  1   1   0
## 4270   4270    0 480 24   1    1  1   1   0
## 4271   4271    0 480 24   1    1  1   1   0
## 4272   4272    0 480 24   1    1  1   1   0
## 4273   4273    0 480 24   1    1  1   1   0
## 4274   4274    0 480 24   1    1  1   1   0
## 4275   4275    0 480 24   1    1  1   1   0
## 4276   4276    0 480 24   1    1  1   1   0
## 4277   4277    0 480 24   1    1  1   1   0
## 4278   4278    0 480 24   1    1  1   1   0
## 4279   4279    0 480 24   1    1  1   1   0
## 4280   4280    0 480 24   1    1  1   1   0
## 4281   4281    0 480 24   1    1  1   1   0
## 4282   4282    0 480 24   1    1  1   1   0
## 4283   4283    0 480 24   1    1  1   1   0
## 4284   4284    0 480 24   1    1  1   1   0
## 4285   4285    0 480 24   1    1  1   1   0
## 4286   4286    0 480 24   1    1  1   1   0
## 4287   4287    0 480 24   1    1  1   1   0
## 4288   4288    0 480 24   1    1  1   1   0
## 4289   4289    0 480 24   1    1  1   1   0
## 4290   4290    0 480 24   1    1  1   1   0
## 4291   4291    0 480 24   1    1  1   1   0
## 4292   4292    0 480 24   1    1  1   1   0
## 4293   4293    0 480 24   1    1  1   1   0
## 4294   4294    0 480 24   1    1  1   1   0
## 4295   4295    0 480 24   1    1  1   1   0
## 4296   4296    0 480 24   1    1  1   1   0
## 4297   4297    0 480 24   1    1  1   1   0
## 4298   4298    0 480 24   1    1  1   1   0
## 4299   4299    0 480 24   1    1  1   1   0
## 4300   4300    0 480 24   1    1  1   1   0
## 4301   4301    0 480 24   1    1  1   1   0
## 4302   4302    0 480 24   1    1  1   1   0
## 4303   4303    0 480 24   1    1  1   1   0
## 4304   4304    0 480 24   1    1  1   1   0
## 4305   4305    0 480 24   1    1  1   1   0
## 4306   4306    0 480 24   1    1  1   1   0
## 4307   4307    0 480 24   1    1  1   1   0
## 4308   4308    0 480 24   1    1  1   1   0
## 4309   4309    0 480 24   1    1  1   1   0
## 4310   4310    0 480 24   1    1  1   1   0
## 4311   4311    0 480 24   1    1  1   1   0
## 4312   4312    0 480 24   1    1  1   1   0
## 4313   4313    0 480 24   1    1  1   1   0
## 4314   4314    0 480 24   1    1  1   1   0
## 4315   4315    0 480 24   1    1  1   1   0
## 4316   4316    0 480 24   1    1  1   1   0
## 4317   4317    0 480 24   1    1  1   1   0
## 4318   4318    0 480 24   1    1  1   1   0
## 4319   4319    0 480 24   1    1  1   1   0
## 4320   4320    0 480 24   1    1  1   1   0
## 4321   4321    0 480 24   1    1  1   1   0
## 4322   4322    0 480 24   1    1  1   1   0
## 4323   4323    0 480 24   1    1  1   1   0
## 4324   4324    0 480 24   1    1  1   1   0
## 4325   4325    0 480 24   1    1  1   1   0
## 4326   4326    0 480 24   1    1  1   1   0
## 4327   4327    0 480 24   1    1  1   1   0
## 4328   4328    0 480 24   1    1  1   1   0
## 4329   4329    0 480 24   1    1  1   1   0
## 4330   4330    0 480 24   1    1  1   1   0
## 4331   4331    0 480 24   1    1  1   1   0
## 4332   4332    0 480 24   1    1  1   1   0
## 4333   4333    0 480 24   1    1  1   1   0
## 4334   4334    0 480 24   1    1  1   1   0
## 4335   4335    0 480 24   1    1  1   1   0
## 4336   4336    0 480 24   1    1  1   1   0
## 4337   4337    0 480 24   1    1  1   1   0
## 4338   4338    0 480 24   1    1  1   1   0
## 4339   4339    0 480 24   1    1  1   1   0
## 4340   4340    0 480 24   1    1  1   1   0
## 4341   4341    0 480 24   1    1  1   1   0
## 4342   4342    0 480 24   1    1  1   1   0
## 4343   4343    0 480 24   1    1  1   1   0
## 4344   4344    0 480 24   1    1  1   1   0
## 4345   4345    0 480 24   1    1  1   1   0
## 4346   4346    0 480 24   1    1  1   1   0
## 4347   4347    0 480 24   1    1  1   1   0
## 4348   4348    0 480 24   1    1  1   1   0
## 4349   4349    0 480 24   1    1  1   1   0
## 4350   4350    0 480 24   1    1  1   1   0
## 4351   4351    0 480 24   1    1  1   1   0
## 4352   4352    0 480 24   1    1  1   1   0
## 4353   4353    0 480 24   1    1  1   1   0
## 4354   4354    0 480 24   1    1  1   1   0
## 4355   4355    0 480 24   1    1  1   1   0
## 4356   4356    0 480 24   1    1  1   1   0
## 4357   4357    0 480 24   1    1  1   1   0
## 4358   4358    0 480 24   1    1  1   1   0
## 4359   4359    0 480 24   1    1  1   1   0
## 4360   4360    0 480 24   1    1  1   1   0
## 4361   4361    0 480 24   1    1  1   1   0
## 4362   4362    0 480 24   1    1  1   1   0
## 4363   4363    0 480 24   1    1  1   1   0
## 4364   4364    0 480 24   1    1  1   1   0
## 4365   4365    0 480 24   1    1  1   1   0
## 4366   4366    0 480 24   1    1  1   1   0
## 4367   4367    0 480 24   1    1  1   1   0
## 4368   4368    0 480 24   1    1  1   1   0
## 4369   4369    0 480 24   1    1  1   1   0
## 4370   4370    0 480 24   1    1  1   1   0
## 4371   4371    0 480 24   1    1  1   1   0
## 4372   4372    0 480 24   1    1  1   1   0
## 4373   4373    0 480 24   1    1  1   1   0
## 4374   4374    0 480 24   1    1  1   1   0
## 4375   4375    0 480 24   1    1  1   1   0
## 4376   4376    0 480 24   1    1  1   1   0
## 4377   4377    0 480 24   1    1  1   1   0
## 4378   4378    0 480 24   1    1  1   1   0
## 4379   4379    0 480 24   1    1  1   1   0
## 4380   4380    0 480 24   1    1  1   1   0
## 4381   4381    0 480 24   1    1  1   1   0
## 4382   4382    0 480 24   1    1  1   1   0
## 4383   4383    0 480 24   1    1  1   1   0
## 4384   4384    0 480 24   1    1  1   1   0
## 4385   4385    0 480 24   1    1  1   1   0
## 4386   4386    0 480 24   1    1  1   1   0
## 4387   4387    0 480 24   1    1  1   1   0
## 4388   4388    0 480 24   1    1  1   1   0
## 4389   4389    0 480 24   1    1  1   1   0
## 4390   4390    0 480 24   1    1  1   1   0
## 4391   4391    0 480 24   1    1  1   1   0
## 4392   4392    0 480 24   1    1  1   1   0
## 4393   4393    0 480 24   1    1  1   1   0
## 4394   4394    0 480 24   1    1  1   1   0
## 4395   4395    0 480 24   1    1  1   1   0
## 4396   4396    0 480 24   1    1  1   1   0
## 4397   4397    0 480 24   1    1  1   1   0
## 4398   4398    0 480 24   1    1  1   1   0
## 4399   4399    0 480 24   1    1  1   1   0
## 4400   4400    0 480 24   1    1  1   1   0
## 4401   4401    0 480 24   1    1  1   1   0
## 4402   4402    0 480 24   1    1  1   1   0
## 4403   4403    0 480 24   1    1  1   1   0
## 4404   4404    0 480 24   1    1  1   1   0
## 4405   4405    0 480 24   1    1  1   1   0
## 4406   4406    0 480 24   1    1  1   1   0
## 4407   4407    0 480 24   1    1  1   1   0
## 4408   4408    0 480 24   1    1  1   1   0
## 4409   4409    0 480 24   1    1  1   1   0
## 4410   4410    0 480 24   1    1  1   1   0
## 4411   4411    0 480 24   1    1  1   1   0
## 4412   4412    0 480 24   1    1  1   1   0
## 4413   4413    0 480 24   1    1  1   1   0
## 4414   4414    0 480 24   1    1  1   1   0
## 4415   4415    0 480 24   1    1  1   1   0
## 4416   4416    0 480 24   1    1  1   1   0
## 4417   4417    0 480 24   1    1  1   1   0
## 4418   4418    0 480 24   1    1  1   1   0
## 4419   4419    0 480 24   1    1  1   1   0
## 4420   4420    0 480 24   1    1  1   1   0
## 4421   4421    0 480 24   1    1  1   1   0
## 4422   4422    0 480 24   1    1  1   1   0
## 4423   4423    0 480 24   1    1  1   1   0
## 4424   4424    0 480 24   1    1  1   1   0
## 4425   4425    0 480 24   1    1  1   1   0
## 4426   4426    0 480 24   1    1  1   1   0
## 4427   4427    0 480 24   1    1  1   1   0
## 4428   4428    0 480 24   1    1  1   1   0
## 4429   4429    0 480 24   1    1  1   1   0
## 4430   4430    0 480 24   1    1  1   1   0
## 4431   4431    0 480 24   1    1  1   1   0
## 4432   4432    0 480 24   1    1  1   1   0
## 4433   4433    0 480 24   1    1  1   1   0
## 4434   4434    0 480 24   1    1  1   1   0
## 4435   4435    0 480 24   1    1  1   1   0
## 4436   4436    0 480 24   1    1  1   1   0
## 4437   4437    0 480 24   1    1  1   1   0
## 4438   4438    0 480 24   1    1  1   1   0
## 4439   4439    0 480 24   1    1  1   1   0
## 4440   4440    0 480 24   1    1  1   1   0
## 4441   4441    0 480 24   1    1  1   1   0
## 4442   4442    0 480 24   1    1  1   1   0
## 4443   4443    0 480 24   1    1  1   1   0
## 4444   4444    0 480 24   1    1  1   1   0
## 4445   4445    0 480 24   1    1  1   1   0
## 4446   4446    0 480 24   1    1  1   1   0
## 4447   4447    0 480 24   1    1  1   1   0
## 4448   4448    0 480 24   1    1  1   1   0
## 4449   4449    0 480 24   1    1  1   1   0
## 4450   4450    0 480 24   1    1  1   1   0
## 4451   4451    0 480 24   1    1  1   1   0
## 4452   4452    0 480 24   1    1  1   1   0
## 4453   4453    0 480 24   1    1  1   1   0
## 4454   4454    0 480 24   1    1  1   1   0
## 4455   4455    0 480 24   1    1  1   1   0
## 4456   4456    0 480 24   1    1  1   1   0
## 4457   4457    0 480 24   1    1  1   1   0
## 4458   4458    0 480 24   1    1  1   1   0
## 4459   4459    0 480 24   1    1  1   1   0
## 4460   4460    0 480 24   1    1  1   1   0
## 4461   4461    0 480 24   1    1  1   1   0
## 4462   4462    0 480 24   1    1  1   1   0
## 4463   4463    0 480 24   1    1  1   1   0
## 4464   4464    0 480 24   1    1  1   1   0
## 4465   4465    0 480 24   1    1  1   1   0
## 4466   4466    0 480 24   1    1  1   1   0
## 4467   4467    0 480 24   1    1  1   1   0
## 4468   4468    0 480 24   1    1  1   1   0
## 4469   4469    0 480 24   1    1  1   1   0
## 4470   4470    0 480 24   1    1  1   1   0
## 4471   4471    0 480 24   1    1  1   1   0
## 4472   4472    0 480 24   1    1  1   1   0
## 4473   4473    0 480 24   1    1  1   1   0
## 4474   4474    0 480 24   1    1  1   1   0
## 4475   4475    0 480 24   1    1  1   1   0
## 4476   4476    0 480 24   1    1  1   1   0
## 4477   4477    0 480 24   1    1  1   1   0
## 4478   4478    0 480 24   1    1  1   1   0
## 4479   4479    0 480 24   1    1  1   1   0
## 4480   4480    0 480 24   1    1  1   1   0
## 4481   4481    0 480 24   1    1  1   1   0
## 4482   4482    0 480 24   1    1  1   1   0
## 4483   4483    0 480 24   1    1  1   1   0
## 4484   4484    0 480 24   1    1  1   1   0
## 4485   4485    0 480 24   1    1  1   1   0
## 4486   4486    0 480 24   1    1  1   1   0
## 4487   4487    0 480 24   1    1  1   1   0
## 4488   4488    0 480 24   1    1  1   1   0
## 4489   4489    0 480 24   1    1  1   1   0
## 4490   4490    0 480 24   1    1  1   1   0
## 4491   4491    0 480 24   1    1  1   1   0
## 4492   4492    0 480 24   1    1  1   1   0
## 4493   4493    0 480 24   1    1  1   1   0
## 4494   4494    0 480 24   1    1  1   1   0
## 4495   4495    0 480 24   1    1  1   1   0
## 4496   4496    0 480 24   1    1  1   1   0
## 4497   4497    0 480 24   1    1  1   1   0
## 4498   4498    0 480 24   1    1  1   1   0
## 4499   4499    0 480 24   1    1  1   1   0
## 4500   4500    0 480 24   1    1  1   1   0
## 4501   4501    0 480 24   1    1  1   1   0
## 4502   4502    0 480 24   1    1  1   1   0
## 4503   4503    0 480 24   1    1  1   1   0
## 4504   4504    0 480 24   1    1  1   1   0
## 4505   4505    0 480 24   1    1  1   1   0
## 4506   4506    0 480 24   1    1  1   1   0
## 4507   4507    0 480 24   1    1  1   1   0
## 4508   4508    0 480 24   1    1  1   1   0
## 4509   4509    0 480 24   1    1  1   1   0
## 4510   4510    0 480 24   1    1  1   1   0
## 4511   4511    0 480 24   1    1  1   1   0
## 4512   4512    0 480 24   1    1  1   1   0
## 4513   4513    0 480 24   1    1  1   1   0
## 4514   4514    0 480 24   1    1  1   1   0
## 4515   4515    0 480 24   1    1  1   1   0
## 4516   4516    0 480 24   1    1  1   1   0
## 4517   4517    0 480 24   1    1  1   1   0
## 4518   4518    0 480 24   1    1  1   1   0
## 4519   4519    0 480 24   1    1  1   1   0
## 4520   4520    0 480 24   1    1  1   1   0
## 4521   4521    0 480 24   1    1  1   1   0
## 4522   4522    0 480 24   1    1  1   1   0
## 4523   4523    0 480 24   1    1  1   1   0
## 4524   4524    0 480 24   1    1  1   1   0
## 4525   4525    0 480 24   1    1  1   1   0
## 4526   4526    0 480 24   1    1  1   1   0
## 4527   4527    0 480 24   1    1  1   1   0
## 4528   4528    0 480 24   1    1  1   1   0
## 4529   4529    0 480 24   1    1  1   1   0
## 4530   4530    0 480 24   1    1  1   1   0
## 4531   4531    0 480 24   1    1  1   1   0
## 4532   4532    0 480 24   1    1  1   1   0
## 4533   4533    0 480 24   1    1  1   1   0
## 4534   4534    0 480 24   1    1  1   1   0
## 4535   4535    0 480 24   1    1  1   1   0
## 4536   4536    0 480 24   1    1  1   1   0
## 4537   4537    0 480 24   1    1  1   1   0
## 4538   4538    0 480 24   1    1  1   1   0
## 4539   4539    0 480 24   1    1  1   1   0
## 4540   4540    0 480 24   1    1  1   1   0
## 4541   4541    0 480 24   1    1  1   1   0
## 4542   4542    0 480 24   1    1  1   1   0
## 4543   4543    0 480 24   1    1  1   1   0
## 4544   4544    0 480 24   1    1  1   1   0
## 4545   4545    0 480 24   1    1  1   1   0
## 4546   4546    0 480 24   1    1  1   1   0
## 4547   4547    0 480 24   1    1  1   1   0
## 4548   4548    0 480 24   1    1  1   1   0
## 4549   4549    0 480 24   1    1  1   1   0
## 4550   4550    0 480 24   1    1  1   1   0
## 4551   4551    0 480 24   1    1  1   1   0
## 4552   4552    0 480 24   1    1  1   1   0
## 4553   4553    0 480 24   1    1  1   1   0
## 4554   4554    0 480 24   1    1  1   1   0
## 4555   4555    0 480 24   1    1  1   1   0
## 4556   4556    0 480 24   1    1  1   1   0
## 4557   4557    0 480 24   1    1  1   1   0
## 4558   4558    0 480 24   1    1  1   1   0
## 4559   4559    0 480 24   1    1  1   1   0
## 4560   4560    0 480 24   1    1  1   1   0
## 4561   4561    0 480 24   1    1  1   1   0
## 4562   4562    0 480 24   1    1  1   1   0
## 4563   4563    0 480 24   1    1  1   1   0
## 4564   4564    0 480 24   1    1  1   1   0
## 4565   4565    0 480 24   1    1  1   1   0
## 4566   4566    0 480 24   1    1  1   1   0
## 4567   4567    0 480 24   1    1  1   1   0
## 4568   4568    0 480 24   1    1  1   1   0
## 4569   4569    0 480 24   1    1  1   1   0
## 4570   4570    0 480 24   1    1  1   1   0
## 4571   4571    0 480 24   1    1  1   1   0
## 4572   4572    0 480 24   1    1  1   1   0
## 4573   4573    0 480 24   1    1  1   1   0
## 4574   4574    0 480 24   1    1  1   1   0
## 4575   4575    0 480 24   1    1  1   1   0
## 4576   4576    0 480 24   1    1  1   1   0
## 4577   4577    0 480 24   1    1  1   1   0
## 4578   4578    0 480 24   1    1  1   1   0
## 4579   4579    0 480 24   1    1  1   1   0
## 4580   4580    0 480 24   1    1  1   1   0
## 4581   4581    0 480 24   1    1  1   1   0
## 4582   4582    0 480 24   1    1  1   1   0
## 4583   4583    0 480 24   1    1  1   1   0
## 4584   4584    0 480 24   1    1  1   1   0
## 4585   4585    0 480 24   1    1  1   1   0
## 4586   4586    0 480 24   1    1  1   1   0
## 4587   4587    0 480 24   1    1  1   1   0
## 4588   4588    0 480 24   1    1  1   1   0
## 4589   4589    0 480 24   1    1  1   1   0
## 4590   4590    0 480 24   1    1  1   1   0
## 4591   4591    0 480 24   1    1  1   1   0
## 4592   4592    0 480 24   1    1  1   1   0
## 4593   4593    0 480 24   1    1  1   1   0
## 4594   4594    0 480 24   1    1  1   1   0
## 4595   4595    0 480 24   1    1  1   1   0
## 4596   4596    0 480 24   1    1  1   1   0
## 4597   4597    0 480 24   1    1  1   1   0
## 4598   4598    0 480 24   1    1  1   1   0
## 4599   4599    0 480 24   1    1  1   1   0
## 4600   4600    0 480 24   1    1  1   1   0
## 4601   4601    0 480 24   1    1  1   1   0
## 4602   4602    0 480 24   1    1  1   1   0
## 4603   4603    0 480 24   1    1  1   1   0
## 4604   4604    0 480 24   1    1  1   1   0
## 4605   4605    0 480 24   1    1  1   1   0
## 4606   4606    0 480 24   1    1  1   1   0
## 4607   4607    0 480 24   1    1  1   1   0
## 4608   4608    0 480 24   1    1  1   1   0
## 4609   4609    0 480 24   1    1  1   1   0
## 4610   4610    0 480 24   1    1  1   1   0
## 4611   4611    0 480 24   1    1  1   1   0
## 4612   4612    0 480 24   1    1  1   1   0
## 4613   4613    0 480 24   1    1  1   1   0
## 4614   4614    0 480 24   1    1  1   1   0
## 4615   4615    0 480 24   1    1  1   1   0
## 4616   4616    0 480 24   1    1  1   1   0
## 4617   4617    0 480 24   1    1  1   1   0
## 4618   4618    0 480 24   1    1  1   1   0
## 4619   4619    0 480 24   1    1  1   1   0
## 4620   4620    0 480 24   1    1  1   1   0
## 4621   4621    0 480 24   1    1  1   1   0
## 4622   4622    0 480 24   1    1  1   1   0
## 4623   4623    0 480 24   1    1  1   1   0
## 4624   4624    0 480 24   1    1  1   1   0
## 4625   4625    0 480 24   1    1  1   1   0
## 4626   4626    0 480 24   1    1  1   1   0
## 4627   4627    0 480 24   1    1  1   1   0
## 4628   4628    0 480 24   1    1  1   1   0
## 4629   4629    0 480 24   1    1  1   1   0
## 4630   4630    0 480 24   1    1  1   1   0
## 4631   4631    0 480 24   1    1  1   1   0
## 4632   4632    0 480 24   1    1  1   1   0
## 4633   4633    0 480 24   1    1  1   1   0
## 4634   4634    0 480 24   1    1  1   1   0
## 4635   4635    0 480 24   1    1  1   1   0
## 4636   4636    0 480 24   1    1  1   1   0
## 4637   4637    0 480 24   1    1  1   1   0
## 4638   4638    0 480 24   1    1  1   1   0
## 4639   4639    0 480 24   1    1  1   1   0
## 4640   4640    0 480 24   1    1  1   1   0
## 4641   4641    0 480 24   1    1  1   1   0
## 4642   4642    0 480 24   1    1  1   1   0
## 4643   4643    0 480 24   1    1  1   1   0
## 4644   4644    0 480 24   1    1  1   1   0
## 4645   4645    0 480 24   1    1  1   1   0
## 4646   4646    0 480 24   1    1  1   1   0
## 4647   4647    0 480 24   1    1  1   1   0
## 4648   4648    0 480 24   1    1  1   1   0
## 4649   4649    0 480 24   1    1  1   1   0
## 4650   4650    0 480 24   1    1  1   1   0
## 4651   4651    0 480 24   1    1  1   1   0
## 4652   4652    0 480 24   1    1  1   1   0
## 4653   4653    0 480 24   1    1  1   1   0
## 4654   4654    0 480 24   1    1  1   1   0
## 4655   4655    0 480 24   1    1  1   1   0
## 4656   4656    0 480 24   1    1  1   1   0
## 4657   4657    0 480 24   1    1  1   1   0
## 4658   4658    0 480 24   1    1  1   1   0
## 4659   4659    0 480 24   1    1  1   1   0
## 4660   4660    0 480 24   1    1  1   1   0
## 4661   4661    0 480 24   1    1  1   1   0
## 4662   4662    0 480 24   1    1  1   1   0
## 4663   4663    0 480 24   1    1  1   1   0
## 4664   4664    0 480 24   1    1  1   1   0
## 4665   4665    0 480 24   1    1  1   1   0
## 4666   4666    0 480 24   1    1  1   1   0
## 4667   4667    0 480 24   1    1  1   1   0
## 4668   4668    0 480 24   1    1  1   1   0
## 4669   4669    0 480 24   1    1  1   1   0
## 4670   4670    0 480 24   1    1  1   1   0
## 4671   4671    0 480 24   1    1  1   1   0
## 4672   4672    0 480 24   1    1  1   1   0
## 4673   4673    0 480 24   1    1  1   1   0
## 4674   4674    0 480 24   1    1  1   1   0
## 4675   4675    0 480 24   1    1  1   1   0
## 4676   4676    0 480 24   1    1  1   1   0
## 4677   4677    0 480 24   1    1  1   1   0
## 4678   4678    0 480 24   1    1  1   1   0
## 4679   4679    0 480 24   1    1  1   1   0
## 4680   4680    0 480 24   1    1  1   1   0
## 4681   4681    0 480 24   1    1  1   1   0
## 4682   4682    0 480 24   1    1  1   1   0
## 4683   4683    0 480 24   1    1  1   1   0
## 4684   4684    0 480 24   1    1  1   1   0
## 4685   4685    0 480 24   1    1  1   1   0
## 4686   4686    0 480 24   1    1  1   1   0
## 4687   4687    0 480 24   1    1  1   1   0
## 4688   4688    0 480 24   1    1  1   1   0
## 4689   4689    0 480 24   1    1  1   1   0
## 4690   4690    0 480 24   1    1  1   1   0
## 4691   4691    0 480 24   1    1  1   1   0
## 4692   4692    0 480 24   1    1  1   1   0
## 4693   4693    0 480 24   1    1  1   1   0
## 4694   4694    0 480 24   1    1  1   1   0
## 4695   4695    0 480 24   1    1  1   1   0
## 4696   4696    0 480 24   1    1  1   1   0
## 4697   4697    0 480 24   1    1  1   1   0
## 4698   4698    0 480 24   1    1  1   1   0
## 4699   4699    0 480 24   1    1  1   1   0
## 4700   4700    0 480 24   1    1  1   1   0
## 4701   4701    0 480 24   1    1  1   1   0
## 4702   4702    0 480 24   1    1  1   1   0
## 4703   4703    0 480 24   1    1  1   1   0
## 4704   4704    0 480 24   1    1  1   1   0
## 4705   4705    0 480 24   1    1  1   1   0
## 4706   4706    0 480 24   1    1  1   1   0
## 4707   4707    0 480 24   1    1  1   1   0
## 4708   4708    0 480 24   1    1  1   1   0
## 4709   4709    0 480 24   1    1  1   1   0
## 4710   4710    0 480 24   1    1  1   1   0
## 4711   4711    0 480 24   1    1  1   1   0
## 4712   4712    0 480 24   1    1  1   1   0
## 4713   4713    0 480 24   1    1  1   1   0
## 4714   4714    0 480 24   1    1  1   1   0
## 4715   4715    0 480 24   1    1  1   1   0
## 4716   4716    0 480 24   1    1  1   1   0
## 4717   4717    0 480 24   1    1  1   1   0
## 4718   4718    0 480 24   1    1  1   1   0
## 4719   4719    0 480 24   1    1  1   1   0
## 4720   4720    0 480 24   1    1  1   1   0
## 4721   4721    0 480 24   1    1  1   1   0
## 4722   4722    0 480 24   1    1  1   1   0
## 4723   4723    0 480 24   1    1  1   1   0
## 4724   4724    0 480 24   1    1  1   1   0
## 4725   4725    0 480 24   1    1  1   1   0
## 4726   4726    0 480 24   1    1  1   1   0
## 4727   4727    0 480 24   1    1  1   1   0
## 4728   4728    0 480 24   1    1  1   1   0
## 4729   4729    0 480 24   1    1  1   1   0
## 4730   4730    0 480 24   1    1  1   1   0
## 4731   4731    0 480 24   1    1  1   1   0
## 4732   4732    0 480 24   1    1  1   1   0
## 4733   4733    0 480 24   1    1  1   1   0
## 4734   4734    0 480 24   1    1  1   1   0
## 4735   4735    0 480 24   1    1  1   1   0
## 4736   4736    0 480 24   1    1  1   1   0
## 4737   4737    0 480 24   1    1  1   1   0
## 4738   4738    0 480 24   1    1  1   1   0
## 4739   4739    0 480 24   1    1  1   1   0
## 4740   4740    0 480 24   1    1  1   1   0
## 4741   4741    0 480 24   1    1  1   1   0
## 4742   4742    0 480 24   1    1  1   1   0
## 4743   4743    0 480 24   1    1  1   1   0
## 4744   4744    0 480 24   1    1  1   1   0
## 4745   4745    0 480 24   1    1  1   1   0
## 4746   4746    0 480 24   1    1  1   1   0
## 4747   4747    0 480 24   1    1  1   1   0
## 4748   4748    0 480 24   1    1  1   1   0
## 4749   4749    0 480 24   1    1  1   1   0
## 4750   4750    0 480 24   1    1  1   1   0
## 4751   4751    0 480 24   1    1  1   1   0
## 4752   4752    0 480 24   1    1  1   1   0
## 4753   4753    0 480 24   1    1  1   1   0
## 4754   4754    0 480 24   1    1  1   1   0
## 4755   4755    0 480 24   1    1  1   1   0
## 4756   4756    0 480 24   1    1  1   1   0
## 4757   4757    0 480 24   1    1  1   1   0
## 4758   4758    0 480 24   1    1  1   1   0
## 4759   4759    0 480 24   1    1  1   1   0
## 4760   4760    0 480 24   1    1  1   1   0
## 4761   4761    0 480 24   1    1  1   1   0
## 4762   4762    0 480 24   1    1  1   1   0
## 4763   4763    0 480 24   1    1  1   1   0
## 4764   4764    0 480 24   1    1  1   1   0
## 4765   4765    0 480 24   1    1  1   1   0
## 4766   4766    0 480 24   1    1  1   1   0
## 4767   4767    0 480 24   1    1  1   1   0
## 4768   4768    0 480 24   1    1  1   1   0
## 4769   4769    0 480 24   1    1  1   1   0
## 4770   4770    0 480 24   1    1  1   1   0
## 4771   4771    0 480 24   1    1  1   1   0
## 4772   4772    0 480 24   1    1  1   1   0
## 4773   4773    0 480 24   1    1  1   1   0
## 4774   4774    0 480 24   1    1  1   1   0
## 4775   4775    0 480 24   1    1  1   1   0
## 4776   4776    0 480 24   1    1  1   1   0
## 4777   4777    0 480 24   1    1  1   1   0
## 4778   4778    0 480 24   1    1  1   1   0
## 4779   4779    0 480 24   1    1  1   1   0
## 4780   4780    0 480 24   1    1  1   1   0
## 4781   4781    0 480 24   1    1  1   1   0
## 4782   4782    0 480 24   1    1  1   1   0
## 4783   4783    0 480 24   1    1  1   1   0
## 4784   4784    0 480 24   1    1  1   1   0
## 4785   4785    0 480 24   1    1  1   1   0
## 4786   4786    0 480 24   1    1  1   1   0
## 4787   4787    0 480 24   1    1  1   1   0
## 4788   4788    0 480 24   1    1  1   1   0
## 4789   4789    0 480 24   1    1  1   1   0
## 4790   4790    0 480 24   1    1  1   1   0
## 4791   4791    0 480 24   1    1  1   1   0
## 4792   4792    0 480 24   1    1  1   1   0
## 4793   4793    0 480 24   1    1  1   1   0
## 4794   4794    0 480 24   1    1  1   1   0
## 4795   4795    0 480 24   1    1  1   1   0
## 4796   4796    0 480 24   1    1  1   1   0
## 4797   4797    0 480 24   1    1  1   1   0
## 4798   4798    0 480 24   1    1  1   1   0
## 4799   4799    0 480 24   1    1  1   1   0
## 4800   4800    0 480 24   1    1  1   1   0
## 4801   4801    0 480 24   1    1  1   1   0
## 4802   4802    0 480 24   1    1  1   1   0
## 4803   4803    0 480 24   1    1  1   1   0
## 4804   4804    0 480 24   1    1  1   1   0
## 4805   4805    0 480 24   1    1  1   1   0
## 4806   4806    0 480 24   1    1  1   1   0
## 4807   4807    0 480 24   1    1  1   1   0
## 4808   4808    0 480 24   1    1  1   1   0
## 4809   4809    0 480 24   1    1  1   1   0
## 4810   4810    0 480 24   1    1  1   1   0
## 4811   4811    0 480 24   1    1  1   1   0
## 4812   4812    0 480 24   1    1  1   1   0
## 4813   4813    0 480 24   1    1  1   1   0
## 4814   4814    0 480 24   1    1  1   1   0
## 4815   4815    0 480 24   1    1  1   1   0
## 4816   4816    0 480 24   1    1  1   1   0
## 4817   4817    0 480 24   1    1  1   1   0
## 4818   4818    0 480 24   1    1  1   1   0
## 4819   4819    0 480 24   1    1  1   1   0
## 4820   4820    0 480 24   1    1  1   1   0
## 4821   4821    0 480 24   1    1  1   1   0
## 4822   4822    0 480 24   1    1  1   1   0
## 4823   4823    0 480 24   1    1  1   1   0
## 4824   4824    0 480 24   1    1  1   1   0
## 4825   4825    0 480 24   1    1  1   1   0
## 4826   4826    0 480 24   1    1  1   1   0
## 4827   4827    0 480 24   1    1  1   1   0
## 4828   4828    0 480 24   1    1  1   1   0
## 4829   4829    0 480 24   1    1  1   1   0
## 4830   4830    0 480 24   1    1  1   1   0
## 4831   4831    0 480 24   1    1  1   1   0
## 4832   4832    0 480 24   1    1  1   1   0
## 4833   4833    0 480 24   1    1  1   1   0
## 4834   4834    0 480 24   1    1  1   1   0
## 4835   4835    0 480 24   1    1  1   1   0
## 4836   4836    0 480 24   1    1  1   1   0
## 4837   4837    0 480 24   1    1  1   1   0
## 4838   4838    0 480 24   1    1  1   1   0
## 4839   4839    0 480 24   1    1  1   1   0
## 4840   4840    0 480 24   1    1  1   1   0
## 4841   4841    0 480 24   1    1  1   1   0
## 4842   4842    0 480 24   1    1  1   1   0
## 4843   4843    0 480 24   1    1  1   1   0
## 4844   4844    0 480 24   1    1  1   1   0
## 4845   4845    0 480 24   1    1  1   1   0
## 4846   4846    0 480 24   1    1  1   1   0
## 4847   4847    0 480 24   1    1  1   1   0
## 4848   4848    0 480 24   1    1  1   1   0
## 4849   4849    0 480 24   1    1  1   1   0
## 4850   4850    0 480 24   1    1  1   1   0
## 4851   4851    0 480 24   1    1  1   1   0
## 4852   4852    0 480 24   1    1  1   1   0
## 4853   4853    0 480 24   1    1  1   1   0
## 4854   4854    0 480 24   1    1  1   1   0
## 4855   4855    0 480 24   1    1  1   1   0
## 4856   4856    0 480 24   1    1  1   1   0
## 4857   4857    0 480 24   1    1  1   1   0
## 4858   4858    0 480 24   1    1  1   1   0
## 4859   4859    0 480 24   1    1  1   1   0
## 4860   4860    0 480 24   1    1  1   1   0
## 4861   4861    0 480 24   1    1  1   1   0
## 4862   4862    0 480 24   1    1  1   1   0
## 4863   4863    0 480 24   1    1  1   1   0
## 4864   4864    0 480 24   1    1  1   1   0
## 4865   4865    0 480 24   1    1  1   1   0
## 4866   4866    0 480 24   1    1  1   1   0
## 4867   4867    0 480 24   1    1  1   1   0
## 4868   4868    0 480 24   1    1  1   1   0
## 4869   4869    0 480 24   1    1  1   1   0
## 4870   4870    0 480 24   1    1  1   1   0
## 4871   4871    0 480 24   1    1  1   1   0
## 4872   4872    0 480 24   1    1  1   1   0
## 4873   4873    0 480 24   1    1  1   1   0
## 4874   4874    0 480 24   1    1  1   1   0
## 4875   4875    0 480 24   1    1  1   1   0
## 4876   4876    0 480 24   1    1  1   1   0
## 4877   4877    0 480 24   1    1  1   1   0
## 4878   4878    0 480 24   1    1  1   1   0
## 4879   4879    0 480 24   1    1  1   1   0
## 4880   4880    0 480 24   1    1  1   1   0
## 4881   4881    0 480 24   1    1  1   1   0
## 4882   4882    0 480 24   1    1  1   1   0
## 4883   4883    0 480 24   1    1  1   1   0
## 4884   4884    0 480 24   1    1  1   1   0
## 4885   4885    0 480 24   1    1  1   1   0
## 4886   4886    0 480 24   1    1  1   1   0
## 4887   4887    0 480 24   1    1  1   1   0
## 4888   4888    0 480 24   1    1  1   1   0
## 4889   4889    0 480 24   1    1  1   1   0
## 4890   4890    0 480 24   1    1  1   1   0
## 4891   4891    0 480 24   1    1  1   1   0
## 4892   4892    0 480 24   1    1  1   1   0
## 4893   4893    0 480 24   1    1  1   1   0
## 4894   4894    0 480 24   1    1  1   1   0
## 4895   4895    0 480 24   1    1  1   1   0
## 4896   4896    0 480 24   1    1  1   1   0
## 4897   4897    0 480 24   1    1  1   1   0
## 4898   4898    0 480 24   1    1  1   1   0
## 4899   4899    0 480 24   1    1  1   1   0
## 4900   4900    0 480 24   1    1  1   1   0
## 4901   4901    0 480 24   1    1  1   1   0
## 4902   4902    0 480 24   1    1  1   1   0
## 4903   4903    0 480 24   1    1  1   1   0
## 4904   4904    0 480 24   1    1  1   1   0
## 4905   4905    0 480 24   1    1  1   1   0
## 4906   4906    0 480 24   1    1  1   1   0
## 4907   4907    0 480 24   1    1  1   1   0
## 4908   4908    0 480 24   1    1  1   1   0
## 4909   4909    0 480 24   1    1  1   1   0
## 4910   4910    0 480 24   1    1  1   1   0
## 4911   4911    0 480 24   1    1  1   1   0
## 4912   4912    0 480 24   1    1  1   1   0
## 4913   4913    0 480 24   1    1  1   1   0
## 4914   4914    0 480 24   1    1  1   1   0
## 4915   4915    0 480 24   1    1  1   1   0
## 4916   4916    0 480 24   1    1  1   1   0
## 4917   4917    0 480 24   1    1  1   1   0
## 4918   4918    0 480 24   1    1  1   1   0
## 4919   4919    0 480 24   1    1  1   1   0
## 4920   4920    0 480 24   1    1  1   1   0
## 4921   4921    0 480 24   1    1  1   1   0
## 4922   4922    0 480 24   1    1  1   1   0
## 4923   4923    0 480 24   1    1  1   1   0
## 4924   4924    0 480 24   1    1  1   1   0
## 4925   4925    0 480 24   1    1  1   1   0
## 4926   4926    0 480 24   1    1  1   1   0
## 4927   4927    0 480 24   1    1  1   1   0
## 4928   4928    0 480 24   1    1  1   1   0
## 4929   4929    0 480 24   1    1  1   1   0
## 4930   4930    0 480 24   1    1  1   1   0
## 4931   4931    0 480 24   1    1  1   1   0
## 4932   4932    0 480 24   1    1  1   1   0
## 4933   4933    0 480 24   1    1  1   1   0
## 4934   4934    0 480 24   1    1  1   1   0
## 4935   4935    0 480 24   1    1  1   1   0
## 4936   4936    0 480 24   1    1  1   1   0
## 4937   4937    0 480 24   1    1  1   1   0
## 4938   4938    0 480 24   1    1  1   1   0
## 4939   4939    0 480 24   1    1  1   1   0
## 4940   4940    0 480 24   1    1  1   1   0
## 4941   4941    0 480 24   1    1  1   1   0
## 4942   4942    0 480 24   1    1  1   1   0
## 4943   4943    0 480 24   1    1  1   1   0
## 4944   4944    0 480 24   1    1  1   1   0
## 4945   4945    0 480 24   1    1  1   1   0
## 4946   4946    0 480 24   1    1  1   1   0
## 4947   4947    0 480 24   1    1  1   1   0
## 4948   4948    0 480 24   1    1  1   1   0
## 4949   4949    0 480 24   1    1  1   1   0
## 4950   4950    0 480 24   1    1  1   1   0
## 4951   4951    0 480 24   1    1  1   1   0
## 4952   4952    0 480 24   1    1  1   1   0
## 4953   4953    0 480 24   1    1  1   1   0
## 4954   4954    0 480 24   1    1  1   1   0
## 4955   4955    0 480 24   1    1  1   1   0
## 4956   4956    0 480 24   1    1  1   1   0
## 4957   4957    0 480 24   1    1  1   1   0
## 4958   4958    0 480 24   1    1  1   1   0
## 4959   4959    0 480 24   1    1  1   1   0
## 4960   4960    0 480 24   1    1  1   1   0
## 4961   4961    0 480 24   1    1  1   1   0
## 4962   4962    0 480 24   1    1  1   1   0
## 4963   4963    0 480 24   1    1  1   1   0
## 4964   4964    0 480 24   1    1  1   1   0
## 4965   4965    0 480 24   1    1  1   1   0
## 4966   4966    0 480 24   1    1  1   1   0
## 4967   4967    0 480 24   1    1  1   1   0
## 4968   4968    0 480 24   1    1  1   1   0
## 4969   4969    0 480 24   1    1  1   1   0
## 4970   4970    0 480 24   1    1  1   1   0
## 4971   4971    0 480 24   1    1  1   1   0
## 4972   4972    0 480 24   1    1  1   1   0
## 4973   4973    0 480 24   1    1  1   1   0
## 4974   4974    0 480 24   1    1  1   1   0
## 4975   4975    0 480 24   1    1  1   1   0
## 4976   4976    0 480 24   1    1  1   1   0
## 4977   4977    0 480 24   1    1  1   1   0
## 4978   4978    0 480 24   1    1  1   1   0
## 4979   4979    0 480 24   1    1  1   1   0
## 4980   4980    0 480 24   1    1  1   1   0
## 4981   4981    0 480 24   1    1  1   1   0
## 4982   4982    0 480 24   1    1  1   1   0
## 4983   4983    0 480 24   1    1  1   1   0
## 4984   4984    0 480 24   1    1  1   1   0
## 4985   4985    0 480 24   1    1  1   1   0
## 4986   4986    0 480 24   1    1  1   1   0
## 4987   4987    0 480 24   1    1  1   1   0
## 4988   4988    0 480 24   1    1  1   1   0
## 4989   4989    0 480 24   1    1  1   1   0
## 4990   4990    0 480 24   1    1  1   1   0
## 4991   4991    0 480 24   1    1  1   1   0
## 4992   4992    0 480 24   1    1  1   1   0
## 4993   4993    0 480 24   1    1  1   1   0
## 4994   4994    0 480 24   1    1  1   1   0
## 4995   4995    0 480 24   1    1  1   1   0
## 4996   4996    0 480 24   1    1  1   1   0
## 4997   4997    0 480 24   1    1  1   1   0
## 4998   4998    0 480 24   1    1  1   1   0
## 4999   4999    0 480 24   1    1  1   1   0
## 5000   5000    0 480 24   1    1  1   1   0
## 5001   5001    0 480 24   1    1  1   1   0
## 5002   5002    0 480 24   1    1  1   1   0
## 5003   5003    0 480 24   1    1  1   1   0
## 5004   5004    0 480 24   1    1  1   1   0
## 5005   5005    0 480 24   1    1  1   1   0
## 5006   5006    0 480 24   1    1  1   1   0
## 5007   5007    0 480 24   1    1  1   1   0
## 5008   5008    0 480 24   1    1  1   1   0
## 5009   5009    0 480 24   1    1  1   1   0
## 5010   5010    0 480 24   1    1  1   1   0
## 5011   5011    0 480 24   1    1  1   1   0
## 5012   5012    0 480 24   1    1  1   1   0
## 5013   5013    0 480 24   1    1  1   1   0
## 5014   5014    0 480 24   1    1  1   1   0
## 5015   5015    0 480 24   1    1  1   1   0
## 5016   5016    0 480 24   1    1  1   1   0
## 5017   5017    0 480 24   1    1  1   1   0
## 5018   5018    0 480 24   1    1  1   1   0
## 5019   5019    0 480 24   1    1  1   1   0
## 5020   5020    0 480 24   1    1  1   1   0
## 5021   5021    0 480 24   1    1  1   1   0
## 5022   5022    0 480 24   1    1  1   1   0
## 5023   5023    0 480 24   1    1  1   1   0
## 5024   5024    0 480 24   1    1  1   1   0
## 5025   5025    0 480 24   1    1  1   1   0
## 5026   5026    0 480 24   1    1  1   1   0
## 5027   5027    0 480 24   1    1  1   1   0
## 5028   5028    0 480 24   1    1  1   1   0
## 5029   5029    0 480 24   1    1  1   1   0
## 5030   5030    0 480 24   1    1  1   1   0
## 5031   5031    0 480 24   1    1  1   1   0
## 5032   5032    0 480 24   1    1  1   1   0
## 5033   5033    0 480 24   1    1  1   1   0
## 5034   5034    0 480 24   1    1  1   1   0
## 5035   5035    0 480 24   1    1  1   1   0
## 5036   5036    0 480 24   1    1  1   1   0
## 5037   5037    0 480 24   1    1  1   1   0
## 5038   5038    0 480 24   1    1  1   1   0
## 5039   5039    0 480 24   1    1  1   1   0
## 5040   5040    0 480 24   1    1  1   1   0
## 5041   5041    0 480 24   1    1  1   1   0
## 5042   5042    0 480 24   1    1  1   1   0
## 5043   5043    0 480 24   1    1  1   1   0
## 5044   5044    0 480 24   1    1  1   1   0
## 5045   5045    0 480 24   1    1  1   1   0
## 5046   5046    0 480 24   1    1  1   1   0
## 5047   5047    0 480 24   1    1  1   1   0
## 5048   5048    0 480 24   1    1  1   1   0
## 5049   5049    0 480 24   1    1  1   1   0
## 5050   5050    0 480 24   1    1  1   1   0
## 5051   5051    0 480 24   1    1  1   1   0
## 5052   5052    0 480 24   1    1  1   1   0
## 5053   5053    0 480 24   1    1  1   1   0
## 5054   5054    0 480 24   1    1  1   1   0
## 5055   5055    0 480 24   1    1  1   1   0
## 5056   5056    0 480 24   1    1  1   1   0
## 5057   5057    0 480 24   1    1  1   1   0
## 5058   5058    0 480 24   1    1  1   1   0
## 5059   5059    0 480 24   1    1  1   1   0
## 5060   5060    0 480 24   1    1  1   1   0
## 5061   5061    0 480 24   1    1  1   1   0
## 5062   5062    0 480 24   1    1  1   1   0
## 5063   5063    0 480 24   1    1  1   1   0
## 5064   5064    0 480 24   1    1  1   1   0
## 5065   5065    0 480 24   1    1  1   1   0
## 5066   5066    0 480 24   1    1  1   1   0
## 5067   5067    0 480 24   1    1  1   1   0
## 5068   5068    0 480 24   1    1  1   1   0
## 5069   5069    0 480 24   1    1  1   1   0
## 5070   5070    0 480 24   1    1  1   1   0
## 5071   5071    0 480 24   1    1  1   1   0
## 5072   5072    0 480 24   1    1  1   1   0
## 5073   5073    0 480 24   1    1  1   1   0
## 5074   5074    0 480 24   1    1  1   1   0
## 5075   5075    0 480 24   1    1  1   1   0
## 5076   5076    0 480 24   1    1  1   1   0
## 5077   5077    0 480 24   1    1  1   1   0
## 5078   5078    0 480 24   1    1  1   1   0
## 5079   5079    0 480 24   1    1  1   1   0
## 5080   5080    0 480 24   1    1  1   1   0
## 5081   5081    0 480 24   1    1  1   1   0
## 5082   5082    0 480 24   1    1  1   1   0
## 5083   5083    0 480 24   1    1  1   1   0
## 5084   5084    0 480 24   1    1  1   1   0
## 5085   5085    0 480 24   1    1  1   1   0
## 5086   5086    0 480 24   1    1  1   1   0
## 5087   5087    0 480 24   1    1  1   1   0
## 5088   5088    0 480 24   1    1  1   1   0
## 5089   5089    0 480 24   1    1  1   1   0
## 5090   5090    0 480 24   1    1  1   1   0
## 5091   5091    0 480 24   1    1  1   1   0
## 5092   5092    0 480 24   1    1  1   1   0
## 5093   5093    0 480 24   1    1  1   1   0
## 5094   5094    0 480 24   1    1  1   1   0
## 5095   5095    0 480 24   1    1  1   1   0
## 5096   5096    0 480 24   1    1  1   1   0
## 5097   5097    0 480 24   1    1  1   1   0
## 5098   5098    0 480 24   1    1  1   1   0
## 5099   5099    0 480 24   1    1  1   1   0
## 5100   5100    0 480 24   1    1  1   1   0
## 5101   5101    0 480 24   1    1  1   1   0
## 5102   5102    0 480 24   1    1  1   1   0
## 5103   5103    0 480 24   1    1  1   1   0
## 5104   5104    0 480 24   1    1  1   1   0
## 5105   5105    0 480 24   1    1  1   1   0
## 5106   5106    0 480 24   1    1  1   1   0
## 5107   5107    0 480 24   1    1  1   1   0
## 5108   5108    0 480 24   1    1  1   1   0
## 5109   5109    0 480 24   1    1  1   1   0
## 5110   5110    0 480 24   1    1  1   1   0
## 5111   5111    0 480 24   1    1  1   1   0
## 5112   5112    0 480 24   1    1  1   1   0
## 5113   5113    0 480 24   1    1  1   1   0
## 5114   5114    0 480 24   1    1  1   1   0
## 5115   5115    0 480 24   1    1  1   1   0
## 5116   5116    0 480 24   1    1  1   1   0
## 5117   5117    0 480 24   1    1  1   1   0
## 5118   5118    0 480 24   1    1  1   1   0
## 5119   5119    0 480 24   1    1  1   1   0
## 5120   5120    0 480 24   1    1  1   1   0
## 5121   5121    0 480 24   1    1  1   1   0
## 5122   5122    0 480 24   1    1  1   1   0
## 5123   5123    0 480 24   1    1  1   1   0
## 5124   5124    0 480 24   1    1  1   1   0
## 5125   5125    0 480 24   1    1  1   1   0
## 5126   5126    0 480 24   1    1  1   1   0
## 5127   5127    0 480 24   1    1  1   1   0
## 5128   5128    0 480 24   1    1  1   1   0
## 5129   5129    0 480 24   1    1  1   1   0
## 5130   5130    0 480 24   1    1  1   1   0
## 5131   5131    0 480 24   1    1  1   1   0
## 5132   5132    0 480 24   1    1  1   1   0
## 5133   5133    0 480 24   1    1  1   1   0
## 5134   5134    0 480 24   1    1  1   1   0
## 5135   5135    0 480 24   1    1  1   1   0
## 5136   5136    0 480 24   1    1  1   1   0
## 5137   5137    0 480 24   1    1  1   1   0
## 5138   5138    0 480 24   1    1  1   1   0
## 5139   5139    0 480 24   1    1  1   1   0
## 5140   5140    0 480 24   1    1  1   1   0
## 5141   5141    0 480 24   1    1  1   1   0
## 5142   5142    0 480 24   1    1  1   1   0
## 5143   5143    0 480 24   1    1  1   1   0
## 5144   5144    0 480 24   1    1  1   1   0
## 5145   5145    0 480 24   1    1  1   1   0
## 5146   5146    0 480 24   1    1  1   1   0
## 5147   5147    0 480 24   1    1  1   1   0
## 5148   5148    0 480 24   1    1  1   1   0
## 5149   5149    0 480 24   1    1  1   1   0
## 5150   5150    0 480 24   1    1  1   1   0
## 5151   5151    0 480 24   1    1  1   1   0
## 5152   5152    0 480 24   1    1  1   1   0
## 5153   5153    0 480 24   1    1  1   1   0
## 5154   5154    0 480 24   1    1  1   1   0
## 5155   5155    0 480 24   1    1  1   1   0
## 5156   5156    0 480 24   1    1  1   1   0
## 5157   5157    0 480 24   1    1  1   1   0
## 5158   5158    0 480 24   1    1  1   1   0
## 5159   5159    0 480 24   1    1  1   1   0
## 5160   5160    0 480 24   1    1  1   1   0
## 5161   5161    0 480 24   1    1  1   1   0
## 5162   5162    0 480 24   1    1  1   1   0
## 5163   5163    0 480 24   1    1  1   1   0
## 5164   5164    0 480 24   1    1  1   1   0
## 5165   5165    0 480 24   1    1  1   1   0
## 5166   5166    0 480 24   1    1  1   1   0
## 5167   5167    0 480 24   1    1  1   1   0
## 5168   5168    0 480 24   1    1  1   1   0
## 5169   5169    0 480 24   1    1  1   1   0
## 5170   5170    0 480 24   1    1  1   1   0
## 5171   5171    0 480 24   1    1  1   1   0
## 5172   5172    0 480 24   1    1  1   1   0
## 5173   5173    0 480 24   1    1  1   1   0
## 5174   5174    0 480 24   1    1  1   1   0
## 5175   5175    0 480 24   1    1  1   1   0
## 5176   5176    0 480 24   1    1  1   1   0
## 5177   5177    0 480 24   1    1  1   1   0
## 5178   5178    0 480 24   1    1  1   1   0
## 5179   5179    0 480 24   1    1  1   1   0
## 5180   5180    0 480 24   1    1  1   1   0
## 5181   5181    0 480 24   1    1  1   1   0
## 5182   5182    0 480 24   1    1  1   1   0
## 5183   5183    0 480 24   1    1  1   1   0
## 5184   5184    0 480 24   1    1  1   1   0
## 5185   5185    0 480 24   1    1  1   1   0
## 5186   5186    0 480 24   1    1  1   1   0
## 5187   5187    0 480 24   1    1  1   1   0
## 5188   5188    0 480 24   1    1  1   1   0
## 5189   5189    0 480 24   1    1  1   1   0
## 5190   5190    0 480 24   1    1  1   1   0
## 5191   5191    0 480 24   1    1  1   1   0
## 5192   5192    0 480 24   1    1  1   1   0
## 5193   5193    0 480 24   1    1  1   1   0
## 5194   5194    0 480 24   1    1  1   1   0
## 5195   5195    0 480 24   1    1  1   1   0
## 5196   5196    0 480 24   1    1  1   1   0
## 5197   5197    0 480 24   1    1  1   1   0
## 5198   5198    0 480 24   1    1  1   1   0
## 5199   5199    0 480 24   1    1  1   1   0
## 5200   5200    0 480 24   1    1  1   1   0
## 5201   5201    0 480 24   1    1  1   1   0
## 5202   5202    0 480 24   1    1  1   1   0
## 5203   5203    0 480 24   1    1  1   1   0
## 5204   5204    0 480 24   1    1  1   1   0
## 5205   5205    0 480 24   1    1  1   1   0
## 5206   5206    0 480 24   1    1  1   1   0
## 5207   5207    0 480 24   1    1  1   1   0
## 5208   5208    0 480 24   1    1  1   1   0
## 5209   5209    0 480 24   1    1  1   1   0
## 5210   5210    0 480 24   1    1  1   1   0
## 5211   5211    0 480 24   1    1  1   1   0
## 5212   5212    0 480 24   1    1  1   1   0
## 5213   5213    0 480 24   1    1  1   1   0
## 5214   5214    0 480 24   1    1  1   1   0
## 5215   5215    0 480 24   1    1  1   1   0
## 5216   5216    0 480 24   1    1  1   1   0
## 5217   5217    0 480 24   1    1  1   1   0
## 5218   5218    0 480 24   1    1  1   1   0
## 5219   5219    0 480 24   1    1  1   1   0
## 5220   5220    0 480 24   1    1  1   1   0
## 5221   5221    0 480 24   1    1  1   1   0
## 5222   5222    0 480 24   1    1  1   1   0
## 5223   5223    0 480 24   1    1  1   1   0
## 5224   5224    0 480 24   1    1  1   1   0
## 5225   5225    0 480 24   1    1  1   1   0
## 5226   5226    0 480 24   1    1  1   1   0
## 5227   5227    0 480 24   1    1  1   1   0
## 5228   5228    0 480 24   1    1  1   1   0
## 5229   5229    0 480 24   1    1  1   1   0
## 5230   5230    0 480 24   1    1  1   1   0
## 5231   5231    0 480 24   1    1  1   1   0
## 5232   5232    0 480 24   1    1  1   1   0
## 5233   5233    0 480 24   1    1  1   1   0
## 5234   5234    0 480 24   1    1  1   1   0
## 5235   5235    0 480 24   1    1  1   1   0
## 5236   5236    0 480 24   1    1  1   1   0
## 5237   5237    0 480 24   1    1  1   1   0
## 5238   5238    0 480 24   1    1  1   1   0
## 5239   5239    0 480 24   1    1  1   1   0
## 5240   5240    0 480 24   1    1  1   1   0
## 5241   5241    0 480 24   1    1  1   1   0
## 5242   5242    0 480 24   1    1  1   1   0
## 5243   5243    0 480 24   1    1  1   1   0
## 5244   5244    0 480 24   1    1  1   1   0
## 5245   5245    0 480 24   1    1  1   1   0
## 5246   5246    0 480 24   1    1  1   1   0
## 5247   5247    0 480 24   1    1  1   1   0
## 5248   5248    0 480 24   1    1  1   1   0
## 5249   5249    0 480 24   1    1  1   1   0
## 5250   5250    0 480 24   1    1  1   1   0
## 5251   5251    0 480 24   1    1  1   1   0
## 5252   5252    0 480 24   1    1  1   1   0
## 5253   5253    0 480 24   1    1  1   1   0
## 5254   5254    0 480 24   1    1  1   1   0
## 5255   5255    0 480 24   1    1  1   1   0
## 5256   5256    0 480 24   1    1  1   1   0
## 5257   5257    0 480 24   1    1  1   1   0
## 5258   5258    0 480 24   1    1  1   1   0
## 5259   5259    0 480 24   1    1  1   1   0
## 5260   5260    0 480 24   1    1  1   1   0
## 5261   5261    0 480 24   1    1  1   1   0
## 5262   5262    0 480 24   1    1  1   1   0
## 5263   5263    0 480 24   1    1  1   1   0
## 5264   5264    0 480 24   1    1  1   1   0
## 5265   5265    0 480 24   1    1  1   1   0
## 5266   5266    0 480 24   1    1  1   1   0
## 5267   5267    0 480 24   1    1  1   1   0
## 5268   5268    0 480 24   1    1  1   1   0
## 5269   5269    0 480 24   1    1  1   1   0
## 5270   5270    0 480 24   1    1  1   1   0
## 5271   5271    0 480 24   1    1  1   1   0
## 5272   5272    0 480 24   1    1  1   1   0
## 5273   5273    0 480 24   1    1  1   1   0
## 5274   5274    0 480 24   1    1  1   1   0
## 5275   5275    0 480 24   1    1  1   1   0
## 5276   5276    0 480 24   1    1  1   1   0
## 5277   5277    0 480 24   1    1  1   1   0
## 5278   5278    0 480 24   1    1  1   1   0
## 5279   5279    0 480 24   1    1  1   1   0
## 5280   5280    0 480 24   1    1  1   1   0
## 5281   5281    0 480 24   1    1  1   1   0
## 5282   5282    0 480 24   1    1  1   1   0
## 5283   5283    0 480 24   1    1  1   1   0
## 5284   5284    0 480 24   1    1  1   1   0
## 5285   5285    0 480 24   1    1  1   1   0
## 5286   5286    0 480 24   1    1  1   1   0
## 5287   5287    0 480 24   1    1  1   1   0
## 5288   5288    0 480 24   1    1  1   1   0
## 5289   5289    0 480 24   1    1  1   1   0
## 5290   5290    0 480 24   1    1  1   1   0
## 5291   5291    0 480 24   1    1  1   1   0
## 5292   5292    0 480 24   1    1  1   1   0
## 5293   5293    0 480 24   1    1  1   1   0
## 5294   5294    0 480 24   1    1  1   1   0
## 5295   5295    0 480 24   1    1  1   1   0
## 5296   5296    0 480 24   1    1  1   1   0
## 5297   5297    0 480 24   1    1  1   1   0
## 5298   5298    0 480 24   1    1  1   1   0
## 5299   5299    0 480 24   1    1  1   1   0
## 5300   5300    0 480 24   1    1  1   1   0
## 5301   5301    0 480 24   1    1  1   1   0
## 5302   5302    0 480 24   1    1  1   1   0
## 5303   5303    0 480 24   1    1  1   1   0
## 5304   5304    0 480 24   1    1  1   1   0
## 5305   5305    0 480 24   1    1  1   1   0
## 5306   5306    0 480 24   1    1  1   1   0
## 5307   5307    0 480 24   1    1  1   1   0
## 5308   5308    0 480 24   1    1  1   1   0
## 5309   5309    0 480 24   1    1  1   1   0
## 5310   5310    0 480 24   1    1  1   1   0
## 5311   5311    0 480 24   1    1  1   1   0
## 5312   5312    0 480 24   1    1  1   1   0
## 5313   5313    0 480 24   1    1  1   1   0
## 5314   5314    0 480 24   1    1  1   1   0
## 5315   5315    0 480 24   1    1  1   1   0
## 5316   5316    0 480 24   1    1  1   1   0
## 5317   5317    0 480 24   1    1  1   1   0
## 5318   5318    0 480 24   1    1  1   1   0
## 5319   5319    0 480 24   1    1  1   1   0
## 5320   5320    0 480 24   1    1  1   1   0
## 5321   5321    0 480 24   1    1  1   1   0
## 5322   5322    0 480 24   1    1  1   1   0
## 5323   5323    0 480 24   1    1  1   1   0
## 5324   5324    0 480 24   1    1  1   1   0
## 5325   5325    0 480 24   1    1  1   1   0
## 5326   5326    0 480 24   1    1  1   1   0
## 5327   5327    0 480 24   1    1  1   1   0
## 5328   5328    0 480 24   1    1  1   1   0
## 5329   5329    0 480 24   1    1  1   1   0
## 5330   5330    0 480 24   1    1  1   1   0
## 5331   5331    0 480 24   1    1  1   1   0
## 5332   5332    0 480 24   1    1  1   1   0
## 5333   5333    0 480 24   1    1  1   1   0
## 5334   5334    0 480 24   1    1  1   1   0
## 5335   5335    0 480 24   1    1  1   1   0
## 5336   5336    0 480 24   1    1  1   1   0
## 5337   5337    0 480 24   1    1  1   1   0
## 5338   5338    0 480 24   1    1  1   1   0
## 5339   5339    0 480 24   1    1  1   1   0
## 5340   5340    0 480 24   1    1  1   1   0
## 5341   5341    0 480 24   1    1  1   1   0
## 5342   5342    0 480 24   1    1  1   1   0
## 5343   5343    0 480 24   1    1  1   1   0
## 5344   5344    0 480 24   1    1  1   1   0
## 5345   5345    0 480 24   1    1  1   1   0
## 5346   5346    0 480 24   1    1  1   1   0
## 5347   5347    0 480 24   1    1  1   1   0
## 5348   5348    0 480 24   1    1  1   1   0
## 5349   5349    0 480 24   1    1  1   1   0
## 5350   5350    0 480 24   1    1  1   1   0
## 5351   5351    0 480 24   1    1  1   1   0
## 5352   5352    0 480 24   1    1  1   1   0
## 5353   5353    0 480 24   1    1  1   1   0
## 5354   5354    0 480 24   1    1  1   1   0
## 5355   5355    0 480 24   1    1  1   1   0
## 5356   5356    0 480 24   1    1  1   1   0
## 5357   5357    0 480 24   1    1  1   1   0
## 5358   5358    0 480 24   1    1  1   1   0
## 5359   5359    0 480 24   1    1  1   1   0
## 5360   5360    0 480 24   1    1  1   1   0
## 5361   5361    0 480 24   1    1  1   1   0
## 5362   5362    0 480 24   1    1  1   1   0
## 5363   5363    0 480 24   1    1  1   1   0
## 5364   5364    0 480 24   1    1  1   1   0
## 5365   5365    0 480 24   1    1  1   1   0
## 5366   5366    0 480 24   1    1  1   1   0
## 5367   5367    0 480 24   1    1  1   1   0
## 5368   5368    0 480 24   1    1  1   1   0
## 5369   5369    0 480 24   1    1  1   1   0
## 5370   5370    0 480 24   1    1  1   1   0
## 5371   5371    0 480 24   1    1  1   1   0
## 5372   5372    0 480 24   1    1  1   1   0
## 5373   5373    0 480 24   1    1  1   1   0
## 5374   5374    0 480 24   1    1  1   1   0
## 5375   5375    0 480 24   1    1  1   1   0
## 5376   5376    0 480 24   1    1  1   1   0
## 5377   5377    0 480 24   1    1  1   1   0
## 5378   5378    0 480 24   1    1  1   1   0
## 5379   5379    0 480 24   1    1  1   1   0
## 5380   5380    0 480 24   1    1  1   1   0
## 5381   5381    0 480 24   1    1  1   1   0
## 5382   5382    0 480 24   1    1  1   1   0
## 5383   5383    0 480 24   1    1  1   1   0
## 5384   5384    0 480 24   1    1  1   1   0
## 5385   5385    0 480 24   1    1  1   1   0
## 5386   5386    0 480 24   1    1  1   1   0
## 5387   5387    0 480 24   1    1  1   1   0
## 5388   5388    0 480 24   1    1  1   1   0
## 5389   5389    0 480 24   1    1  1   1   0
## 5390   5390    0 480 24   1    1  1   1   0
## 5391   5391    0 480 24   1    1  1   1   0
## 5392   5392    0 480 24   1    1  1   1   0
## 5393   5393    0 480 24   1    1  1   1   0
## 5394   5394    0 480 24   1    1  1   1   0
## 5395   5395    0 480 24   1    1  1   1   0
## 5396   5396    0 480 24   1    1  1   1   0
## 5397   5397    0 480 24   1    1  1   1   0
## 5398   5398    0 480 24   1    1  1   1   0
## 5399   5399    0 480 24   1    1  1   1   0
## 5400   5400    0 480 24   1    1  1   1   0
## 5401   5401    0 480 24   1    1  1   1   0
## 5402   5402    0 480 24   1    1  1   1   0
## 5403   5403    0 480 24   1    1  1   1   0
## 5404   5404    0 480 24   1    1  1   1   0
## 5405   5405    0 480 24   1    1  1   1   0
## 5406   5406    0 480 24   1    1  1   1   0
## 5407   5407    0 480 24   1    1  1   1   0
## 5408   5408    0 480 24   1    1  1   1   0
## 5409   5409    0 480 24   1    1  1   1   0
## 5410   5410    0 480 24   1    1  1   1   0
## 5411   5411    0 480 24   1    1  1   1   0
## 5412   5412    0 480 24   1    1  1   1   0
## 5413   5413    0 480 24   1    1  1   1   0
## 5414   5414    0 480 24   1    1  1   1   0
## 5415   5415    0 480 24   1    1  1   1   0
## 5416   5416    0 480 24   1    1  1   1   0
## 5417   5417    0 480 24   1    1  1   1   0
## 5418   5418    0 480 24   1    1  1   1   0
## 5419   5419    0 480 24   1    1  1   1   0
## 5420   5420    0 480 24   1    1  1   1   0
## 5421   5421    0 480 24   1    1  1   1   0
## 5422   5422    0 480 24   1    1  1   1   0
## 5423   5423    0 480 24   1    1  1   1   0
## 5424   5424    0 480 24   1    1  1   1   0
## 5425   5425    0 480 24   1    1  1   1   0
## 5426   5426    0 480 24   1    1  1   1   0
## 5427   5427    0 480 24   1    1  1   1   0
## 5428   5428    0 480 24   1    1  1   1   0
## 5429   5429    0 480 24   1    1  1   1   0
## 5430   5430    0 480 24   1    1  1   1   0
## 5431   5431    0 480 24   1    1  1   1   0
## 5432   5432    0 480 24   1    1  1   1   0
## 5433   5433    0 480 24   1    1  1   1   0
## 5434   5434    0 480 24   1    1  1   1   0
## 5435   5435    0 480 24   1    1  1   1   0
## 5436   5436    0 480 24   1    1  1   1   0
## 5437   5437    0 480 24   1    1  1   1   0
## 5438   5438    0 480 24   1    1  1   1   0
## 5439   5439    0 480 24   1    1  1   1   0
## 5440   5440    0 480 24   1    1  1   1   0
## 5441   5441    0 480 24   1    1  1   1   0
## 5442   5442    0 480 24   1    1  1   1   0
## 5443   5443    0 480 24   1    1  1   1   0
## 5444   5444    0 480 24   1    1  1   1   0
## 5445   5445    0 480 24   1    1  1   1   0
## 5446   5446    0 480 24   1    1  1   1   0
## 5447   5447    0 480 24   1    1  1   1   0
## 5448   5448    0 480 24   1    1  1   1   0
## 5449   5449    0 480 24   1    1  1   1   0
## 5450   5450    0 480 24   1    1  1   1   0
## 5451   5451    0 480 24   1    1  1   1   0
## 5452   5452    0 480 24   1    1  1   1   0
## 5453   5453    0 480 24   1    1  1   1   0
## 5454   5454    0 480 24   1    1  1   1   0
## 5455   5455    0 480 24   1    1  1   1   0
## 5456   5456    0 480 24   1    1  1   1   0
## 5457   5457    0 480 24   1    1  1   1   0
## 5458   5458    0 480 24   1    1  1   1   0
## 5459   5459    0 480 24   1    1  1   1   0
## 5460   5460    0 480 24   1    1  1   1   0
## 5461   5461    0 480 24   1    1  1   1   0
## 5462   5462    0 480 24   1    1  1   1   0
## 5463   5463    0 480 24   1    1  1   1   0
## 5464   5464    0 480 24   1    1  1   1   0
## 5465   5465    0 480 24   1    1  1   1   0
## 5466   5466    0 480 24   1    1  1   1   0
## 5467   5467    0 480 24   1    1  1   1   0
## 5468   5468    0 480 24   1    1  1   1   0
## 5469   5469    0 480 24   1    1  1   1   0
## 5470   5470    0 480 24   1    1  1   1   0
## 5471   5471    0 480 24   1    1  1   1   0
## 5472   5472    0 480 24   1    1  1   1   0
## 5473   5473    0 480 24   1    1  1   1   0
## 5474   5474    0 480 24   1    1  1   1   0
## 5475   5475    0 480 24   1    1  1   1   0
## 5476   5476    0 480 24   1    1  1   1   0
## 5477   5477    0 480 24   1    1  1   1   0
## 5478   5478    0 480 24   1    1  1   1   0
## 5479   5479    0 480 24   1    1  1   1   0
## 5480   5480    0 480 24   1    1  1   1   0
## 5481   5481    0 480 24   1    1  1   1   0
## 5482   5482    0 480 24   1    1  1   1   0
## 5483   5483    0 480 24   1    1  1   1   0
## 5484   5484    0 480 24   1    1  1   1   0
## 5485   5485    0 480 24   1    1  1   1   0
## 5486   5486    0 480 24   1    1  1   1   0
## 5487   5487    0 480 24   1    1  1   1   0
## 5488   5488    0 480 24   1    1  1   1   0
## 5489   5489    0 480 24   1    1  1   1   0
## 5490   5490    0 480 24   1    1  1   1   0
## 5491   5491    0 480 24   1    1  1   1   0
## 5492   5492    0 480 24   1    1  1   1   0
## 5493   5493    0 480 24   1    1  1   1   0
## 5494   5494    0 480 24   1    1  1   1   0
## 5495   5495    0 480 24   1    1  1   1   0
## 5496   5496    0 480 24   1    1  1   1   0
## 5497   5497    0 480 24   1    1  1   1   0
## 5498   5498    0 480 24   1    1  1   1   0
## 5499   5499    0 480 24   1    1  1   1   0
## 5500   5500    0 480 24   1    1  1   1   0
## 5501   5501    0 480 24   1    1  1   1   0
## 5502   5502    0 480 24   1    1  1   1   0
## 5503   5503    0 480 24   1    1  1   1   0
## 5504   5504    0 480 24   1    1  1   1   0
## 5505   5505    0 480 24   1    1  1   1   0
## 5506   5506    0 480 24   1    1  1   1   0
## 5507   5507    0 480 24   1    1  1   1   0
## 5508   5508    0 480 24   1    1  1   1   0
## 5509   5509    0 480 24   1    1  1   1   0
## 5510   5510    0 480 24   1    1  1   1   0
## 5511   5511    0 480 24   1    1  1   1   0
## 5512   5512    0 480 24   1    1  1   1   0
## 5513   5513    0 480 24   1    1  1   1   0
## 5514   5514    0 480 24   1    1  1   1   0
## 5515   5515    0 480 24   1    1  1   1   0
## 5516   5516    0 480 24   1    1  1   1   0
## 5517   5517    0 480 24   1    1  1   1   0
## 5518   5518    0 480 24   1    1  1   1   0
## 5519   5519    0 480 24   1    1  1   1   0
## 5520   5520    0 480 24   1    1  1   1   0
## 5521   5521    0 480 24   1    1  1   1   0
## 5522   5522    0 480 24   1    1  1   1   0
## 5523   5523    0 480 24   1    1  1   1   0
## 5524   5524    0 480 24   1    1  1   1   0
## 5525   5525    0 480 24   1    1  1   1   0
## 5526   5526    0 480 24   1    1  1   1   0
## 5527   5527    0 480 24   1    1  1   1   0
## 5528   5528    0 480 24   1    1  1   1   0
## 5529   5529    0 480 24   1    1  1   1   0
## 5530   5530    0 480 24   1    1  1   1   0
## 5531   5531    0 480 24   1    1  1   1   0
## 5532   5532    0 480 24   1    1  1   1   0
## 5533   5533    0 480 24   1    1  1   1   0
## 5534   5534    0 480 24   1    1  1   1   0
## 5535   5535    0 480 24   1    1  1   1   0
## 5536   5536    0 480 24   1    1  1   1   0
## 5537   5537    0 480 24   1    1  1   1   0
## 5538   5538    0 480 24   1    1  1   1   0
## 5539   5539    0 480 24   1    1  1   1   0
## 5540   5540    0 480 24   1    1  1   1   0
## 5541   5541    0 480 24   1    1  1   1   0
## 5542   5542    0 480 24   1    1  1   1   0
## 5543   5543    0 480 24   1    1  1   1   0
## 5544   5544    0 480 24   1    1  1   1   0
## 5545   5545    0 480 24   1    1  1   1   0
## 5546   5546    0 480 24   1    1  1   1   0
## 5547   5547    0 480 24   1    1  1   1   0
## 5548   5548    0 480 24   1    1  1   1   0
## 5549   5549    0 480 24   1    1  1   1   0
## 5550   5550    0 480 24   1    1  1   1   0
## 5551   5551    0 480 24   1    1  1   1   0
## 5552   5552    0 480 24   1    1  1   1   0
## 5553   5553    0 480 24   1    1  1   1   0
## 5554   5554    0 480 24   1    1  1   1   0
## 5555   5555    0 480 24   1    1  1   1   0
## 5556   5556    0 480 24   1    1  1   1   0
## 5557   5557    0 480 24   1    1  1   1   0
## 5558   5558    0 480 24   1    1  1   1   0
## 5559   5559    0 480 24   1    1  1   1   0
## 5560   5560    0 480 24   1    1  1   1   0
## 5561   5561    0 480 24   1    1  1   1   0
## 5562   5562    0 480 24   1    1  1   1   0
## 5563   5563    0 480 24   1    1  1   1   0
## 5564   5564    0 480 24   1    1  1   1   0
## 5565   5565    0 480 24   1    1  1   1   0
## 5566   5566    0 480 24   1    1  1   1   0
## 5567   5567    0 480 24   1    1  1   1   0
## 5568   5568    0 480 24   1    1  1   1   0
## 5569   5569    0 480 24   1    1  1   1   0
## 5570   5570    0 480 24   1    1  1   1   0
## 5571   5571    0 480 24   1    1  1   1   0
## 5572   5572    0 480 24   1    1  1   1   0
## 5573   5573    0 480 24   1    1  1   1   0
## 5574   5574    0 480 24   1    1  1   1   0
## 5575   5575    0 480 24   1    1  1   1   0
## 5576   5576    0 480 24   1    1  1   1   0
## 5577   5577    0 480 24   1    1  1   1   0
## 5578   5578    0 480 24   1    1  1   1   0
## 5579   5579    0 480 24   1    1  1   1   0
## 5580   5580    0 480 24   1    1  1   1   0
## 5581   5581    0 480 24   1    1  1   1   0
## 5582   5582    0 480 24   1    1  1   1   0
## 5583   5583    0 480 24   1    1  1   1   0
## 5584   5584    0 480 24   1    1  1   1   0
## 5585   5585    0 480 24   1    1  1   1   0
## 5586   5586    0 480 24   1    1  1   1   0
## 5587   5587    0 480 24   1    1  1   1   0
## 5588   5588    0 480 24   1    1  1   1   0
## 5589   5589    0 480 24   1    1  1   1   0
## 5590   5590    0 480 24   1    1  1   1   0
## 5591   5591    0 480 24   1    1  1   1   0
## 5592   5592    0 480 24   1    1  1   1   0
## 5593   5593    0 480 24   1    1  1   1   0
## 5594   5594    0 480 24   1    1  1   1   0
## 5595   5595    0 480 24   1    1  1   1   0
## 5596   5596    0 480 24   1    1  1   1   0
## 5597   5597    0 480 24   1    1  1   1   0
## 5598   5598    0 480 24   1    1  1   1   0
## 5599   5599    0 480 24   1    1  1   1   0
## 5600   5600    0 480 24   1    1  1   1   0
## 5601   5601    0 480 24   1    1  1   1   0
## 5602   5602    0 480 24   1    1  1   1   0
## 5603   5603    0 480 24   1    1  1   1   0
## 5604   5604    0 480 24   1    1  1   1   0
## 5605   5605    0 480 24   1    1  1   1   0
## 5606   5606    0 480 24   1    1  1   1   0
## 5607   5607    0 480 24   1    1  1   1   0
## 5608   5608    0 480 24   1    1  1   1   0
## 5609   5609    0 480 24   1    1  1   1   0
## 5610   5610    0 480 24   1    1  1   1   0
## 5611   5611    0 480 24   1    1  1   1   0
## 5612   5612    0 480 24   1    1  1   1   0
## 5613   5613    0 480 24   1    1  1   1   0
## 5614   5614    0 480 24   1    1  1   1   0
## 5615   5615    0 480 24   1    1  1   1   0
## 5616   5616    0 480 24   1    1  1   1   0
## 5617   5617    0 480 24   1    1  1   1   0
## 5618   5618    0 480 24   1    1  1   1   0
## 5619   5619    0 480 24   1    1  1   1   0
## 5620   5620    0 480 24   1    1  1   1   0
## 5621   5621    0 480 24   1    1  1   1   0
## 5622   5622    0 480 24   1    1  1   1   0
## 5623   5623    0 480 24   1    1  1   1   0
## 5624   5624    0 480 24   1    1  1   1   0
## 5625   5625    0 480 24   1    1  1   1   0
## 5626   5626    0 480 24   1    1  1   1   0
## 5627   5627    0 480 24   1    1  1   1   0
## 5628   5628    0 480 24   1    1  1   1   0
## 5629   5629    0 480 24   1    1  1   1   0
## 5630   5630    0 480 24   1    1  1   1   0
## 5631   5631    0 480 24   1    1  1   1   0
## 5632   5632    0 480 24   1    1  1   1   0
## 5633   5633    0 480 24   1    1  1   1   0
## 5634   5634    0 480 24   1    1  1   1   0
## 5635   5635    0 480 24   1    1  1   1   0
## 5636   5636    0 480 24   1    1  1   1   0
## 5637   5637    0 480 24   1    1  1   1   0
## 5638   5638    0 480 24   1    1  1   1   0
## 5639   5639    0 480 24   1    1  1   1   0
## 5640   5640    0 480 24   1    1  1   1   0
## 5641   5641    0 480 24   1    1  1   1   0
## 5642   5642    0 480 24   1    1  1   1   0
## 5643   5643    0 480 24   1    1  1   1   0
## 5644   5644    0 480 24   1    1  1   1   0
## 5645   5645    0 480 24   1    1  1   1   0
## 5646   5646    0 480 24   1    1  1   1   0
## 5647   5647    0 480 24   1    1  1   1   0
## 5648   5648    0 480 24   1    1  1   1   0
## 5649   5649    0 480 24   1    1  1   1   0
## 5650   5650    0 480 24   1    1  1   1   0
## 5651   5651    0 480 24   1    1  1   1   0
## 5652   5652    0 480 24   1    1  1   1   0
## 5653   5653    0 480 24   1    1  1   1   0
## 5654   5654    0 480 24   1    1  1   1   0
## 5655   5655    0 480 24   1    1  1   1   0
## 5656   5656    0 480 24   1    1  1   1   0
## 5657   5657    0 480 24   1    1  1   1   0
## 5658   5658    0 480 24   1    1  1   1   0
## 5659   5659    0 480 24   1    1  1   1   0
## 5660   5660    0 480 24   1    1  1   1   0
## 5661   5661    0 480 24   1    1  1   1   0
## 5662   5662    0 480 24   1    1  1   1   0
## 5663   5663    0 480 24   1    1  1   1   0
## 5664   5664    0 480 24   1    1  1   1   0
## 5665   5665    0 480 24   1    1  1   1   0
## 5666   5666    0 480 24   1    1  1   1   0
## 5667   5667    0 480 24   1    1  1   1   0
## 5668   5668    0 480 24   1    1  1   1   0
## 5669   5669    0 480 24   1    1  1   1   0
## 5670   5670    0 480 24   1    1  1   1   0
## 5671   5671    0 480 24   1    1  1   1   0
## 5672   5672    0 480 24   1    1  1   1   0
## 5673   5673    0 480 24   1    1  1   1   0
## 5674   5674    0 480 24   1    1  1   1   0
## 5675   5675    0 480 24   1    1  1   1   0
## 5676   5676    0 480 24   1    1  1   1   0
## 5677   5677    0 480 24   1    1  1   1   0
## 5678   5678    0 480 24   1    1  1   1   0
## 5679   5679    0 480 24   1    1  1   1   0
## 5680   5680    0 480 24   1    1  1   1   0
## 5681   5681    0 480 24   1    1  1   1   0
## 5682   5682    0 480 24   1    1  1   1   0
## 5683   5683    0 480 24   1    1  1   1   0
## 5684   5684    0 480 24   1    1  1   1   0
## 5685   5685    0 480 24   1    1  1   1   0
## 5686   5686    0 480 24   1    1  1   1   0
## 5687   5687    0 480 24   1    1  1   1   0
## 5688   5688    0 480 24   1    1  1   1   0
## 5689   5689    0 480 24   1    1  1   1   0
## 5690   5690    0 480 24   1    1  1   1   0
## 5691   5691    0 480 24   1    1  1   1   0
## 5692   5692    0 480 24   1    1  1   1   0
## 5693   5693    0 480 24   1    1  1   1   0
## 5694   5694    0 480 24   1    1  1   1   0
## 5695   5695    0 480 24   1    1  1   1   0
## 5696   5696    0 480 24   1    1  1   1   0
## 5697   5697    0 480 24   1    1  1   1   0
## 5698   5698    0 480 24   1    1  1   1   0
## 5699   5699    0 480 24   1    1  1   1   0
## 5700   5700    0 480 24   1    1  1   1   0
## 5701   5701    0 480 24   1    1  1   1   0
## 5702   5702    0 480 24   1    1  1   1   0
## 5703   5703    0 480 24   1    1  1   1   0
## 5704   5704    0 480 24   1    1  1   1   0
## 5705   5705    0 480 24   1    1  1   1   0
## 5706   5706    0 480 24   1    1  1   1   0
## 5707   5707    0 480 24   1    1  1   1   0
## 5708   5708    0 480 24   1    1  1   1   0
## 5709   5709    0 480 24   1    1  1   1   0
## 5710   5710    0 480 24   1    1  1   1   0
## 5711   5711    0 480 24   1    1  1   1   0
## 5712   5712    0 480 24   1    1  1   1   0
## 5713   5713    0 480 24   1    1  1   1   0
## 5714   5714    0 480 24   1    1  1   1   0
## 5715   5715    0 480 24   1    1  1   1   0
## 5716   5716    0 480 24   1    1  1   1   0
## 5717   5717    0 480 24   1    1  1   1   0
## 5718   5718    0 480 24   1    1  1   1   0
## 5719   5719    0 480 24   1    1  1   1   0
## 5720   5720    0 480 24   1    1  1   1   0
## 5721   5721    0 480 24   1    1  1   1   0
## 5722   5722    0 480 24   1    1  1   1   0
## 5723   5723    0 480 24   1    1  1   1   0
## 5724   5724    0 480 24   1    1  1   1   0
## 5725   5725    0 480 24   1    1  1   1   0
## 5726   5726    0 480 24   1    1  1   1   0
## 5727   5727    0 480 24   1    1  1   1   0
## 5728   5728    0 480 24   1    1  1   1   0
## 5729   5729    0 480 24   1    1  1   1   0
## 5730   5730    0 480 24   1    1  1   1   0
## 5731   5731    0 480 24   1    1  1   1   0
## 5732   5732    0 480 24   1    1  1   1   0
## 5733   5733    0 480 24   1    1  1   1   0
## 5734   5734    0 480 24   1    1  1   1   0
## 5735   5735    0 480 24   1    1  1   1   0
## 5736   5736    0 480 24   1    1  1   1   0
## 5737   5737    0 480 24   1    1  1   1   0
## 5738   5738    0 480 24   1    1  1   1   0
## 5739   5739    0 480 24   1    1  1   1   0
## 5740   5740    0 480 24   1    1  1   1   0
## 5741   5741    0 480 24   1    1  1   1   0
## 5742   5742    0 480 24   1    1  1   1   0
## 5743   5743    0 480 24   1    1  1   1   0
## 5744   5744    0 480 24   1    1  1   1   0
## 5745   5745    0 480 24   1    1  1   1   0
## 5746   5746    0 480 24   1    1  1   1   0
## 5747   5747    0 480 24   1    1  1   1   0
## 5748   5748    0 480 24   1    1  1   1   0
## 5749   5749    0 480 24   1    1  1   1   0
## 5750   5750    0 480 24   1    1  1   1   0
## 5751   5751    0 480 24   1    1  1   1   0
## 5752   5752    0 480 24   1    1  1   1   0
## 5753   5753    0 480 24   1    1  1   1   0
## 5754   5754    0 480 24   1    1  1   1   0
## 5755   5755    0 480 24   1    1  1   1   0
## 5756   5756    0 480 24   1    1  1   1   0
## 5757   5757    0 480 24   1    1  1   1   0
## 5758   5758    0 480 24   1    1  1   1   0
## 5759   5759    0 480 24   1    1  1   1   0
## 5760   5760    0 480 24   1    1  1   1   0
## 5761   5761    0 480 24   1    1  1   1   0
## 5762   5762    0 480 24   1    1  1   1   0
## 5763   5763    0 480 24   1    1  1   1   0
## 5764   5764    0 480 24   1    1  1   1   0
## 5765   5765    0 480 24   1    1  1   1   0
## 5766   5766    0 480 24   1    1  1   1   0
## 5767   5767    0 480 24   1    1  1   1   0
## 5768   5768    0 480 24   1    1  1   1   0
## 5769   5769    0 480 24   1    1  1   1   0
## 5770   5770    0 480 24   1    1  1   1   0
## 5771   5771    0 480 24   1    1  1   1   0
## 5772   5772    0 480 24   1    1  1   1   0
## 5773   5773    0 480 24   1    1  1   1   0
## 5774   5774    0 480 24   1    1  1   1   0
## 5775   5775    0 480 24   1    1  1   1   0
## 5776   5776    0 480 24   1    1  1   1   0
## 5777   5777    0 480 24   1    1  1   1   0
## 5778   5778    0 480 24   1    1  1   1   0
## 5779   5779    0 480 24   1    1  1   1   0
## 5780   5780    0 480 24   1    1  1   1   0
## 5781   5781    0 480 24   1    1  1   1   0
## 5782   5782    0 480 24   1    1  1   1   0
## 5783   5783    0 480 24   1    1  1   1   0
## 5784   5784    0 480 24   1    1  1   1   0
## 5785   5785    0 480 24   1    1  1   1   0
## 5786   5786    0 480 24   1    1  1   1   0
## 5787   5787    0 480 24   1    1  1   1   0
## 5788   5788    0 480 24   1    1  1   1   0
## 5789   5789    0 480 24   1    1  1   1   0
## 5790   5790    0 480 24   1    1  1   1   0
## 5791   5791    0 480 24   1    1  1   1   0
## 5792   5792    0 480 24   1    1  1   1   0
## 5793   5793    0 480 24   1    1  1   1   0
## 5794   5794    0 480 24   1    1  1   1   0
## 5795   5795    0 480 24   1    1  1   1   0
## 5796   5796    0 480 24   1    1  1   1   0
## 5797   5797    0 480 24   1    1  1   1   0
## 5798   5798    0 480 24   1    1  1   1   0
## 5799   5799    0 480 24   1    1  1   1   0
## 5800   5800    0 480 24   1    1  1   1   0
## 5801   5801    0 480 24   1    1  1   1   0
## 5802   5802    0 480 24   1    1  1   1   0
## 5803   5803    0 480 24   1    1  1   1   0
## 5804   5804    0 480 24   1    1  1   1   0
## 5805   5805    0 480 24   1    1  1   1   0
## 5806   5806    0 480 24   1    1  1   1   0
## 5807   5807    0 480 24   1    1  1   1   0
## 5808   5808    0 480 24   1    1  1   1   0
## 5809   5809    0 480 24   1    1  1   1   0
## 5810   5810    0 480 24   1    1  1   1   0
## 5811   5811    0 480 24   1    1  1   1   0
## 5812   5812    0 480 24   1    1  1   1   0
## 5813   5813    0 480 24   1    1  1   1   0
## 5814   5814    0 480 24   1    1  1   1   0
## 5815   5815    0 480 24   1    1  1   1   0
## 5816   5816    0 480 24   1    1  1   1   0
## 5817   5817    0 480 24   1    1  1   1   0
## 5818   5818    0 480 24   1    1  1   1   0
## 5819   5819    0 480 24   1    1  1   1   0
## 5820   5820    0 480 24   1    1  1   1   0
## 5821   5821    0 480 24   1    1  1   1   0
## 5822   5822    0 480 24   1    1  1   1   0
## 5823   5823    0 480 24   1    1  1   1   0
## 5824   5824    0 480 24   1    1  1   1   0
## 5825   5825    0 480 24   1    1  1   1   0
## 5826   5826    0 480 24   1    1  1   1   0
## 5827   5827    0 480 24   1    1  1   1   0
## 5828   5828    0 480 24   1    1  1   1   0
## 5829   5829    0 480 24   1    1  1   1   0
## 5830   5830    0 480 24   1    1  1   1   0
## 5831   5831    0 480 24   1    1  1   1   0
## 5832   5832    0 480 24   1    1  1   1   0
## 5833   5833    0 480 24   1    1  1   1   0
## 5834   5834    0 480 24   1    1  1   1   0
## 5835   5835    0 480 24   1    1  1   1   0
## 5836   5836    0 480 24   1    1  1   1   0
## 5837   5837    0 480 24   1    1  1   1   0
## 5838   5838    0 480 24   1    1  1   1   0
## 5839   5839    0 480 24   1    1  1   1   0
## 5840   5840    0 480 24   1    1  1   1   0
## 5841   5841    0 480 24   1    1  1   1   0
## 5842   5842    0 480 24   1    1  1   1   0
## 5843   5843    0 480 24   1    1  1   1   0
## 5844   5844    0 480 24   1    1  1   1   0
## 5845   5845    0 480 24   1    1  1   1   0
## 5846   5846    0 480 24   1    1  1   1   0
## 5847   5847    0 480 24   1    1  1   1   0
## 5848   5848    0 480 24   1    1  1   1   0
## 5849   5849    0 480 24   1    1  1   1   0
## 5850   5850    0 480 24   1    1  1   1   0
## 5851   5851    0 480 24   1    1  1   1   0
## 5852   5852    0 480 24   1    1  1   1   0
## 5853   5853    0 480 24   1    1  1   1   0
## 5854   5854    0 480 24   1    1  1   1   0
## 5855   5855    0 480 24   1    1  1   1   0
## 5856   5856    0 480 24   1    1  1   1   0
## 5857   5857    0 480 24   1    1  1   1   0
## 5858   5858    0 480 24   1    1  1   1   0
## 5859   5859    0 480 24   1    1  1   1   0
## 5860   5860    0 480 24   1    1  1   1   0
## 5861   5861    0 480 24   1    1  1   1   0
## 5862   5862    0 480 24   1    1  1   1   0
## 5863   5863    0 480 24   1    1  1   1   0
## 5864   5864    0 480 24   1    1  1   1   0
## 5865   5865    0 480 24   1    1  1   1   0
## 5866   5866    0 480 24   1    1  1   1   0
## 5867   5867    0 480 24   1    1  1   1   0
## 5868   5868    0 480 24   1    1  1   1   0
## 5869   5869    0 480 24   1    1  1   1   0
## 5870   5870    0 480 24   1    1  1   1   0
## 5871   5871    0 480 24   1    1  1   1   0
## 5872   5872    0 480 24   1    1  1   1   0
## 5873   5873    0 480 24   1    1  1   1   0
## 5874   5874    0 480 24   1    1  1   1   0
## 5875   5875    0 480 24   1    1  1   1   0
## 5876   5876    0 480 24   1    1  1   1   0
## 5877   5877    0 480 24   1    1  1   1   0
## 5878   5878    0 480 24   1    1  1   1   0
## 5879   5879    0 480 24   1    1  1   1   0
## 5880   5880    0 480 24   1    1  1   1   0
## 5881   5881    0 480 24   1    1  1   1   0
## 5882   5882    0 480 24   1    1  1   1   0
## 5883   5883    0 480 24   1    1  1   1   0
## 5884   5884    0 480 24   1    1  1   1   0
## 5885   5885    0 480 24   1    1  1   1   0
## 5886   5886    0 480 24   1    1  1   1   0
## 5887   5887    0 480 24   1    1  1   1   0
## 5888   5888    0 480 24   1    1  1   1   0
## 5889   5889    0 480 24   1    1  1   1   0
## 5890   5890    0 480 24   1    1  1   1   0
## 5891   5891    0 480 24   1    1  1   1   0
## 5892   5892    0 480 24   1    1  1   1   0
## 5893   5893    0 480 24   1    1  1   1   0
## 5894   5894    0 480 24   1    1  1   1   0
## 5895   5895    0 480 24   1    1  1   1   0
## 5896   5896    0 480 24   1    1  1   1   0
## 5897   5897    0 480 24   1    1  1   1   0
## 5898   5898    0 480 24   1    1  1   1   0
## 5899   5899    0 480 24   1    1  1   1   0
## 5900   5900    0 480 24   1    1  1   1   0
## 5901   5901    0 480 24   1    1  1   1   0
## 5902   5902    0 480 24   1    1  1   1   0
## 5903   5903    0 480 24   1    1  1   1   0
## 5904   5904    0 480 24   1    1  1   1   0
## 5905   5905    0 480 24   1    1  1   1   0
## 5906   5906    0 480 24   1    1  1   1   0
## 5907   5907    0 480 24   1    1  1   1   0
## 5908   5908    0 480 24   1    1  1   1   0
## 5909   5909    0 480 24   1    1  1   1   0
## 5910   5910    0 480 24   1    1  1   1   0
## 5911   5911    0 480 24   1    1  1   1   0
## 5912   5912    0 480 24   1    1  1   1   0
## 5913   5913    0 480 24   1    1  1   1   0
## 5914   5914    0 480 24   1    1  1   1   0
## 5915   5915    0 480 24   1    1  1   1   0
## 5916   5916    0 480 24   1    1  1   1   0
## 5917   5917    0 480 24   1    1  1   1   0
## 5918   5918    0 480 24   1    1  1   1   0
## 5919   5919    0 480 24   1    1  1   1   0
## 5920   5920    0 480 24   1    1  1   1   0
## 5921   5921    0 480 24   1    1  1   1   0
## 5922   5922    0 480 24   1    1  1   1   0
## 5923   5923    0 480 24   1    1  1   1   0
## 5924   5924    0 480 24   1    1  1   1   0
## 5925   5925    0 480 24   1    1  1   1   0
## 5926   5926    0 480 24   1    1  1   1   0
## 5927   5927    0 480 24   1    1  1   1   0
## 5928   5928    0 480 24   1    1  1   1   0
## 5929   5929    0 480 24   1    1  1   1   0
## 5930   5930    0 480 24   1    1  1   1   0
## 5931   5931    0 480 24   1    1  1   1   0
## 5932   5932    0 480 24   1    1  1   1   0
## 5933   5933    0 480 24   1    1  1   1   0
## 5934   5934    0 480 24   1    1  1   1   0
## 5935   5935    0 480 24   1    1  1   1   0
## 5936   5936    0 480 24   1    1  1   1   0
## 5937   5937    0 480 24   1    1  1   1   0
## 5938   5938    0 480 24   1    1  1   1   0
## 5939   5939    0 480 24   1    1  1   1   0
## 5940   5940    0 480 24   1    1  1   1   0
## 5941   5941    0 480 24   1    1  1   1   0
## 5942   5942    0 480 24   1    1  1   1   0
## 5943   5943    0 480 24   1    1  1   1   0
## 5944   5944    0 480 24   1    1  1   1   0
## 5945   5945    0 480 24   1    1  1   1   0
## 5946   5946    0 480 24   1    1  1   1   0
## 5947   5947    0 480 24   1    1  1   1   0
## 5948   5948    0 480 24   1    1  1   1   0
## 5949   5949    0 480 24   1    1  1   1   0
## 5950   5950    0 480 24   1    1  1   1   0
## 5951   5951    0 480 24   1    1  1   1   0
## 5952   5952    0 480 24   1    1  1   1   0
## 5953   5953    0 480 24   1    1  1   1   0
## 5954   5954    0 480 24   1    1  1   1   0
## 5955   5955    0 480 24   1    1  1   1   0
## 5956   5956    0 480 24   1    1  1   1   0
## 5957   5957    0 480 24   1    1  1   1   0
## 5958   5958    0 480 24   1    1  1   1   0
## 5959   5959    0 480 24   1    1  1   1   0
## 5960   5960    0 480 24   1    1  1   1   0
## 5961   5961    0 480 24   1    1  1   1   0
## 5962   5962    0 480 24   1    1  1   1   0
## 5963   5963    0 480 24   1    1  1   1   0
## 5964   5964    0 480 24   1    1  1   1   0
## 5965   5965    0 480 24   1    1  1   1   0
## 5966   5966    0 480 24   1    1  1   1   0
## 5967   5967    0 480 24   1    1  1   1   0
## 5968   5968    0 480 24   1    1  1   1   0
## 5969   5969    0 480 24   1    1  1   1   0
## 5970   5970    0 480 24   1    1  1   1   0
## 5971   5971    0 480 24   1    1  1   1   0
## 5972   5972    0 480 24   1    1  1   1   0
## 5973   5973    0 480 24   1    1  1   1   0
## 5974   5974    0 480 24   1    1  1   1   0
## 5975   5975    0 480 24   1    1  1   1   0
## 5976   5976    0 480 24   1    1  1   1   0
## 5977   5977    0 480 24   1    1  1   1   0
## 5978   5978    0 480 24   1    1  1   1   0
## 5979   5979    0 480 24   1    1  1   1   0
## 5980   5980    0 480 24   1    1  1   1   0
## 5981   5981    0 480 24   1    1  1   1   0
## 5982   5982    0 480 24   1    1  1   1   0
## 5983   5983    0 480 24   1    1  1   1   0
## 5984   5984    0 480 24   1    1  1   1   0
## 5985   5985    0 480 24   1    1  1   1   0
## 5986   5986    0 480 24   1    1  1   1   0
## 5987   5987    0 480 24   1    1  1   1   0
## 5988   5988    0 480 24   1    1  1   1   0
## 5989   5989    0 480 24   1    1  1   1   0
## 5990   5990    0 480 24   1    1  1   1   0
## 5991   5991    0 480 24   1    1  1   1   0
## 5992   5992    0 480 24   1    1  1   1   0
## 5993   5993    0 480 24   1    1  1   1   0
## 5994   5994    0 480 24   1    1  1   1   0
## 5995   5995    0 480 24   1    1  1   1   0
## 5996   5996    0 480 24   1    1  1   1   0
## 5997   5997    0 480 24   1    1  1   1   0
## 5998   5998    0 480 24   1    1  1   1   0
## 5999   5999    0 480 24   1    1  1   1   0
## 6000   6000    0 480 24   1    1  1   1   0
## 6001   6001    0 480 24   1    1  1   1   0
## 6002   6002    0 480 24   1    1  1   1   0
## 6003   6003    0 480 24   1    1  1   1   0
## 6004   6004    0 480 24   1    1  1   1   0
## 6005   6005    0 480 24   1    1  1   1   0
## 6006   6006    0 480 24   1    1  1   1   0
## 6007   6007    0 480 24   1    1  1   1   0
## 6008   6008    0 480 24   1    1  1   1   0
## 6009   6009    0 480 24   1    1  1   1   0
## 6010   6010    0 480 24   1    1  1   1   0
## 6011   6011    0 480 24   1    1  1   1   0
## 6012   6012    0 480 24   1    1  1   1   0
## 6013   6013    0 480 24   1    1  1   1   0
## 6014   6014    0 480 24   1    1  1   1   0
## 6015   6015    0 480 24   1    1  1   1   0
## 6016   6016    0 480 24   1    1  1   1   0
## 6017   6017    0 480 24   1    1  1   1   0
## 6018   6018    0 480 24   1    1  1   1   0
## 6019   6019    0 480 24   1    1  1   1   0
## 6020   6020    0 480 24   1    1  1   1   0
## 6021   6021    0 480 24   1    1  1   1   0
## 6022   6022    0 480 24   1    1  1   1   0
## 6023   6023    0 480 24   1    1  1   1   0
## 6024   6024    0 480 24   1    1  1   1   0
## 6025   6025    0 480 24   1    1  1   1   0
## 6026   6026    0 480 24   1    1  1   1   0
## 6027   6027    0 480 24   1    1  1   1   0
## 6028   6028    0 480 24   1    1  1   1   0
## 6029   6029    0 480 24   1    1  1   1   0
## 6030   6030    0 480 24   1    1  1   1   0
## 6031   6031    0 480 24   1    1  1   1   0
## 6032   6032    0 480 24   1    1  1   1   0
## 6033   6033    0 480 24   1    1  1   1   0
## 6034   6034    0 480 24   1    1  1   1   0
## 6035   6035    0 480 24   1    1  1   1   0
## 6036   6036    0 480 24   1    1  1   1   0
## 6037   6037    0 480 24   1    1  1   1   0
## 6038   6038    0 480 24   1    1  1   1   0
## 6039   6039    0 480 24   1    1  1   1   0
## 6040   6040    0 480 24   1    1  1   1   0
## 6041   6041    0 480 24   1    1  1   1   0
## 6042   6042    0 480 24   1    1  1   1   0
## 6043   6043    0 480 24   1    1  1   1   0
## 6044   6044    0 480 24   1    1  1   1   0
## 6045   6045    0 480 24   1    1  1   1   0
## 6046   6046    0 480 24   1    1  1   1   0
## 6047   6047    0 480 24   1    1  1   1   0
## 6048   6048    0 480 24   1    1  1   1   0
## 6049   6049    0 480 24   1    1  1   1   0
## 6050   6050    0 480 24   1    1  1   1   0
## 6051   6051    0 480 24   1    1  1   1   0
## 6052   6052    0 480 24   1    1  1   1   0
## 6053   6053    0 480 24   1    1  1   1   0
## 6054   6054    0 480 24   1    1  1   1   0
## 6055   6055    0 480 24   1    1  1   1   0
## 6056   6056    0 480 24   1    1  1   1   0
## 6057   6057    0 480 24   1    1  1   1   0
## 6058   6058    0 480 24   1    1  1   1   0
## 6059   6059    0 480 24   1    1  1   1   0
## 6060   6060    0 480 24   1    1  1   1   0
## 6061   6061    0 480 24   1    1  1   1   0
## 6062   6062    0 480 24   1    1  1   1   0
## 6063   6063    0 480 24   1    1  1   1   0
## 6064   6064    0 480 24   1    1  1   1   0
## 6065   6065    0 480 24   1    1  1   1   0
## 6066   6066    0 480 24   1    1  1   1   0
## 6067   6067    0 480 24   1    1  1   1   0
## 6068   6068    0 480 24   1    1  1   1   0
## 6069   6069    0 480 24   1    1  1   1   0
## 6070   6070    0 480 24   1    1  1   1   0
## 6071   6071    0 480 24   1    1  1   1   0
## 6072   6072    0 480 24   1    1  1   1   0
## 6073   6073    0 480 24   1    1  1   1   0
## 6074   6074    0 480 24   1    1  1   1   0
## 6075   6075    0 480 24   1    1  1   1   0
## 6076   6076    0 480 24   1    1  1   1   0
## 6077   6077    0 480 24   1    1  1   1   0
## 6078   6078    0 480 24   1    1  1   1   0
## 6079   6079    0 480 24   1    1  1   1   0
## 6080   6080    0 480 24   1    1  1   1   0
## 6081   6081    0 480 24   1    1  1   1   0
## 6082   6082    0 480 24   1    1  1   1   0
## 6083   6083    0 480 24   1    1  1   1   0
## 6084   6084    0 480 24   1    1  1   1   0
## 6085   6085    0 480 24   1    1  1   1   0
## 6086   6086    0 480 24   1    1  1   1   0
## 6087   6087    0 480 24   1    1  1   1   0
## 6088   6088    0 480 24   1    1  1   1   0
## 6089   6089    0 480 24   1    1  1   1   0
## 6090   6090    0 480 24   1    1  1   1   0
## 6091   6091    0 480 24   1    1  1   1   0
## 6092   6092    0 480 24   1    1  1   1   0
## 6093   6093    0 480 24   1    1  1   1   0
## 6094   6094    0 480 24   1    1  1   1   0
## 6095   6095    0 480 24   1    1  1   1   0
## 6096   6096    0 480 24   1    1  1   1   0
## 6097   6097    0 480 24   1    1  1   1   0
## 6098   6098    0 480 24   1    1  1   1   0
## 6099   6099    0 480 24   1    1  1   1   0
## 6100   6100    0 480 24   1    1  1   1   0
## 6101   6101    0 480 24   1    1  1   1   0
## 6102   6102    0 480 24   1    1  1   1   0
## 6103   6103    0 480 24   1    1  1   1   0
## 6104   6104    0 480 24   1    1  1   1   0
## 6105   6105    0 480 24   1    1  1   1   0
## 6106   6106    0 480 24   1    1  1   1   0
## 6107   6107    0 480 24   1    1  1   1   0
## 6108   6108    0 480 24   1    1  1   1   0
## 6109   6109    0 480 24   1    1  1   1   0
## 6110   6110    0 480 24   1    1  1   1   0
## 6111   6111    0 480 24   1    1  1   1   0
## 6112   6112    0 480 24   1    1  1   1   0
## 6113   6113    0 480 24   1    1  1   1   0
## 6114   6114    0 480 24   1    1  1   1   0
## 6115   6115    0 480 24   1    1  1   1   0
## 6116   6116    0 480 24   1    1  1   1   0
## 6117   6117    0 480 24   1    1  1   1   0
## 6118   6118    0 480 24   1    1  1   1   0
## 6119   6119    0 480 24   1    1  1   1   0
## 6120   6120    0 480 24   1    1  1   1   0
## 6121   6121    0 480 24   1    1  1   1   0
## 6122   6122    0 480 24   1    1  1   1   0
## 6123   6123    0 480 24   1    1  1   1   0
## 6124   6124    0 480 24   1    1  1   1   0
## 6125   6125    0 480 24   1    1  1   1   0
## 6126   6126    0 480 24   1    1  1   1   0
## 6127   6127    0 480 24   1    1  1   1   0
## 6128   6128    0 480 24   1    1  1   1   0
## 6129   6129    0 480 24   1    1  1   1   0
## 6130   6130    0 480 24   1    1  1   1   0
## 6131   6131    0 480 24   1    1  1   1   0
## 6132   6132    0 480 24   1    1  1   1   0
## 6133   6133    0 480 24   1    1  1   1   0
## 6134   6134    0 480 24   1    1  1   1   0
## 6135   6135    0 480 24   1    1  1   1   0
## 6136   6136    0 480 24   1    1  1   1   0
## 6137   6137    0 480 24   1    1  1   1   0
## 6138   6138    0 480 24   1    1  1   1   0
## 6139   6139    0 480 24   1    1  1   1   0
## 6140   6140    0 480 24   1    1  1   1   0
## 6141   6141    0 480 24   1    1  1   1   0
## 6142   6142    0 480 24   1    1  1   1   0
## 6143   6143    0 480 24   1    1  1   1   0
## 6144   6144    0 480 24   1    1  1   1   0
## 6145   6145    0 480 24   1    1  1   1   0
## 6146   6146    0 480 24   1    1  1   1   0
## 6147   6147    0 480 24   1    1  1   1   0
## 6148   6148    0 480 24   1    1  1   1   0
## 6149   6149    0 480 24   1    1  1   1   0
## 6150   6150    0 480 24   1    1  1   1   0
## 6151   6151    0 480 24   1    1  1   1   0
## 6152   6152    0 480 24   1    1  1   1   0
## 6153   6153    0 480 24   1    1  1   1   0
## 6154   6154    0 480 24   1    1  1   1   0
## 6155   6155    0 480 24   1    1  1   1   0
## 6156   6156    0 480 24   1    1  1   1   0
## 6157   6157    0 480 24   1    1  1   1   0
## 6158   6158    0 480 24   1    1  1   1   0
## 6159   6159    0 480 24   1    1  1   1   0
## 6160   6160    0 480 24   1    1  1   1   0
## 6161   6161    0 480 24   1    1  1   1   0
## 6162   6162    0 480 24   1    1  1   1   0
## 6163   6163    0 480 24   1    1  1   1   0
## 6164   6164    0 480 24   1    1  1   1   0
## 6165   6165    0 480 24   1    1  1   1   0
## 6166   6166    0 480 24   1    1  1   1   0
## 6167   6167    0 480 24   1    1  1   1   0
## 6168   6168    0 480 24   1    1  1   1   0
## 6169   6169    0 480 24   1    1  1   1   0
## 6170   6170    0 480 24   1    1  1   1   0
## 6171   6171    0 480 24   1    1  1   1   0
## 6172   6172    0 480 24   1    1  1   1   0
## 6173   6173    0 480 24   1    1  1   1   0
## 6174   6174    0 480 24   1    1  1   1   0
## 6175   6175    0 480 24   1    1  1   1   0
## 6176   6176    0 480 24   1    1  1   1   0
## 6177   6177    0 480 24   1    1  1   1   0
## 6178   6178    0 480 24   1    1  1   1   0
## 6179   6179    0 480 24   1    1  1   1   0
## 6180   6180    0 480 24   1    1  1   1   0
## 6181   6181    0 480 24   1    1  1   1   0
## 6182   6182    0 480 24   1    1  1   1   0
## 6183   6183    0 480 24   1    1  1   1   0
## 6184   6184    0 480 24   1    1  1   1   0
## 6185   6185    0 480 24   1    1  1   1   0
## 6186   6186    0 480 24   1    1  1   1   0
## 6187   6187    0 480 24   1    1  1   1   0
## 6188   6188    0 480 24   1    1  1   1   0
## 6189   6189    0 480 24   1    1  1   1   0
## 6190   6190    0 480 24   1    1  1   1   0
## 6191   6191    0 480 24   1    1  1   1   0
## 6192   6192    0 480 24   1    1  1   1   0
## 6193   6193    0 480 24   1    1  1   1   0
## 6194   6194    0 480 24   1    1  1   1   0
## 6195   6195    0 480 24   1    1  1   1   0
## 6196   6196    0 480 24   1    1  1   1   0
## 6197   6197    0 480 24   1    1  1   1   0
## 6198   6198    0 480 24   1    1  1   1   0
## 6199   6199    0 480 24   1    1  1   1   0
## 6200   6200    0 480 24   1    1  1   1   0
## 6201   6201    0 480 24   1    1  1   1   0
## 6202   6202    0 480 24   1    1  1   1   0
## 6203   6203    0 480 24   1    1  1   1   0
## 6204   6204    0 480 24   1    1  1   1   0
## 6205   6205    0 480 24   1    1  1   1   0
## 6206   6206    0 480 24   1    1  1   1   0
## 6207   6207    0 480 24   1    1  1   1   0
## 6208   6208    0 480 24   1    1  1   1   0
## 6209   6209    0 480 24   1    1  1   1   0
## 6210   6210    0 480 24   1    1  1   1   0
## 6211   6211    0 480 24   1    1  1   1   0
## 6212   6212    0 480 24   1    1  1   1   0
## 6213   6213    0 480 24   1    1  1   1   0
## 6214   6214    0 480 24   1    1  1   1   0
## 6215   6215    0 480 24   1    1  1   1   0
## 6216   6216    0 480 24   1    1  1   1   0
## 6217   6217    0 480 24   1    1  1   1   0
## 6218   6218    0 480 24   1    1  1   1   0
## 6219   6219    0 480 24   1    1  1   1   0
## 6220   6220    0 480 24   1    1  1   1   0
## 6221   6221    0 480 24   1    1  1   1   0
## 6222   6222    0 480 24   1    1  1   1   0
## 6223   6223    0 480 24   1    1  1   1   0
## 6224   6224    0 480 24   1    1  1   1   0
## 6225   6225    0 480 24   1    1  1   1   0
## 6226   6226    0 480 24   1    1  1   1   0
## 6227   6227    0 480 24   1    1  1   1   0
## 6228   6228    0 480 24   1    1  1   1   0
## 6229   6229    0 480 24   1    1  1   1   0
## 6230   6230    0 480 24   1    1  1   1   0
## 6231   6231    0 480 24   1    1  1   1   0
## 6232   6232    0 480 24   1    1  1   1   0
## 6233   6233    0 480 24   1    1  1   1   0
## 6234   6234    0 480 24   1    1  1   1   0
## 6235   6235    0 480 24   1    1  1   1   0
## 6236   6236    0 480 24   1    1  1   1   0
## 6237   6237    0 480 24   1    1  1   1   0
## 6238   6238    0 480 24   1    1  1   1   0
## 6239   6239    0 480 24   1    1  1   1   0
## 6240   6240    0 480 24   1    1  1   1   0
## 6241   6241    0 480 24   1    1  1   1   0
## 6242   6242    0 480 24   1    1  1   1   0
## 6243   6243    0 480 24   1    1  1   1   0
## 6244   6244    0 480 24   1    1  1   1   0
## 6245   6245    0 480 24   1    1  1   1   0
## 6246   6246    0 480 24   1    1  1   1   0
## 6247   6247    0 480 24   1    1  1   1   0
## 6248   6248    0 480 24   1    1  1   1   0
## 6249   6249    0 480 24   1    1  1   1   0
## 6250   6250    0 480 24   1    1  1   1   0
## 6251   6251    0 480 24   1    1  1   1   0
## 6252   6252    0 480 24   1    1  1   1   0
## 6253   6253    0 480 24   1    1  1   1   0
## 6254   6254    0 480 24   1    1  1   1   0
## 6255   6255    0 480 24   1    1  1   1   0
## 6256   6256    0 480 24   1    1  1   1   0
## 6257   6257    0 480 24   1    1  1   1   0
## 6258   6258    0 480 24   1    1  1   1   0
## 6259   6259    0 480 24   1    1  1   1   0
## 6260   6260    0 480 24   1    1  1   1   0
## 6261   6261    0 480 24   1    1  1   1   0
## 6262   6262    0 480 24   1    1  1   1   0
## 6263   6263    0 480 24   1    1  1   1   0
## 6264   6264    0 480 24   1    1  1   1   0
## 6265   6265    0 480 24   1    1  1   1   0
## 6266   6266    0 480 24   1    1  1   1   0
## 6267   6267    0 480 24   1    1  1   1   0
## 6268   6268    0 480 24   1    1  1   1   0
## 6269   6269    0 480 24   1    1  1   1   0
## 6270   6270    0 480 24   1    1  1   1   0
## 6271   6271    0 480 24   1    1  1   1   0
## 6272   6272    0 480 24   1    1  1   1   0
## 6273   6273    0 480 24   1    1  1   1   0
## 6274   6274    0 480 24   1    1  1   1   0
## 6275   6275    0 480 24   1    1  1   1   0
## 6276   6276    0 480 24   1    1  1   1   0
## 6277   6277    0 480 24   1    1  1   1   0
## 6278   6278    0 480 24   1    1  1   1   0
## 6279   6279    0 480 24   1    1  1   1   0
## 6280   6280    0 480 24   1    1  1   1   0
## 6281   6281    0 480 24   1    1  1   1   0
## 6282   6282    0 480 24   1    1  1   1   0
## 6283   6283    0 480 24   1    1  1   1   0
## 6284   6284    0 480 24   1    1  1   1   0
## 6285   6285    0 480 24   1    1  1   1   0
## 6286   6286    0 480 24   1    1  1   1   0
## 6287   6287    0 480 24   1    1  1   1   0
## 6288   6288    0 480 24   1    1  1   1   0
## 6289   6289    0 480 24   1    1  1   1   0
## 6290   6290    0 480 24   1    1  1   1   0
## 6291   6291    0 480 24   1    1  1   1   0
## 6292   6292    0 480 24   1    1  1   1   0
## 6293   6293    0 480 24   1    1  1   1   0
## 6294   6294    0 480 24   1    1  1   1   0
## 6295   6295    0 480 24   1    1  1   1   0
## 6296   6296    0 480 24   1    1  1   1   0
## 6297   6297    0 480 24   1    1  1   1   0
## 6298   6298    0 480 24   1    1  1   1   0
## 6299   6299    0 480 24   1    1  1   1   0
## 6300   6300    0 480 24   1    1  1   1   0
## 6301   6301    0 480 24   1    1  1   1   0
## 6302   6302    0 480 24   1    1  1   1   0
## 6303   6303    0 480 24   1    1  1   1   0
## 6304   6304    0 480 24   1    1  1   1   0
## 6305   6305    0 480 24   1    1  1   1   0
## 6306   6306    0 480 24   1    1  1   1   0
## 6307   6307    0 480 24   1    1  1   1   0
## 6308   6308    0 480 24   1    1  1   1   0
## 6309   6309    0 480 24   1    1  1   1   0
## 6310   6310    0 480 24   1    1  1   1   0
## 6311   6311    0 480 24   1    1  1   1   0
## 6312   6312    0 480 24   1    1  1   1   0
## 6313   6313    0 480 24   1    1  1   1   0
## 6314   6314    0 480 24   1    1  1   1   0
## 6315   6315    0 480 24   1    1  1   1   0
## 6316   6316    0 480 24   1    1  1   1   0
## 6317   6317    0 480 24   1    1  1   1   0
## 6318   6318    0 480 24   1    1  1   1   0
## 6319   6319    0 480 24   1    1  1   1   0
## 6320   6320    0 480 24   1    1  1   1   0
## 6321   6321    0 480 24   1    1  1   1   0
## 6322   6322    0 480 24   1    1  1   1   0
## 6323   6323    0 480 24   1    1  1   1   0
## 6324   6324    0 480 24   1    1  1   1   0
## 6325   6325    0 480 24   1    1  1   1   0
## 6326   6326    0 480 24   1    1  1   1   0
## 6327   6327    0 480 24   1    1  1   1   0
## 6328   6328    0 480 24   1    1  1   1   0
## 6329   6329    0 480 24   1    1  1   1   0
## 6330   6330    0 480 24   1    1  1   1   0
## 6331   6331    0 480 24   1    1  1   1   0
## 6332   6332    0 480 24   1    1  1   1   0
## 6333   6333    0 480 24   1    1  1   1   0
## 6334   6334    0 480 24   1    1  1   1   0
## 6335   6335    0 480 24   1    1  1   1   0
## 6336   6336    0 480 24   1    1  1   1   0
## 6337   6337    0 480 24   1    1  1   1   0
## 6338   6338    0 480 24   1    1  1   1   0
## 6339   6339    0 480 24   1    1  1   1   0
## 6340   6340    0 480 24   1    1  1   1   0
## 6341   6341    0 480 24   1    1  1   1   0
## 6342   6342    0 480 24   1    1  1   1   0
## 6343   6343    0 480 24   1    1  1   1   0
## 6344   6344    0 480 24   1    1  1   1   0
## 6345   6345    0 480 24   1    1  1   1   0
## 6346   6346    0 480 24   1    1  1   1   0
## 6347   6347    0 480 24   1    1  1   1   0
## 6348   6348    0 480 24   1    1  1   1   0
## 6349   6349    0 480 24   1    1  1   1   0
## 6350   6350    0 480 24   1    1  1   1   0
## 6351   6351    0 480 24   1    1  1   1   0
## 6352   6352    0 480 24   1    1  1   1   0
## 6353   6353    0 480 24   1    1  1   1   0
## 6354   6354    0 480 24   1    1  1   1   0
## 6355   6355    0 480 24   1    1  1   1   0
## 6356   6356    0 480 24   1    1  1   1   0
## 6357   6357    0 480 24   1    1  1   1   0
## 6358   6358    0 480 24   1    1  1   1   0
## 6359   6359    0 480 24   1    1  1   1   0
## 6360   6360    0 480 24   1    1  1   1   0
## 6361   6361    0 480 24   1    1  1   1   0
## 6362   6362    0 480 24   1    1  1   1   0
## 6363   6363    0 480 24   1    1  1   1   0
## 6364   6364    0 480 24   1    1  1   1   0
## 6365   6365    0 480 24   1    1  1   1   0
## 6366   6366    0 480 24   1    1  1   1   0
## 6367   6367    0 480 24   1    1  1   1   0
## 6368   6368    0 480 24   1    1  1   1   0
## 6369   6369    0 480 24   1    1  1   1   0
## 6370   6370    0 480 24   1    1  1   1   0
## 6371   6371    0 480 24   1    1  1   1   0
## 6372   6372    0 480 24   1    1  1   1   0
## 6373   6373    0 480 24   1    1  1   1   0
## 6374   6374    0 480 24   1    1  1   1   0
## 6375   6375    0 480 24   1    1  1   1   0
## 6376   6376    0 480 24   1    1  1   1   0
## 6377   6377    0 480 24   1    1  1   1   0
## 6378   6378    0 480 24   1    1  1   1   0
## 6379   6379    0 480 24   1    1  1   1   0
## 6380   6380    0 480 24   1    1  1   1   0
## 6381   6381    0 480 24   1    1  1   1   0
## 6382   6382    0 480 24   1    1  1   1   0
## 6383   6383    0 480 24   1    1  1   1   0
## 6384   6384    0 480 24   1    1  1   1   0
## 6385   6385    0 480 24   1    1  1   1   0
## 6386   6386    0 480 24   1    1  1   1   0
## 6387   6387    0 480 24   1    1  1   1   0
## 6388   6388    0 480 24   1    1  1   1   0
## 6389   6389    0 480 24   1    1  1   1   0
## 6390   6390    0 480 24   1    1  1   1   0
## 6391   6391    0 480 24   1    1  1   1   0
## 6392   6392    0 480 24   1    1  1   1   0
## 6393   6393    0 480 24   1    1  1   1   0
## 6394   6394    0 480 24   1    1  1   1   0
## 6395   6395    0 480 24   1    1  1   1   0
## 6396   6396    0 480 24   1    1  1   1   0
## 6397   6397    0 480 24   1    1  1   1   0
## 6398   6398    0 480 24   1    1  1   1   0
## 6399   6399    0 480 24   1    1  1   1   0
## 6400   6400    0 480 24   1    1  1   1   0
## 6401   6401    0 480 24   1    1  1   1   0
## 6402   6402    0 480 24   1    1  1   1   0
## 6403   6403    0 480 24   1    1  1   1   0
## 6404   6404    0 480 24   1    1  1   1   0
## 6405   6405    0 480 24   1    1  1   1   0
## 6406   6406    0 480 24   1    1  1   1   0
## 6407   6407    0 480 24   1    1  1   1   0
## 6408   6408    0 480 24   1    1  1   1   0
## 6409   6409    0 480 24   1    1  1   1   0
## 6410   6410    0 480 24   1    1  1   1   0
## 6411   6411    0 480 24   1    1  1   1   0
## 6412   6412    0 480 24   1    1  1   1   0
## 6413   6413    0 480 24   1    1  1   1   0
## 6414   6414    0 480 24   1    1  1   1   0
## 6415   6415    0 480 24   1    1  1   1   0
## 6416   6416    0 480 24   1    1  1   1   0
## 6417   6417    0 480 24   1    1  1   1   0
## 6418   6418    0 480 24   1    1  1   1   0
## 6419   6419    0 480 24   1    1  1   1   0
## 6420   6420    0 480 24   1    1  1   1   0
## 6421   6421    0 480 24   1    1  1   1   0
## 6422   6422    0 480 24   1    1  1   1   0
## 6423   6423    0 480 24   1    1  1   1   0
## 6424   6424    0 480 24   1    1  1   1   0
## 6425   6425    0 480 24   1    1  1   1   0
## 6426   6426    0 480 24   1    1  1   1   0
## 6427   6427    0 480 24   1    1  1   1   0
## 6428   6428    0 480 24   1    1  1   1   0
## 6429   6429    0 480 24   1    1  1   1   0
## 6430   6430    0 480 24   1    1  1   1   0
## 6431   6431    0 480 24   1    1  1   1   0
## 6432   6432    0 480 24   1    1  1   1   0
## 6433   6433    0 480 24   1    1  1   1   0
## 6434   6434    0 480 24   1    1  1   1   0
## 6435   6435    0 480 24   1    1  1   1   0
## 6436   6436    0 480 24   1    1  1   1   0
## 6437   6437    0 480 24   1    1  1   1   0
## 6438   6438    0 480 24   1    1  1   1   0
## 6439   6439    0 480 24   1    1  1   1   0
## 6440   6440    0 480 24   1    1  1   1   0
## 6441   6441    0 480 24   1    1  1   1   0
## 6442   6442    0 480 24   1    1  1   1   0
## 6443   6443    0 480 24   1    1  1   1   0
## 6444   6444    0 480 24   1    1  1   1   0
## 6445   6445    0 480 24   1    1  1   1   0
## 6446   6446    0 480 24   1    1  1   1   0
## 6447   6447    0 480 24   1    1  1   1   0
## 6448   6448    0 480 24   1    1  1   1   0
## 6449   6449    0 480 24   1    1  1   1   0
## 6450   6450    0 480 24   1    1  1   1   0
## 6451   6451    0 480 24   1    1  1   1   0
## 6452   6452    0 480 24   1    1  1   1   0
## 6453   6453    0 480 24   1    1  1   1   0
## 6454   6454    0 480 24   1    1  1   1   0
## 6455   6455    0 480 24   1    1  1   1   0
## 6456   6456    0 480 24   1    1  1   1   0
## 6457   6457    0 480 24   1    1  1   1   0
## 6458   6458    0 480 24   1    1  1   1   0
## 6459   6459    0 480 24   1    1  1   1   0
## 6460   6460    0 480 24   1    1  1   1   0
## 6461   6461    0 480 24   1    1  1   1   0
## 6462   6462    0 480 24   1    1  1   1   0
## 6463   6463    0 480 24   1    1  1   1   0
## 6464   6464    0 480 24   1    1  1   1   0
## 6465   6465    0 480 24   1    1  1   1   0
## 6466   6466    0 480 24   1    1  1   1   0
## 6467   6467    0 480 24   1    1  1   1   0
## 6468   6468    0 480 24   1    1  1   1   0
## 6469   6469    0 480 24   1    1  1   1   0
## 6470   6470    0 480 24   1    1  1   1   0
## 6471   6471    0 480 24   1    1  1   1   0
## 6472   6472    0 480 24   1    1  1   1   0
## 6473   6473    0 480 24   1    1  1   1   0
## 6474   6474    0 480 24   1    1  1   1   0
## 6475   6475    0 480 24   1    1  1   1   0
## 6476   6476    0 480 24   1    1  1   1   0
## 6477   6477    0 480 24   1    1  1   1   0
## 6478   6478    0 480 24   1    1  1   1   0
## 6479   6479    0 480 24   1    1  1   1   0
## 6480   6480    0 480 24   1    1  1   1   0
## 6481   6481    0 480 24   1    1  1   1   0
## 6482   6482    0 480 24   1    1  1   1   0
## 6483   6483    0 480 24   1    1  1   1   0
## 6484   6484    0 480 24   1    1  1   1   0
## 6485   6485    0 480 24   1    1  1   1   0
## 6486   6486    0 480 24   1    1  1   1   0
## 6487   6487    0 480 24   1    1  1   1   0
## 6488   6488    0 480 24   1    1  1   1   0
## 6489   6489    0 480 24   1    1  1   1   0
## 6490   6490    0 480 24   1    1  1   1   0
## 6491   6491    0 480 24   1    1  1   1   0
## 6492   6492    0 480 24   1    1  1   1   0
## 6493   6493    0 480 24   1    1  1   1   0
## 6494   6494    0 480 24   1    1  1   1   0
## 6495   6495    0 480 24   1    1  1   1   0
## 6496   6496    0 480 24   1    1  1   1   0
## 6497   6497    0 480 24   1    1  1   1   0
## 6498   6498    0 480 24   1    1  1   1   0
## 6499   6499    0 480 24   1    1  1   1   0
## 6500   6500    0 480 24   1    1  1   1   0
## 6501   6501    0 480 24   1    1  1   1   0
## 6502   6502    0 480 24   1    1  1   1   0
## 6503   6503    0 480 24   1    1  1   1   0
## 6504   6504    0 480 24   1    1  1   1   0
## 6505   6505    0 480 24   1    1  1   1   0
## 6506   6506    0 480 24   1    1  1   1   0
## 6507   6507    0 480 24   1    1  1   1   0
## 6508   6508    0 480 24   1    1  1   1   0
## 6509   6509    0 480 24   1    1  1   1   0
## 6510   6510    0 480 24   1    1  1   1   0
## 6511   6511    0 480 24   1    1  1   1   0
## 6512   6512    0 480 24   1    1  1   1   0
## 6513   6513    0 480 24   1    1  1   1   0
## 6514   6514    0 480 24   1    1  1   1   0
## 6515   6515    0 480 24   1    1  1   1   0
## 6516   6516    0 480 24   1    1  1   1   0
## 6517   6517    0 480 24   1    1  1   1   0
## 6518   6518    0 480 24   1    1  1   1   0
## 6519   6519    0 480 24   1    1  1   1   0
## 6520   6520    0 480 24   1    1  1   1   0
## 6521   6521    0 480 24   1    1  1   1   0
## 6522   6522    0 480 24   1    1  1   1   0
## 6523   6523    0 480 24   1    1  1   1   0
## 6524   6524    0 480 24   1    1  1   1   0
## 6525   6525    0 480 24   1    1  1   1   0
## 6526   6526    0 480 24   1    1  1   1   0
## 6527   6527    0 480 24   1    1  1   1   0
## 6528   6528    0 480 24   1    1  1   1   0
## 6529   6529    0 480 24   1    1  1   1   0
## 6530   6530    0 480 24   1    1  1   1   0
## 6531   6531    0 480 24   1    1  1   1   0
## 6532   6532    0 480 24   1    1  1   1   0
## 6533   6533    0 480 24   1    1  1   1   0
## 6534   6534    0 480 24   1    1  1   1   0
## 6535   6535    0 480 24   1    1  1   1   0
## 6536   6536    0 480 24   1    1  1   1   0
## 6537   6537    0 480 24   1    1  1   1   0
## 6538   6538    0 480 24   1    1  1   1   0
## 6539   6539    0 480 24   1    1  1   1   0
## 6540   6540    0 480 24   1    1  1   1   0
## 6541   6541    0 480 24   1    1  1   1   0
## 6542   6542    0 480 24   1    1  1   1   0
## 6543   6543    0 480 24   1    1  1   1   0
## 6544   6544    0 480 24   1    1  1   1   0
## 6545   6545    0 480 24   1    1  1   1   0
## 6546   6546    0 480 24   1    1  1   1   0
## 6547   6547    0 480 24   1    1  1   1   0
## 6548   6548    0 480 24   1    1  1   1   0
## 6549   6549    0 480 24   1    1  1   1   0
## 6550   6550    0 480 24   1    1  1   1   0
## 6551   6551    0 480 24   1    1  1   1   0
## 6552   6552    0 480 24   1    1  1   1   0
## 6553   6553    0 480 24   1    1  1   1   0
## 6554   6554    0 480 24   1    1  1   1   0
## 6555   6555    0 480 24   1    1  1   1   0
## 6556   6556    0 480 24   1    1  1   1   0
## 6557   6557    0 480 24   1    1  1   1   0
## 6558   6558    0 480 24   1    1  1   1   0
## 6559   6559    0 480 24   1    1  1   1   0
## 6560   6560    0 480 24   1    1  1   1   0
## 6561   6561    0 480 24   1    1  1   1   0
## 6562   6562    0 480 24   1    1  1   1   0
## 6563   6563    0 480 24   1    1  1   1   0
## 6564   6564    0 480 24   1    1  1   1   0
## 6565   6565    0 480 24   1    1  1   1   0
## 6566   6566    0 480 24   1    1  1   1   0
## 6567   6567    0 480 24   1    1  1   1   0
## 6568   6568    0 480 24   1    1  1   1   0
## 6569   6569    0 480 24   1    1  1   1   0
## 6570   6570    0 480 24   1    1  1   1   0
## 6571   6571    0 480 24   1    1  1   1   0
## 6572   6572    0 480 24   1    1  1   1   0
## 6573   6573    0 480 24   1    1  1   1   0
## 6574   6574    0 480 24   1    1  1   1   0
## 6575   6575    0 480 24   1    1  1   1   0
## 6576   6576    0 480 24   1    1  1   1   0
## 6577   6577    0 480 24   1    1  1   1   0
## 6578   6578    0 480 24   1    1  1   1   0
## 6579   6579    0 480 24   1    1  1   1   0
## 6580   6580    0 480 24   1    1  1   1   0
## 6581   6581    0 480 24   1    1  1   1   0
## 6582   6582    0 480 24   1    1  1   1   0
## 6583   6583    0 480 24   1    1  1   1   0
## 6584   6584    0 480 24   1    1  1   1   0
## 6585   6585    0 480 24   1    1  1   1   0
## 6586   6586    0 480 24   1    1  1   1   0
## 6587   6587    0 480 24   1    1  1   1   0
## 6588   6588    0 480 24   1    1  1   1   0
## 6589   6589    0 480 24   1    1  1   1   0
## 6590   6590    0 480 24   1    1  1   1   0
## 6591   6591    0 480 24   1    1  1   1   0
## 6592   6592    0 480 24   1    1  1   1   0
## 6593   6593    0 480 24   1    1  1   1   0
## 6594   6594    0 480 24   1    1  1   1   0
## 6595   6595    0 480 24   1    1  1   1   0
## 6596   6596    0 480 24   1    1  1   1   0
## 6597   6597    0 480 24   1    1  1   1   0
## 6598   6598    0 480 24   1    1  1   1   0
## 6599   6599    0 480 24   1    1  1   1   0
## 6600   6600    0 480 24   1    1  1   1   0
## 6601   6601    0 480 24   1    1  1   1   0
## 6602   6602    0 480 24   1    1  1   1   0
## 6603   6603    0 480 24   1    1  1   1   0
## 6604   6604    0 480 24   1    1  1   1   0
## 6605   6605    0 480 24   1    1  1   1   0
## 6606   6606    0 480 24   1    1  1   1   0
## 6607   6607    0 480 24   1    1  1   1   0
## 6608   6608    0 480 24   1    1  1   1   0
## 6609   6609    0 480 24   1    1  1   1   0
## 6610   6610    0 480 24   1    1  1   1   0
## 6611   6611    0 480 24   1    1  1   1   0
## 6612   6612    0 480 24   1    1  1   1   0
## 6613   6613    0 480 24   1    1  1   1   0
## 6614   6614    0 480 24   1    1  1   1   0
## 6615   6615    0 480 24   1    1  1   1   0
## 6616   6616    0 480 24   1    1  1   1   0
## 6617   6617    0 480 24   1    1  1   1   0
## 6618   6618    0 480 24   1    1  1   1   0
## 6619   6619    0 480 24   1    1  1   1   0
## 6620   6620    0 480 24   1    1  1   1   0
## 6621   6621    0 480 24   1    1  1   1   0
## 6622   6622    0 480 24   1    1  1   1   0
## 6623   6623    0 480 24   1    1  1   1   0
## 6624   6624    0 480 24   1    1  1   1   0
## 6625   6625    0 480 24   1    1  1   1   0
## 6626   6626    0 480 24   1    1  1   1   0
## 6627   6627    0 480 24   1    1  1   1   0
## 6628   6628    0 480 24   1    1  1   1   0
## 6629   6629    0 480 24   1    1  1   1   0
## 6630   6630    0 480 24   1    1  1   1   0
## 6631   6631    0 480 24   1    1  1   1   0
## 6632   6632    0 480 24   1    1  1   1   0
## 6633   6633    0 480 24   1    1  1   1   0
## 6634   6634    0 480 24   1    1  1   1   0
## 6635   6635    0 480 24   1    1  1   1   0
## 6636   6636    0 480 24   1    1  1   1   0
## 6637   6637    0 480 24   1    1  1   1   0
## 6638   6638    0 480 24   1    1  1   1   0
## 6639   6639    0 480 24   1    1  1   1   0
## 6640   6640    0 480 24   1    1  1   1   0
## 6641   6641    0 480 24   1    1  1   1   0
## 6642   6642    0 480 24   1    1  1   1   0
## 6643   6643    0 480 24   1    1  1   1   0
## 6644   6644    0 480 24   1    1  1   1   0
## 6645   6645    0 480 24   1    1  1   1   0
## 6646   6646    0 480 24   1    1  1   1   0
## 6647   6647    0 480 24   1    1  1   1   0
## 6648   6648    0 480 24   1    1  1   1   0
## 6649   6649    0 480 24   1    1  1   1   0
## 6650   6650    0 480 24   1    1  1   1   0
## 6651   6651    0 480 24   1    1  1   1   0
## 6652   6652    0 480 24   1    1  1   1   0
## 6653   6653    0 480 24   1    1  1   1   0
## 6654   6654    0 480 24   1    1  1   1   0
## 6655   6655    0 480 24   1    1  1   1   0
## 6656   6656    0 480 24   1    1  1   1   0
## 6657   6657    0 480 24   1    1  1   1   0
## 6658   6658    0 480 24   1    1  1   1   0
## 6659   6659    0 480 24   1    1  1   1   0
## 6660   6660    0 480 24   1    1  1   1   0
## 6661   6661    0 480 24   1    1  1   1   0
## 6662   6662    0 480 24   1    1  1   1   0
## 6663   6663    0 480 24   1    1  1   1   0
## 6664   6664    0 480 24   1    1  1   1   0
## 6665   6665    0 480 24   1    1  1   1   0
## 6666   6666    0 480 24   1    1  1   1   0
## 6667   6667    0 480 24   1    1  1   1   0
## 6668   6668    0 480 24   1    1  1   1   0
## 6669   6669    0 480 24   1    1  1   1   0
## 6670   6670    0 480 24   1    1  1   1   0
## 6671   6671    0 480 24   1    1  1   1   0
## 6672   6672    0 480 24   1    1  1   1   0
## 6673   6673    0 480 24   1    1  1   1   0
## 6674   6674    0 480 24   1    1  1   1   0
## 6675   6675    0 480 24   1    1  1   1   0
## 6676   6676    0 480 24   1    1  1   1   0
## 6677   6677    0 480 24   1    1  1   1   0
## 6678   6678    0 480 24   1    1  1   1   0
## 6679   6679    0 480 24   1    1  1   1   0
## 6680   6680    0 480 24   1    1  1   1   0
## 6681   6681    0 480 24   1    1  1   1   0
## 6682   6682    0 480 24   1    1  1   1   0
## 6683   6683    0 480 24   1    1  1   1   0
## 6684   6684    0 480 24   1    1  1   1   0
## 6685   6685    0 480 24   1    1  1   1   0
## 6686   6686    0 480 24   1    1  1   1   0
## 6687   6687    0 480 24   1    1  1   1   0
## 6688   6688    0 480 24   1    1  1   1   0
## 6689   6689    0 480 24   1    1  1   1   0
## 6690   6690    0 480 24   1    1  1   1   0
## 6691   6691    0 480 24   1    1  1   1   0
## 6692   6692    0 480 24   1    1  1   1   0
## 6693   6693    0 480 24   1    1  1   1   0
## 6694   6694    0 480 24   1    1  1   1   0
## 6695   6695    0 480 24   1    1  1   1   0
## 6696   6696    0 480 24   1    1  1   1   0
## 6697   6697    0 480 24   1    1  1   1   0
## 6698   6698    0 480 24   1    1  1   1   0
## 6699   6699    0 480 24   1    1  1   1   0
## 6700   6700    0 480 24   1    1  1   1   0
## 6701   6701    0 480 24   1    1  1   1   0
## 6702   6702    0 480 24   1    1  1   1   0
## 6703   6703    0 480 24   1    1  1   1   0
## 6704   6704    0 480 24   1    1  1   1   0
## 6705   6705    0 480 24   1    1  1   1   0
## 6706   6706    0 480 24   1    1  1   1   0
## 6707   6707    0 480 24   1    1  1   1   0
## 6708   6708    0 480 24   1    1  1   1   0
## 6709   6709    0 480 24   1    1  1   1   0
## 6710   6710    0 480 24   1    1  1   1   0
## 6711   6711    0 480 24   1    1  1   1   0
## 6712   6712    0 480 24   1    1  1   1   0
## 6713   6713    0 480 24   1    1  1   1   0
## 6714   6714    0 480 24   1    1  1   1   0
## 6715   6715    0 480 24   1    1  1   1   0
## 6716   6716    0 480 24   1    1  1   1   0
## 6717   6717    0 480 24   1    1  1   1   0
## 6718   6718    0 480 24   1    1  1   1   0
## 6719   6719    0 480 24   1    1  1   1   0
## 6720   6720    0 480 24   1    1  1   1   0
## 6721   6721    0 480 24   1    1  1   1   0
## 6722   6722    0 480 24   1    1  1   1   0
## 6723   6723    0 480 24   1    1  1   1   0
## 6724   6724    0 480 24   1    1  1   1   0
## 6725   6725    0 480 24   1    1  1   1   0
## 6726   6726    0 480 24   1    1  1   1   0
## 6727   6727    0 480 24   1    1  1   1   0
## 6728   6728    0 480 24   1    1  1   1   0
## 6729   6729    0 480 24   1    1  1   1   0
## 6730   6730    0 480 24   1    1  1   1   0
## 6731   6731    0 480 24   1    1  1   1   0
## 6732   6732    0 480 24   1    1  1   1   0
## 6733   6733    0 480 24   1    1  1   1   0
## 6734   6734    0 480 24   1    1  1   1   0
## 6735   6735    0 480 24   1    1  1   1   0
## 6736   6736    0 480 24   1    1  1   1   0
## 6737   6737    0 480 24   1    1  1   1   0
## 6738   6738    0 480 24   1    1  1   1   0
## 6739   6739    0 480 24   1    1  1   1   0
## 6740   6740    0 480 24   1    1  1   1   0
## 6741   6741    0 480 24   1    1  1   1   0
## 6742   6742    0 480 24   1    1  1   1   0
## 6743   6743    0 480 24   1    1  1   1   0
## 6744   6744    0 480 24   1    1  1   1   0
## 6745   6745    0 480 24   1    1  1   1   0
## 6746   6746    0 480 24   1    1  1   1   0
## 6747   6747    0 480 24   1    1  1   1   0
## 6748   6748    0 480 24   1    1  1   1   0
## 6749   6749    0 480 24   1    1  1   1   0
## 6750   6750    0 480 24   1    1  1   1   0
## 6751   6751    0 480 24   1    1  1   1   0
## 6752   6752    0 480 24   1    1  1   1   0
## 6753   6753    0 480 24   1    1  1   1   0
## 6754   6754    0 480 24   1    1  1   1   0
## 6755   6755    0 480 24   1    1  1   1   0
## 6756   6756    0 480 24   1    1  1   1   0
## 6757   6757    0 480 24   1    1  1   1   0
## 6758   6758    0 480 24   1    1  1   1   0
## 6759   6759    0 480 24   1    1  1   1   0
## 6760   6760    0 480 24   1    1  1   1   0
## 6761   6761    0 480 24   1    1  1   1   0
## 6762   6762    0 480 24   1    1  1   1   0
## 6763   6763    0 480 24   1    1  1   1   0
## 6764   6764    0 480 24   1    1  1   1   0
## 6765   6765    0 480 24   1    1  1   1   0
## 6766   6766    0 480 24   1    1  1   1   0
## 6767   6767    0 480 24   1    1  1   1   0
## 6768   6768    0 480 24   1    1  1   1   0
## 6769   6769    0 480 24   1    1  1   1   0
## 6770   6770    0 480 24   1    1  1   1   0
## 6771   6771    0 480 24   1    1  1   1   0
## 6772   6772    0 480 24   1    1  1   1   0
## 6773   6773    0 480 24   1    1  1   1   0
## 6774   6774    0 480 24   1    1  1   1   0
## 6775   6775    0 480 24   1    1  1   1   0
## 6776   6776    0 480 24   1    1  1   1   0
## 6777   6777    0 480 24   1    1  1   1   0
## 6778   6778    0 480 24   1    1  1   1   0
## 6779   6779    0 480 24   1    1  1   1   0
## 6780   6780    0 480 24   1    1  1   1   0
## 6781   6781    0 480 24   1    1  1   1   0
## 6782   6782    0 480 24   1    1  1   1   0
## 6783   6783    0 480 24   1    1  1   1   0
## 6784   6784    0 480 24   1    1  1   1   0
## 6785   6785    0 480 24   1    1  1   1   0
## 6786   6786    0 480 24   1    1  1   1   0
## 6787   6787    0 480 24   1    1  1   1   0
## 6788   6788    0 480 24   1    1  1   1   0
## 6789   6789    0 480 24   1    1  1   1   0
## 6790   6790    0 480 24   1    1  1   1   0
## 6791   6791    0 480 24   1    1  1   1   0
## 6792   6792    0 480 24   1    1  1   1   0
## 6793   6793    0 480 24   1    1  1   1   0
## 6794   6794    0 480 24   1    1  1   1   0
## 6795   6795    0 480 24   1    1  1   1   0
## 6796   6796    0 480 24   1    1  1   1   0
## 6797   6797    0 480 24   1    1  1   1   0
## 6798   6798    0 480 24   1    1  1   1   0
## 6799   6799    0 480 24   1    1  1   1   0
## 6800   6800    0 480 24   1    1  1   1   0
## 6801   6801    0 480 24   1    1  1   1   0
## 6802   6802    0 480 24   1    1  1   1   0
## 6803   6803    0 480 24   1    1  1   1   0
## 6804   6804    0 480 24   1    1  1   1   0
## 6805   6805    0 480 24   1    1  1   1   0
## 6806   6806    0 480 24   1    1  1   1   0
## 6807   6807    0 480 24   1    1  1   1   0
## 6808   6808    0 480 24   1    1  1   1   0
## 6809   6809    0 480 24   1    1  1   1   0
## 6810   6810    0 480 24   1    1  1   1   0
## 6811   6811    0 480 24   1    1  1   1   0
## 6812   6812    0 480 24   1    1  1   1   0
## 6813   6813    0 480 24   1    1  1   1   0
## 6814   6814    0 480 24   1    1  1   1   0
## 6815   6815    0 480 24   1    1  1   1   0
## 6816   6816    0 480 24   1    1  1   1   0
## 6817   6817    0 480 24   1    1  1   1   0
## 6818   6818    0 480 24   1    1  1   1   0
## 6819   6819    0 480 24   1    1  1   1   0
## 6820   6820    0 480 24   1    1  1   1   0
## 6821   6821    0 480 24   1    1  1   1   0
## 6822   6822    0 480 24   1    1  1   1   0
## 6823   6823    0 480 24   1    1  1   1   0
## 6824   6824    0 480 24   1    1  1   1   0
## 6825   6825    0 480 24   1    1  1   1   0
## 6826   6826    0 480 24   1    1  1   1   0
## 6827   6827    0 480 24   1    1  1   1   0
## 6828   6828    0 480 24   1    1  1   1   0
## 6829   6829    0 480 24   1    1  1   1   0
## 6830   6830    0 480 24   1    1  1   1   0
## 6831   6831    0 480 24   1    1  1   1   0
## 6832   6832    0 480 24   1    1  1   1   0
## 6833   6833    0 480 24   1    1  1   1   0
## 6834   6834    0 480 24   1    1  1   1   0
## 6835   6835    0 480 24   1    1  1   1   0
## 6836   6836    0 480 24   1    1  1   1   0
## 6837   6837    0 480 24   1    1  1   1   0
## 6838   6838    0 480 24   1    1  1   1   0
## 6839   6839    0 480 24   1    1  1   1   0
## 6840   6840    0 480 24   1    1  1   1   0
## 6841   6841    0 480 24   1    1  1   1   0
## 6842   6842    0 480 24   1    1  1   1   0
## 6843   6843    0 480 24   1    1  1   1   0
## 6844   6844    0 480 24   1    1  1   1   0
## 6845   6845    0 480 24   1    1  1   1   0
## 6846   6846    0 480 24   1    1  1   1   0
## 6847   6847    0 480 24   1    1  1   1   0
## 6848   6848    0 480 24   1    1  1   1   0
## 6849   6849    0 480 24   1    1  1   1   0
## 6850   6850    0 480 24   1    1  1   1   0
## 6851   6851    0 480 24   1    1  1   1   0
## 6852   6852    0 480 24   1    1  1   1   0
## 6853   6853    0 480 24   1    1  1   1   0
## 6854   6854    0 480 24   1    1  1   1   0
## 6855   6855    0 480 24   1    1  1   1   0
## 6856   6856    0 480 24   1    1  1   1   0
## 6857   6857    0 480 24   1    1  1   1   0
## 6858   6858    0 480 24   1    1  1   1   0
## 6859   6859    0 480 24   1    1  1   1   0
## 6860   6860    0 480 24   1    1  1   1   0
## 6861   6861    0 480 24   1    1  1   1   0
## 6862   6862    0 480 24   1    1  1   1   0
## 6863   6863    0 480 24   1    1  1   1   0
## 6864   6864    0 480 24   1    1  1   1   0
## 6865   6865    0 480 24   1    1  1   1   0
## 6866   6866    0 480 24   1    1  1   1   0
## 6867   6867    0 480 24   1    1  1   1   0
## 6868   6868    0 480 24   1    1  1   1   0
## 6869   6869    0 480 24   1    1  1   1   0
## 6870   6870    0 480 24   1    1  1   1   0
## 6871   6871    0 480 24   1    1  1   1   0
## 6872   6872    0 480 24   1    1  1   1   0
## 6873   6873    0 480 24   1    1  1   1   0
## 6874   6874    0 480 24   1    1  1   1   0
## 6875   6875    0 480 24   1    1  1   1   0
## 6876   6876    0 480 24   1    1  1   1   0
## 6877   6877    0 480 24   1    1  1   1   0
## 6878   6878    0 480 24   1    1  1   1   0
## 6879   6879    0 480 24   1    1  1   1   0
## 6880   6880    0 480 24   1    1  1   1   0
## 6881   6881    0 480 24   1    1  1   1   0
## 6882   6882    0 480 24   1    1  1   1   0
## 6883   6883    0 480 24   1    1  1   1   0
## 6884   6884    0 480 24   1    1  1   1   0
## 6885   6885    0 480 24   1    1  1   1   0
## 6886   6886    0 480 24   1    1  1   1   0
## 6887   6887    0 480 24   1    1  1   1   0
## 6888   6888    0 480 24   1    1  1   1   0
## 6889   6889    0 480 24   1    1  1   1   0
## 6890   6890    0 480 24   1    1  1   1   0
## 6891   6891    0 480 24   1    1  1   1   0
## 6892   6892    0 480 24   1    1  1   1   0
## 6893   6893    0 480 24   1    1  1   1   0
## 6894   6894    0 480 24   1    1  1   1   0
## 6895   6895    0 480 24   1    1  1   1   0
## 6896   6896    0 480 24   1    1  1   1   0
## 6897   6897    0 480 24   1    1  1   1   0
## 6898   6898    0 480 24   1    1  1   1   0
## 6899   6899    0 480 24   1    1  1   1   0
## 6900   6900    0 480 24   1    1  1   1   0
## 6901   6901    0 480 24   1    1  1   1   0
## 6902   6902    0 480 24   1    1  1   1   0
## 6903   6903    0 480 24   1    1  1   1   0
## 6904   6904    0 480 24   1    1  1   1   0
## 6905   6905    0 480 24   1    1  1   1   0
## 6906   6906    0 480 24   1    1  1   1   0
## 6907   6907    0 480 24   1    1  1   1   0
## 6908   6908    0 480 24   1    1  1   1   0
## 6909   6909    0 480 24   1    1  1   1   0
## 6910   6910    0 480 24   1    1  1   1   0
## 6911   6911    0 480 24   1    1  1   1   0
## 6912   6912    0 480 24   1    1  1   1   0
## 6913   6913    0 480 24   1    1  1   1   0
## 6914   6914    0 480 24   1    1  1   1   0
## 6915   6915    0 480 24   1    1  1   1   0
## 6916   6916    0 480 24   1    1  1   1   0
## 6917   6917    0 480 24   1    1  1   1   0
## 6918   6918    0 480 24   1    1  1   1   0
## 6919   6919    0 480 24   1    1  1   1   0
## 6920   6920    0 480 24   1    1  1   1   0
## 6921   6921    0 480 24   1    1  1   1   0
## 6922   6922    0 480 24   1    1  1   1   0
## 6923   6923    0 480 24   1    1  1   1   0
## 6924   6924    0 480 24   1    1  1   1   0
## 6925   6925    0 480 24   1    1  1   1   0
## 6926   6926    0 480 24   1    1  1   1   0
## 6927   6927    0 480 24   1    1  1   1   0
## 6928   6928    0 480 24   1    1  1   1   0
## 6929   6929    0 480 24   1    1  1   1   0
## 6930   6930    0 480 24   1    1  1   1   0
## 6931   6931    0 480 24   1    1  1   1   0
## 6932   6932    0 480 24   1    1  1   1   0
## 6933   6933    0 480 24   1    1  1   1   0
## 6934   6934    0 480 24   1    1  1   1   0
## 6935   6935    0 480 24   1    1  1   1   0
## 6936   6936    0 480 24   1    1  1   1   0
## 6937   6937    0 480 24   1    1  1   1   0
## 6938   6938    0 480 24   1    1  1   1   0
## 6939   6939    0 480 24   1    1  1   1   0
## 6940   6940    0 480 24   1    1  1   1   0
## 6941   6941    0 480 24   1    1  1   1   0
## 6942   6942    0 480 24   1    1  1   1   0
## 6943   6943    0 480 24   1    1  1   1   0
## 6944   6944    0 480 24   1    1  1   1   0
## 6945   6945    0 480 24   1    1  1   1   0
## 6946   6946    0 480 24   1    1  1   1   0
## 6947   6947    0 480 24   1    1  1   1   0
## 6948   6948    0 480 24   1    1  1   1   0
## 6949   6949    0 480 24   1    1  1   1   0
## 6950   6950    0 480 24   1    1  1   1   0
## 6951   6951    0 480 24   1    1  1   1   0
## 6952   6952    0 480 24   1    1  1   1   0
## 6953   6953    0 480 24   1    1  1   1   0
## 6954   6954    0 480 24   1    1  1   1   0
## 6955   6955    0 480 24   1    1  1   1   0
## 6956   6956    0 480 24   1    1  1   1   0
## 6957   6957    0 480 24   1    1  1   1   0
## 6958   6958    0 480 24   1    1  1   1   0
## 6959   6959    0 480 24   1    1  1   1   0
## 6960   6960    0 480 24   1    1  1   1   0
## 6961   6961    0 480 24   1    1  1   1   0
## 6962   6962    0 480 24   1    1  1   1   0
## 6963   6963    0 480 24   1    1  1   1   0
## 6964   6964    0 480 24   1    1  1   1   0
## 6965   6965    0 480 24   1    1  1   1   0
## 6966   6966    0 480 24   1    1  1   1   0
## 6967   6967    0 480 24   1    1  1   1   0
## 6968   6968    0 480 24   1    1  1   1   0
## 6969   6969    0 480 24   1    1  1   1   0
## 6970   6970    0 480 24   1    1  1   1   0
## 6971   6971    0 480 24   1    1  1   1   0
## 6972   6972    0 480 24   1    1  1   1   0
## 6973   6973    0 480 24   1    1  1   1   0
## 6974   6974    0 480 24   1    1  1   1   0
## 6975   6975    0 480 24   1    1  1   1   0
## 6976   6976    0 480 24   1    1  1   1   0
## 6977   6977    0 480 24   1    1  1   1   0
## 6978   6978    0 480 24   1    1  1   1   0
## 6979   6979    0 480 24   1    1  1   1   0
## 6980   6980    0 480 24   1    1  1   1   0
## 6981   6981    0 480 24   1    1  1   1   0
## 6982   6982    0 480 24   1    1  1   1   0
## 6983   6983    0 480 24   1    1  1   1   0
## 6984   6984    0 480 24   1    1  1   1   0
## 6985   6985    0 480 24   1    1  1   1   0
## 6986   6986    0 480 24   1    1  1   1   0
## 6987   6987    0 480 24   1    1  1   1   0
## 6988   6988    0 480 24   1    1  1   1   0
## 6989   6989    0 480 24   1    1  1   1   0
## 6990   6990    0 480 24   1    1  1   1   0
## 6991   6991    0 480 24   1    1  1   1   0
## 6992   6992    0 480 24   1    1  1   1   0
## 6993   6993    0 480 24   1    1  1   1   0
## 6994   6994    0 480 24   1    1  1   1   0
## 6995   6995    0 480 24   1    1  1   1   0
## 6996   6996    0 480 24   1    1  1   1   0
## 6997   6997    0 480 24   1    1  1   1   0
## 6998   6998    0 480 24   1    1  1   1   0
## 6999   6999    0 480 24   1    1  1   1   0
## 7000   7000    0 480 24   1    1  1   1   0
## 7001   7001    0 480 24   1    1  1   1   0
## 7002   7002    0 480 24   1    1  1   1   0
## 7003   7003    0 480 24   1    1  1   1   0
## 7004   7004    0 480 24   1    1  1   1   0
## 7005   7005    0 480 24   1    1  1   1   0
## 7006   7006    0 480 24   1    1  1   1   0
## 7007   7007    0 480 24   1    1  1   1   0
## 7008   7008    0 480 24   1    1  1   1   0
## 7009   7009    0 480 24   1    1  1   1   0
## 7010   7010    0 480 24   1    1  1   1   0
## 7011   7011    0 480 24   1    1  1   1   0
## 7012   7012    0 480 24   1    1  1   1   0
## 7013   7013    0 480 24   1    1  1   1   0
## 7014   7014    0 480 24   1    1  1   1   0
## 7015   7015    0 480 24   1    1  1   1   0
## 7016   7016    0 480 24   1    1  1   1   0
## 7017   7017    0 480 24   1    1  1   1   0
## 7018   7018    0 480 24   1    1  1   1   0
## 7019   7019    0 480 24   1    1  1   1   0
## 7020   7020    0 480 24   1    1  1   1   0
## 7021   7021    0 480 24   1    1  1   1   0
## 7022   7022    0 480 24   1    1  1   1   0
## 7023   7023    0 480 24   1    1  1   1   0
## 7024   7024    0 480 24   1    1  1   1   0
## 7025   7025    0 480 24   1    1  1   1   0
## 7026   7026    0 480 24   1    1  1   1   0
## 7027   7027    0 480 24   1    1  1   1   0
## 7028   7028    0 480 24   1    1  1   1   0
## 7029   7029    0 480 24   1    1  1   1   0
## 7030   7030    0 480 24   1    1  1   1   0
## 7031   7031    0 480 24   1    1  1   1   0
## 7032   7032    0 480 24   1    1  1   1   0
## 7033   7033    0 480 24   1    1  1   1   0
## 7034   7034    0 480 24   1    1  1   1   0
## 7035   7035    0 480 24   1    1  1   1   0
## 7036   7036    0 480 24   1    1  1   1   0
## 7037   7037    0 480 24   1    1  1   1   0
## 7038   7038    0 480 24   1    1  1   1   0
## 7039   7039    0 480 24   1    1  1   1   0
## 7040   7040    0 480 24   1    1  1   1   0
## 7041   7041    0 480 24   1    1  1   1   0
## 7042   7042    0 480 24   1    1  1   1   0
## 7043   7043    0 480 24   1    1  1   1   0
## 7044   7044    0 480 24   1    1  1   1   0
## 7045   7045    0 480 24   1    1  1   1   0
## 7046   7046    0 480 24   1    1  1   1   0
## 7047   7047    0 480 24   1    1  1   1   0
## 7048   7048    0 480 24   1    1  1   1   0
## 7049   7049    0 480 24   1    1  1   1   0
## 7050   7050    0 480 24   1    1  1   1   0
## 7051   7051    0 480 24   1    1  1   1   0
## 7052   7052    0 480 24   1    1  1   1   0
## 7053   7053    0 480 24   1    1  1   1   0
## 7054   7054    0 480 24   1    1  1   1   0
## 7055   7055    0 480 24   1    1  1   1   0
## 7056   7056    0 480 24   1    1  1   1   0
## 7057   7057    0 480 24   1    1  1   1   0
## 7058   7058    0 480 24   1    1  1   1   0
## 7059   7059    0 480 24   1    1  1   1   0
## 7060   7060    0 480 24   1    1  1   1   0
## 7061   7061    0 480 24   1    1  1   1   0
## 7062   7062    0 480 24   1    1  1   1   0
## 7063   7063    0 480 24   1    1  1   1   0
## 7064   7064    0 480 24   1    1  1   1   0
## 7065   7065    0 480 24   1    1  1   1   0
## 7066   7066    0 480 24   1    1  1   1   0
## 7067   7067    0 480 24   1    1  1   1   0
## 7068   7068    0 480 24   1    1  1   1   0
## 7069   7069    0 480 24   1    1  1   1   0
## 7070   7070    0 480 24   1    1  1   1   0
## 7071   7071    0 480 24   1    1  1   1   0
## 7072   7072    0 480 24   1    1  1   1   0
## 7073   7073    0 480 24   1    1  1   1   0
## 7074   7074    0 480 24   1    1  1   1   0
## 7075   7075    0 480 24   1    1  1   1   0
## 7076   7076    0 480 24   1    1  1   1   0
## 7077   7077    0 480 24   1    1  1   1   0
## 7078   7078    0 480 24   1    1  1   1   0
## 7079   7079    0 480 24   1    1  1   1   0
## 7080   7080    0 480 24   1    1  1   1   0
## 7081   7081    0 480 24   1    1  1   1   0
## 7082   7082    0 480 24   1    1  1   1   0
## 7083   7083    0 480 24   1    1  1   1   0
## 7084   7084    0 480 24   1    1  1   1   0
## 7085   7085    0 480 24   1    1  1   1   0
## 7086   7086    0 480 24   1    1  1   1   0
## 7087   7087    0 480 24   1    1  1   1   0
## 7088   7088    0 480 24   1    1  1   1   0
## 7089   7089    0 480 24   1    1  1   1   0
## 7090   7090    0 480 24   1    1  1   1   0
## 7091   7091    0 480 24   1    1  1   1   0
## 7092   7092    0 480 24   1    1  1   1   0
## 7093   7093    0 480 24   1    1  1   1   0
## 7094   7094    0 480 24   1    1  1   1   0
## 7095   7095    0 480 24   1    1  1   1   0
## 7096   7096    0 480 24   1    1  1   1   0
## 7097   7097    0 480 24   1    1  1   1   0
## 7098   7098    0 480 24   1    1  1   1   0
## 7099   7099    0 480 24   1    1  1   1   0
## 7100   7100    0 480 24   1    1  1   1   0
## 7101   7101    0 480 24   1    1  1   1   0
## 7102   7102    0 480 24   1    1  1   1   0
## 7103   7103    0 480 24   1    1  1   1   0
## 7104   7104    0 480 24   1    1  1   1   0
## 7105   7105    0 480 24   1    1  1   1   0
## 7106   7106    0 480 24   1    1  1   1   0
## 7107   7107    0 480 24   1    1  1   1   0
## 7108   7108    0 480 24   1    1  1   1   0
## 7109   7109    0 480 24   1    1  1   1   0
## 7110   7110    0 480 24   1    1  1   1   0
## 7111   7111    0 480 24   1    1  1   1   0
## 7112   7112    0 480 24   1    1  1   1   0
## 7113   7113    0 480 24   1    1  1   1   0
## 7114   7114    0 480 24   1    1  1   1   0
## 7115   7115    0 480 24   1    1  1   1   0
## 7116   7116    0 480 24   1    1  1   1   0
## 7117   7117    0 480 24   1    1  1   1   0
## 7118   7118    0 480 24   1    1  1   1   0
## 7119   7119    0 480 24   1    1  1   1   0
## 7120   7120    0 480 24   1    1  1   1   0
## 7121   7121    0 480 24   1    1  1   1   0
## 7122   7122    0 480 24   1    1  1   1   0
## 7123   7123    0 480 24   1    1  1   1   0
## 7124   7124    0 480 24   1    1  1   1   0
## 7125   7125    0 480 24   1    1  1   1   0
## 7126   7126    0 480 24   1    1  1   1   0
## 7127   7127    0 480 24   1    1  1   1   0
## 7128   7128    0 480 24   1    1  1   1   0
## 7129   7129    0 480 24   1    1  1   1   0
## 7130   7130    0 480 24   1    1  1   1   0
## 7131   7131    0 480 24   1    1  1   1   0
## 7132   7132    0 480 24   1    1  1   1   0
## 7133   7133    0 480 24   1    1  1   1   0
## 7134   7134    0 480 24   1    1  1   1   0
## 7135   7135    0 480 24   1    1  1   1   0
## 7136   7136    0 480 24   1    1  1   1   0
## 7137   7137    0 480 24   1    1  1   1   0
## 7138   7138    0 480 24   1    1  1   1   0
## 7139   7139    0 480 24   1    1  1   1   0
## 7140   7140    0 480 24   1    1  1   1   0
## 7141   7141    0 480 24   1    1  1   1   0
## 7142   7142    0 480 24   1    1  1   1   0
## 7143   7143    0 480 24   1    1  1   1   0
## 7144   7144    0 480 24   1    1  1   1   0
## 7145   7145    0 480 24   1    1  1   1   0
## 7146   7146    0 480 24   1    1  1   1   0
## 7147   7147    0 480 24   1    1  1   1   0
## 7148   7148    0 480 24   1    1  1   1   0
## 7149   7149    0 480 24   1    1  1   1   0
## 7150   7150    0 480 24   1    1  1   1   0
## 7151   7151    0 480 24   1    1  1   1   0
## 7152   7152    0 480 24   1    1  1   1   0
## 7153   7153    0 480 24   1    1  1   1   0
## 7154   7154    0 480 24   1    1  1   1   0
## 7155   7155    0 480 24   1    1  1   1   0
## 7156   7156    0 480 24   1    1  1   1   0
## 7157   7157    0 480 24   1    1  1   1   0
## 7158   7158    0 480 24   1    1  1   1   0
## 7159   7159    0 480 24   1    1  1   1   0
## 7160   7160    0 480 24   1    1  1   1   0
## 7161   7161    0 480 24   1    1  1   1   0
## 7162   7162    0 480 24   1    1  1   1   0
## 7163   7163    0 480 24   1    1  1   1   0
## 7164   7164    0 480 24   1    1  1   1   0
## 7165   7165    0 480 24   1    1  1   1   0
## 7166   7166    0 480 24   1    1  1   1   0
## 7167   7167    0 480 24   1    1  1   1   0
## 7168   7168    0 480 24   1    1  1   1   0
## 7169   7169    0 480 24   1    1  1   1   0
## 7170   7170    0 480 24   1    1  1   1   0
## 7171   7171    0 480 24   1    1  1   1   0
## 7172   7172    0 480 24   1    1  1   1   0
## 7173   7173    0 480 24   1    1  1   1   0
## 7174   7174    0 480 24   1    1  1   1   0
## 7175   7175    0 480 24   1    1  1   1   0
## 7176   7176    0 480 24   1    1  1   1   0
## 7177   7177    0 480 24   1    1  1   1   0
## 7178   7178    0 480 24   1    1  1   1   0
## 7179   7179    0 480 24   1    1  1   1   0
## 7180   7180    0 480 24   1    1  1   1   0
## 7181   7181    0 480 24   1    1  1   1   0
## 7182   7182    0 480 24   1    1  1   1   0
## 7183   7183    0 480 24   1    1  1   1   0
## 7184   7184    0 480 24   1    1  1   1   0
## 7185   7185    0 480 24   1    1  1   1   0
## 7186   7186    0 480 24   1    1  1   1   0
## 7187   7187    0 480 24   1    1  1   1   0
## 7188   7188    0 480 24   1    1  1   1   0
## 7189   7189    0 480 24   1    1  1   1   0
## 7190   7190    0 480 24   1    1  1   1   0
## 7191   7191    0 480 24   1    1  1   1   0
## 7192   7192    0 480 24   1    1  1   1   0
## 7193   7193    0 480 24   1    1  1   1   0
## 7194   7194    0 480 24   1    1  1   1   0
## 7195   7195    0 480 24   1    1  1   1   0
## 7196   7196    0 480 24   1    1  1   1   0
## 7197   7197    0 480 24   1    1  1   1   0
## 7198   7198    0 480 24   1    1  1   1   0
## 7199   7199    0 480 24   1    1  1   1   0
## 7200   7200    0 480 24   1    1  1   1   0
## 7201   7201    0 480 24   1    1  1   1   0
## 7202   7202    0 480 24   1    1  1   1   0
## 7203   7203    0 480 24   1    1  1   1   0
## 7204   7204    0 480 24   1    1  1   1   0
## 7205   7205    0 480 24   1    1  1   1   0
## 7206   7206    0 480 24   1    1  1   1   0
## 7207   7207    0 480 24   1    1  1   1   0
## 7208   7208    0 480 24   1    1  1   1   0
## 7209   7209    0 480 24   1    1  1   1   0
## 7210   7210    0 480 24   1    1  1   1   0
## 7211   7211    0 480 24   1    1  1   1   0
## 7212   7212    0 480 24   1    1  1   1   0
## 7213   7213    0 480 24   1    1  1   1   0
## 7214   7214    0 480 24   1    1  1   1   0
## 7215   7215    0 480 24   1    1  1   1   0
## 7216   7216    0 480 24   1    1  1   1   0
## 7217   7217    0 480 24   1    1  1   1   0
## 7218   7218    0 480 24   1    1  1   1   0
## 7219   7219    0 480 24   1    1  1   1   0
## 7220   7220    0 480 24   1    1  1   1   0
## 7221   7221    0 480 24   1    1  1   1   0
## 7222   7222    0 480 24   1    1  1   1   0
## 7223   7223    0 480 24   1    1  1   1   0
## 7224   7224    0 480 24   1    1  1   1   0
## 7225   7225    0 480 24   1    1  1   1   0
## 7226   7226    0 480 24   1    1  1   1   0
## 7227   7227    0 480 24   1    1  1   1   0
## 7228   7228    0 480 24   1    1  1   1   0
## 7229   7229    0 480 24   1    1  1   1   0
## 7230   7230    0 480 24   1    1  1   1   0
## 7231   7231    0 480 24   1    1  1   1   0
## 7232   7232    0 480 24   1    1  1   1   0
## 7233   7233    0 480 24   1    1  1   1   0
## 7234   7234    0 480 24   1    1  1   1   0
## 7235   7235    0 480 24   1    1  1   1   0
## 7236   7236    0 480 24   1    1  1   1   0
## 7237   7237    0 480 24   1    1  1   1   0
## 7238   7238    0 480 24   1    1  1   1   0
## 7239   7239    0 480 24   1    1  1   1   0
## 7240   7240    0 480 24   1    1  1   1   0
## 7241   7241    0 480 24   1    1  1   1   0
## 7242   7242    0 480 24   1    1  1   1   0
## 7243   7243    0 480 24   1    1  1   1   0
## 7244   7244    0 480 24   1    1  1   1   0
## 7245   7245    0 480 24   1    1  1   1   0
## 7246   7246    0 480 24   1    1  1   1   0
## 7247   7247    0 480 24   1    1  1   1   0
## 7248   7248    0 480 24   1    1  1   1   0
## 7249   7249    0 480 24   1    1  1   1   0
## 7250   7250    0 480 24   1    1  1   1   0
## 7251   7251    0 480 24   1    1  1   1   0
## 7252   7252    0 480 24   1    1  1   1   0
## 7253   7253    0 480 24   1    1  1   1   0
## 7254   7254    0 480 24   1    1  1   1   0
## 7255   7255    0 480 24   1    1  1   1   0
## 7256   7256    0 480 24   1    1  1   1   0
## 7257   7257    0 480 24   1    1  1   1   0
## 7258   7258    0 480 24   1    1  1   1   0
## 7259   7259    0 480 24   1    1  1   1   0
## 7260   7260    0 480 24   1    1  1   1   0
## 7261   7261    0 480 24   1    1  1   1   0
## 7262   7262    0 480 24   1    1  1   1   0
## 7263   7263    0 480 24   1    1  1   1   0
## 7264   7264    0 480 24   1    1  1   1   0
## 7265   7265    0 480 24   1    1  1   1   0
## 7266   7266    0 480 24   1    1  1   1   0
## 7267   7267    0 480 24   1    1  1   1   0
## 7268   7268    0 480 24   1    1  1   1   0
## 7269   7269    0 480 24   1    1  1   1   0
## 7270   7270    0 480 24   1    1  1   1   0
## 7271   7271    0 480 24   1    1  1   1   0
## 7272   7272    0 480 24   1    1  1   1   0
## 7273   7273    0 480 24   1    1  1   1   0
## 7274   7274    0 480 24   1    1  1   1   0
## 7275   7275    0 480 24   1    1  1   1   0
## 7276   7276    0 480 24   1    1  1   1   0
## 7277   7277    0 480 24   1    1  1   1   0
## 7278   7278    0 480 24   1    1  1   1   0
## 7279   7279    0 480 24   1    1  1   1   0
## 7280   7280    0 480 24   1    1  1   1   0
## 7281   7281    0 480 24   1    1  1   1   0
## 7282   7282    0 480 24   1    1  1   1   0
## 7283   7283    0 480 24   1    1  1   1   0
## 7284   7284    0 480 24   1    1  1   1   0
## 7285   7285    0 480 24   1    1  1   1   0
## 7286   7286    0 480 24   1    1  1   1   0
## 7287   7287    0 480 24   1    1  1   1   0
## 7288   7288    0 480 24   1    1  1   1   0
## 7289   7289    0 480 24   1    1  1   1   0
## 7290   7290    0 480 24   1    1  1   1   0
## 7291   7291    0 480 24   1    1  1   1   0
## 7292   7292    0 480 24   1    1  1   1   0
## 7293   7293    0 480 24   1    1  1   1   0
## 7294   7294    0 480 24   1    1  1   1   0
## 7295   7295    0 480 24   1    1  1   1   0
## 7296   7296    0 480 24   1    1  1   1   0
## 7297   7297    0 480 24   1    1  1   1   0
## 7298   7298    0 480 24   1    1  1   1   0
## 7299   7299    0 480 24   1    1  1   1   0
## 7300   7300    0 480 24   1    1  1   1   0
## 7301   7301    0 480 24   1    1  1   1   0
## 7302   7302    0 480 24   1    1  1   1   0
## 7303   7303    0 480 24   1    1  1   1   0
## 7304   7304    0 480 24   1    1  1   1   0
## 7305   7305    0 480 24   1    1  1   1   0
## 7306   7306    0 480 24   1    1  1   1   0
## 7307   7307    0 480 24   1    1  1   1   0
## 7308   7308    0 480 24   1    1  1   1   0
## 7309   7309    0 480 24   1    1  1   1   0
## 7310   7310    0 480 24   1    1  1   1   0
## 7311   7311    0 480 24   1    1  1   1   0
## 7312   7312    0 480 24   1    1  1   1   0
## 7313   7313    0 480 24   1    1  1   1   0
## 7314   7314    0 480 24   1    1  1   1   0
## 7315   7315    0 480 24   1    1  1   1   0
## 7316   7316    0 480 24   1    1  1   1   0
## 7317   7317    0 480 24   1    1  1   1   0
## 7318   7318    0 480 24   1    1  1   1   0
## 7319   7319    0 480 24   1    1  1   1   0
## 7320   7320    0 480 24   1    1  1   1   0
## 7321   7321    0 480 24   1    1  1   1   0
## 7322   7322    0 480 24   1    1  1   1   0
## 7323   7323    0 480 24   1    1  1   1   0
## 7324   7324    0 480 24   1    1  1   1   0
## 7325   7325    0 480 24   1    1  1   1   0
## 7326   7326    0 480 24   1    1  1   1   0
## 7327   7327    0 480 24   1    1  1   1   0
## 7328   7328    0 480 24   1    1  1   1   0
## 7329   7329    0 480 24   1    1  1   1   0
## 7330   7330    0 480 24   1    1  1   1   0
## 7331   7331    0 480 24   1    1  1   1   0
## 7332   7332    0 480 24   1    1  1   1   0
## 7333   7333    0 480 24   1    1  1   1   0
## 7334   7334    0 480 24   1    1  1   1   0
## 7335   7335    0 480 24   1    1  1   1   0
## 7336   7336    0 480 24   1    1  1   1   0
## 7337   7337    0 480 24   1    1  1   1   0
## 7338   7338    0 480 24   1    1  1   1   0
## 7339   7339    0 480 24   1    1  1   1   0
## 7340   7340    0 480 24   1    1  1   1   0
## 7341   7341    0 480 24   1    1  1   1   0
## 7342   7342    0 480 24   1    1  1   1   0
## 7343   7343    0 480 24   1    1  1   1   0
## 7344   7344    0 480 24   1    1  1   1   0
## 7345   7345    0 480 24   1    1  1   1   0
## 7346   7346    0 480 24   1    1  1   1   0
## 7347   7347    0 480 24   1    1  1   1   0
## 7348   7348    0 480 24   1    1  1   1   0
## 7349   7349    0 480 24   1    1  1   1   0
## 7350   7350    0 480 24   1    1  1   1   0
## 7351   7351    0 480 24   1    1  1   1   0
## 7352   7352    0 480 24   1    1  1   1   0
## 7353   7353    0 480 24   1    1  1   1   0
## 7354   7354    0 480 24   1    1  1   1   0
## 7355   7355    0 480 24   1    1  1   1   0
## 7356   7356    0 480 24   1    1  1   1   0
## 7357   7357    0 480 24   1    1  1   1   0
## 7358   7358    0 480 24   1    1  1   1   0
## 7359   7359    0 480 24   1    1  1   1   0
## 7360   7360    0 480 24   1    1  1   1   0
## 7361   7361    0 480 24   1    1  1   1   0
## 7362   7362    0 480 24   1    1  1   1   0
## 7363   7363    0 480 24   1    1  1   1   0
## 7364   7364    0 480 24   1    1  1   1   0
## 7365   7365    0 480 24   1    1  1   1   0
## 7366   7366    0 480 24   1    1  1   1   0
## 7367   7367    0 480 24   1    1  1   1   0
## 7368   7368    0 480 24   1    1  1   1   0
## 7369   7369    0 480 24   1    1  1   1   0
## 7370   7370    0 480 24   1    1  1   1   0
## 7371   7371    0 480 24   1    1  1   1   0
## 7372   7372    0 480 24   1    1  1   1   0
## 7373   7373    0 480 24   1    1  1   1   0
## 7374   7374    0 480 24   1    1  1   1   0
## 7375   7375    0 480 24   1    1  1   1   0
## 7376   7376    0 480 24   1    1  1   1   0
## 7377   7377    0 480 24   1    1  1   1   0
## 7378   7378    0 480 24   1    1  1   1   0
## 7379   7379    0 480 24   1    1  1   1   0
## 7380   7380    0 480 24   1    1  1   1   0
## 7381   7381    0 480 24   1    1  1   1   0
## 7382   7382    0 480 24   1    1  1   1   0
## 7383   7383    0 480 24   1    1  1   1   0
## 7384   7384    0 480 24   1    1  1   1   0
## 7385   7385    0 480 24   1    1  1   1   0
## 7386   7386    0 480 24   1    1  1   1   0
## 7387   7387    0 480 24   1    1  1   1   0
## 7388   7388    0 480 24   1    1  1   1   0
## 7389   7389    0 480 24   1    1  1   1   0
## 7390   7390    0 480 24   1    1  1   1   0
## 7391   7391    0 480 24   1    1  1   1   0
## 7392   7392    0 480 24   1    1  1   1   0
## 7393   7393    0 480 24   1    1  1   1   0
## 7394   7394    0 480 24   1    1  1   1   0
## 7395   7395    0 480 24   1    1  1   1   0
## 7396   7396    0 480 24   1    1  1   1   0
## 7397   7397    0 480 24   1    1  1   1   0
## 7398   7398    0 480 24   1    1  1   1   0
## 7399   7399    0 480 24   1    1  1   1   0
## 7400   7400    0 480 24   1    1  1   1   0
## 7401   7401    0 480 24   1    1  1   1   0
## 7402   7402    0 480 24   1    1  1   1   0
## 7403   7403    0 480 24   1    1  1   1   0
## 7404   7404    0 480 24   1    1  1   1   0
## 7405   7405    0 480 24   1    1  1   1   0
## 7406   7406    0 480 24   1    1  1   1   0
## 7407   7407    0 480 24   1    1  1   1   0
## 7408   7408    0 480 24   1    1  1   1   0
## 7409   7409    0 480 24   1    1  1   1   0
## 7410   7410    0 480 24   1    1  1   1   0
## 7411   7411    0 480 24   1    1  1   1   0
## 7412   7412    0 480 24   1    1  1   1   0
## 7413   7413    0 480 24   1    1  1   1   0
## 7414   7414    0 480 24   1    1  1   1   0
## 7415   7415    0 480 24   1    1  1   1   0
## 7416   7416    0 480 24   1    1  1   1   0
## 7417   7417    0 480 24   1    1  1   1   0
## 7418   7418    0 480 24   1    1  1   1   0
## 7419   7419    0 480 24   1    1  1   1   0
## 7420   7420    0 480 24   1    1  1   1   0
## 7421   7421    0 480 24   1    1  1   1   0
## 7422   7422    0 480 24   1    1  1   1   0
## 7423   7423    0 480 24   1    1  1   1   0
## 7424   7424    0 480 24   1    1  1   1   0
## 7425   7425    0 480 24   1    1  1   1   0
## 7426   7426    0 480 24   1    1  1   1   0
## 7427   7427    0 480 24   1    1  1   1   0
## 7428   7428    0 480 24   1    1  1   1   0
## 7429   7429    0 480 24   1    1  1   1   0
## 7430   7430    0 480 24   1    1  1   1   0
## 7431   7431    0 480 24   1    1  1   1   0
## 7432   7432    0 480 24   1    1  1   1   0
## 7433   7433    0 480 24   1    1  1   1   0
## 7434   7434    0 480 24   1    1  1   1   0
## 7435   7435    0 480 24   1    1  1   1   0
## 7436   7436    0 480 24   1    1  1   1   0
## 7437   7437    0 480 24   1    1  1   1   0
## 7438   7438    0 480 24   1    1  1   1   0
## 7439   7439    0 480 24   1    1  1   1   0
## 7440   7440    0 480 24   1    1  1   1   0
## 7441   7441    0 480 24   1    1  1   1   0
## 7442   7442    0 480 24   1    1  1   1   0
## 7443   7443    0 480 24   1    1  1   1   0
## 7444   7444    0 480 24   1    1  1   1   0
## 7445   7445    0 480 24   1    1  1   1   0
## 7446   7446    0 480 24   1    1  1   1   0
## 7447   7447    0 480 24   1    1  1   1   0
## 7448   7448    0 480 24   1    1  1   1   0
## 7449   7449    0 480 24   1    1  1   1   0
## 7450   7450    0 480 24   1    1  1   1   0
## 7451   7451    0 480 24   1    1  1   1   0
## 7452   7452    0 480 24   1    1  1   1   0
## 7453   7453    0 480 24   1    1  1   1   0
## 7454   7454    0 480 24   1    1  1   1   0
## 7455   7455    0 480 24   1    1  1   1   0
## 7456   7456    0 480 24   1    1  1   1   0
## 7457   7457    0 480 24   1    1  1   1   0
## 7458   7458    0 480 24   1    1  1   1   0
## 7459   7459    0 480 24   1    1  1   1   0
## 7460   7460    0 480 24   1    1  1   1   0
## 7461   7461    0 480 24   1    1  1   1   0
## 7462   7462    0 480 24   1    1  1   1   0
## 7463   7463    0 480 24   1    1  1   1   0
## 7464   7464    0 480 24   1    1  1   1   0
## 7465   7465    0 480 24   1    1  1   1   0
## 7466   7466    0 480 24   1    1  1   1   0
## 7467   7467    0 480 24   1    1  1   1   0
## 7468   7468    0 480 24   1    1  1   1   0
## 7469   7469    0 480 24   1    1  1   1   0
## 7470   7470    0 480 24   1    1  1   1   0
## 7471   7471    0 480 24   1    1  1   1   0
## 7472   7472    0 480 24   1    1  1   1   0
## 7473   7473    0 480 24   1    1  1   1   0
## 7474   7474    0 480 24   1    1  1   1   0
## 7475   7475    0 480 24   1    1  1   1   0
## 7476   7476    0 480 24   1    1  1   1   0
## 7477   7477    0 480 24   1    1  1   1   0
## 7478   7478    0 480 24   1    1  1   1   0
## 7479   7479    0 480 24   1    1  1   1   0
## 7480   7480    0 480 24   1    1  1   1   0
## 7481   7481    0 480 24   1    1  1   1   0
## 7482   7482    0 480 24   1    1  1   1   0
## 7483   7483    0 480 24   1    1  1   1   0
## 7484   7484    0 480 24   1    1  1   1   0
## 7485   7485    0 480 24   1    1  1   1   0
## 7486   7486    0 480 24   1    1  1   1   0
## 7487   7487    0 480 24   1    1  1   1   0
## 7488   7488    0 480 24   1    1  1   1   0
## 7489   7489    0 480 24   1    1  1   1   0
## 7490   7490    0 480 24   1    1  1   1   0
## 7491   7491    0 480 24   1    1  1   1   0
## 7492   7492    0 480 24   1    1  1   1   0
## 7493   7493    0 480 24   1    1  1   1   0
## 7494   7494    0 480 24   1    1  1   1   0
## 7495   7495    0 480 24   1    1  1   1   0
## 7496   7496    0 480 24   1    1  1   1   0
## 7497   7497    0 480 24   1    1  1   1   0
## 7498   7498    0 480 24   1    1  1   1   0
## 7499   7499    0 480 24   1    1  1   1   0
## 7500   7500    0 480 24   1    1  1   1   0
## 7501   7501    0 480 24   1    1  1   1   0
## 7502   7502    0 480 24   1    1  1   1   0
## 7503   7503    0 480 24   1    1  1   1   0
## 7504   7504    0 480 24   1    1  1   1   0
## 7505   7505    0 480 24   1    1  1   1   0
## 7506   7506    0 480 24   1    1  1   1   0
## 7507   7507    0 480 24   1    1  1   1   0
## 7508   7508    0 480 24   1    1  1   1   0
## 7509   7509    0 480 24   1    1  1   1   0
## 7510   7510    0 480 24   1    1  1   1   0
## 7511   7511    0 480 24   1    1  1   1   0
## 7512   7512    0 480 24   1    1  1   1   0
## 7513   7513    0 480 24   1    1  1   1   0
## 7514   7514    0 480 24   1    1  1   1   0
## 7515   7515    0 480 24   1    1  1   1   0
## 7516   7516    0 480 24   1    1  1   1   0
## 7517   7517    0 480 24   1    1  1   1   0
## 7518   7518    0 480 24   1    1  1   1   0
## 7519   7519    0 480 24   1    1  1   1   0
## 7520   7520    0 480 24   1    1  1   1   0
## 7521   7521    0 480 24   1    1  1   1   0
## 7522   7522    0 480 24   1    1  1   1   0
## 7523   7523    0 480 24   1    1  1   1   0
## 7524   7524    0 480 24   1    1  1   1   0
## 7525   7525    0 480 24   1    1  1   1   0
## 7526   7526    0 480 24   1    1  1   1   0
## 7527   7527    0 480 24   1    1  1   1   0
## 7528   7528    0 480 24   1    1  1   1   0
## 7529   7529    0 480 24   1    1  1   1   0
## 7530   7530    0 480 24   1    1  1   1   0
## 7531   7531    0 480 24   1    1  1   1   0
## 7532   7532    0 480 24   1    1  1   1   0
## 7533   7533    0 480 24   1    1  1   1   0
## 7534   7534    0 480 24   1    1  1   1   0
## 7535   7535    0 480 24   1    1  1   1   0
## 7536   7536    0 480 24   1    1  1   1   0
## 7537   7537    0 480 24   1    1  1   1   0
## 7538   7538    0 480 24   1    1  1   1   0
## 7539   7539    0 480 24   1    1  1   1   0
## 7540   7540    0 480 24   1    1  1   1   0
## 7541   7541    0 480 24   1    1  1   1   0
## 7542   7542    0 480 24   1    1  1   1   0
## 7543   7543    0 480 24   1    1  1   1   0
## 7544   7544    0 480 24   1    1  1   1   0
## 7545   7545    0 480 24   1    1  1   1   0
## 7546   7546    0 480 24   1    1  1   1   0
## 7547   7547    0 480 24   1    1  1   1   0
## 7548   7548    0 480 24   1    1  1   1   0
## 7549   7549    0 480 24   1    1  1   1   0
## 7550   7550    0 480 24   1    1  1   1   0
## 7551   7551    0 480 24   1    1  1   1   0
## 7552   7552    0 480 24   1    1  1   1   0
## 7553   7553    0 480 24   1    1  1   1   0
## 7554   7554    0 480 24   1    1  1   1   0
## 7555   7555    0 480 24   1    1  1   1   0
## 7556   7556    0 480 24   1    1  1   1   0
## 7557   7557    0 480 24   1    1  1   1   0
## 7558   7558    0 480 24   1    1  1   1   0
## 7559   7559    0 480 24   1    1  1   1   0
## 7560   7560    0 480 24   1    1  1   1   0
## 7561   7561    0 480 24   1    1  1   1   0
## 7562   7562    0 480 24   1    1  1   1   0
## 7563   7563    0 480 24   1    1  1   1   0
## 7564   7564    0 480 24   1    1  1   1   0
## 7565   7565    0 480 24   1    1  1   1   0
## 7566   7566    0 480 24   1    1  1   1   0
## 7567   7567    0 480 24   1    1  1   1   0
## 7568   7568    0 480 24   1    1  1   1   0
## 7569   7569    0 480 24   1    1  1   1   0
## 7570   7570    0 480 24   1    1  1   1   0
## 7571   7571    0 480 24   1    1  1   1   0
## 7572   7572    0 480 24   1    1  1   1   0
## 7573   7573    0 480 24   1    1  1   1   0
## 7574   7574    0 480 24   1    1  1   1   0
## 7575   7575    0 480 24   1    1  1   1   0
## 7576   7576    0 480 24   1    1  1   1   0
## 7577   7577    0 480 24   1    1  1   1   0
## 7578   7578    0 480 24   1    1  1   1   0
## 7579   7579    0 480 24   1    1  1   1   0
## 7580   7580    0 480 24   1    1  1   1   0
## 7581   7581    0 480 24   1    1  1   1   0
## 7582   7582    0 480 24   1    1  1   1   0
## 7583   7583    0 480 24   1    1  1   1   0
## 7584   7584    0 480 24   1    1  1   1   0
## 7585   7585    0 480 24   1    1  1   1   0
## 7586   7586    0 480 24   1    1  1   1   0
## 7587   7587    0 480 24   1    1  1   1   0
## 7588   7588    0 480 24   1    1  1   1   0
## 7589   7589    0 480 24   1    1  1   1   0
## 7590   7590    0 480 24   1    1  1   1   0
## 7591   7591    0 480 24   1    1  1   1   0
## 7592   7592    0 480 24   1    1  1   1   0
## 7593   7593    0 480 24   1    1  1   1   0
## 7594   7594    0 480 24   1    1  1   1   0
## 7595   7595    0 480 24   1    1  1   1   0
## 7596   7596    0 480 24   1    1  1   1   0
## 7597   7597    0 480 24   1    1  1   1   0
## 7598   7598    0 480 24   1    1  1   1   0
## 7599   7599    0 480 24   1    1  1   1   0
## 7600   7600    0 480 24   1    1  1   1   0
## 7601   7601    0 480 24   1    1  1   1   0
## 7602   7602    0 480 24   1    1  1   1   0
## 7603   7603    0 480 24   1    1  1   1   0
## 7604   7604    0 480 24   1    1  1   1   0
## 7605   7605    0 480 24   1    1  1   1   0
## 7606   7606    0 480 24   1    1  1   1   0
## 7607   7607    0 480 24   1    1  1   1   0
## 7608   7608    0 480 24   1    1  1   1   0
## 7609   7609    0 480 24   1    1  1   1   0
## 7610   7610    0 480 24   1    1  1   1   0
## 7611   7611    0 480 24   1    1  1   1   0
## 7612   7612    0 480 24   1    1  1   1   0
## 7613   7613    0 480 24   1    1  1   1   0
## 7614   7614    0 480 24   1    1  1   1   0
## 7615   7615    0 480 24   1    1  1   1   0
## 7616   7616    0 480 24   1    1  1   1   0
## 7617   7617    0 480 24   1    1  1   1   0
## 7618   7618    0 480 24   1    1  1   1   0
## 7619   7619    0 480 24   1    1  1   1   0
## 7620   7620    0 480 24   1    1  1   1   0
## 7621   7621    0 480 24   1    1  1   1   0
## 7622   7622    0 480 24   1    1  1   1   0
## 7623   7623    0 480 24   1    1  1   1   0
## 7624   7624    0 480 24   1    1  1   1   0
## 7625   7625    0 480 24   1    1  1   1   0
## 7626   7626    0 480 24   1    1  1   1   0
## 7627   7627    0 480 24   1    1  1   1   0
## 7628   7628    0 480 24   1    1  1   1   0
## 7629   7629    0 480 24   1    1  1   1   0
## 7630   7630    0 480 24   1    1  1   1   0
## 7631   7631    0 480 24   1    1  1   1   0
## 7632   7632    0 480 24   1    1  1   1   0
## 7633   7633    0 480 24   1    1  1   1   0
## 7634   7634    0 480 24   1    1  1   1   0
## 7635   7635    0 480 24   1    1  1   1   0
## 7636   7636    0 480 24   1    1  1   1   0
## 7637   7637    0 480 24   1    1  1   1   0
## 7638   7638    0 480 24   1    1  1   1   0
## 7639   7639    0 480 24   1    1  1   1   0
## 7640   7640    0 480 24   1    1  1   1   0
## 7641   7641    0 480 24   1    1  1   1   0
## 7642   7642    0 480 24   1    1  1   1   0
## 7643   7643    0 480 24   1    1  1   1   0
## 7644   7644    0 480 24   1    1  1   1   0
## 7645   7645    0 480 24   1    1  1   1   0
## 7646   7646    0 480 24   1    1  1   1   0
## 7647   7647    0 480 24   1    1  1   1   0
## 7648   7648    0 480 24   1    1  1   1   0
## 7649   7649    0 480 24   1    1  1   1   0
## 7650   7650    0 480 24   1    1  1   1   0
## 7651   7651    0 480 24   1    1  1   1   0
## 7652   7652    0 480 24   1    1  1   1   0
## 7653   7653    0 480 24   1    1  1   1   0
## 7654   7654    0 480 24   1    1  1   1   0
## 7655   7655    0 480 24   1    1  1   1   0
## 7656   7656    0 480 24   1    1  1   1   0
## 7657   7657    0 480 24   1    1  1   1   0
## 7658   7658    0 480 24   1    1  1   1   0
## 7659   7659    0 480 24   1    1  1   1   0
## 7660   7660    0 480 24   1    1  1   1   0
## 7661   7661    0 480 24   1    1  1   1   0
## 7662   7662    0 480 24   1    1  1   1   0
## 7663   7663    0 480 24   1    1  1   1   0
## 7664   7664    0 480 24   1    1  1   1   0
## 7665   7665    0 480 24   1    1  1   1   0
## 7666   7666    0 480 24   1    1  1   1   0
## 7667   7667    0 480 24   1    1  1   1   0
## 7668   7668    0 480 24   1    1  1   1   0
## 7669   7669    0 480 24   1    1  1   1   0
## 7670   7670    0 480 24   1    1  1   1   0
## 7671   7671    0 480 24   1    1  1   1   0
## 7672   7672    0 480 24   1    1  1   1   0
## 7673   7673    0 480 24   1    1  1   1   0
## 7674   7674    0 480 24   1    1  1   1   0
## 7675   7675    0 480 24   1    1  1   1   0
## 7676   7676    0 480 24   1    1  1   1   0
## 7677   7677    0 480 24   1    1  1   1   0
## 7678   7678    0 480 24   1    1  1   1   0
## 7679   7679    0 480 24   1    1  1   1   0
## 7680   7680    0 480 24   1    1  1   1   0
## 7681   7681    0 480 24   1    1  1   1   0
## 7682   7682    0 480 24   1    1  1   1   0
## 7683   7683    0 480 24   1    1  1   1   0
## 7684   7684    0 480 24   1    1  1   1   0
## 7685   7685    0 480 24   1    1  1   1   0
## 7686   7686    0 480 24   1    1  1   1   0
## 7687   7687    0 480 24   1    1  1   1   0
## 7688   7688    0 480 24   1    1  1   1   0
## 7689   7689    0 480 24   1    1  1   1   0
## 7690   7690    0 480 24   1    1  1   1   0
## 7691   7691    0 480 24   1    1  1   1   0
## 7692   7692    0 480 24   1    1  1   1   0
## 7693   7693    0 480 24   1    1  1   1   0
## 7694   7694    0 480 24   1    1  1   1   0
## 7695   7695    0 480 24   1    1  1   1   0
## 7696   7696    0 480 24   1    1  1   1   0
## 7697   7697    0 480 24   1    1  1   1   0
## 7698   7698    0 480 24   1    1  1   1   0
## 7699   7699    0 480 24   1    1  1   1   0
## 7700   7700    0 480 24   1    1  1   1   0
## 7701   7701    0 480 24   1    1  1   1   0
## 7702   7702    0 480 24   1    1  1   1   0
## 7703   7703    0 480 24   1    1  1   1   0
## 7704   7704    0 480 24   1    1  1   1   0
## 7705   7705    0 480 24   1    1  1   1   0
## 7706   7706    0 480 24   1    1  1   1   0
## 7707   7707    0 480 24   1    1  1   1   0
## 7708   7708    0 480 24   1    1  1   1   0
## 7709   7709    0 480 24   1    1  1   1   0
## 7710   7710    0 480 24   1    1  1   1   0
## 7711   7711    0 480 24   1    1  1   1   0
## 7712   7712    0 480 24   1    1  1   1   0
## 7713   7713    0 480 24   1    1  1   1   0
## 7714   7714    0 480 24   1    1  1   1   0
## 7715   7715    0 480 24   1    1  1   1   0
## 7716   7716    0 480 24   1    1  1   1   0
## 7717   7717    0 480 24   1    1  1   1   0
## 7718   7718    0 480 24   1    1  1   1   0
## 7719   7719    0 480 24   1    1  1   1   0
## 7720   7720    0 480 24   1    1  1   1   0
## 7721   7721    0 480 24   1    1  1   1   0
## 7722   7722    0 480 24   1    1  1   1   0
## 7723   7723    0 480 24   1    1  1   1   0
## 7724   7724    0 480 24   1    1  1   1   0
## 7725   7725    0 480 24   1    1  1   1   0
## 7726   7726    0 480 24   1    1  1   1   0
## 7727   7727    0 480 24   1    1  1   1   0
## 7728   7728    0 480 24   1    1  1   1   0
## 7729   7729    0 480 24   1    1  1   1   0
## 7730   7730    0 480 24   1    1  1   1   0
## 7731   7731    0 480 24   1    1  1   1   0
## 7732   7732    0 480 24   1    1  1   1   0
## 7733   7733    0 480 24   1    1  1   1   0
## 7734   7734    0 480 24   1    1  1   1   0
## 7735   7735    0 480 24   1    1  1   1   0
## 7736   7736    0 480 24   1    1  1   1   0
## 7737   7737    0 480 24   1    1  1   1   0
## 7738   7738    0 480 24   1    1  1   1   0
## 7739   7739    0 480 24   1    1  1   1   0
## 7740   7740    0 480 24   1    1  1   1   0
## 7741   7741    0 480 24   1    1  1   1   0
## 7742   7742    0 480 24   1    1  1   1   0
## 7743   7743    0 480 24   1    1  1   1   0
## 7744   7744    0 480 24   1    1  1   1   0
## 7745   7745    0 480 24   1    1  1   1   0
## 7746   7746    0 480 24   1    1  1   1   0
## 7747   7747    0 480 24   1    1  1   1   0
## 7748   7748    0 480 24   1    1  1   1   0
## 7749   7749    0 480 24   1    1  1   1   0
## 7750   7750    0 480 24   1    1  1   1   0
## 7751   7751    0 480 24   1    1  1   1   0
## 7752   7752    0 480 24   1    1  1   1   0
## 7753   7753    0 480 24   1    1  1   1   0
## 7754   7754    0 480 24   1    1  1   1   0
## 7755   7755    0 480 24   1    1  1   1   0
## 7756   7756    0 480 24   1    1  1   1   0
## 7757   7757    0 480 24   1    1  1   1   0
## 7758   7758    0 480 24   1    1  1   1   0
## 7759   7759    0 480 24   1    1  1   1   0
## 7760   7760    0 480 24   1    1  1   1   0
## 7761   7761    0 480 24   1    1  1   1   0
## 7762   7762    0 480 24   1    1  1   1   0
## 7763   7763    0 480 24   1    1  1   1   0
## 7764   7764    0 480 24   1    1  1   1   0
## 7765   7765    0 480 24   1    1  1   1   0
## 7766   7766    0 480 24   1    1  1   1   0
## 7767   7767    0 480 24   1    1  1   1   0
## 7768   7768    0 480 24   1    1  1   1   0
## 7769   7769    0 480 24   1    1  1   1   0
## 7770   7770    0 480 24   1    1  1   1   0
## 7771   7771    0 480 24   1    1  1   1   0
## 7772   7772    0 480 24   1    1  1   1   0
## 7773   7773    0 480 24   1    1  1   1   0
## 7774   7774    0 480 24   1    1  1   1   0
## 7775   7775    0 480 24   1    1  1   1   0
## 7776   7776    0 480 24   1    1  1   1   0
## 7777   7777    0 480 24   1    1  1   1   0
## 7778   7778    0 480 24   1    1  1   1   0
## 7779   7779    0 480 24   1    1  1   1   0
## 7780   7780    0 480 24   1    1  1   1   0
## 7781   7781    0 480 24   1    1  1   1   0
## 7782   7782    0 480 24   1    1  1   1   0
## 7783   7783    0 480 24   1    1  1   1   0
## 7784   7784    0 480 24   1    1  1   1   0
## 7785   7785    0 480 24   1    1  1   1   0
## 7786   7786    0 480 24   1    1  1   1   0
## 7787   7787    0 480 24   1    1  1   1   0
## 7788   7788    0 480 24   1    1  1   1   0
## 7789   7789    0 480 24   1    1  1   1   0
## 7790   7790    0 480 24   1    1  1   1   0
## 7791   7791    0 480 24   1    1  1   1   0
## 7792   7792    0 480 24   1    1  1   1   0
## 7793   7793    0 480 24   1    1  1   1   0
## 7794   7794    0 480 24   1    1  1   1   0
## 7795   7795    0 480 24   1    1  1   1   0
## 7796   7796    0 480 24   1    1  1   1   0
## 7797   7797    0 480 24   1    1  1   1   0
## 7798   7798    0 480 24   1    1  1   1   0
## 7799   7799    0 480 24   1    1  1   1   0
## 7800   7800    0 480 24   1    1  1   1   0
## 7801   7801    0 480 24   1    1  1   1   0
## 7802   7802    0 480 24   1    1  1   1   0
## 7803   7803    0 480 24   1    1  1   1   0
## 7804   7804    0 480 24   1    1  1   1   0
## 7805   7805    0 480 24   1    1  1   1   0
## 7806   7806    0 480 24   1    1  1   1   0
## 7807   7807    0 480 24   1    1  1   1   0
## 7808   7808    0 480 24   1    1  1   1   0
## 7809   7809    0 480 24   1    1  1   1   0
## 7810   7810    0 480 24   1    1  1   1   0
## 7811   7811    0 480 24   1    1  1   1   0
## 7812   7812    0 480 24   1    1  1   1   0
## 7813   7813    0 480 24   1    1  1   1   0
## 7814   7814    0 480 24   1    1  1   1   0
## 7815   7815    0 480 24   1    1  1   1   0
## 7816   7816    0 480 24   1    1  1   1   0
## 7817   7817    0 480 24   1    1  1   1   0
## 7818   7818    0 480 24   1    1  1   1   0
## 7819   7819    0 480 24   1    1  1   1   0
## 7820   7820    0 480 24   1    1  1   1   0
## 7821   7821    0 480 24   1    1  1   1   0
## 7822   7822    0 480 24   1    1  1   1   0
## 7823   7823    0 480 24   1    1  1   1   0
## 7824   7824    0 480 24   1    1  1   1   0
## 7825   7825    0 480 24   1    1  1   1   0
## 7826   7826    0 480 24   1    1  1   1   0
## 7827   7827    0 480 24   1    1  1   1   0
## 7828   7828    0 480 24   1    1  1   1   0
## 7829   7829    0 480 24   1    1  1   1   0
## 7830   7830    0 480 24   1    1  1   1   0
## 7831   7831    0 480 24   1    1  1   1   0
## 7832   7832    0 480 24   1    1  1   1   0
## 7833   7833    0 480 24   1    1  1   1   0
## 7834   7834    0 480 24   1    1  1   1   0
## 7835   7835    0 480 24   1    1  1   1   0
## 7836   7836    0 480 24   1    1  1   1   0
## 7837   7837    0 480 24   1    1  1   1   0
## 7838   7838    0 480 24   1    1  1   1   0
## 7839   7839    0 480 24   1    1  1   1   0
## 7840   7840    0 480 24   1    1  1   1   0
## 7841   7841    0 480 24   1    1  1   1   0
## 7842   7842    0 480 24   1    1  1   1   0
## 7843   7843    0 480 24   1    1  1   1   0
## 7844   7844    0 480 24   1    1  1   1   0
## 7845   7845    0 480 24   1    1  1   1   0
## 7846   7846    0 480 24   1    1  1   1   0
## 7847   7847    0 480 24   1    1  1   1   0
## 7848   7848    0 480 24   1    1  1   1   0
## 7849   7849    0 480 24   1    1  1   1   0
## 7850   7850    0 480 24   1    1  1   1   0
## 7851   7851    0 480 24   1    1  1   1   0
## 7852   7852    0 480 24   1    1  1   1   0
## 7853   7853    0 480 24   1    1  1   1   0
## 7854   7854    0 480 24   1    1  1   1   0
## 7855   7855    0 480 24   1    1  1   1   0
## 7856   7856    0 480 24   1    1  1   1   0
## 7857   7857    0 480 24   1    1  1   1   0
## 7858   7858    0 480 24   1    1  1   1   0
## 7859   7859    0 480 24   1    1  1   1   0
## 7860   7860    0 480 24   1    1  1   1   0
## 7861   7861    0 480 24   1    1  1   1   0
## 7862   7862    0 480 24   1    1  1   1   0
## 7863   7863    0 480 24   1    1  1   1   0
## 7864   7864    0 480 24   1    1  1   1   0
## 7865   7865    0 480 24   1    1  1   1   0
## 7866   7866    0 480 24   1    1  1   1   0
## 7867   7867    0 480 24   1    1  1   1   0
## 7868   7868    0 480 24   1    1  1   1   0
## 7869   7869    0 480 24   1    1  1   1   0
## 7870   7870    0 480 24   1    1  1   1   0
## 7871   7871    0 480 24   1    1  1   1   0
## 7872   7872    0 480 24   1    1  1   1   0
## 7873   7873    0 480 24   1    1  1   1   0
## 7874   7874    0 480 24   1    1  1   1   0
## 7875   7875    0 480 24   1    1  1   1   0
## 7876   7876    0 480 24   1    1  1   1   0
## 7877   7877    0 480 24   1    1  1   1   0
## 7878   7878    0 480 24   1    1  1   1   0
## 7879   7879    0 480 24   1    1  1   1   0
## 7880   7880    0 480 24   1    1  1   1   0
## 7881   7881    0 480 24   1    1  1   1   0
## 7882   7882    0 480 24   1    1  1   1   0
## 7883   7883    0 480 24   1    1  1   1   0
## 7884   7884    0 480 24   1    1  1   1   0
## 7885   7885    0 480 24   1    1  1   1   0
## 7886   7886    0 480 24   1    1  1   1   0
## 7887   7887    0 480 24   1    1  1   1   0
## 7888   7888    0 480 24   1    1  1   1   0
## 7889   7889    0 480 24   1    1  1   1   0
## 7890   7890    0 480 24   1    1  1   1   0
## 7891   7891    0 480 24   1    1  1   1   0
## 7892   7892    0 480 24   1    1  1   1   0
## 7893   7893    0 480 24   1    1  1   1   0
## 7894   7894    0 480 24   1    1  1   1   0
## 7895   7895    0 480 24   1    1  1   1   0
## 7896   7896    0 480 24   1    1  1   1   0
## 7897   7897    0 480 24   1    1  1   1   0
## 7898   7898    0 480 24   1    1  1   1   0
## 7899   7899    0 480 24   1    1  1   1   0
## 7900   7900    0 480 24   1    1  1   1   0
## 7901   7901    0 480 24   1    1  1   1   0
## 7902   7902    0 480 24   1    1  1   1   0
## 7903   7903    0 480 24   1    1  1   1   0
## 7904   7904    0 480 24   1    1  1   1   0
## 7905   7905    0 480 24   1    1  1   1   0
## 7906   7906    0 480 24   1    1  1   1   0
## 7907   7907    0 480 24   1    1  1   1   0
## 7908   7908    0 480 24   1    1  1   1   0
## 7909   7909    0 480 24   1    1  1   1   0
## 7910   7910    0 480 24   1    1  1   1   0
## 7911   7911    0 480 24   1    1  1   1   0
## 7912   7912    0 480 24   1    1  1   1   0
## 7913   7913    0 480 24   1    1  1   1   0
## 7914   7914    0 480 24   1    1  1   1   0
## 7915   7915    0 480 24   1    1  1   1   0
## 7916   7916    0 480 24   1    1  1   1   0
## 7917   7917    0 480 24   1    1  1   1   0
## 7918   7918    0 480 24   1    1  1   1   0
## 7919   7919    0 480 24   1    1  1   1   0
## 7920   7920    0 480 24   1    1  1   1   0
## 7921   7921    0 480 24   1    1  1   1   0
## 7922   7922    0 480 24   1    1  1   1   0
## 7923   7923    0 480 24   1    1  1   1   0
## 7924   7924    0 480 24   1    1  1   1   0
## 7925   7925    0 480 24   1    1  1   1   0
## 7926   7926    0 480 24   1    1  1   1   0
## 7927   7927    0 480 24   1    1  1   1   0
## 7928   7928    0 480 24   1    1  1   1   0
## 7929   7929    0 480 24   1    1  1   1   0
## 7930   7930    0 480 24   1    1  1   1   0
## 7931   7931    0 480 24   1    1  1   1   0
## 7932   7932    0 480 24   1    1  1   1   0
## 7933   7933    0 480 24   1    1  1   1   0
## 7934   7934    0 480 24   1    1  1   1   0
## 7935   7935    0 480 24   1    1  1   1   0
## 7936   7936    0 480 24   1    1  1   1   0
## 7937   7937    0 480 24   1    1  1   1   0
## 7938   7938    0 480 24   1    1  1   1   0
## 7939   7939    0 480 24   1    1  1   1   0
## 7940   7940    0 480 24   1    1  1   1   0
## 7941   7941    0 480 24   1    1  1   1   0
## 7942   7942    0 480 24   1    1  1   1   0
## 7943   7943    0 480 24   1    1  1   1   0
## 7944   7944    0 480 24   1    1  1   1   0
## 7945   7945    0 480 24   1    1  1   1   0
## 7946   7946    0 480 24   1    1  1   1   0
## 7947   7947    0 480 24   1    1  1   1   0
## 7948   7948    0 480 24   1    1  1   1   0
## 7949   7949    0 480 24   1    1  1   1   0
## 7950   7950    0 480 24   1    1  1   1   0
## 7951   7951    0 480 24   1    1  1   1   0
## 7952   7952    0 480 24   1    1  1   1   0
## 7953   7953    0 480 24   1    1  1   1   0
## 7954   7954    0 480 24   1    1  1   1   0
## 7955   7955    0 480 24   1    1  1   1   0
## 7956   7956    0 480 24   1    1  1   1   0
## 7957   7957    0 480 24   1    1  1   1   0
## 7958   7958    0 480 24   1    1  1   1   0
## 7959   7959    0 480 24   1    1  1   1   0
## 7960   7960    0 480 24   1    1  1   1   0
## 7961   7961    0 480 24   1    1  1   1   0
## 7962   7962    0 480 24   1    1  1   1   0
## 7963   7963    0 480 24   1    1  1   1   0
## 7964   7964    0 480 24   1    1  1   1   0
## 7965   7965    0 480 24   1    1  1   1   0
## 7966   7966    0 480 24   1    1  1   1   0
## 7967   7967    0 480 24   1    1  1   1   0
## 7968   7968    0 480 24   1    1  1   1   0
## 7969   7969    0 480 24   1    1  1   1   0
## 7970   7970    0 480 24   1    1  1   1   0
## 7971   7971    0 480 24   1    1  1   1   0
## 7972   7972    0 480 24   1    1  1   1   0
## 7973   7973    0 480 24   1    1  1   1   0
## 7974   7974    0 480 24   1    1  1   1   0
## 7975   7975    0 480 24   1    1  1   1   0
## 7976   7976    0 480 24   1    1  1   1   0
## 7977   7977    0 480 24   1    1  1   1   0
## 7978   7978    0 480 24   1    1  1   1   0
## 7979   7979    0 480 24   1    1  1   1   0
## 7980   7980    0 480 24   1    1  1   1   0
## 7981   7981    0 480 24   1    1  1   1   0
## 7982   7982    0 480 24   1    1  1   1   0
## 7983   7983    0 480 24   1    1  1   1   0
## 7984   7984    0 480 24   1    1  1   1   0
## 7985   7985    0 480 24   1    1  1   1   0
## 7986   7986    0 480 24   1    1  1   1   0
## 7987   7987    0 480 24   1    1  1   1   0
## 7988   7988    0 480 24   1    1  1   1   0
## 7989   7989    0 480 24   1    1  1   1   0
## 7990   7990    0 480 24   1    1  1   1   0
## 7991   7991    0 480 24   1    1  1   1   0
## 7992   7992    0 480 24   1    1  1   1   0
## 7993   7993    0 480 24   1    1  1   1   0
## 7994   7994    0 480 24   1    1  1   1   0
## 7995   7995    0 480 24   1    1  1   1   0
## 7996   7996    0 480 24   1    1  1   1   0
## 7997   7997    0 480 24   1    1  1   1   0
## 7998   7998    0 480 24   1    1  1   1   0
## 7999   7999    0 480 24   1    1  1   1   0
## 8000   8000    0 480 24   1    1  1   1   0
## 8001   8001    0 480 24   1    1  1   1   0
## 8002   8002    0 480 24   1    1  1   1   0
## 8003   8003    0 480 24   1    1  1   1   0
## 8004   8004    0 480 24   1    1  1   1   0
## 8005   8005    0 480 24   1    1  1   1   0
## 8006   8006    0 480 24   1    1  1   1   0
## 8007   8007    0 480 24   1    1  1   1   0
## 8008   8008    0 480 24   1    1  1   1   0
## 8009   8009    0 480 24   1    1  1   1   0
## 8010   8010    0 480 24   1    1  1   1   0
## 8011   8011    0 480 24   1    1  1   1   0
## 8012   8012    0 480 24   1    1  1   1   0
## 8013   8013    0 480 24   1    1  1   1   0
## 8014   8014    0 480 24   1    1  1   1   0
## 8015   8015    0 480 24   1    1  1   1   0
## 8016   8016    0 480 24   1    1  1   1   0
## 8017   8017    0 480 24   1    1  1   1   0
## 8018   8018    0 480 24   1    1  1   1   0
## 8019   8019    0 480 24   1    1  1   1   0
## 8020   8020    0 480 24   1    1  1   1   0
## 8021   8021    0 480 24   1    1  1   1   0
## 8022   8022    0 480 24   1    1  1   1   0
## 8023   8023    0 480 24   1    1  1   1   0
## 8024   8024    0 480 24   1    1  1   1   0
## 8025   8025    0 480 24   1    1  1   1   0
## 8026   8026    0 480 24   1    1  1   1   0
## 8027   8027    0 480 24   1    1  1   1   0
## 8028   8028    0 480 24   1    1  1   1   0
## 8029   8029    0 480 24   1    1  1   1   0
## 8030   8030    0 480 24   1    1  1   1   0
## 8031   8031    0 480 24   1    1  1   1   0
## 8032   8032    0 480 24   1    1  1   1   0
## 8033   8033    0 480 24   1    1  1   1   0
## 8034   8034    0 480 24   1    1  1   1   0
## 8035   8035    0 480 24   1    1  1   1   0
## 8036   8036    0 480 24   1    1  1   1   0
## 8037   8037    0 480 24   1    1  1   1   0
## 8038   8038    0 480 24   1    1  1   1   0
## 8039   8039    0 480 24   1    1  1   1   0
## 8040   8040    0 480 24   1    1  1   1   0
## 8041   8041    0 480 24   1    1  1   1   0
## 8042   8042    0 480 24   1    1  1   1   0
## 8043   8043    0 480 24   1    1  1   1   0
## 8044   8044    0 480 24   1    1  1   1   0
## 8045   8045    0 480 24   1    1  1   1   0
## 8046   8046    0 480 24   1    1  1   1   0
## 8047   8047    0 480 24   1    1  1   1   0
## 8048   8048    0 480 24   1    1  1   1   0
## 8049   8049    0 480 24   1    1  1   1   0
## 8050   8050    0 480 24   1    1  1   1   0
## 8051   8051    0 480 24   1    1  1   1   0
## 8052   8052    0 480 24   1    1  1   1   0
## 8053   8053    0 480 24   1    1  1   1   0
## 8054   8054    0 480 24   1    1  1   1   0
## 8055   8055    0 480 24   1    1  1   1   0
## 8056   8056    0 480 24   1    1  1   1   0
## 8057   8057    0 480 24   1    1  1   1   0
## 8058   8058    0 480 24   1    1  1   1   0
## 8059   8059    0 480 24   1    1  1   1   0
## 8060   8060    0 480 24   1    1  1   1   0
## 8061   8061    0 480 24   1    1  1   1   0
## 8062   8062    0 480 24   1    1  1   1   0
## 8063   8063    0 480 24   1    1  1   1   0
## 8064   8064    0 480 24   1    1  1   1   0
## 8065   8065    0 480 24   1    1  1   1   0
## 8066   8066    0 480 24   1    1  1   1   0
## 8067   8067    0 480 24   1    1  1   1   0
## 8068   8068    0 480 24   1    1  1   1   0
## 8069   8069    0 480 24   1    1  1   1   0
## 8070   8070    0 480 24   1    1  1   1   0
## 8071   8071    0 480 24   1    1  1   1   0
## 8072   8072    0 480 24   1    1  1   1   0
## 8073   8073    0 480 24   1    1  1   1   0
## 8074   8074    0 480 24   1    1  1   1   0
## 8075   8075    0 480 24   1    1  1   1   0
## 8076   8076    0 480 24   1    1  1   1   0
## 8077   8077    0 480 24   1    1  1   1   0
## 8078   8078    0 480 24   1    1  1   1   0
## 8079   8079    0 480 24   1    1  1   1   0
## 8080   8080    0 480 24   1    1  1   1   0
## 8081   8081    0 480 24   1    1  1   1   0
## 8082   8082    0 480 24   1    1  1   1   0
## 8083   8083    0 480 24   1    1  1   1   0
## 8084   8084    0 480 24   1    1  1   1   0
## 8085   8085    0 480 24   1    1  1   1   0
## 8086   8086    0 480 24   1    1  1   1   0
## 8087   8087    0 480 24   1    1  1   1   0
## 8088   8088    0 480 24   1    1  1   1   0
## 8089   8089    0 480 24   1    1  1   1   0
## 8090   8090    0 480 24   1    1  1   1   0
## 8091   8091    0 480 24   1    1  1   1   0
## 8092   8092    0 480 24   1    1  1   1   0
## 8093   8093    0 480 24   1    1  1   1   0
## 8094   8094    0 480 24   1    1  1   1   0
## 8095   8095    0 480 24   1    1  1   1   0
## 8096   8096    0 480 24   1    1  1   1   0
## 8097   8097    0 480 24   1    1  1   1   0
## 8098   8098    0 480 24   1    1  1   1   0
## 8099   8099    0 480 24   1    1  1   1   0
## 8100   8100    0 480 24   1    1  1   1   0
## 8101   8101    0 480 24   1    1  1   1   0
## 8102   8102    0 480 24   1    1  1   1   0
## 8103   8103    0 480 24   1    1  1   1   0
## 8104   8104    0 480 24   1    1  1   1   0
## 8105   8105    0 480 24   1    1  1   1   0
## 8106   8106    0 480 24   1    1  1   1   0
## 8107   8107    0 480 24   1    1  1   1   0
## 8108   8108    0 480 24   1    1  1   1   0
## 8109   8109    0 480 24   1    1  1   1   0
## 8110   8110    0 480 24   1    1  1   1   0
## 8111   8111    0 480 24   1    1  1   1   0
## 8112   8112    0 480 24   1    1  1   1   0
## 8113   8113    0 480 24   1    1  1   1   0
## 8114   8114    0 480 24   1    1  1   1   0
## 8115   8115    0 480 24   1    1  1   1   0
## 8116   8116    0 480 24   1    1  1   1   0
## 8117   8117    0 480 24   1    1  1   1   0
## 8118   8118    0 480 24   1    1  1   1   0
## 8119   8119    0 480 24   1    1  1   1   0
## 8120   8120    0 480 24   1    1  1   1   0
## 8121   8121    0 480 24   1    1  1   1   0
## 8122   8122    0 480 24   1    1  1   1   0
## 8123   8123    0 480 24   1    1  1   1   0
## 8124   8124    0 480 24   1    1  1   1   0
## 8125   8125    0 480 24   1    1  1   1   0
## 8126   8126    0 480 24   1    1  1   1   0
## 8127   8127    0 480 24   1    1  1   1   0
## 8128   8128    0 480 24   1    1  1   1   0
## 8129   8129    0 480 24   1    1  1   1   0
## 8130   8130    0 480 24   1    1  1   1   0
## 8131   8131    0 480 24   1    1  1   1   0
## 8132   8132    0 480 24   1    1  1   1   0
## 8133   8133    0 480 24   1    1  1   1   0
## 8134   8134    0 480 24   1    1  1   1   0
## 8135   8135    0 480 24   1    1  1   1   0
## 8136   8136    0 480 24   1    1  1   1   0
## 8137   8137    0 480 24   1    1  1   1   0
## 8138   8138    0 480 24   1    1  1   1   0
## 8139   8139    0 480 24   1    1  1   1   0
## 8140   8140    0 480 24   1    1  1   1   0
## 8141   8141    0 480 24   1    1  1   1   0
## 8142   8142    0 480 24   1    1  1   1   0
## 8143   8143    0 480 24   1    1  1   1   0
## 8144   8144    0 480 24   1    1  1   1   0
## 8145   8145    0 480 24   1    1  1   1   0
## 8146   8146    0 480 24   1    1  1   1   0
## 8147   8147    0 480 24   1    1  1   1   0
## 8148   8148    0 480 24   1    1  1   1   0
## 8149   8149    0 480 24   1    1  1   1   0
## 8150   8150    0 480 24   1    1  1   1   0
## 8151   8151    0 480 24   1    1  1   1   0
## 8152   8152    0 480 24   1    1  1   1   0
## 8153   8153    0 480 24   1    1  1   1   0
## 8154   8154    0 480 24   1    1  1   1   0
## 8155   8155    0 480 24   1    1  1   1   0
## 8156   8156    0 480 24   1    1  1   1   0
## 8157   8157    0 480 24   1    1  1   1   0
## 8158   8158    0 480 24   1    1  1   1   0
## 8159   8159    0 480 24   1    1  1   1   0
## 8160   8160    0 480 24   1    1  1   1   0
## 8161   8161    0 480 24   1    1  1   1   0
## 8162   8162    0 480 24   1    1  1   1   0
## 8163   8163    0 480 24   1    1  1   1   0
## 8164   8164    0 480 24   1    1  1   1   0
## 8165   8165    0 480 24   1    1  1   1   0
## 8166   8166    0 480 24   1    1  1   1   0
## 8167   8167    0 480 24   1    1  1   1   0
## 8168   8168    0 480 24   1    1  1   1   0
## 8169   8169    0 480 24   1    1  1   1   0
## 8170   8170    0 480 24   1    1  1   1   0
## 8171   8171    0 480 24   1    1  1   1   0
## 8172   8172    0 480 24   1    1  1   1   0
## 8173   8173    0 480 24   1    1  1   1   0
## 8174   8174    0 480 24   1    1  1   1   0
## 8175   8175    0 480 24   1    1  1   1   0
## 8176   8176    0 480 24   1    1  1   1   0
## 8177   8177    0 480 24   1    1  1   1   0
## 8178   8178    0 480 24   1    1  1   1   0
## 8179   8179    0 480 24   1    1  1   1   0
## 8180   8180    0 480 24   1    1  1   1   0
## 8181   8181    0 480 24   1    1  1   1   0
## 8182   8182    0 480 24   1    1  1   1   0
## 8183   8183    0 480 24   1    1  1   1   0
## 8184   8184    0 480 24   1    1  1   1   0
## 8185   8185    0 480 24   1    1  1   1   0
## 8186   8186    0 480 24   1    1  1   1   0
## 8187   8187    0 480 24   1    1  1   1   0
## 8188   8188    0 480 24   1    1  1   1   0
## 8189   8189    0 480 24   1    1  1   1   0
## 8190   8190    0 480 24   1    1  1   1   0
## 8191   8191    0 480 24   1    1  1   1   0
## 8192   8192    0 480 24   1    1  1   1   0
## 8193   8193    0 480 24   1    1  1   1   0
## 8194   8194    0 480 24   1    1  1   1   0
## 8195   8195    0 480 24   1    1  1   1   0
## 8196   8196    0 480 24   1    1  1   1   0
## 8197   8197    0 480 24   1    1  1   1   0
## 8198   8198    0 480 24   1    1  1   1   0
## 8199   8199    0 480 24   1    1  1   1   0
## 8200   8200    0 480 24   1    1  1   1   0
## 8201   8201    0 480 24   1    1  1   1   0
## 8202   8202    0 480 24   1    1  1   1   0
## 8203   8203    0 480 24   1    1  1   1   0
## 8204   8204    0 480 24   1    1  1   1   0
## 8205   8205    0 480 24   1    1  1   1   0
## 8206   8206    0 480 24   1    1  1   1   0
## 8207   8207    0 480 24   1    1  1   1   0
## 8208   8208    0 480 24   1    1  1   1   0
## 8209   8209    0 480 24   1    1  1   1   0
## 8210   8210    0 480 24   1    1  1   1   0
## 8211   8211    0 480 24   1    1  1   1   0
## 8212   8212    0 480 24   1    1  1   1   0
## 8213   8213    0 480 24   1    1  1   1   0
## 8214   8214    0 480 24   1    1  1   1   0
## 8215   8215    0 480 24   1    1  1   1   0
## 8216   8216    0 480 24   1    1  1   1   0
## 8217   8217    0 480 24   1    1  1   1   0
## 8218   8218    0 480 24   1    1  1   1   0
## 8219   8219    0 480 24   1    1  1   1   0
## 8220   8220    0 480 24   1    1  1   1   0
## 8221   8221    0 480 24   1    1  1   1   0
## 8222   8222    0 480 24   1    1  1   1   0
## 8223   8223    0 480 24   1    1  1   1   0
## 8224   8224    0 480 24   1    1  1   1   0
## 8225   8225    0 480 24   1    1  1   1   0
## 8226   8226    0 480 24   1    1  1   1   0
## 8227   8227    0 480 24   1    1  1   1   0
## 8228   8228    0 480 24   1    1  1   1   0
## 8229   8229    0 480 24   1    1  1   1   0
## 8230   8230    0 480 24   1    1  1   1   0
## 8231   8231    0 480 24   1    1  1   1   0
## 8232   8232    0 480 24   1    1  1   1   0
## 8233   8233    0 480 24   1    1  1   1   0
## 8234   8234    0 480 24   1    1  1   1   0
## 8235   8235    0 480 24   1    1  1   1   0
## 8236   8236    0 480 24   1    1  1   1   0
## 8237   8237    0 480 24   1    1  1   1   0
## 8238   8238    0 480 24   1    1  1   1   0
## 8239   8239    0 480 24   1    1  1   1   0
## 8240   8240    0 480 24   1    1  1   1   0
## 8241   8241    0 480 24   1    1  1   1   0
## 8242   8242    0 480 24   1    1  1   1   0
## 8243   8243    0 480 24   1    1  1   1   0
## 8244   8244    0 480 24   1    1  1   1   0
## 8245   8245    0 480 24   1    1  1   1   0
## 8246   8246    0 480 24   1    1  1   1   0
## 8247   8247    0 480 24   1    1  1   1   0
## 8248   8248    0 480 24   1    1  1   1   0
[truncated: 264,298 more chars]
